# All About the Base: Electrochemical Isomerization of Terminal Propargyl Compounds toward the Synthesis of Allenes and Phenols

**DOI:** 10.1021/acs.orglett.6c01122

**Published:** 2026-05-11

**Authors:** Krzysztof Grudzień, Ireneusz Tomczyk, Natalia Kominek, Katarzyna Rybicka-Jasińska

**Affiliations:** Institute of Organic Chemistry, Polish Academy of Sciences, Kasprzaka 44/52, 01-224 Warsaw, Poland

## Abstract

Herein we present a mild, efficient, and selective electrochemical method for terminal allene synthesis through isomerization of propargyl compounds. We postulate that the process occurs through *in situ* electrochemical generation of a strong base. The proposed mechanism is supported by calculations; the reaction thermodynamics together with other computed parameters, combined with kinetic model sensitivity analysis, clarifies the role of the applied current in controlling the reaction pathway. The electrochemical pathway allows for the selective synthesis of not only allenes but also phenols.

## Graphical abstract

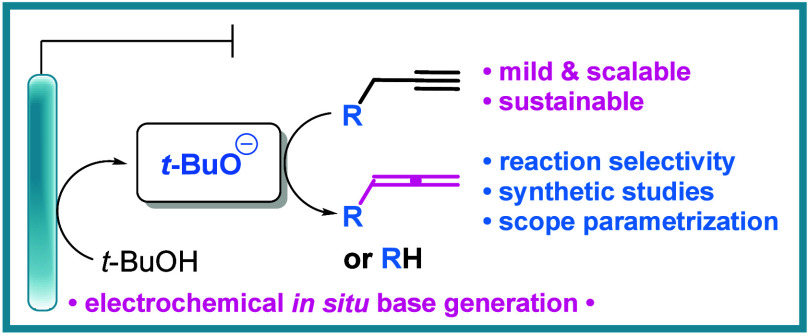

Many conventional synthetic transformations rely on stoichiometric strong bases, which, while essential, are corrosive and air- and moisture-sensitive and pose significant operational and environmental challenges.^1^ Electrochemistry offers a sustainable alternative to conventional synthetic methods by enabling the *in situ* generation of reactive species (e.g., radicals, radical anions and cations, and ionic intermediates) under mild and controllable conditions.^2–10^ While modern synthetic electrochemistry has largely focused on group-transfer reactions or substrate oxidations, the deliberate generation of simple anions for base-promoted transformations remains rather underexplored.^11–14^ Cathodic processes provide a practical platform for *in situ* base formation under exceptionally mild conditions from readily available and nontoxic precursors, eliminating the need for bulk handling of strong bases.^15^

Allenes constitute a unique class of organic compounds characterized by the presence of two cumulated double bonds (C=C=C) within a three-carbon skeleton. These compounds display a distinct spatial geometry and high chemical reactivity, which makes them valuable substrates in organic synthesis.^16,17^ Despite their intriguing structural and electronic properties, the broader synthetic utility of allenes has been limited by challenges associated with their preparation and controlling their reactivity.^18–20^ In recent years, however, interest in allenes as substrates for C–C and C–X bond-forming reactions has increased significantly.^21–24^ This growing interest has created a demand for new, efficient, economical, and selective methods for allene synthesis that facilitate their use in the preparation of high-value compounds, especially under electrochemical conditions.^25–27^ The most straightforward approach to the synthesis of terminal allenes involves base-mediated isomerization of a propargyl fragment, typically employing catalytic or stoichiometric amounts of strong bases such as *t*-BuOK, NaH, or CsOH under anhydrous conditions (Figure 1).^28–37^ However, this strategy suffers from several limitations, including the requirement for highly reactive bases and limited control over selectivity and reactivity, particularly when multiple propargyl moieties are present.

**Figure 1:**
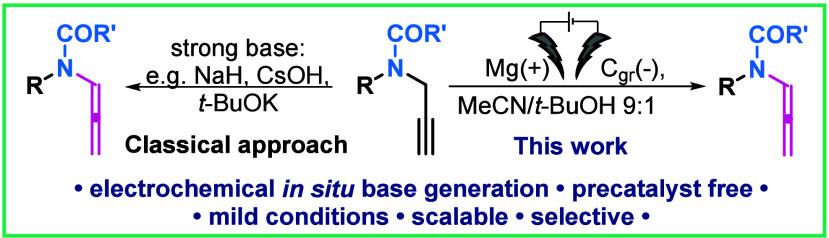
Synthesis of terminal allenes.

An attractive solution to these challenges may lie in the electrochemical *in situ* generation of a strong base capable of catalyzing the isomerization of propargyl compounds into allenes. While the electrochemical possibility of isomerization of propargyl derivatives has been described in analytical studies,^38^ we wondered whether electrochemically *in situ*-generated base would be able to promote terminal allene synthesis. *To the best of our knowledge, such an approach could offer significant advantages in terms of control over selectivity, efficiency, atom economy (through precatalyst-free conditions), scalability, and green chemistry principles* (Figure 1) *and remains unexplored.*

In order to establish the electrochemical isomerization of propargyl amide **1a** to allene moiety **2a**, we initiated our studies by exploring the reactivity of **1a** in the presence of the commonly used electrolyte NBu_4_Cl under action of a constant current (5 mA, 1.2 F/mol). The reaction produced the desired product **2a** in 43% yield as the main product (see the Supporting Information (SI)).

Subsequently, several reaction parameters (solvent, concentration, type of electrodes and electrolytes, time, current density, and applied potential; for details, see the SI) were optimized. Background experiments confirmed that the desired transformation cannot take place without electricity and addition of alcohol (Figure 2, entries 2 and 3). The most effective anode material was magnesium. In the case of the cathode material, efficiency similar to that of graphite was achieved for Ni, stainless steel (SS), and Pt, but taking into consideration cost-effectiveness, we chose to use graphite for further studies. Overall, anodic oxidation with constant current (*I* = 5 mA) of propargyl amide **1a** with a magnesium electrode as the anode and a graphite electrode as the cathode in NBu_4_Cl solution (*c* = 0.05 M) at room temperature for 0.3 F/mol gave allene derivative **2a** in 97% yield.

**Figure 2:**
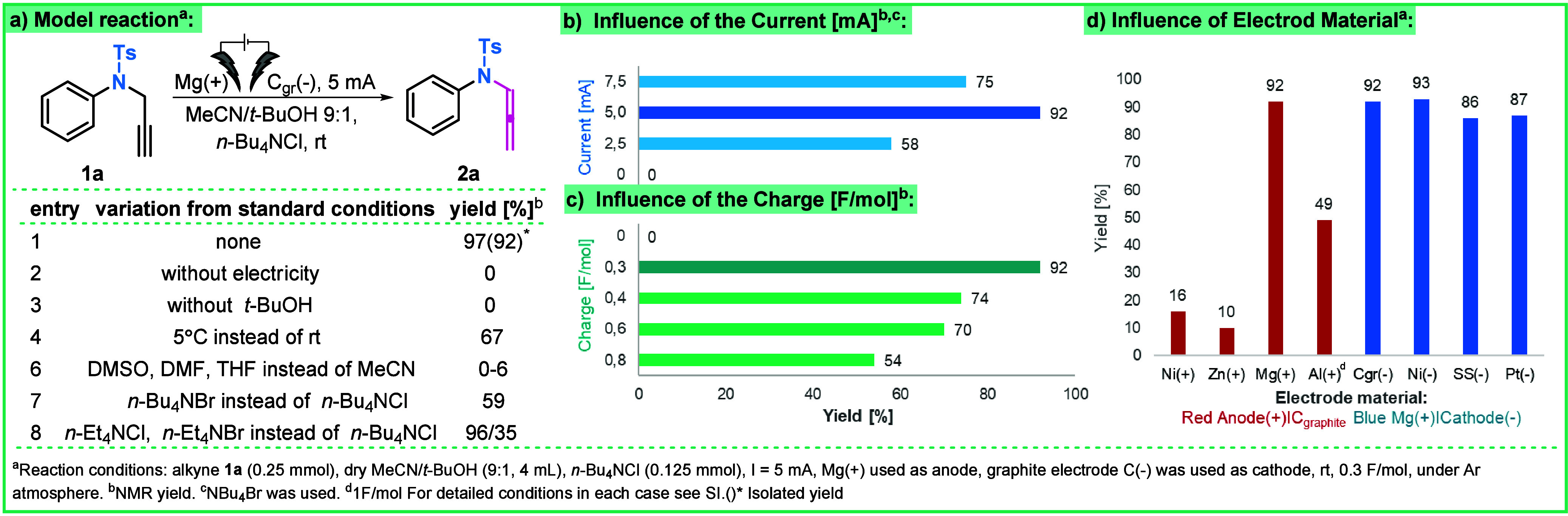
Optimization studies.

During the optimization step we observed that effectiveness of the reaction strongly depends on the current (mA) and the amount of charge that has passed during electrolysis (F/mol) (Figure 2; for details, see the SI). Excess charge applied to model substrate **1a** resulted in decomposition, including possible depropargylation, and formation of byproducts. Equipped with this knowledge and the optimal conditions for the model reaction, we proceeded to investigate the scope and limitations. First we examined a set of different propargyl amides and carbamates (Ts, Ac, Boc, CO_2_Me, COPh) (Figure 3a). The electrochemical allene isomerization was applicable to different amide groups in good to excellent yields (54–92%), with the Ts derivative being the most reactive one (92% for only 0.3 F/mol). However, in comparison to the model compound, for all of the examples higher electrical current and amount of charge were necessary to achieve good effectiveness. Then we successfully tested the aliphatic substituent on the amide group: we first tested simple a Me substituent (**2g**, 72% yield) and then proceeded toward a functionalized melatonin derivative (**2h**, 62%). Both transformations proceeded in good yields (Figure 3a). In general, the reaction proceeds more readily for more acidic substrates, which translates to those forming more stabilized anions (for the calculation details regarding this parameter, see the SI). Although batch electrochemical reactions often face challenges in scale-up, our procedure could be readily applied under increased concentration conditions (e.g., 60% yield of product **2a** on a 1.0 mmol scale).

**Figure 3:**
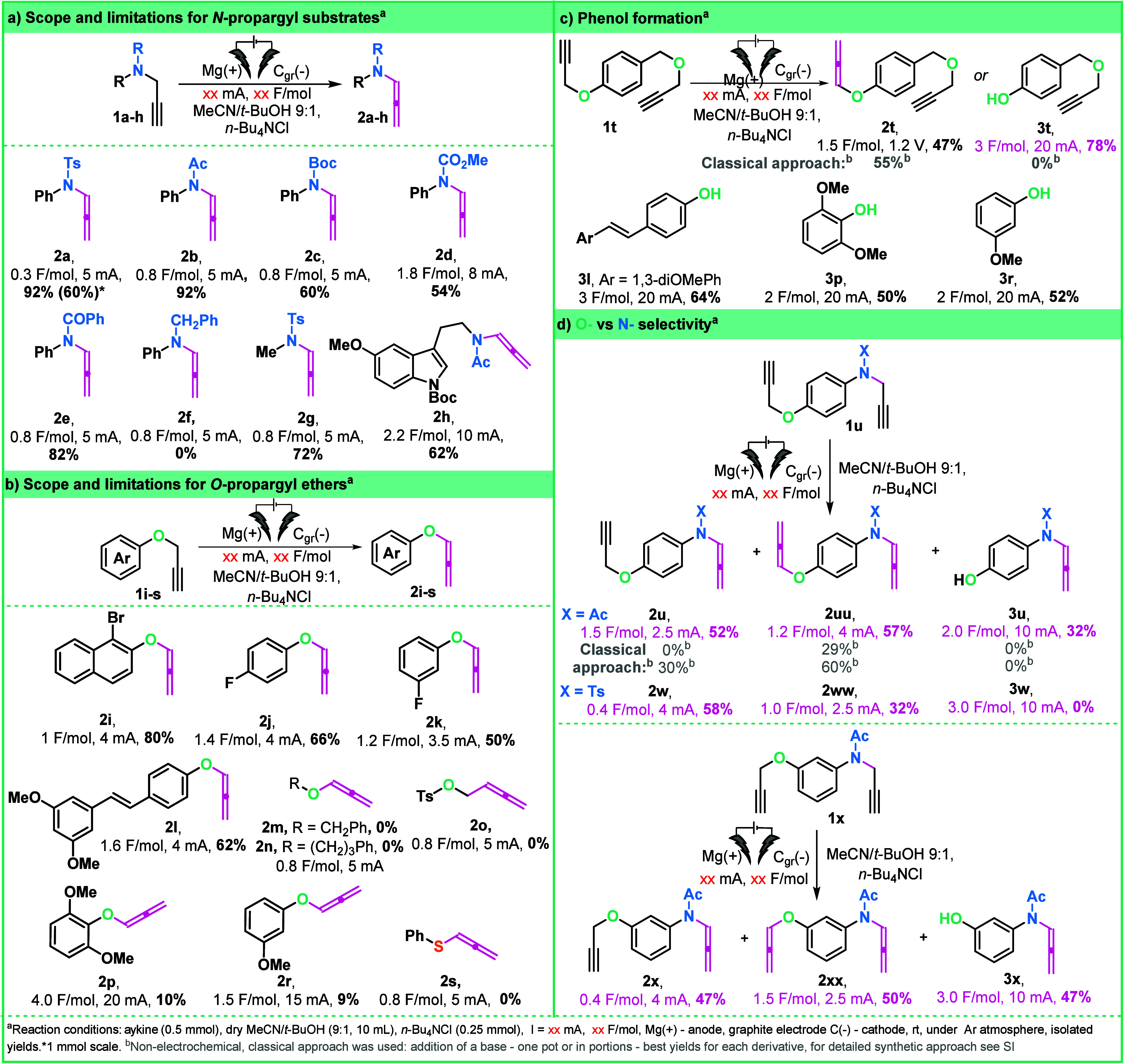
Scope and limitations.

Next, we investigated the scope of propargyl ethers (Figure 3b). For a range of propargylic ethers, including bromo-substituted naphthalene derivative **2i** as well as fluoro- (**2j**, **2k**) and vinyl-substituted (**2l**) substrates, good yields were obtained after brief preliminary optimization of the applied charge and current density (for details, see the SI). In contrast, aliphatic derivatives (**2m**–**2o**) did not undergo the reaction at all. Furthermore, the reaction also did not proceed in the case of sulfur derivatives (**2s**) (for the full list of unsuccessful examples, see the SI). Notably, substrates bearing electron-donating substituents displayed a markedly different reactivity profile: their conversion toward the corresponding allene derivatives was very low, and at higher currents we observed the formation of the corresponding phenols through formal *O*-deprotection.^39^ Intrigued by these results, we investigated the reactivity of four different derivatives, i.e., **1t** possessing two chemically different R–O–propargyl ether substituents (Figure 3c) and **1u**–**1x** possessing both propargyl phenolic ether and propargyl amide moieties within their structures (Figure 3d). Control of our electrochemical approach toward allene formation was achieved by modulating the current intensity and total charge passed, which taking into consideration the classical pathway of this reaction (deprotonation and isomerization) presumably correspond to the amount and rate of base catalyst formation, i.e., the amount and formation rate of base are expected to be key driving forces for the reaction to occur. For substrate **1t**, a brief optimization was carried out for both the electrochemical method (constant current, constant potential, and charge; for details, see the SI) and the classical method (addition of KO*t*Bu in one portion or dropwise addition of its solution over time; see the SI). Under classical conditions, the best results were observed for the formation of the bisallene, which was not detected at all under electrochemical conditions (see the SI). This reflects the requirement for an appropriate rate of base addition to achieve monoallylation. Conversely, none of the classical variants enabled the formation of phenol **3t** (see the SI), whereas the electrochemical method afforded **3t** in up to 78% yield. The synthesis of monoisomerized product **2t** could be achieved in both methods in similar yields, however, the electrochemical setup allows for better control of the selectivity. Furthermore, the electrochemical method exhibits high chemoselectivity, enabling selective deprotection of the propargyl moiety exclusively from aromatic derivatives and leading to phenol formation rather than aliphatic alcohols. Notably, this transformation is inaccessible under classical conditions.^40^ In this part of the study, we tested a few propargyl derivatives toward the synthesis of phenols (**3l**, **3p**, **3r**), and indeed, the reaction proceeded in good yields (52–64%). After successful application of electron-rich propargyl phenolic ethers in the chemoselective electrochemical formal deprotection, we turned our attention toward the study of the reactivity of paracetamol derivatives **1u**, **1w**, and **1x**, which possess both propargyl phenolic ethers and propargylamide moieties within the same structure (Figure 3d). Once again, by the controlled manipulation of various electrochemical parameters, we were able to achieve selective formation of mono- (**2u**, **2w**, **2x**) and bisisomerized products (**2uu**, **2ww**, **2xx**) under milder conditions along with the deprotected product (**3u, 3x**) under harsher electrochemical conditions in good yields. We also compared these results with those for classical addition of the base (see Figure 3c,d and the SI). In summary, the electrochemical method enables access to both mono- and bisfunctionalized products, similar to the classical approach; however, it offers a markedly higher level of control over the reaction outcome. Notably, it enables chemoselective phenol formation through formal deprotection, a transformation that remains inaccessible under classical conditions, especially for substrates bearing multiple propargyl groups. Selective monopropargylation is difficult due to competing multiple substitutions. Our method, demonstrated for substrates **1u** and **1x**, uses the propargyl group as a OH-protecting group. Although *O*-propargylation is easier than *N*-propargylation, the *O*-propargyl group is more electrolabile under our conditions, enabling selective formation of *N*-allenyl phenols (**3u**, **3x**) in a single step from readily obtained, fully propargylated precursors. This also highlights the *O*-propargyl group as a useful electrolabile protecting group for phenols.

Based on the aforementioned results and literature data,^28,41^ we propose a plausible reaction pathway for the electrochemical terminal allene synthesis. We postulate that the process occurs through *in situ* electrochemical generation of a strong base (Figure 4a). During electrolysis, a metal cation–*tert*-butoxide ion pair is generated in a controlled manner. This occurs through the oxidative dissolution of a sacrificial metal electrode, which releases metal cations into the solution while simultaneously producing *tert*-butoxide anions. These anions serve as active catalysts for the isomerization of the propargyl group into the allene functionality. During that part of study we also tested the addition of different additives to the reaction mixture (see the SI). In contrast to basic additives, the presence of an acidic salt strongly suppresses the reaction, suggesting quenching of basic species that are present and required for the reaction to proceed. Furthermore, to address the limited mechanistic insight into propargyl–allene isomerization^41^ and the observed current dependence of the reaction outcome, we performed DFT calculations at the ωB97X-D/def2-TZVP/def2-SVP level of theory (for details, see the SI) to clarify the effect of the base generation rate and define the relevant thermodynamic parameter space, supporting thermodynamic control of the isomerization.^42–44^ We considered a simplified mechanistic pathway (Figure 4b) for model substrate **1a** (denoted **AH**). Deprotonation of **AH** at the terminal (≡CH) group by *t-*BuO^–^ to give the acetylide anion **A** ^–^ is strongly exergonic (−14.3 kcal mol^–1^). Subsequent single proton transfer from the acetylide methylene unit (−CH_2_−) to the formerly deprotonated carbon results in rearrangement to give the higher-energy propargylic anionic intermediate **C** ^–^ (+5.7 kcal mol^–1^), which can evolve to allenyl anion **B** ^–^ (−1.7 kcal mol^–1^ relative to **C** ^–^) by subsequent proton transfer. Final protonation of **B** ^–^ affords the neutral allene **BH** (5.5 kcal mol^–1^ relative to **B** ^–^, −4.8 kcal mol^–1^ relative to **AH**). Within this model, the initially formed anion **A** ^–^ is the thermodynamically most stable species along the computed profile. However, as the reaction is conducted in MeCN/*t*BuOH (9:1), the protic cosolvent is expected to shift the equilibrium toward the lowest energetically neutral species, **BH** (−4.8 kcal/mol relative to **AH**). To verify the steady state of the simplified model and assess the impact of the base generation mode, zero-dimensional kinetic simulations were performed using COPASI (Figure 4c and the SI).^45,46^ In all cases, the final equilibrium was similar, with **BH** as the major species. However, the transient concentration of acetylide **A** ^–^ strongly depended on screened reaction rate constants. A high initial concentration of *t*-BuO^–^ caused short-lived accumulation of **A** ^–^, while slow continuous base formation kept its levels low. The model suggests that high transient anion concentrations may promote competing side reactions such as oxidation or polymerization. Finally, multidimensional analysis of the calculated thermodynamic and redox parameters for 20 substrates revealed that high yields cluster within a defined space governed by the **BH** dissociation energy, **AH** reduction potential, and **B** ^–^ oxidation potential (Figure 4d; also see the SI).^47^ In general, a more negative **BH** dissociation energy increases the concentration of **B** ^–^ (and related anions) and facilitates their oxidation. As **B** ^–^ remained negligible in simulations, its depletion may become relevant at higher transient **A** ^–^ concentrations, while **AH** reduction competes with isomerization and can trigger decomposition. For propargylic phenolic ethers, elevated currents promote depropargylation via low-barrier, highly exergonic C–O bond cleavage after single-electron reduction, whereas the alternative radical cation pathway is energetically disfavored (Figure 4e).

**Figure 4:**
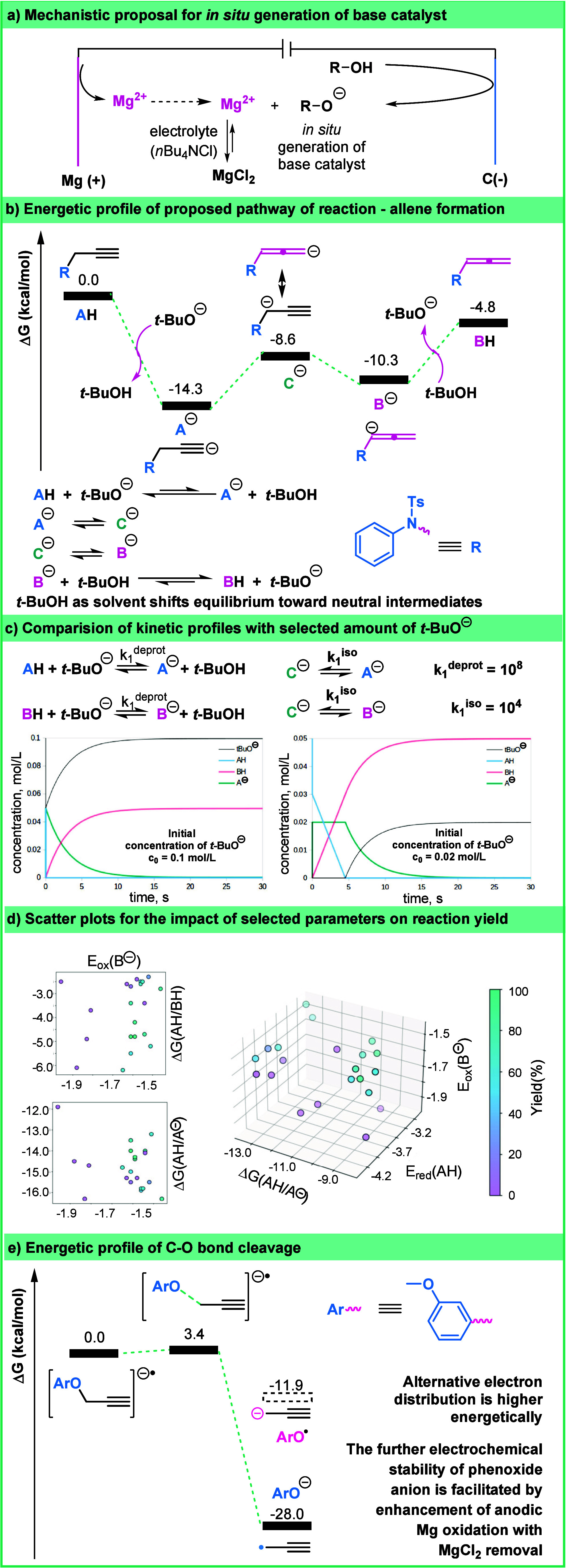
Mechanistic investigation—theoretical studies.

In conclusion, this work describes a mild and efficient electrochemical method for the synthesis of terminal allenes and phenols through isomerization of propargyl amides, carbamates, and phenolic ethers. We postulate that the process occurs through *in situ* electrochemical generation of a strong base. During electrolysis, a *tert*-butoxide anion is generated in a controlled manner from *tert*-butyl alcohol. Controlled manipulation of the electrochemical pathway allowed for not only the selective synthesis of allenes but also the production of formally deprotected products (phenols). Theoretical calculations (with respect to relative redox potentials, propargyl and allene dissociation, or anion isomerization free energy) were combined with kinetic model sensitivity analysis to elucidate the role of the applied current in governing the reaction outcome. Furthermore, the established methodology holds significant potential for application in other base-catalyzed reactions, and preliminary investigations in this area are currently underway in our laboratory.

## Supplementary Material





## Data Availability

The data underlying this study are available in the published article and its Supporting Information and are openly available in RepOD at 10.18150/PR7XSF.

## Notes

Optimization details, experimental procedures, characterization data for all new compounds and theoretical calculation description (PDF)
Computed Cartesian coordinates for optimized structures in a format for convenient visualization (ZIP)
