# Supplementary material for: All About the Base: Electrochemical Isomerization of Terminal Propargyl Compounds toward the Synthesis of Allenes and Phenols
Source: Org Lett. 2026 May 11;28(20):6190–5. doi: 10.1021/acs.orglett.6c01122 (PMC13309000; doi:10.1021/acs.orglett.6c01122)
Supplement: Supplementary file 1 [file ol6c01122_si_001.pdf]

Supporting Information

All About the Base – *e*-Isomerization of Terminal Propargyl Compounds towards Synthesis of Allenes and Phenols

Krzysztof Grudzień[a], Ireneusz Tomczyk[a], Natalia Kominek[a] and Katarzyna Rybicka-Jasińska[a*]

[a]Institute of Organic Chemistry, Polish Academy of Sciences,
Kasprzaka 44/52, 01-224 Warsaw, Poland

e-mail: *katarzyna.rybicka-jasinska@icho.edu.pl

Table of contents:

1. General information **S3**
2. Preparation of starting materials **S4**
3. Electrosynthesis of terminal allenes
–optimization studies, general procedure, scope and limitation studies **S19**
4. Cyclic voltammetry measurements **S39**
5. References **S43**
6. **NMR spectra** **S45**
7. Theoretical considerations and calculations **S76**
7.1. General information **S76**
7.2. Notation for Structures and Thermodynamic Parameters **S77**
7.3. Kinetics simulations for 1a isomerization **S78**
7.4. Thermodynamic scatter plots **S86**
7.5. Thermodynamic considerations **S94**
7.6. Thermodynamic parameters **S97**
7.7. Additional computational data **S100**
7.8. References **S101**
7.9. Energies and Energy Corrections of Stationary Points **S102**

1. General Information

Materials

All solvents and commercially available reagents were purchased (Sigma-Aldrich, TCI, Acros Organics, Thermo Scientific, BLD Pharm) and were used without further purification unless otherwise indicated. Anhydrous solvents were obtained either by passage through an SPScolumn (CH_3CN, THF, DCM) or purchased from Sigma-Aldrich (DMF, DMSO, *t*-BuOH). All deuterated solvents were purchased from Eurisotop. Cautionary notes for hazardous reactants that were used in this study:

***n*-BuLi (*n*-butyllithium):** *very pyrophoric. It must be handled using proper needle and syringe techniques.*
Propargyl bromide: *very toxic, corrosive effects, causes burns to skin and eyes, strong lachrymator*
CH_3I (methyl iodide): *inhalation of vapor causes lung congestion and pulmonary edema, higher concentrations causes rapid narcosis, contact with liquid irritates eyes and burns skin*
***n*-Bu_4NBr (tetrabutylammonium bromide)**: *highly irritant to skin and respiratory system, suspected of damaging fertility and the unborn child*
NaH (sodium hydride): *highly reactive and flammable, pyrophoric in air*
Ac_2O (acetic anhydride): *corrosive, lachrymator*

Experimental procedures

All electrochemical reactions were performed in 5 or 10mL screw-cap glass vials sealed with screw-caps containing rubber septa. Reactions were monitored by thin-layer chromatography (TLC) using 0.20-mm aluminum plates coated with SiO_2 (60F-254) purchased from Merck, visualized with UV light or chemical developers ($KMnO_4$, cerium molybdenum, anisaldehyde). Column chromatography was performed on SiO_2 60 (230-400 mesh) purchased from Merck. All reactions that required heating were performed using metal heat blocks unless stated otherwise.

Instrumentation

NMR spectra were recorded at ambient temperature (unless otherwise stated) on Bruker 400 or 500 MHz and Varian 500 or 600 MHz. Chemical shifts are reported in ppm relative to the tetramethyl silane signal or solvent peak ($CHCl_3$: 7.26 ppm for ^1H and 77.00 ppm for ^{13}C; DMSO-d6: 2.50 ppm for ^1H and 39.52 ppm for ^{13}C). Multiplicities are given as: singlet (s), doublet (d), triplet (t), doublet of doublets (dd), triplet of triplets (tt), quartet (q), multiplet (m), broad singlet (bs).
LR and HRMS Low-resolution mass spectra (LRMS) were recorded on an Applied Biosystems API 365 mass spectrometer using electrospray ionization (ESI) technique. High-resolution mass spectra (HRMS) were recorded on Waters SYNAPT G2-S HDMS instrument using, electrospray ionization (ESI), or atmospheric-pressure chemical ionization (APCI) with time of flight detector (TOF).

Electrochemical setup:

ElectraSyn 2.0 IKA (picture available at the IKA website:https://www.ika.com) with **5** and **10 mL vials** with **caps** and **magnesium**, **graphite** and other **electrodes** (commercially available, purchased from IKA, for technical details see: https://www.ika.com/en/Products-LabEq/Electrochemistry-Kit-pg516/Accessories-cspacc.html). In some cases **BioLogic FC-45** potentiostat was used instead, employing the same vial, cap and electrode setup as with ElectraSyn 2.0.

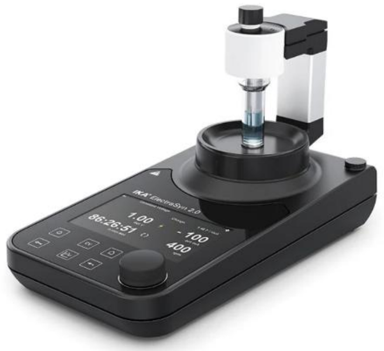

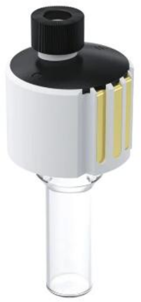

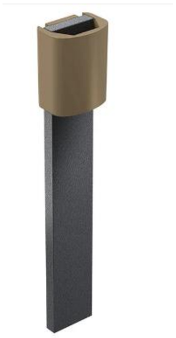

1. Preparation of starting materials

N-propargilation of aniline

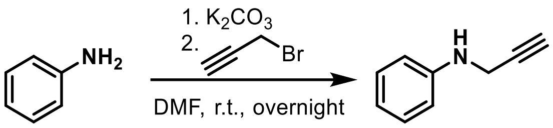

S1

Following a modified literature procedure:[1]

To a solution of aniline (11.20 g, 120 mmol) in DMF (100 mL), K_2CO_3 (8.29 g, 60.0 mmol) was added, then solution of propargyl bromide (3.3 mL, ~9.2M, ~30 mmol, *caution: very toxic, corrosive effects, causes burns to skin and eyes, strong lachrymator*) in DMF (20 mL) was added dropwise (addition time: about 15-20 min) with vigorous stirring (>600 rpm). The reaction was continued overnight, after which water (120 mL) was added. The flask contents were transferred to a 1 L separatory funnel and extracted with petroleum ether / diethyl ether 80:20 (3 × 250 mL). The combined organic phases were washed with brine (200 mL) and dried over anhydrous Na_2SO_4. The drying agent was filtered off, volatiles were removed by rotary evaporation, and the crude product was purified by column chromatography on SiO_2 (petroleum ether / ethyl acetate, 85:15). 3.18 g (24.2 mmol, 81%) of **S1** was obtained as a yellow oil. The reaction was repeated on the same scale to give a yield of 73%.

[1] *Org. Syn.* **2012**, 89, 294-306

N-(Prop-2-yn-1-yl)aniline (**S1**)

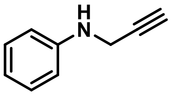

^{1}H NMR (400 MHz, $CDCl_3$) δ 7.32 – 7.12 (m, 2H), 6.86 – 6.74 (m, 1H), 6.74 – 6.61 (m, 2H), 3.95 (bs, 2H), 3.87 (s, 1H), 2.23 (t, *J* = 2.4 Hz, 1H) ppm.
^{13}C NMR (126 MHz, $CDCl_3$) δ 146.8, 129.2, 118.6, 113.5, 81.0, 71.2, 33.6 ppm.
The results of ^{1}H and ^{13}C NMR analysis are in agreement with the literature data.[1]

***N*-tosylation of S1**

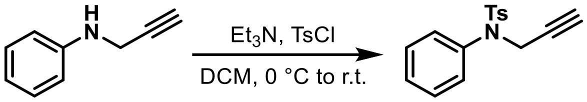

S1 **1a**

Following a literature procedure,[2] on a 25.0 mmol scale of **S1**, 4.40 g (15.4 mmol, 62%) **1a** was obtained as a pale yellow crystalline solid. Reaction repeated twice in 10.0 mmol scale resulted in 74% yield of **1a**.

4-Methyl-*N*-phenyl-*N*-(prop-2-yn-1-yl)benzenesulfonamide (**1a**)

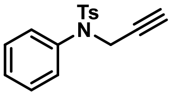

^{1}H NMR (400 MHz, $CDCl_3$) δ 7.54 (d, *J* = 8.3 Hz, 2H), 7.35 – 7.28 (m, 2H), 7.25 – 7.20 (m, 5H), 4.44 (d, *J* = 2.5 Hz, 2H), 2.42 (s, 3H), 2.16 (t, *J* = 2.5 Hz, 1H) ppm.
^{13}C NMR (126 MHz, $CDCl_3$) δ 143.4, 139.0, 135.3, 129.0, 128.7, 128.1, 127.9, 127.7, 76.5, 73.5, 40.7, 21.3 ppm.
The results of ^{1}H and ^{13}C NMR analysis are in agreement with the literature data.[3]

***N*-acylation of S1**

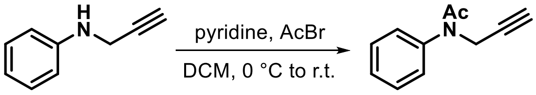

S1 **1b**

A 100 mL round-bottom flask was charged with **S1** (1.31 g, 10.0 mmol), DCM (5 mL) andpyridine (5 mL). The flask was placed in an ice/water bath and AcBr (0.93 mL, 12.5 mmol) was added dropwise. After the addition, the bath was removed and the mixture was stirred overnight at room temperature. Mixture was concentrated using a rotary evaporator, diluted with diethyl ether to approximately 100 mL, transferred to a separatory funnel, washed with dilute HCl_{aq} (3M, 2 × 50 mL) and brine (100 mL),then dried over anhydrous Na_2SO_4. The drying agent was filtered off, volatiles removed by rotary evaporation, and the crude product was purified by column chromatography on SiO_2 (petroleum ether / ethyl acetate, 75:25). 1.21 g (7.0 mmol, 70%) of **1b** was obtained as a white solid.

N-Phenyl-*N*-(prop-2-yn-1-yl)acetamide (**1b**)

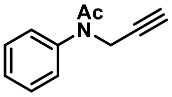

^{1}H NMR (500 MHz, $CDCl_3$) δ 7.44 (dd, *J* = 8.3, 6.7 Hz, 2H), 7.41 – 7.36 (m, 1H), 7.29 (dd, *J* = 8.3, 1.4 Hz, 2H), 4.48 (d, *J* = 2.5 Hz, 2H), 2.20 (t, *J* = 2.4 Hz, 1H), 1.87 (s, 3H) ppm.
^{13}C NMR (126 MHz, $CDCl_3$) δ 170.1, 142.3, 129.7, 128.4, 128.1, 79.2, 72.0, 38.2, 22.4 ppm.
The results of ^{1}H and ^{13}C NMR analysis are in agreement with the literature data.[4]

---

[2] *Chem. Commun.* **2019**,55, 4355-4358
[3] *Dalton Trans.*, **2016**, 45, 1546–1553

N-Boc protection of S1

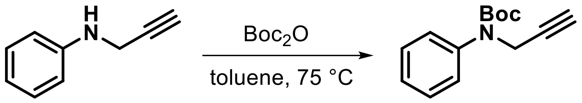

S1 **1c**

A 100 mL round-bottom flask was charged with **S1** (655 mg, 5.0 mmol), toluene (10 mL) and Boc_2O (1.31 g, 6.0 mmol). The contents of the flask were stirred at 75 °C for 15 h. Toluene was removed by evaporation and the crude product was purified by column chromatography on SiO_2 (hexane / diethyl ether, 90:10). 1.15 g (4.83 mmol, 97%) of **1c** was obtained as a colorless oil.

Tert-butyl phenyl(prop-2-yn-1-yl)carbamate (**1c**)

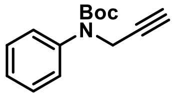

^{1}H NMR (500 MHz, $CDCl_3$) δ 7.38 – 7.30 (m, 4H), 7.22 (m, 1H), 4.37 (d, *J* = 2.5 Hz, 2H), 2.24 (t, *J* = 2.4 Hz, 1H), 1.46 (s, 9H) ppm.
^{13}C NMR (126 MHz, $CDCl_3$) δ 154.0, 142.1, 128.7, 126.28, 126.25, 81.0, 80.0, 71.7, 39.7, 28.2 ppm.
The results of ^{1}H and ^{13}C NMR analysis are in agreement with the literature data.[5]

N-methoxycarbonylation of S1

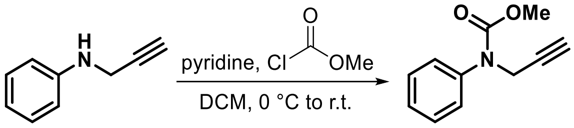

S1 **1d**

A procedure analogous to that for acylation on a scale of 3.0 mmol **S1** gave 2.5 mmol (83%) **S4** as a pale yellow oil.

Methyl phenyl(prop-2-yn-1-yl)carbamate (**1d**)

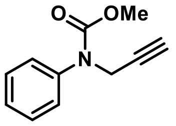

^{1}H NMR (500 MHz, $CDCl_3$) δ 7.75 – 7.69 (m, 2H), 7.66 (m, 2H), 7.65 – 7.61 (m, 1H), 4.75 (d, *J* = 2.5 Hz, 2H), 4.07 (s, 3H), 2.61 (t, *J* = 2.4 Hz, 1H) ppm.
^{13}C NMR (126 MHz, $CDCl_3$) δ 155.6, 141.4, 129.0, 127.2, 126.8, 79.5, 72.2, 53.2, 40.2 ppm.
The results of ^{1}H and ^{13}C NMR analysis are in agreement with the literature data.[6]

N-benzoilation of S1

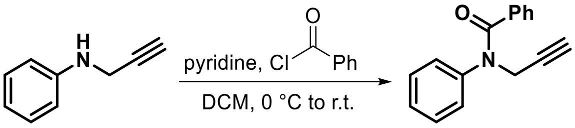

S1 **1e**

A procedure analogous to that for acylation on a scale of 4.8 mmol **S1** gave 3.9 mmol (81%) **1e** as a white solid.

---

[4] *Org. Lett.* **2014***,* 16, 14, 3752–3755
[5] *J. Org. Chem.***1996**, 61, 26, 9168–9177; *Synthesis***2021**, 53, 123–134
[6] *J. Am. Chem. Soc.* **2017***,* 139, 1998–2005

N-Phenyl-*N*-(prop-2-yn-1-yl)benzamide (**1e**)

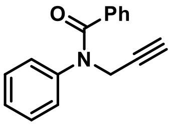

^{1}H NMR (500 MHz, $CDCl_3$) δ 7.36 – 7.30 (m, 2H), 7.28 – 7.21 (m, 3H), 7.21 – 7.09 (m, 5H), 4.68 (d, *J* = 2.5 Hz, 2H), 2.25 (t, *J* = 2.5 Hz, 1H) ppm.
^{13}C NMR (126 MHz, $CDCl_3$) δ 170.1, 142.8, 135.1, 129.9, 129.1, 128.8, 127.69, 127.65, 127.1, 79.0, 72.16, 39.8 ppm.
The results of ^{1}H and ^{13}C NMR analysis are in agreement with the literature data.[4]

N-benzylation of S1

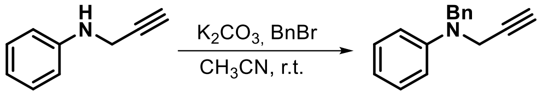

S1 **1f**

A 100 mL round-bottom flask was charged with **1**(656 mg, 5.0 mmol), CH_3CN (10 mL), K_2CO_3 (1.38 g, 10.0 mmol) and finally BnBr (0.89 mL, 7.5 mmol), dropwise. The mixture was stirred overnight at room temperature, then filtered through a pad of celite.The filtrate was concentrated to dryness and the residue was purified by repeated column chromatography on SiO_2 (petroleum ether / ethyl acetate, 95: 5 → 97: 3). 0.89 g (4.0 mmol, 80%) of **1f** was obtained as a colorless oil.

N-Benzyl-*N*-(prop-2-yn-1-yl)aniline (**1f**)

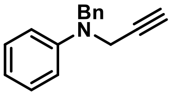

^{1}H NMR (600 MHz, $CDCl_3$) δ 7.42 – 7.38 (m, 4H), 7.36 – 7.30 (m, 3H), 6.98 (d, *J* = 7.8 Hz, 2H), 6.89 (m, 1H), 4.63 (s, 2H), 4.09 (d, *J* = 2.4 Hz, 2H), 2.28 (t, *J* = 2.4 Hz, 1H) ppm.
^{13}C NMR (151 MHz, $CDCl_3$) δ 148.7, 138.4, 129.1, 128. 6, 127.1, 127.1, 118.4, 114.2, 79.7, 72.1, 55.0, 39.8 ppm.
The results of ^{1}H and ^{13}C NMR analysis are in agreement with the literature data.[4]

General procedure for propargylation in acetonitrile using potassium or cesium carbonate (procedure A):

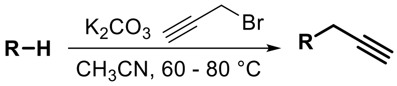

A 100 mL round-bottom flask was charged with R-H (1 equiv.)along with teflon-coated magnetic stirring bar, CH_3CN (HPLC purity, around 3 mL / 1 mmol R-H) was added, then K_2CO_3 (2 equivalents)and propargyl bromide (9.2M solution in toluene, 1.2-1.5 equiv., *caution: very toxic, corrosive effects, causes burns to skin and eyes, strong lachrymator*) were added successively. The flask was sealed with septum and placed in a heatingplate (temperature at 60-80 °C), mixture was stirred intensively (>600 rpm), usually overnight. Volatiles were removed by a rotary evaporation, the residue was purifiedby a column chromatography on SiO_2or a simple filtration through a pad of SiO_2on a Schott funnel.

Synthesis of 1g

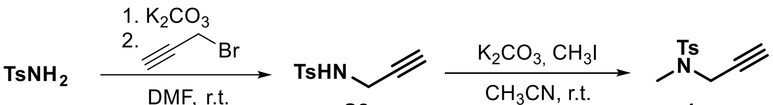

S2 **1g**

Two-step reaction, first step according to a modified procedure A on a scale of 55.2 mmol tosylamide, 36.0 mmol K_2CO_3, 18.4 mmol propargyl bromide and 3.0 mmol ***n*-Bu$_4$NBr** (*caution: highly irritant to skin and respiratory system, suspected of damaging fertility and the unborn child*) carried out overnight at 60 °C. The monopropargylation product was isolated by column chromatography on SiO_2 (petroleum ether / ethyl acetate, 80:20), yielding 1297mg of **S2** (6.20 mmol, 34%) as a white solid that was used in the following reaction.

Second step: **S2** (627 mg, 2.5 mol), K_2CO_3 (1.24 g, 9.0 mmol), and CH_3CN (20 mL) were placed in a 100 mL round-bottomed flask. Next CH_3I (0.37 mL, 6.0 mmol, *caution: inhalation of vapor causes lung congestion and pulmonary edema, higher concentrations causes rapid narcosis, contact with liquid irritates eyes and burns skin*) was added, the flask was sealed with a rubber septum, and the contents were stirred for 18 h at room temperature. After this time, the flask was transferred to a separatory funnel and diluted with a mixture of Et_2O (50 mL) and hexane (25 mL). The organic layer was washed with 10% $NH_4Cl_{aq.}$ (50 mL), water (50 mL), and brine (50 mL) and then dried over anhydrous Na_2SO_4. The drying agent was filtered off, the solvents were removed by rotary evaporation and the crude product was purified by column chromatography on SiO_2 (petroleum ether : ethyl acetate, 80:20). 0.334 g (1.50 mmol, 60%) of**1g** a white solid was obtained.

N,4-Dimethyl-*N*-(prop-2-yn-1-yl)benzenesulfonamide (**1g**)

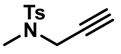

^{1}H NMR (500 MHz, $CDCl_3$) δ 7.70 (d, *J* = 8.3 Hz, 2H), 7.31 (d, *J* = 7.9 Hz, 2H), 4.01 (d, *J* = 2.5 Hz, 2H), 2.82 (s, 3H), 2.43 (s, 3H), 2.08 (t, *J* = 2.5 Hz, 1H) ppm.
^{13}C NMR (126 MHz, $CDCl_3$) δ 143.7, 134.2, 129.5, 127.9, 76.4, 73.9, 39.7, 34.3, 21.5 ppm.
The results of ^{1}H and ^{13}C NMR analysis are in agreement with the literature data.[7]

Synthesis of 1h

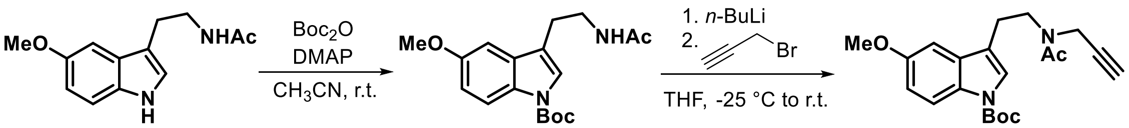

S3 **1h**

Step 1: To a 50 mL round-bottomed flask were added sequentially: melatonin (1161 mg, 5.00 mmol), CH_3CN (20 mL), Boc_2O (1310 mg, 6.00 mmol), and DMAP (24 mg, 0.20 mmol). The mixture was stirred for 20 h at room temperature. The acetonitrile was removed by rotary evaporation, and the crude product was purified by simple filtration through a SiO_2 pad on a Schott funnel (DCM/AcOEt 80:20, large ammount). 1657 mg (4.98 mmol, >99%) of**S3** as a beige solid was obtainedand used entirely in the next reaction. The reaction, repeated on a larger scale (10.0 mmol melatonin), gave a yield of 70% (probably due to incomplete elution of the product from the SiO_2 layer; the use of a more polar eluent is recommended).

Tert-butyl 3-(2-acetamidoethyl)-5-methoxy-1H-indole-1-carboxylate (**S3**)

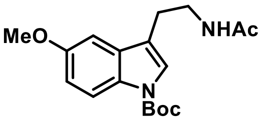

^{1}H NMR (400 MHz, $CDCl_3$) δ 8.00 (s, 1H), 7.39 (m, 1H), 6.99 (d, *J* = 2.5 Hz, 1H), 6.94 (dd, *J* = 9.0, 2.6 Hz, 1H), 5.55 (s, 1H), 3.87 (s, 3H), 3.58 (q, *J* = 6.6 Hz, 2H), 2.88 (t, *J* = 7.1 Hz, 2H), 1.96 (s, 3H), 1.66 (s, 9H) ppm.
^{13}C NMR (151 MHz, $CDCl_3$) δ 170.1, 155.9, 131.2, 123.7, 117.5, 116.1, 113.2, 101.6, 83.5, 55.7, 39.1, 28.2, 25.1, 23.4 ppm.

---

[7] *Dalton. Trans.* **2016**, 45, 1546–1553

The results of ^{1}H and ^{13}C NMR analysis are in agreement with the literature data.[8]

Step 2: To a 100 mL round-bottomed flask under argon **S3** (1.657 g, 4.98 mmol) and anhydrous THF (25 mL)were added. The flask was placed in an EtOH/H_2O/dry ice bath (- 25 °C) and stirred for about 20-25 min before dropwise addition of a solution of *n*-BuLi in hexane (2.5M, 2.2 mL, 5.5 mmol, *caution: very pyrophoric. It must be handled using proper needle and syringe techniques.*). After the addition, the solution was stirred for another 45 min while slowly warming to 0 °C, then a solution of propargyl bromide in toluene (~9.2M, 1 mL, ~9.2 mmol), was added dropwise. Stirring was continued while slowly warming to room temperature overnight. The reaction was quenched with 10% $NH_4Cl_{aq.}$ (50 mL), most of the THF was removed by evaporation, the residue was transferred to a separatory funnel, H_2O (75 mL) and AcOEt (75 mL) were added. The phases were separated, and the aqueous layer was further extracted with AcOEt (75 mL) and $CHCl_3$ (75 mL). All three organic phases were combined and washed with brine (100 mL), then dried over anhydrous Na_2SO_4. The drying agent was filtered off, the solvents were removed by rotary evaporation, and the crude product was purified by column chromatography on SiO_2 (Et_2O). 0.603 g (1.63 mmol, 33%) of **1h** was obtained as a yellow oil. About 1 g of unreacted starting material was also recovered.

Tert-butyl 5-methoxy-3-(2-(*N*-(prop-2-yn-1-yl)acetamido)ethyl)-1H-indole-1-carboxylate (**1h**)

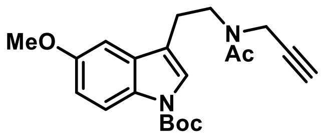

At 25 °C, the ^{1}H NMR spectrum in DMSO and $CHCl_3$ indicates the presence of two rotamers in a ratio of A:B 55:45. Coalescence of the signals in the ^{1}H NMR spectrum in DMSO is observed at approximately 80 °C.

^{1}H NMR (500 MHz, DMSO-d_6, 25 °C) δ 7.99 – 7.83 (m, 1H, both rotamers), 7.54 (s, 1H, rotamer **A**), 7.46 (s, 1H, rotamer **B**), 7.22 (d, *J* = 2.6 Hz, 1H, rotamer **B**), 7.16 (d, *J* = 2.6 Hz, 1H, rotamer **A**), 6.97 – 6.88 (m, 1H, both rotamers), 4.24 (s, 2H, both rotamers), 3.81 (s, 3H, both rotamers), 3.68 – 3.62 (m, 2H, rotamer **A**), 3.61 – 3.54 (m, 2H, rotamer **B**), 3.35 (t, *J* = 2.4 Hz, 1H, rotamer **B**), 3.19 (t, *J* = 2.4 Hz, 1H, rotamer **B**), 2.99 (dd, *J* = 8.5, 6.6 Hz, 2H, rotamer **A**), 2.87 (dd, *J* = 9.3, 6.3 Hz, 2H, rotamer **B**), 2.10 (s, 3H, rotamer **B**), 1.93 (s, 3H, rotamer **B**), 1.62 – 1.59 (two overlapping singlets, 9H, both rotamers) ppm.
^{1}H NMR (500 MHz, DMSO-d_6, 80 °C) δ 7.93 (d, *J* = 8.9 Hz, 1H), 7.48 (s, 1H), 7.17 (s, 1H), 6.94 (dd, *J* = 9.0, 2.5 Hz, 1H), 4.22 (d, *J* = 2.5 Hz, 2H), 3.83 (s, 3H), 3.66 (bs, 2H), 2.97 (bs, 3H), 2.17 – 1.88 (m, 3H), 1.63 (s, 9H) ppm.
^{13}C NMR (126 MHz, DMSO-d_6, 80 °C) δ 169.0, 155.3, 148.6, 130.7, 129.3, 123.4, 117.0, 115.1, 112.5, 102.0, 82.9, 73.8, 73.0, 55.2, 47.1, 45.2, 37.7, 33.3, 27.4, 23.0, 20.6 ppm.
HRMS (ESI-TOF) m/z: $[M + Na]^+$ calculated for $C_{21}H_{26}N_2O_4Na$ $[M + Na]^+$ 393.1790; found 393.1791

Synthesis of 1i

Reaction according to modified procedure**A**on a scale of 5.0 mmol 1-bromo-2-naphthol, 10.0 mmol K_2CO_3, and 6.0 mmol propargyl bromide in DMF solution (5 mL) was carried out for 18 h at 50 °C. After this time, the flask contents were transferred to a separatory funnel and diluted with a mixture of Et_2O (100 mL) and hexane (100 mL). The organic layer was washed with water (3 × 50 mL) and brine (2 × 50 mL). Purification was carried out by simple filtration through a SiO_2 pad on a Schott funnel (eluent: DCM). 1.042 g (3.99 mmol, 80%) of**1i** a brown solid was obtained.

[8]*Green. Chem.* **2017**, 19, 2952–2956

1-Bromo-2-(prop-2-yn-1-yloxy)naphthalene (**1i**)

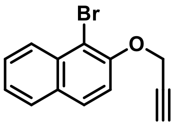

^{1}H NMR (500 MHz, $CDCl_3$) δ 8.25 (dd, *J* = 8.7, 1.1 Hz, 1H), 7.83 – 7.78 (m, 2H), 7.58 (ddd, *J* = 8.4, 6.9, 1.3 Hz, 1H), 7.43 (ddd, *J* = 8.1, 6.8, 1.1 Hz, 1H), 7.40 (d, *J* = 9.0 Hz, 1H), 4.90 (d, *J* = 2.4 Hz, 2H), 2.56 (t, *J* = 2.4 Hz, 1H) ppm.

^{13}C NMR (126 MHz, $CDCl_3$) δ 152.0, 133.1, 130.4, 128.7, 128.0, 127.7, 126.4, 124.8, 115.8, 110.4, 78.3, 76.2, 57.8 ppm.

The results of ^{1}H and ^{13}C NMR analysis are in agreement with the literature data.[9]

Synthesis of 1j

The reaction according to the procedure A was carried out on a scale of 6.0 mmol 4-fluorophenol, 12.0 mmol K_2CO_3, and 9.2 mmol propargyl bromide for 18 h at 60 °C. Purification was carried out by simple filtration through a SiO_2 pad on a Schott funnel (hexane : Et2O 90:10). 0.826 g (5.5 mmol, 92%) of**1j** as a pale yellow oil was obtained.

1-Fluoro-4-(prop-2-yn-1-yloxy)benzene (**1j**)

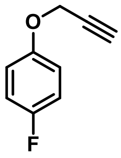

^{1}H NMR (500 MHz, $CDCl_3$) δ 7.03 – 6.96 (m, 2H), 6.96 – 6.90 (m, 2H), 4.66 (d, *J* = 2.5 Hz, 2H), 2.52 (t, *J* = 2.4 Hz, 1H) ppm.

^{13}C NMR (126 MHz, $CDCl_3$) δ 157. 8 (d, *J* = 239.4 Hz), 153.6 (d, *J* = 2.3 Hz), 116.2 (d, *J* = 8.0 Hz), 115.85 (d, *J* = 23.0 Hz), 115.8, 78.4, 75.6, 56.5 ppm.

The results of ^{1}H and ^{13}C NMR analysis are in agreement with the literature data.[10]

Synthesis of 1k

Reaction according to procedure A on a scale of 22.0 mmol 3-fluorophenol, 40.0 mmol K_2CO_3 and 33.0 mmol propargyl bromide for 18 h at 70 °C. Purified by simple filtration through a SiO_2 pad on a Schott funnel (hexane / Et_2O 90:10). 3.024 g (20.1 mmol, 92%) of**1k** as a pale yellow oil was obtained.

1-Fluoro-3-(prop-2-yn-1-yloxy)benzene (**1k**)

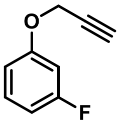

^{1}H NMR (500 MHz, $CDCl_3$) δ 7.30 – 7.17 (m, 1H), 6.79 – 6.74 (m, 1H), 6.74 – 6.66 (m, 2H), 4.68 (d, *J* = 2.5 Hz, 2H), 2.54 (t, *J* = 2.4 Hz, 1H) ppm.

^{13}C NMR (126 MHz, $CDCl_3$) δ 163.5 (d, *J* = 245.8 Hz), 158.8 (d, *J* = 10.6 Hz), 130.2 (d, *J* = 9.7 Hz), 110.6 (d, *J* = 3.4 Hz), 108.4 (d, *J* = 21.3 Hz), 102.8 (d, *J* = 25.3 Hz), 78.0, 75.9, 56.0 ppm.

The results of ^{1}H and ^{13}C NMR analysis are in agreement with the literature data.[10]

Synthesis of 1l

Reaction according to theprocedure A on a scale of 6.0 mmol pterostilbene, 12.0 mmol K_2CO_3, and 9.2 mmol propargyl bromide for 18 h at 70 °C. Purified by simple filtration through a SiO_2 pad on a Schott funnel (eluent: $CHCl_3$). 1.687 g (5.73 mmol, 96%) of**1l** as a white solid was obtained.

[9] *Org. Lett.* **2019***,* 21, 24, 10057–10062
[10] *Bioorg. Chem.* **2021**, 112, 104831

(*E*)-1,3-Dimethoxy-5-(4-(prop-2-yn-1-yloxy)styryl)benzene (**1l**)

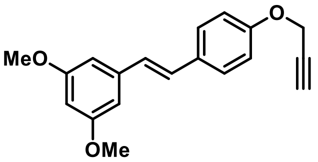

^{1}H NMR (400 MHz, $CDCl_3$) δ 7.46 (d, *J* = 8.8 Hz, 2H), 7.04 (d, *J* = 16.3 Hz, 1H), 6.97 (d, *J* = 8.8 Hz, 2H), 6.91 (d, *J* = 16.2 Hz, 1H), 6.65 (d, *J* = 2.3 Hz, 2H), 6.38 (t, *J* = 2.3 Hz, 1H), 4.71 (d, *J* = 2.4 Hz, 2H), 3.83 (s, 6H), 2.53 (t, *J* = 2.4 Hz, 1H) ppm.

^{13}C NMR (126 MHz, $CDCl_3$) δ 160.9, 157.3, 139.5, 130.8, 128.5, 127.7, 127.0, 115.1, 104.4, 99.7, 78.4, 75.6, 55.8, 55.3 ppm.

The results of ^{1}H and ^{13}C NMR analysis are in agreement with the literature data.[11]

Synthesis of 1m

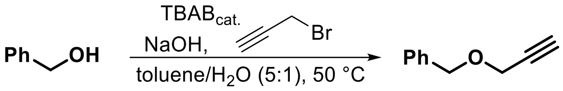

1m

A 50 mL round-bottom flask was charged with propargyl alcohol (336 mg, 6.00 mmol), toluene (5 mL), NaOH (240 mg, 6.00 mmol), ***n*-Bu$_4$NBr** (161 mg, 0.50 mmol), and water (1 mL). The flask was placed in a heating plate (50 °C), and BnBr (0.60 mL, 5.0 mmol) was added dropwise. The mixture was stirred at 50 °C overnight. After cooling down to room temperature, the mixture was transferred to a separatory funnel and diluted with diethyl ether (100 mL). The organic phase was washed with water (3 × 50 mL) and brine (2 × 50 mL), then dried over anhydrous Na_2SO_4. The drying agent was filtered off, the solvents were removed by rotary evaporation, and the crude product was purified by simple filtration through a SiO_2 pad on a Schott funnel (eluent: DCM). 660 mg (4.51 mg, 90%) of**1m** as a colorless oil were obtained.

((Prop-2-yn-1-yloxy)methyl)benzene (**1m**)

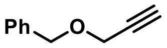

^{1}H NMR (600 MHz, $CDCl_3$) δ 7.41 – 7.29 (m, 5H), 4.62 (s, 2H), 4.18 (d, *J* = 2.4 Hz, 2H), 2.47 (t, *J* = 2.4 Hz, 1H) ppm.

^{13}C NMR (151 MHz, $CDCl_3$) δ 137.3, 128.4, 128.1, 127.9, 79.6, 74.6, 71.5, 57.1 ppm.

The results of ^{1}H and ^{13}C NMR analysis are in agreement with the literature data.[12]

Synthesis of 1n

To a flame-dried round-bottom flask equipped with a magnetic stir bar under an inert atmosphere sodium hydride (NaH, 60% dispersion in mineral oil, 1.2 equiv, 2.4 mmol, 96 mg of dispersion, *caution: highly reactive and flammable, pyrophoric in air*) suspended in anhydrous THF was added. Hydrocinnamyl alcohol (1.0 equiv, 2.0 mmol, 0.27 mL) was added dropwise at room temperature, and the mixture was stirred until the evolution of gas ceased. Propargyl bromide (80 wt% solution in toluene, 1.2 equiv, 2.4 mmol, 0.27 mL) was then added dropwise. After reaction was stirred by 20 hours, the mixture was diluted with Et_2O and quenched with saturated aqueous NH_4Cl. The layers were separated, and the aqueous phase was extracted with Et_2O (3x). The combined organic layers were dried over anhydrous Na_2SO_4, filtered, and concentrated under reduced pressure. Purification by flash column chromatography on silica gel (hexane) afforded propargyl hydrocinnamyl ether**1n** as a colorless oil (250.6 mg, 1.44 mmol, 72%).

---

[11] Patent nr CN119462384A

[12] *Org. Lett.* **2020**, 22, 653–655

(2-(prop-2-yn-1-yloxy)propyl)benzene (**1n**)

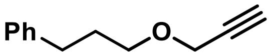

^{1}H NMR (500 MHz, $CDCl_3$) δ 7.34 – 7.27 (m, 2H), 7.25 – 7.17 (m, 3H), 4.16 (dd, J = 2.4, 0.8 Hz, 2H), 3.56 (t, J = 6.4 Hz, 2H), 2.77 – 2.70 (m, 2H), 2.44(m, 1H), 2.00 – 1.90 (m, 2H) ppm.
^{13}C NMR (126 MHz, $CDCl_3$) δ 141.8, 128.4, 128.3, 125.8, 79.9, 74.1, 69.2, 58.0, 32.2, 31.1 ppm.
The results of ^{1}H and ^{13}C NMR analysis are in agreement with the literature data.[13]

Synthesis of 1p

Reaction according to modified procedure A on a scale of 10.0 mmol 2,6-dimethoxyphenol, 15.0 mmol Cs_2CO_3 and 15.0 mmol propargyl bromide in DMF solution (5 mL) was carried out for 18 h at 50 °C. After this time, water (20 mL) was added and the mixture was extracted with diethyl ether (3 × 50 mL), the combined ether layers were washed with water (2 × 50 mL) and brine (50 mL) then dried over anhydrous Na_2SO_4. The drying agent was filtered off, the solvents were removed by rotary evaporation, and the crude product was purified by simple filtration through a SiO_2 pad on a Schott funnel (eluent: $CHCl_3$). 1.857 g (9.76 mmol, 98%) of **1p** as a colorless oil was obtained.

1,3-Dimethoxy-2-(prop-2-yn-1-yloxy)benzene (**1p**)

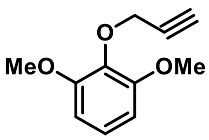

^{1}H NMR (500 MHz, $CDCl_3$) δ 7.01 (t, J = 8.4 Hz, 1H), 6.58 (d, J = 8.4 Hz, 2H), 4.71 (d, J = 2.4 Hz, 2H), 3.85 (s, 6H), 2.43 (t, J = 2.4 Hz, 1H) ppm.
^{13}C NMR (126 MHz, $CDCl_3$) δ 153.7, 135.6, 124.3, 105.2, 79.4, 74.7, 59.9, 56.1 ppm.
HRMS (ESI-TOF) m/z: $[M + Na]^+$ calculated for $C_{11}H_{12}O_3Na$ 215.0684; found 215.0686

Synthesis of 1r

Reaction according to theprocedure A on a scale of 6.0 mmol 3-methoxyphenol, 12.0 mmol K_2CO_3, and 9.2 mmol propargyl bromide was carried out for 18 h at 60 °C. After removal of volatiles on a rotary evaporator, the residue was suspended in a mixture of Et_2O (50 mL) and hexane (25 mL), transferred to a separatory funnel and washed with 10% $NH_4Cl_{aq.}$ (50 mL), water (50 mL), and brine (50 mL), then dried over anhydrous Na_2SO_4. The drying agent was filtered off, volatiles were removed on a rotary evaporator and the crude product was purified by column chromatography on SiO_2 (petroleum ether / ethyl acetate, 90:10). 0.652 g (4.02 mmol, 67%) of**1r** a light yellow oil was obtained.

1-Methoxy-3-(prop-2-yn-1-yloxy)benzene (**1r**)

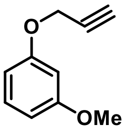

^{1}H NMR (500 MHz, $CDCl_3$) δ 7.20 (ddd, J = 8.5, 7.2, 1.6 Hz, 1H), 6.61 – 6.54 (m, 3H), 4.68 (d, J = 2.4 Hz, 2H), 3.80 (s, 3H), 2.53 (t, J = 2.4 Hz, 1H) ppm.
^{13}C NMR (126 MHz, $CDCl_3$) δ 160.8, 158.8, 129.9, 107.2, 106. 9, 101.5, 78.5, 75.5, 55.8, 55.3 ppm.
The results of ^{1}H and ^{13}C NMR analysis are in agreement with the literature data.[14]

---

[13] *Org. Proc. Res. Develop.* **2018** 22 (11), 1509-1517
[14] *J. Am. Chem. Soc.* **2017**, 139, 11, 4035–4041

Synthesis of 1t

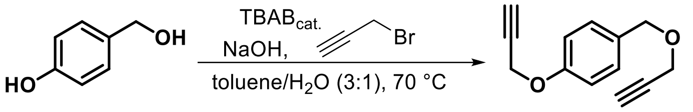

1t

A 100 mL round-bottom flask was charged with 4-hydroxybenzyl alcohol (1.24 g, 10.0 mmol), toluene (15 mL), NaOH (1200 mg, 30.0 mmol), ***n*-Bu$_4$NBr** (322 mg, 1.00 mmol), propargyl bromide (~25.0 mmol) and water (5 mL). The mixture was stirred at 70 °C overnight. After cooling down to room temperature, the mixture was transferred to a separatory funnel and diluted with diethyl ether (150 mL) and chloroform (50 mL). The organic phase was washed with 10% NH_4Cl_{aq}(50 mL) water (50 mL) and brine (50 mL), then dried over anhydrous Na_2SO_4. The drying agent was filtered off, the solvents were removed by rotary evaporation, and the crude product was purified by flash chromarography (SiO_2,petroleum ether : AcOEt 95:5 → 90:10 → 80:20). **1t** as a colorless oil was obtained in 60% yield (avarage of two runs).

1-(Prop-2-yn-1-yloxy)-4-((prop-2-yn-1-yloxy)methyl)benzene (**1t**)

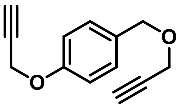

^{1}H NMR (500 MHz, $CDCl_3$) δ 7.30 (d, *J* = 8.5 Hz, 2H), 6.96 (d, *J* = 8.7 Hz, 2H), 4.69 (d, *J* = 2.4 Hz, 2H), 4.55 (s, 2H), 4.15 (d, *J* = 2.4 Hz, 2H), 2.51 (t, *J* = 2.4 Hz, 1H), 2.46 (t, *J* = 2.4 Hz, 1H) ppm.
^{13}C NMR (126 MHz, $CDCl_3$)δ 157.3, 130.3, 129.7, 114.9, 79.7, 78.5, 75.5, 74.5, 71.0, 56.79, 55.8.
HRMS (APCI-TOF, ESI-TOF): decomposition

Synthesis of 1u (two-step procedure)

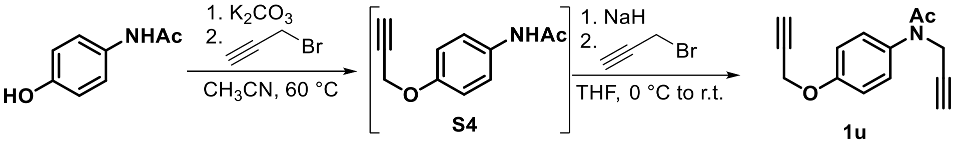

1u

Two-step reaction, step one according to the procedure A on a scale of 10.0 mmol paracetamol, 20.0 mmol K_2CO_3, and 14.0 mmol propargyl bromide was carried out for 18 h at 60 °C. Purification was carried out by simple filtration through a SiO_2 pad on a Schott funnel ($CHCl_3$→$CHCl_3$/MeOH 95:5). 1.89 g (quantitative yield) of the off-white solid **S4**,monopropargylation product, was obtained.It was all used in the following reaction: **S4** was dissolved in anhydrous THF (20 mL), the solution was cooled in an ice/water bath, NaH (600 mg, 15.0 mmol) was added under argon, and after 30 min of stirring, propargyl bromide (80% solution in toluene, 2 mL, 18.4 mmol) was added. The bath was removed, and the reaction was continued overnight at room temperature. The THF was removed by rotary evaporation, the residue was suspended in Et_2O (100 mL), transferred to a separatory funnel and washed with 10% $NH_4Cl_{aq.}$ (100 mL), water (100 mL) and brine (100 mL), then dried over anhydrous Na_2SO_4. The drying agent was filtered off, the solvents were removed by rotary evaporation, and the crude product was purified by column chromatography on SiO_2 (petroleum ether /Et_2O, 4:6). 1.610 g (7.08 mmol, 71%) of **1u** was obtained as an orange, crystalline solid.

Synthesis of 1u (one step, PTC conditions)

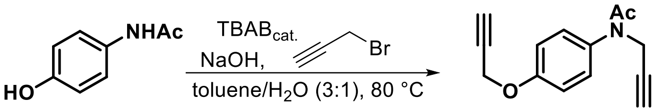

S5 1u

Alternatively **1u** can be synthesized directly from paracetamol in one step via phase-transfer catalysed bispropargylation. A 100 mL round-bottom flask was charged with paracetamol (1.51 g, 10.0 mmol), toluene (15 mL), NaOH (1200 mg, 30.0 mmol), ***n*-Bu$_4$NBr** (322 mg, 1.00 mmol), propargyl bromide (~25.0 mmol) and water (5 mL. The mixture was stirred at 80 °C overnight. After cooling down to room temperature, the mixture was transferred to a separatory funnel and diluted with diethyl ether (150 mL) and chloroform (50 mL). The organic phase was washed with 10% NH_4Cl_{aq}(50 mL) water (50 mL) and brine (50 mL), then dried over anhydrous Na_2SO_4. The drying agent was filtered off, the solvents were removed by rotary evaporation, and the crude product was purified by flash chromarography (SiO_2,n-hexane:Et_2O 75:25 → 50:50 → 25:75 → 0:100). Orange crystalline solid**1u** was obtained in 59% yield (avarage of two runs).

N-(Prop-2-yn-1-yl)-*N*-(4-(prop-2-yn-1-yloxy)phenyl)acetamide (**1u**)

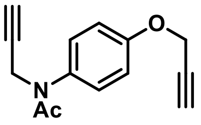

^{1}H NMR (500 MHz, $CDCl_3$) δ 7.20 (d, *J* = 8.9 Hz, 2H), 7.00 (d, *J* = 8.8 Hz, 2H), 4.70 (d, *J* = 2.4 Hz, 2H), 4.42 (d, *J* = 2.5 Hz, 2H), 2.55 (t, *J* = 2.4 Hz, 1H), 2.18 (t, *J* = 2.5 Hz, 1H) ppm.
^{13}C NMR (126 MHz, $CDCl_3$) δ 170.3, 157.3, 135.8, 129.2, 115.7, 79.2, 78.0, 75.9, 71.9, 56.0, 38.2, 22.3 ppm.
HRMS (ESI-TOF) m/z: $[M + H]^+$ calculated for $C_{14}H_{14}NO_2$ 228.1025; found 228.1027

Synthesis of 1w (two steps)

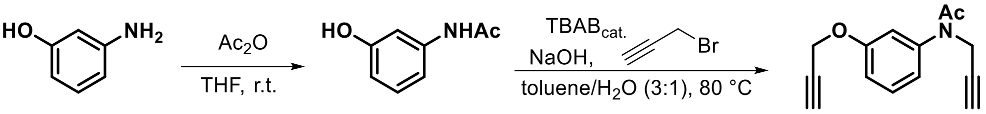

S6 1w

3.64 g (24.1 mmol, 96%) of metacetamol**S6** as a gray powder was obtained from 3-aminophenol (25.0 mmol) following a literature procedure.[18]

N-(3-Hydroxyphenyl)acetamide (**S6**)

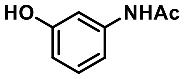

^{1}H NMR (500 MHz, DMSO-d6) δ 9.75 (s, 1H), 9.30 (s, 1H), 7.26 – 7.14 (m, 1H), 7.04 (m, 1H), 6.97 – 6.88 (m, 1H), 6.47 – 6.34 (m, 1H), 2.01 (s, 3H) ppm.
^{13}C NMR (126 MHz, DMSO-d6) δ 168.1, 157.6, 140.4, 129.3, 110.1, 109.8, 106.2, 24.0 ppm.
The results of ^{1}H and ^{13}C NMR analysis are in agreement with the literature data.[15]

A 100 mL round-bottom flask was charged with **S6** (1.51 g, 10.0 mmol), toluene (15 mL), NaOH (1200 mg, 30.0 mmol), ***n*-Bu$_4$NBr** (322 mg, 1.00 mmol), propargyl bromide (~25.0 mmol) and water (5 mL). The mixture was stirred at 70 °C overnight. After cooling down to room temperature, the mixture was transferred to a separatory funnel and diluted with diethyl

[15] *J. Am. Chem. Soc.* **2019**, 141, 15, 6352–6361

ether (150 mL) and chloroform (50 mL). The organic phase was washed with 10% NH_4Cl_{aq} (50 mL) water (50 mL) and brine (50 mL), then dried over anhydrous Na_2SO_4. The drying agent was filtered off, the solvents were removed by rotary evaporation, and the crude product was purified by flash chromarography (SiO_2, petroleum ether : AcOEt 75:25 → 60:40 → 40:60). 1.64 g (7.22 mmol, 72%) of **1w** as an orange oil was obtained.

N-(Prop-2-yn-1-yl)-N-(3-(prop-2-yn-1-yloxy)phenyl)acetamide (**1w**)

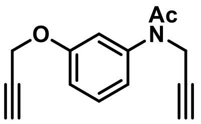

^{1}H NMR (500 MHz, $CDCl_3$) δ 7.34 (m, 1H), 7.03 – 6.94 (m, 1H), 6.94 – 6.80 (m, 2H), 4.70 (d, *J* = 2.4 Hz, 2H), 4.44 (d, *J* = 2.5 Hz, 2H), 2.53 (t, *J* = 2.4 Hz, 1H), 2.19 (t, *J* = 2.6 Hz, 1H), 1.88 (s, 3H) ppm.
^{13}C NMR (126 MHz, $CDCl_3$) δ 169.9, 158.3, 143.3, 130.3, 121.0, 114.9, 114.84, 79.1, 78.0, 76.0, 71.9, 55.9, 38.1, 26.8, 22.3 ppm.
HRMS (APCI-TOF) m/z: $[M + H]^+$ calculated for $C_{14}H_{14}NO_2$ 228.1025; found 228.1022

Synthesis of 1x (two steps)

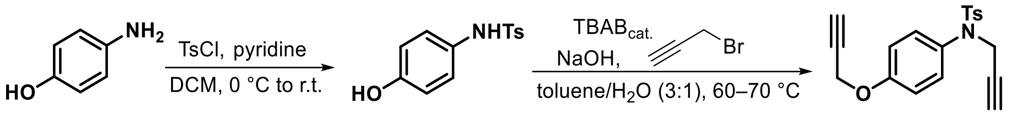

S7 1x

4.08 g (15.5 mmol, 78%) of **S7** as an off-white crystalline solid was obtained from 4-aminophenol (20.0 mmol) following a literature.[16]

N-(4-Hydroxyphenyl)-4-methylbenzenesulfonamide (**S7**)

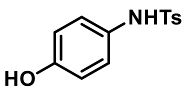

^{1}H NMR (500 MHz, $CDCl_3$) δ 7.60 – 7.51 (m, 2H), 7.23 – 7.19 (m, 2H), 6.96 – 6.85 (m, 2H), 6.76 – 6.59 (m, 2H), 6.24 (s, 1H), 4.94 (s, 1H), 2.39 (s, 3H) ppm.
^{13}C NMR (126 MHz, $CDCl_3$) δ 154.2, 143.7, 136.0, 129.5, 128.9, 127.3, 125.9, 116.0, 21.5 ppm.
The results of ^{1}H and ^{13}C NMR analysis are in agreement with the literature data.[17]

A 100 mL round-bottom flask was charged with **S7** (2.63 g, 10.0 mmol), toluene (15 mL), NaOH (1200 mg, 30.0 mmol), ***n*-Bu$_4$NBr** (322 mg, 1.00 mmol), propargyl bromide (~25.0 mmol) and water (5 mL). The mixture was stirred at 70 °C overnight. After cooling down to room temperature, the mixture was transferred to a separatory funnel and diluted with diethyl ether (150 mL) and chloroform (50 mL). The organic phase was washed with 10% NH_4Cl_{aq} (50 mL) water (50 mL) and brine (50 mL), then dried over anhydrous Na_2SO_4. The drying agent was filtered off, the solvents were removed by rotary evaporation, and the crude product was purified by flash chromarography (SiO_2, petroleum ether : AcOEt 90:10 → 80:20 → 70:30). 1.73 g (7.22 mmol, 72%) of **1x** as an orange oil (that solidified overnight) was obtained.

Reaction was repeated in 5.00 mmol scale of **S7**, with 3 equiv. of propargyl bromide, 4 equiv. of NaOH and 0.15 equiv. of ***n*-Bu$_4$NBr**. Reaction temperature: 60 °C, time: 20 h. 1.55 g (4.57 mmol, 91%) of **1x** as an yellow, crystalline solid was obtained.

[16] *Tetrahedron*, **2021**, 77, 131742
[17] *Adv. Synth. Catal.* **2025**, 367, e202401349.

4-Methyl-N-(prop-2-yn-1-yl)-N-(4-(prop-2-yn-1-yloxy)phenyl)benzenesulfonamide (**1x**)

^{1}H NMR (500 MHz, $CDCl_3$) δ 7.55 (d, *J* = 8.4 Hz, 2H), 7.23 (d, *J* = 8.0 Hz, 2H), 7.14 (d, *J* = 9.0 Hz, 2H), 6.89 (d, *J* = 9.0 Hz, 2H), 4.67 (d, *J* = 2.4 Hz, 2H), 4.40 (d, *J* = 2.4 Hz, 2H), 2.53 (t, *J* = 2.4 Hz, 1H), 2.41 (s, 3H), 2.16 (t, *J* = 2.5 Hz, 1H) ppm.

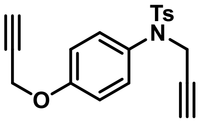

^{13}C NMR (126 MHz, $CDCl_3$)δ 157.3, 143.6, 135.6, 132.6, 130.0, 129.2, 128.0, 115.1, 78.1, 75.8, 73.8, 55.9, 41.3, 21.5 ppm.

HRMS (APCI-TOF) m/z: $[M + H]^+$ calculated for $C_{20}H_{17}NO_3S$ 340.1007; found 340.1005

Synthesis of **S8**

Reaction according to the procedure A was carried out on a scale of 10.0 mmol 4-hydroxy-3-methoxybenzaldehyde (vanillin), 20.0 mmol K_2CO_3, and 13.0 mmol propargyl bromide for 18 h at 70 °C. Purification was carried out by simple filtration through a SiO_2 pad on a Schott funnel (eluent: $CHCl_3$). 1.65 g (8.7 mmol, 87%) of**S8** as a pale yellow crystalline solid was obtained.

3-Methoxy-4-(prop-2-yn-1-yloxy)benzaldehyde (**S8**)

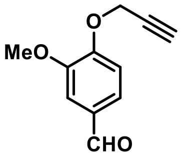

^{1}H NMR (500 MHz, $CDCl_3$) δ 9.85 (s, 1H), 7.44 (dd, *J* = 8.2, 1.9 Hz, 1H), 7.41 (d, *J* = 1.8 Hz, 1H), 7.12 (d, *J* = 8.2 Hz, 1H), 4.84 (d, *J* = 2.4 Hz, 2H), 3.92 (s, 3H), 2.56 (t, *J* = 2.4 Hz, 1H) ppm.

^{13}C NMR (126 MHz, $CDCl_3$) δ 190.8, 152.1, 150.0, 130.9, 126.2, 112.5, 109.4, 77.40, 76.6, 56.5, 56.0 ppm.

The results of ^{1}H and ^{13}C NMR analysis are in agreement with the literature data.[18]

Synthesis of S9

Reaction according to the procedure A on a scale of 6.0 mmol N-Boc-tyrosine, 12.0 mmol K_2CO_3, and 9.2 mmol propargyl bromide was carried out for 18 h at 70 °C. Purification was carried out by column chromatography on SiO_2 (petroleum ether:ethyl acetate, 80:20). 1.980 g (5.94 mmol, 99%) of **S9** as a colorless, crystalline solid was obtained.

Methyl 2-((tert-butoxycarbonyl)amino)-3-(4-(prop-2-yn-1-yloxy)phenyl)propanoate (**S9**)

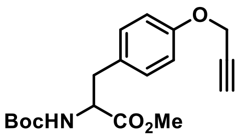

^{1}H NMR (500 MHz, $CDCl_3$) δ 7.05 (d, *J* = 8.5 Hz, 2H), 6.90 (d, *J* = 8.6 Hz, 2H), 5.03 – 4.90 (m, 1H), 4.66 (d, *J* = 2.4 Hz, 2H),4.59 – 4.47 (m, 1H), 3.70 (s, 3H), 3.14 – 2.90 (m, 2H), 2.51 (t, *J* = 2.4 Hz, 1H), 1.41 (s, 9H) ppm.

^{13}C NMR (126 MHz, $CDCl_3$)) δ 172.3, 156.6, 155.1, 130.3, 129.0, 114.9, 79.9, 78.5, 75.5, 55.8, 54.5, 52.2, 37.5, 28.3 ppm.

The results of ^{1}H and ^{13}C NMR analysis are in agreement with the literature data.[19]

Synthesis of **S10**

Reaction according to the procedure A was carried out on a scale of 6.0 mmol 7-hydroxycoumarin (umbelliferone), 12.0 mmol K_2CO_3 and 9.2 mmol propargyl bromide for 18 h at 70 °C. Purification was carried out by simple filtration through a SiO_2 pad on a Schott funnel (eluent: $CHCl_3$). 0.785 g (3.92 mmol, 65%) of **S10** a yellow oil was obtained.

[18] *Inorg. Chem.* **2021**, 60, 24, 18715–18725

[19] *Molecules* **2024**, 29, 8, 1845

7-(Prop-2-yn-1-yloxy)-2H-chromen-2-one (**S10**)

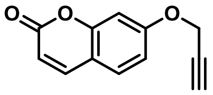

^{1}H NMR (500 MHz, $CDCl_3$) δ 7.64 (d, *J* = 9.5 Hz, 1H), 7.40 (d, *J* = 8.5 Hz, 1H), 6.94 (d, *J* = 2.4 Hz, 1H), 6.91 (dd, *J* = 8.5, 2.5 Hz, 1H), 6.27 (d, *J* = 9.5 Hz, 1H), 4.76 (d, *J* = 2.4 Hz, 2H), 2.57 (t, *J* = 2.5 Hz, 1H)ppm.
^{13}C NMR (126 MHz, $CDCl_3$) δ 161.00, 160.5, 155.6, 143.2, 128.8, 113.7, 113.2, 113.1, 102.1, 77.3, 76.5, 56.2 ppm.
The results of ^{1}H and ^{13}C NMR analysis are in agreement with the literature data.[20]

Synthesis of **S12** (two step procedure)

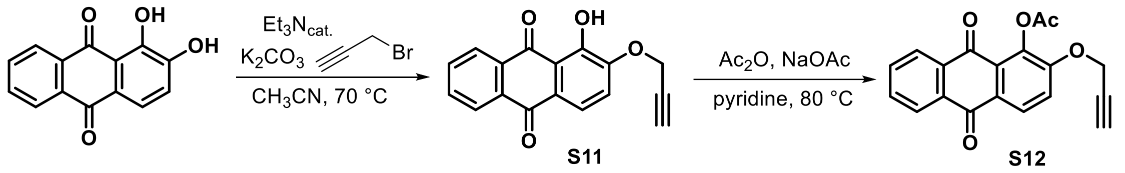

Step 1: Reaction according to the procedure **A**was carried out on a scale of 4.0 mmol 1,2-dihydroxyanthraquinone (alizarin), 4.5 mmol K_2CO_3, and 4.6 mmol propargyl bromide (plus a catalytic amount of triethylamine, ~0.2 mL) for 65 h at 70 °C. Purification was carried out by column chromatography over SiO_2 (eluent: $CHCl_3$). 0.532 g (1.91 mmol, 48%) of **S11** as an orange solid was obtained.

1-Hydroxy-2-(prop-2-yn-1-yloxy)anthracene-9,10-dione (**S11**)

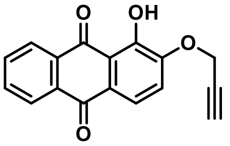

^{1}H NMR (500 MHz, $CDCl_3$) δ 13.01 (s, 1H), 8.41 – 8.23 (m, 2H), 7.88 (dd, *J* = 8.4, 1.1 Hz, 1H), 7.84 – 7.76 (m, 2H), 7.36 (d, *J* = 8.4 Hz, 1H), 4.93 (d, *J* = 2.5 Hz, 2H), 2.59 (t, *J* = 2.3 Hz, 1H) ppm.
^{13}C NMR (126 MHz, $CDCl_3$) δ 189.1, 181.5, 153.2, 151.6, 134.8, 134.0, 133.9, 133.3, 127.4, 126.9, 126.4, 120.5, 118.7, 116.5, 77.03, 56.89 ppm.
The results of ^{1}H and ^{13}C NMR analysis are in agreement with the literature data.[21]

Step 2:S11 (389 mg, 1.40 mmol) was placed in a 100 mL round-bottomed flask, pyridine (7 mL), acetic anhydride (0.70 mL, *caution: corrosive, lachrymator*) and reacts violently with water to form acetic acid), and NaOAc (246 mg, 3.00 mmol) were added. The flask was sealed with a rubber septum, and the mixture wasa stirred on air for 4 hours at 80 °C. After cooling down to room temperature, the mixture was poured into dilute HCl_{aq} (2M, 100 mL), transferred into a separatory funnel and extracted with chloroform (3 × 30 mL). The combined organic layers were dried over anhydrous Na_2SO_4. The drying agent was filtered off, the solvents were removed by rotary evaporation, and the crude product was purified by column chromatography on SiO_2 ($CHCl_3$/ hexane, 9: 1→1: 0). 0.367 g (1.15 mmol, 82%) of**S12**as a yellow solid was obtained. Alternatively, this acylation can be performed in pure acetic anhydride, 5 mL / 0.75 mmol of substrate + 1.0 mmol NaOAc was stirred for 18 h at 80 °C. After cooling down to room temperature, the mixture was poured into water (50 mL), transferred into a separatory funnel and extracted with DCM (2× 50 mL).The combined organic layers were washed with saturated $NaHCO_{3aq}$ (2×50 mL) and brine. After drying over anhydrous Na_2SO_4, filtration and concentration to dryness, 190 mg (0.59 mmol, 79%) of **S12** as a yellow solid was obtained.

[20]*J. Org. Chem.***2021**, 86, 3, 2772–2783
[21]*Phys. Chem. Chem. Phys.* **2014**, 16, 10059–10074

9,10-Dioxo-2-(prop-2-yn-1-yloxy)-9,10-dihydroanthracen-1-yl acetate (**S12**)

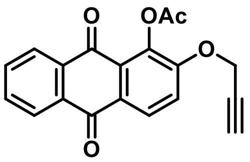

^{1}H NMR (500 MHz, $CDCl_3$) δ 8.29 (d, *J* = 8.8 Hz, 1H), 8.27 – 8.15 (m, 2H), 7.80 – 7.70 (m, 2H), 7.49 (d, *J* = 8.7 Hz, 1H), 4.85 (d, *J* = 2.4 Hz, 2H), 2.60 (t, *J* = 2.4 Hz, 1H), 2.51 (s, 3H) ppm.
^{13}C NMR (126 MHz, $CDCl_3$) δ 181.9, 181.7, 168.9, 155.0, 139.6, 134.4, 134.0, 133.9, 132.9, 127.8, 127.1, 127.0, 126.9, 126.1, 118.1, 77.23, 76.9, 56.9, 20.9 ppm.
HRMS(ESI-TOF) m/z: $[M + Na]^+$ calculated for $C_{19}H_{12}O_5Na$ 343.0582; found 343.0581

Synthesis of S13

Reaction according to the procedure **A** on a scale of 2.5 mmol α-tocopherol, 5.0 mmol K_2CO_3, and 4.6 mmol propargyl bromide (plus a catalytic amount of ***n*-Bu$_4$NBr**, 0.50 mmol) in toluene (10 mL) for 65 h at 80 °C. Purification by column chromatography on SiO_2 (hexane/$CHCl_3$, 95:5→90:10). 0.840 g (1.79 mmol, 72%) of **S13** as a pale yellow oil.

(R)-2,5,7,8-Tetramethyl-6-(prop-2-yn-1-yloxy)-2-((4R,8R)-4,8,12-trimethyltridecyl)chromane (**S13**)

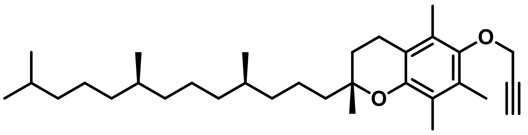

^{1}H NMR (500 MHz, $CDCl_3$) δ 4.37 (d, *J* = 2.4 Hz, 2H), 2.58 (t, *J* = 6.9 Hz, 2H), 2.50 (t, *J* = 2.5 Hz, 1H), 2.21 (s, 3H), 2.18 (s, 3H), 2.09 (s, 3H), 1.87 – 1.70 (m, 2H), 1.63 – 1.25 (m, 15H), 1.24 (s, 3H), 1.22 – 1.01 (m, 5H), 0.90 – 0.82 (m, 13H) ppm.
^{13}C NMR (126 MHz, $CDCl_3$) δ 148.2, 147.9, 127.9, 126.0, 123.0, 117.6, 79.8, 74.9, 74.5, 60.5, 40.1, 39.4, 37.6, 37.5, 37.4, 37.3, 32.8, 32.7, 31.3, 31.2, 28.0, 24.8, 24.4, 23.9, 22.7, 22.6, 21.0, 20.6, 19.7, 19.6, 13.1, 12.2, 11.8 ppm.
The results of ^{1}H and ^{13}C NMR analysis are in agreement with the literature data.[22]

Synthesis of S14

Estrone (540.7 mg, 1.0 equiv, 2.0 mmol) and K_2CO_3 (552.8 mg, 2.0 equiv, 4.0 mmol) were placed in a 25 mL round-bottom flask and dissolved in MeCN (5 mL). Propargyl bromide (80 wt% in toluene, 1.5 equiv, 3.0 mmol, 0.33 mL) was added dropwise at room temperature, and the reaction mixture was stirred at room temperature for 17 h. The mixture was diluted with EtOAc and quenched with water. The layers were separated, and the aqueous phase was extracted 3 times with EtOAc. The combined organic layers were dried over anhydrous Na_2SO_4, filtered, and concentrated under reduced pressure. The crude residue was purified by column chromatography (petroleum ether/EtOAc = 20:1) to afford 3-O-propargyl estrone as white solid (419.4 mg, 1.36 mmol, 68%).

(8R,9S,13S,14S)-13-methyl-3-(prop-2-yn-1-yloxy)-6,7,8,9,11,12,13,14,15,16-decahydro-17H-cyclopenta[a]phenanthren-17-one (**S14**)

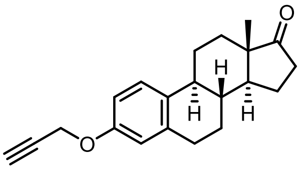

^{1}H NMR (400 MHz, $CDCl_3$) δ 7.22 (d, *J* = 8.6 Hz, 1H), 6.78 (dd, *J* = 8.6, 2.8 Hz, 1H), 6.72 (d, *J* = 2.8 Hz, 1H), 4.66 (d, *J* = 2.4 Hz, 2H), 2.98 – 2.79 (m, 2H), 2.59 – 2.42 (m, 2H), 2.45 – 2.27 (m, 1H), 2.31 – 2.20 (m, 1H), 2.22 – 2.08 (m, 1H), 2.13 – 1.89 (m, 3H), 1.70 – 1.36 (m, 6H), 0.91 (s, 3H) ppm.

[22]*Bioorg. Med. Chem.* **2021**, 40, 127907

^{13}C NMR (101 MHz, $CDCl_3$) δ 155.5, 137.8, 132.9, 126.3, 114.9, 112.3, 78.8, 75.3, 55.7, 50.4, 47.9, 43.9, 38.3, 35.8, 31.5, 29.6, 26.5, 25.8, 21.6,13.8 ppm.
The results of ^{1}H and ^{13}C NMR analysis are in agreement with the literature data.[23]

2. Electrosynthesis of terminal allenes

3.1. Optimization studies.

Table 1. Optimization studies for the synthesis of **2a** (part 1)

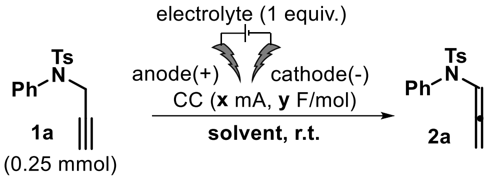

Entry	Solvent	Electrolyte	Current [mA]	Charge [F/mol]	Conversion[a] [%]	Yield[a] [%]
1	EtOH, 99.9%	*n*-Bu_4NBr	4	1.2	25	0
2	**CH_3CN^b/*t*BuOH, (9:1)**	*n*-Bu_4NBr	5	1.2	72	33
3	CH_3CN^b/*t*BuOH, (9:1)	$LiOCl_4$	5	1.2	0	0
4	CH_3CN^b/*t*BuOH, (9:1)	*n*-Bu_4NCl	5	1.2	>95	54
5	CH_3CN^b/*t*BuOH, (9:1)	*n*-Bu_4NI	5	1.2	>90	60
6	**CH_3CN^c/*t*BuOH, (9:1)**	*n*-Bu_4NBr	5	1.2	>95	43
7	**THF^c/*t*BuOH, (9:1)**	LiCl	5	2.0	0	0
8	CH_3CN^b/*t*BuOH, (9:1)	*n*-Bu_4NBr^d	2.5	1.0	>95	50
9	**CH_3CN^c/*t*BuOH, (9:1)**	*n*-Bu_4NBr^d	**5**	**1.0**	**95**	**63**

Standard reaction conditions: **S1** (0.25 mmol), electrolyte (commercial, >98% purity, 0.25 mmol, 1.0 equiv.), solvent (c = 0.05M), Mg(+)|(-)graphite, constant current (x mA, y F/mol), undivided cell, ElectraSyn 2.0 IKA potentiostat, argon, r.t.[a]based on ^{1}H NMR analysis using 1,3,5-trimethoxybenzene as an internal standard [b]HPLC grade solvent [c]anhydrous solvent [d]additional purification by heating overnight at 110 °C under high vacuum (oil pump)

Table 2. Optimization studies for the synthesis of **2a** (part 2)

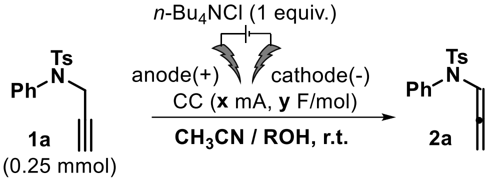

Entry	ROH	Anode(+)	Cathode(-)	Current [mA]	Charge [F/mol]	Conversion[a] [%]	Yield[a] [%]
1	***t*BuOH**	**Mg**	**graphite**	4	1.2	**95**	**63**
2	*t*BuOH	Mg	graphite	5	1.2	0	0
3	*t*BuOH	Mg	**Steel**	5	1.2	84	32
4	*t*BuOH	**Zn**	graphite	5	1.2	57	22
5	tBuOH	**C-graphite**	graphite	5	1.2	0	0
6	*t*BuOH	Mg	graphite	5	1.2	75	34
7	**MeOH**	Mg	graphite	5	2.0	0	0
8	**EtOH**	Mg	graphite	2.5	1.0	0	0

[23]*Eur. J. Org. Chem.***2019**, 1166-1169

9[b]	**tBuOH**	**Mg**	**graphite**	**5**	**1.0**	**64**	**64**

Standard reaction conditions: **S1** (0.25 mmol), electrolyte (dried at high temperature under high vacuum overnight, 0.25 mmol, 1.0 equiv.), solvent (0.05M):anhydrous CH_3CN (4.5 mL) + added anhydrous alcohol (0.5 mL), anode(+)|(-)cathode, constant current (x mA, y F/mol),undivided cell, ElectraSyn 2.0 IKA potentiostat, argon,r.t.[a]based on ^1H NMR analysis using 1,3,5-trimethoxybenzene as an internal standard [b]Reaction was ran in the fridge (around 5 °C)

Table 3. Opimization studies for the synthesis of **2a** (part 3)

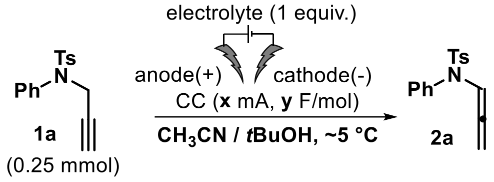

Entry	Electrolyte	Anode(+)	Cathode(-)	Current [mA]	Charge[F/mol]	Conversion[a] [%]	Yield[a] [%]
**1**	***n*-Bu$_4$NBr**	**Mg**	**graphite**	**5**	**1.0**	**64**	**64**
**2**	***n*-Bu$_4$NBr**	Mg	graphite	5	**0.5**	63	63
**3**	***n*-Bu$_4$NBr**	Mg	**Ni-foam**	5	0.5	57	57
**4**	***n*-Bu$_4$NBr**	Mg	**glassy carbon**	5	0.5	6	60
**5**	***n*-Bu$_4$NBr**	Mg	**BDD**	5	0.5	68	45
**6**	***n*-Bu$_4$NCl**	Mg	graphite	5	0.5	79	69
**7**	***n*-Bu$_4$NCl**	Mg	graphite	5	**0.8**	82	82
**8**	***n*-Bu$_4$NCl**	**Mg[b]**	graphite	5	0.8	**97**	**97**
**9**	***n*-Bu$_4$NCl**	**Mg[b]**	graphite	5	**1.0**	97	70
**10**	***n*-Bu$_4$NCl**	**Mg[b]**	**glassy carbon**	5	0.8	98	64

Standard reaction conditions: **S1** (0.25 mmol), electrolyte (dried at high temperature under high vacuum overnight, 0.25 mmol, 1.0 equiv.), solvent (0.05M): anhydrous CH_3CN (4.5 mL) + anhydrous *t*BuOH (0.5 mL), anode (+)|(-) cathode, constant current (x mA, y F/mol), undivided cell, ElectraSyn 2.0 IKA potentiostat, argon,r.t.[a]based on ^1H NMR analysis using 1,3,5-trimethoxybenzene as an internal standard [b]Mg electrodes were additionaly activated by soaking them in diluted HCl_{aq} before reaction

Table 4. Opimization studies for the synthesis of **P1** (part 4)

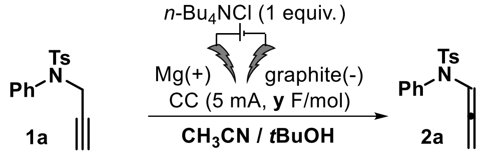

Entry	Temperature	Scale	Charge [F/mol]	Conversion[a] [%]	Yield[a,b] [%]
**1**	**fridge (around 5 °C)**	**0.25 mmol**	**0.8**	**97**	**97 (85)**
**2**	fridge (around 5 °C)	0.25 mmol	**0.5**	58	53
**3**	**r.t. (around 25 °C)**	0.25 mmol	**0.8**	98	70
**4**	r.t. (around 25 °C)	0.25 mmol	**0.5**	n.d.	(87)
**5[c]/6[c]/7[c]**	r.t. (around 25 °C)	0.25 mmol	**0.4**	93/95/>99	89/87/>99
**8[d]**	r.t. (around 25 °C)	0.25 mmol	**0.4**	95	77
**9[e]**	**r.t. (around 25 °C)**	**0.25 mmol**	**0.3**	**100**	**97**
**10[e]**	r.t. (around 25 °C)	**0.50 mmol**	0.4	100	(74)
**11[e]**	r.t. (around 25 °C)	0.50 mmol	**0.6**	100	(71)
**12[e]**	r.t. (around 25 °C)	0.50 mmol	**0.3**	100	(92)
**13[e]**	**fridge (around 5 °C)**	**1.0 mmol**	**0.8**	96	80

Standard reaction conditions: **S1**, electrolyte (dried at high temperature under high vacuum overnight, 1.0 equiv.), solvent (0.05M): anhydrous CH_3CN (4.5 mL) + anhydrous *t*BuOH (0.5 mL), Mg(+)|(-)graphite, constant current (5 mA, y F/mol), Mg electrodes were additionaly activated by soaking them in diluted HCl_{aq} before reaction, undivided cell, ElectraSyn 2.0 IKA potentiostat, argon[a]based on ^{1}H NMR analysis using 1,3,5-trimethoxybenzene as an internal standard[b]isolated yield in parentheses[c]repeated reaction, deviation of the results may be the result of wear of the sacrificial magnesium electrodes [d]BioLogicpotentiostat instead of ElectraSyn 2.0 IKA[e]fresh magnesium electrodes activated by soaking them in diluted HCl_{aq}before reaction

Table 5. Variation of the optimized conditions

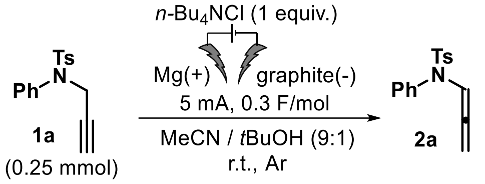

Entry	Variation from standard conditions	Yield[a] [%]
**1**	None	97 (100)
**2**	Without electricity	0 (0)
**3**	0.125 mmol*n*Bu_4NCl	79 (100)
**4**	0.5 mmol*n*Bu_4NCl	74 (100)
**5**	*n*Bu_4NBr instead of *n*Bu_4NCl	45 (45)
**6**	*n*Bu_4NI instead of *n*Bu_4NCl	46 (46)
**7**	*n*Et_4NCl instead of *n*Bu_4NCl	95 (95)
**8**	nEt_4NBr instead of *n*Bu_4NCl	35 (52)
**9**	Al(+) instead of Mg(+)	14 (17)
**10**	Al(+) instead of Mg(+), 8 mA, 1.0 F/mol	49 (77)
**11**	Zn(+) instead of Mg(+)	10 (13)
**12**	Ni foam(+) instead of Mg(+)	16 (29)
**13**	Glassy carbon(-) instead of graphite(-)	92 (100)
**14**	Pt(-) instead of grpahite(-)	87 (100)
**15**	Stainless steel(-) instead of graphite(-)	86 (100)
**16**	Ni foam(-) instead of graphite (-)	93 (100)
**17**	Addition of TEMPO (0.25 equiv.)	80 (100)
**18**	Addition of TEMPO (2 equiv.)	86 (97)
**19**	Addition of NH_4Cl (2 equiv.)	16 (18)
**20**	Addition of Na_2SO_4 (2 equiv.)	89 (94)
**21**	Addition of K_2CO_3 (2 equiv.)	79 (93)
**22**	On air	44 (48)

[a]**Standard reaction conditions:1a** (0.25 mmol), nBu_4NCl (0.25 mmol), MeCN (4.5 mL), *t*BuOH (0.5 mL), I = 5 mA, q = 0.3 F/mol, Mg used as an anode(+), graphite used as cathode(-), undivided cell, IKA Electrasyn 2.0 potentiostat, rt, under Ar atmosphere. [b] NMR yield of **2a**, conversion of **1a** in parentheses.

General procedure for the electrosynthesis of terminal allenes (procedure B)

Alkyne substrate (usually 0.25 to 0.50 mmol) was weighed into a dedicated vial (5 or 10 mL capacity). A stock solution of ***n*-Bu$_4$NCl** (1 equiv.) in a mixture of anhydrous CH_3CN (4.5 mL / 0.25 mmol) and anhydrous *t*-BuOH (0.5 mL / 0.25 mmol) was added. The vial was sealed with a cap equipped with electrodes: magnesium (working anode) and graphite (counter electrode), distance between electrodes: ~5 mm. Magnesium electrodes were activated beforehand by soaking them in diluted HCl_{aq}(1-2M) for 15-60 seconds, then washed with deionized water and acetone and dried. The atmosphere inside the vial was made inert by passing a stream of argon from a balloon through a needle inserted into the septum of the cap. The vial was connected to the IKA Electrasyn 2.0 potentiostat, and electrolysis was performed at a constant current mode (in some cases constant potential without the reference electrode) by passing an appropriate electron charge and stirring the vial contents at 800 rpm using a cylindrical, Teflon-coated magnetic stirring bar (10 mm length for 5 mL vial, 15 mm length for 10 mL vial) in an undivided cell. After completion of the electrolysis, the reaction mixture was transferred to a separatory funnel, diluted with diethyl ether (approximately 50 mL), carefully rinsing the magnetic stirring bar, reaction vessel, and electrodes (the latter in an ultrasonic bath). 10% NH_4Cl_{aq}. (50 mL) and in some casesthe internal standard (1,3,5-trimethoxybenzene, 0.333 equiv.) was added. The phases were separated, the organic layer was washed with additional water (50 mL) and brine (50 mL), then dried over anhydrous Na_2SO_4.The drying agent was filtered off, the solvents were removed by evaporation and the crude product was analyzed by ^{1}H NMR to investigate the conversion of substrate as well as yield of the product and/or purified by (flash) chromatography on SiO_2 to isolate the pure allene.

3.2. Scope and limitations studies

Reactivity of 1a

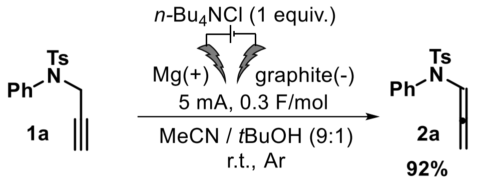

Run 1: Following the general procedure B in CC (constant current) mode (5 mA, 0.3F/mol) on a 0.5 mmol scale of **1a**: 130 mg (0.92 mmol, 92%) of **2a** as a pale yellow crystalline solid was obtained.
Run 2: HPLC grade CH_3CN was used as a solvent. 60% yield of **2a** was obtained.
Run 3: Following the general procedure B in CC (constant current) mode (5 mA, 0.3F/mol) on a 1.0 mmol scale of **1a** and its concentration 0.1M instead of 0.05M: 170 mg (0.60 mmol, 60%) of **2a** as a white crystalline solid was obtained.

4-Methyl-*N*-phenyl-*N*-(propa-1,2-dien-1-yl)benzenesulfonamide (**2a**)

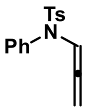

^{1}H NMR (500 MHz, $CDCl_3$) δ 7.56 (d, *J* = 8.3 Hz, 2H), 7.34 – 7.22 (m, 5H), 7.11 (t, *J* = 6.3 Hz, 1H), 7.01 (dd, *J* = 7.8, 1.9 Hz, 2H), 5.03 (d, *J* = 6.3 Hz, 2H), 2.44 (s, 3H) ppm.
^{13}C NMR (126 MHz, $CDCl_3$) δ 201.1, 143.9, 137.2, 135.4, 129.6, 129.5, 128.7, 128.6, 127.7, 102.4, 87.4, 21.57 ppm.
The results of ^{1}H and ^{13}C NMR analysis are in agreement with the literature data.[24]

Reactivity of 1b

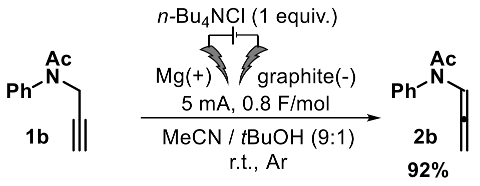

Run 1: Following the general procedure B in CC mode (5 mA, 0.8 F/mol) on a 0.5 mmol scale of **1b**: 80 mg (0.46 mmol, 92%) of **2b** as a colorless oil was obtained.
Run 2: CC (5 mA, 0.6 F/mol): 86% isolated yield of **2b**.
Run 3: CC (5 mA, 0.4 F/mol): 78% isolated yield of **2b**.

N-Phenyl-*N*-(propa-1,2-dien-1-yl)acetamide (**2b**)

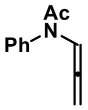

^{1}H NMR (400 MHz, $CDCl_3$) δ 7.66 (t, *J* = 6.5 Hz, 1H), 7.42 – 7.33 (m, 3H), 7.15 (d, *J* = 7.0 Hz, 2H), 4.96 (d, *J* = 6.4 Hz, 2H), 1.87 (s, 3H) ppm.
^{13}C NMR (126 MHz, $CDCl_3$) δ 202.6, 168.5, 140.1, 129.4, 128.4, 100.9, 86.3, 29.7, 22.9 ppm.
The results of ^{1}H and ^{13}C NMR analysis are in agreement with the literature data.[25]

[24]*Org. Lett.* **2012**, 14, 2, 436–439
[25]*Chem. Eur. J.* **2018**, 24, 53, 14059–14063

Reactivity of 1c

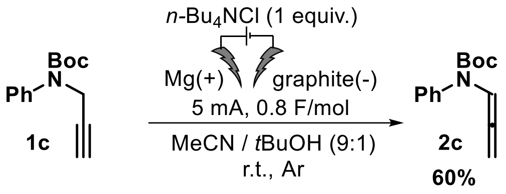

Run 1: Following the general procedure B in CC mode (5 mA, 0.8F/mol) on a 0.5 mmol scale of **1c**: 70 mg (0.30 mmol, 60%) of **2c** as a colorless oil was obtained.

Tert-butyl phenyl(propa-1,2-dien-1-yl)carbamate (**2c**)

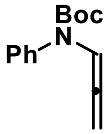

^{1}**H NMR** (400 MHz CDCl$_3$) δ 7.39 – 7.30 (m, 2H), 7.29 – 7.23 (m, 2H), 7.20 – 7.12 (m, 2H), 5.02 (d, *J* = 6.5 Hz, 2H), 1.44 (s, 9H) ppm.

^{13}**C NMR** (126 MHz, CDCl$_3$) δ 201.6, 152.4, 139.3, 128.5, 128.1, 127.1, 102.1, 86.3, 81.4, 28.2 ppm.

The results of ^{1}H and ^{13}C NMR analysis are in agreement with the literature data.[26]

Reactivity of 1d

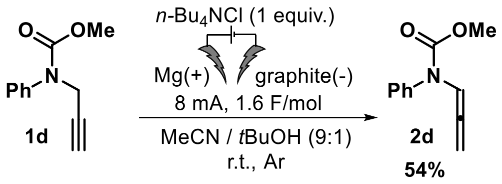

Run 1: Following the general procedure B in CC (constant current) mode (8 mA, 1.6F/mol) on a 0.5 mmol scale of **1d**: 51 mg (0.27 mmol, 54%) of **2d** as a colorless oil was obtained.
Run 2:CC (5 mA,0.8 F/mol) charge was passed: 6% isolated yield of **2d**.
Run 3:CC (5 mA, 0.3 F/mol) charge was passed: 4% isolated yield of **2d**.

Methyl phenyl(propa-1,2-dien-1-yl)carbamate (**2d**)

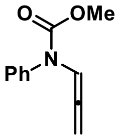

^{1}**H NMR** (500 MHz, CDCl$_3$) δ 7.40 – 7.34 (m, 2H), 7.34 – 7.27 (m, 2H), 7.23 – 7.15 (m, 2H), 5.03 (d, *J* = 6.4 Hz, 2H), 3.73 (s, 3H) ppm.

^{13}**C NMR** (126 MHz, CDCl$_3$) δ 201.6, 153. 9, 138.7, 128.7, 128.1, 127.6, 102.3, 86.8, 53.3 ppm.

The results of ^{1}H and ^{13}C NMR analysis are in agreement with the literature data.[27]

Reactivity of 1e

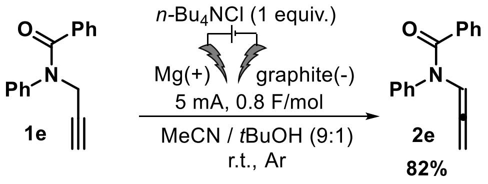

Run 1: Following the general procedure B in CC (constant current) mode (5 mA, 0.8F/mol) on a 0.5 mmol scale of **1e**: 95 mg (0.41 mmol, 82%) of **2e** as a yellow solid was obtained.

[26] *Org. Lett.* **2025**, 27, 9, 2053–2059
[27] *Org. Lett.* **2007**, 9, 23, 4821–4824

Run 2:CC (5 mA 0.3 F/mol): 62% isolated yield of **2e**.

N-phenyl-*N*-(propa-1,2-dien-1-yl)benzamide (**2e**)

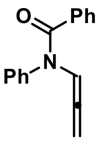

^{1}H NMR (500 MHz, $CDCl_3$) δ 7.57 (t, *J* = 6.3 Hz, 1H), 7.27 (d, *J* = 6.9 Hz, 2H), 7.21 – 7.07 (m, 6H), 7.05 – 6.98 (m, 2H), 5.00 (d, *J* = 6.3 Hz, 2H).
^{13}C NMR (126 MHz, $CDCl_3$) δ 202.8, 168.5, 140.3, 135.1, 130.0, 129.23, 129.17, 128.81, 128.77, 128.6, 128.5, 127.8, 127.5, 102.0, 86.7 ppm.
The results of ^{1}H and ^{13}C NMR analysis are in agreement with the literature data.[25]

Reactivity of 1f

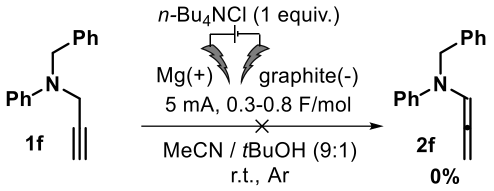

Run 1: Following the general procedure B in CC (constant current) mode (5 mA, 0.3 F/mol) on a 0.5 mmol scale of **1f**: minimal conversion of propargylamide, no allene formation observed
Run 2:CC (5 mA 0.8 F/mol): no allene formation observed

Reactivity of 1g

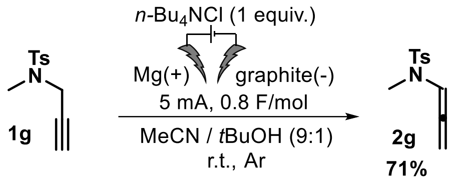

Run 1: Following the general procedure B in CC (constant current) mode (5 mA, 0.8 F/mol) on a 0.5 mmol scale of **1g**: 80 mg (0.36 mmol, 72%) of **2g** as a white solid was obtained.
Run 2: CC (5 mA 0.3 F/mol): 54% isolated yield of **2g**

4-Methyl-*N*-phenyl-*N*-(propa-1,2-dien-1-yl)benzenesulfonamide (**2g**)

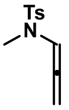

^{1}H NMR (500 MHz, $CDCl_3$) δ 7.67 (d, *J* = 8.3 Hz, 2H), 7.32 (d, *J* = 8.0 Hz, 2H), 6.90 (t, *J* = 6.2 Hz, 1H), 5.29 (d, *J* = 6.2 Hz, 2H), 2.71 (s, 3H), 2.43 (s, 3H) ppm.
^{13}C NMR (126 MHz, $CDCl_3$) δ 201.5, 143.8, 133.8, 129.7, 127.4, 101.7, 87.6, 33.2, 21.5 ppm.
The results of ^{1}H and ^{13}C NMR analysis are in agreement with the literature data.[25]

Reactivity of 1h

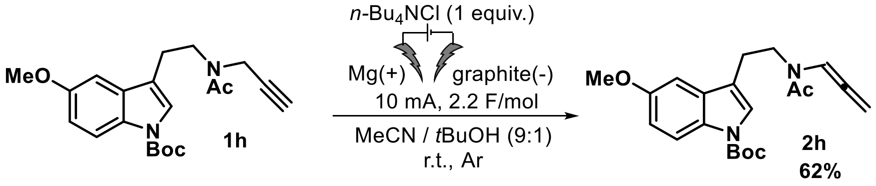

Run 1: Following the general procedure B in CC (constant current) mode (10 mA, 2.2 F/mol) on a 0.5 mmol scale of **1h**: 116 mg (0.31 mmol, 62%) of **2h** as a colorless oil was obtained.

Run 2: CC (5 mA 1.0 F/mol): 6% NMR yield of **2h**(25% conversion of **1h**)

Tert-butyl 5-methoxy-3-(2-(N-(propa-1,2-dien-1-yl)acetamido)ethyl)-1H-indole-1-carboxylate (**2h**)

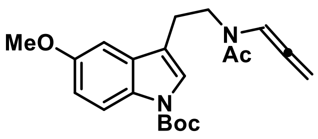

At 25 °C, the 1H NMR spectrum of **P8** in DMSO and CHCl3 indicates the presence of two rotamers Coalescence of the signals in the 1H NMR spectrum in DMSO is observed at approximately 100 °C.

^{1}H NMR (500 MHz, DMSO-d_6, 100 °C) δ7.92 (d, J = 9.0 Hz, 1H), 7.43 (s, 1H), 7.12 (s, 1H), 6.94 (dd, J = 8.9, 2.6 Hz, 1H), 5.46 (d, J = 6.4 Hz, 2H), 3.83 (s, 3H), 3.75 – 3.66 (m, 2H), 2.92 – 2.87 (m, 3H), 2.10 (s, 3H), 1.64 (s, 9H) ppm.
^{13}C NMR (126 MHz, DMSO-d_6, 100 °C) δ 167.5, 155.3, 148.5, 130.7, 129.2, 123.3, 117.0, 114.9, 112.5, 102.1, 85.9, 82.9, 55.2, 27.3, 22.4, 20.8 ppm.
HRMS (ESI-TOF) m/z: [M + Na]$^+$ calculated for $C_{21}H_{26}N_2O_4Na$ 393.1790; found 393.1787

Reactivity of 1i

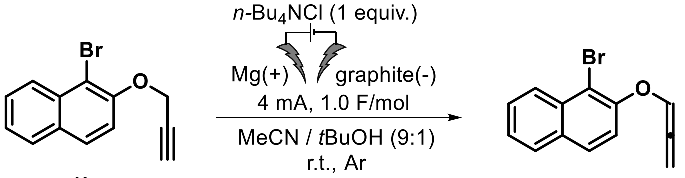

1i **2i** **80%**

Runs 1 & 2: Following the general procedure B in CC (constant current) mode (4 mA, 1.6 F/mol) on a 0.5 mmol scale of **1i**: 103 mg (0.390 mmol, 78%, run 1) or 108 mg (0.409 mmol, 82%, run 2) of **2i** as a yellow oil was obtained. Average yield based on 2 runs: 80%.

1-Bromo-2-(propa-1,2-dien-1-yloxy)naphthalene (**2i**)

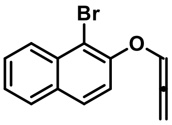

^{1}H NMR (500 MHz, $CDCl_3$) δ 8.27 (d, J = 8.5 Hz, 1H), 7.86 – 7.74 (m, 2H), 7.60 (ddd, J = 8.3, 6.8, 1.3 Hz, 1H), 7.47 (ddd, J = 8.0, 6.9, 1.1 Hz, 1H), 7.39 (d, J = 8.9 Hz, 1H), 6.99 (t, J = 5.9 Hz, 1H), 5.43 (d, J = 5.9 Hz, 2H) ppm.
.**^{13}C NMR** (126 MHz, $CDCl_3$) δ 202.0, 151.4, 133.0, 131.2, 128.6, 128.1, 127.7, 126.7, 125.4, 119.5, 118.7, 111.7, 91.0 ppm.
The results of ^{1}H and ^{13}C NMR analysis are in agreement with the literature data.[28]

Reactivity of 2j

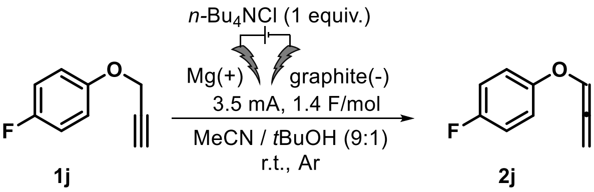

1j **2j** **66%**

Run 1: Following the general procedure B in CC (constant current) mode (4 mA, 1.4 F/mol) on a 0.5 mmol scale of **1j**: 66% NMR yield of **2j** (81% conversion of **1j**).
Run 2: CC (4 mA, 1.0 F/mol): 38% NMR yield of **2j** was obtained (79% conversion of **1j**).

[28]*Angew. Chem. Int. Ed.* **2024**, 63, e202411074

Run 3: CC (3.5 mA, 1.6 F/mol): 58% NMR yield of **2j** was obtained (83% conversion of **1j**).

1-Fluoro-4-(propa-1,2-dien-1-yloxy)benzene (**2j**)

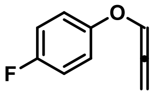

^{1}H NMR (500 MHz, $CDCl_3$) δ 7.06 – 6.94 (m, 4H), 6.81 (t, *J* = 5.9 Hz, 1H), 5.44 (d, *J* = 5.9 Hz, 2H) ppm.
^{13}C NMR (126 MHz, $CDCl_3$) δ 202.4, 158.5 (d, *J* = 240.8 Hz), 153.0 (d, *J* = 2.4 Hz), 118.7, 118.4 (d, *J* = 8.2 Hz), 115.9 (d, *J* = 23.4 Hz), 90.0 ppm.
The results of ^{1}H and ^{13}C NMR analysis are in agreement with the literature data.[29]

Reactivity of 1k

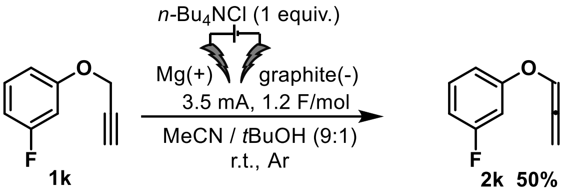

Run 1: Following the general procedure B in CC (constant current) mode (3.5 mA, 1.2 F/mol) on a 0.5 mmol scale of **1k**: 50% NMR yield of **2k** (63% conversion of **S13**).
Run 2: CC (5mA, 0.8 F/mol): 44% NMR yield of **2k** was obtained (80% conversion of **1k**).

1-fluoro-3-(propa-1,2-dien-1-yloxy)benzene (**2k**)

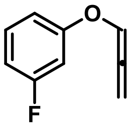

^{1}H NMR (500 MHz, $CDCl_3$) δ 7.33 – 7.17 (m, 1H), 6.93 – 6.70 (m, 4H), 5.47 (d, *J* = 5.9 Hz, 2H) ppm.
^{13}C NMR (126 MHz, $CDCl_3$) δ 202.7, 163.4 (d, *J* = 246.3 Hz), 158.4 (d, *J* = 10.6 Hz), 130.3 (d, *J* = 9.9 Hz), 117.3, 112.3 (d, *J* = 3.0 Hz), 109.5 (d, *J* = 21.4 Hz), 104.5 (d, *J* = 24.9 Hz), 89.7 ppm.
HRMS (APCI-TOF) m/z: $[M + H]^+$ calculated for C_9H_8FO 151.0559; found 151.0550.

Reactivity of 1l

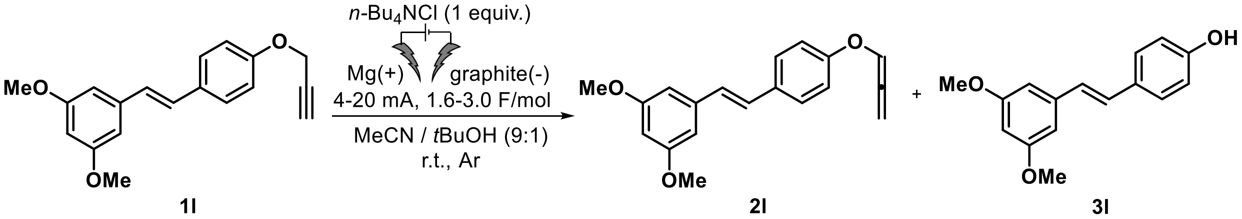

Run 1: Following the general procedure B in CC (constant current) mode (4 mA, 1.6 F/mol) on a 0.5 mmol scale of **1l**: 92 mg (0.31 mmol, 62%) of **2l** as a white, oily solid was obtained (70% conversion of **1l**).
Run 2: CC (5 mA, 1.6 F/mol): 24 mg (0.082 mmol, 16%) of **2l** as a white, oily solid and 30 mg (0.12 mmol, 24%) of pterostilbene **3l** as a white solid was obtained (73% conversion of **S10**).
Run 3: CC (20 mA, 3.0 F/mol): 62% isolated yield (66% NMR yield) of **3l** was obtained (86% conversion of **1l**).

[29] *RSC. Adv.* **2025**, 15, 11770–11773

E-1,3-dimethoxy-5-(4-(propa-1,2-dien-1-yloxy)styryl)benzene (**2l**)

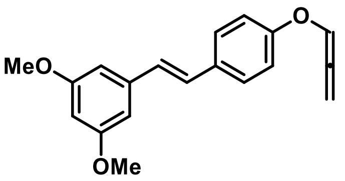

^{1}H NMR (500 MHz, $CDCl_3$) δ 7.46 (d, *J* = 8.7 Hz, 2H), 7.09 – 7.01 (m, 3H), 6.94 (d, *J* = 16.3 Hz, 1H), 6.86 (t, *J* = 5.9 Hz, 1H), 6.66 (d, *J* = 2.3 Hz, 2H), 6.39 (t, *J* = 2.2 Hz, 1H), 5.47 (d, *J* = 5.9 Hz, 2H), 3.83 (s, 6H) ppm.
^{13}C NMR (126 MHz, $CDCl_3$) δ 202.8, 161.96, 156.8, 139.4, 131.9, 128.4, 127.7, 127.6, 117.7, 116.9, 104.4, 99.8, 89.6, 55.3 ppm.
HRMS (ESI-TOF) m/z: $[M + H]^+$ calculated for $C_{19}H_{19}O_3$ 295.1334; found: 295.1333.

E-4-(3,5-dimethoxystyryl)phenol, pterostilbene (**3l**)

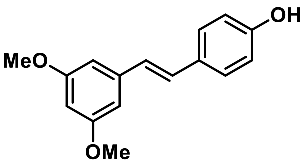

^{1}H NMR (600 MHz, $CDCl_3$) δ 7.40 (d, *J* = 8.7 Hz, 2H), 7.03 (d, *J* = 16.3 Hz, 1H), 6.90 (d, *J* = 16.3 Hz, 1H), 6.83 (d, *J* = 8.7 Hz, 2H), 6.67 (d, *J* = 2.3 Hz, 2H), 6.41 (t, *J* = 2.3 Hz, 1H), 3.84 (s, 6H) ppm.
^{13}C NMR (151 MHz, $CDCl_3$) δ 160.9, 155.4, 139.7, 130.0, 128.7, 128.0, 126.5, 115.6, 104.4, 99.6, 55.4 ppm.
The results of ^{1}H and ^{13}C NMR analysis are in agreement with the literature data.[30]

Reactivity of 1m

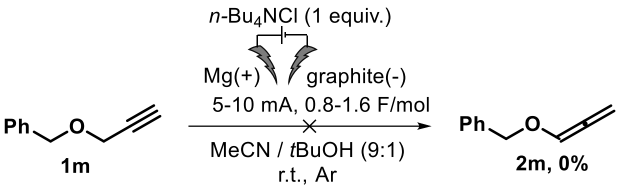

Run 1: Following the general procedure B in CC (constant current) mode (5 mA, 0.8 F/mol) on a 0.5 mmol scale of **1m**: 0% conversion of **1m.**
Run 2: CC (10 mA, 1.6 F/mol): 0% conversion of **1m** (NMR).

Reactivity of 1n

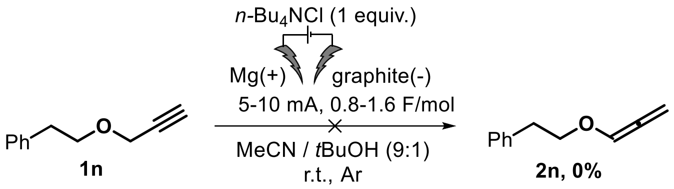

Run 1: Following the general procedure B in CC (constant current) mode (5 mA, 0.8 F/mol) on a 0.5 mmol scale of **1n**: 0% conversion of **1n.**
Run 2: CC (10 mA, 1.6 F/mol): 0% conversion of **1n** (NMR).

Reactivity of 1o

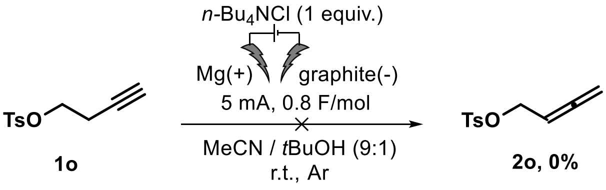

[30] *J. Org. Chem.* **2025**, 90, 9, 3480–3484

Following the general procedure Bin CC (constant current) mode (5 mA, 0.8 F/mol) on a 0.5 mmol scale of **1o**(commercial reagent, no additional purification): 0% conversion of **1o**

Reactivity of 1p

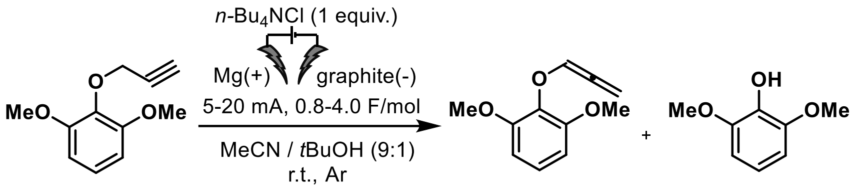

1p **2p** **3p**

Run 1: Following the general procedure B in CC (constant current) mode (5 mA, 0.8 F/mol) on a 0.5 mmol scale of **1p**: 0% conversion of **1p** (NMR)
Run 2: CC (10 mA, 1.6 F/mol): 0% conversion of **1p** (NMR)
Run 3: CC (20 mA, 2.0 F/mol, HPLC grade CH_3CN): 35% conversion of **1p**, 10% NMR yield of **P15a**, 25% NMR yield of **3p**
Run 4: CC (20 mA, 4.0 F/mol, HPLC grade CH_3CN): 60% conversion of **1p**, 10% NMR yield of **P15a**, 50% NMR yield **3p**
Run 5: CC (20 mA, 4.0 F/mol, HPLC grade CH_3CN, TEAC instead of ***n*-Bu$_4$NCl**): 65% conversion of **1p**, 3% NMR yield of **2p**, 52% NMR yield **3p**

Products were not isolated, identification by comparison of the crude mixture spectra with the literature spectra of **2p**[31] and **3p**.[32]

Reactivity of 1r

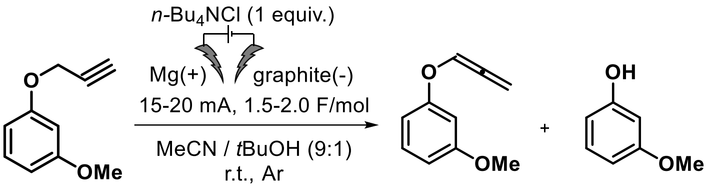

1r **2r** **3r**

Run 1: Following the general procedure B in CC (constant current) mode (15 mA, 0.3 F/mol) on a 0.5 mmol scale of **1r**: 63% conversion of **1r**, 9% NMR yield of **2r**, 32% NMR yield of **3r**
Run 2: CC (20 mA, 2.0 F/mol, HPLC grade CH_3CN): 65% conversion of **1r**, 3% NMR yield of **2r**, 52% NMR yield of **3r**
Products were not isolated, identification by comparison of the crude mixture spectra with the literature spectra of **2r**[33] and **3r**.[34]

[31] *Org. Lett.* **2025**, 27, 45, 12448–12452
[32] *Org. Lett.* **2012**, 14, 13, 3494–3497
[33] *Angew. Chem. Int. Ed.* **2022**, *61*, e202201753
[34] *Org. Lett.* **2017**, 19, 10, 2670–2673

Reactivity of 1s

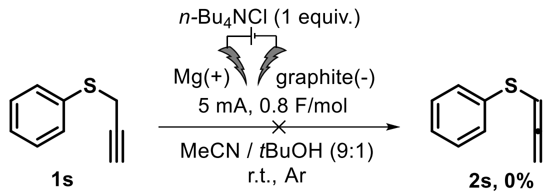

Following the general procedure B in CC (constant current) mode (5 mA, 0.8 F/mol) on a 0.5 mmol scale of **1s**(commercial reagent, no additional purification): 0% conversion of **1s**.

Reactivity of 1t

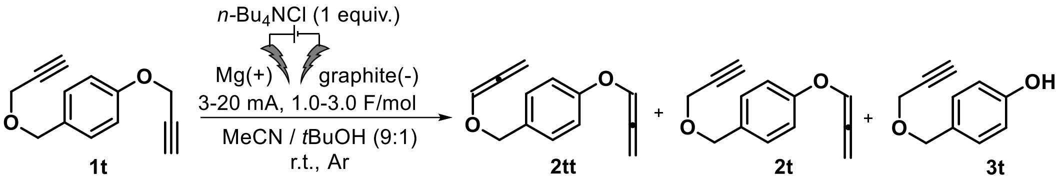

Table 6. Electrochemical reactivity of **1t**: constant current experiments

Entry	Current [mA]	Charge [F/mol]	1t conversion[a] [%]	2tt yield[a,b] [%]	2t yield[a,b] [%]	3t yield[a,b] [%]
**1**	5	1.0	50	n.d.	6	30
**2**	4	1.0	50	n.d.	23	17
**3**	5	2.0	53	n.d.	9	27
**4**	3	1.5	39	n.d.	33	0
**5**	3	2.0	48	n.d.	9	21
**6**	3	2.5	53	n.d.	5	31
**7**	10	2.0	71	n.d.	17	33
**8**	12	2.0	84	n.d.	10	62
**9**	12.5	2.5	88	n.d.	4	65
**10[c]**	12.5	2.5	92	n.d.	2	68
**11**	20	3.0	>95	n.d.	n.d.	80
**12[c]**	20	3.0	>95	n.d.	n.d.	78
**13[c]**	20	4.0	>95	n.d.	n.d.	65

Standard reaction conditions: **1t** (0.25 mmol), ***n*-Bu$_4$NCl** (dried at high temperature under high vacuum overnight, 1.0 equiv.), solvent (0.05M): anhydrous CH_3CN (4.5 mL) + anhydrous *t*BuOH (0.5 mL), Mg(+)|(-)graphite, constant current (x mA, y F/mol), Mg electrodes were additionaly activated by soaking them in diluted HCl_{aq} before reaction undivided cell, ElectraSyn 2.0 IKA potentiostat, argon [a]Based on ^{1}H NMR analysis using 1,3,5-trimethoxybenzene as an internal standard [b]isolated yield in parentheses [c]HPLC grade CH_3CN was used as a solvent

Table 7. Electrochemical reactivity of **1l:** constant potential experiments

Entry	Potential [V]	Charge [F/mol]	1tconversion[a] [%]	2tt yield[a,b] [%]	2t yield[a,b] [%]	3t yield[a,b] [%]
**1**	1.2	1.0	36	n.d.	21	n.d.
**2**	1.2	1.5	65	n.d.	43	n.d.
**3**	1.2	2.0	100	n.d.	76 (46)	n.d.

Standard reaction conditions: **1t** (0.25 mmol), ***n*-Bu$_4$NCl** (dried at high temperature under high vacuum overnight, 1.0 equiv.), solvent (0.05M): anhydrous CH_3CN (4.5 mL) + anhydrous *t*BuOH (0.5 mL),

Mg(+)|(-)graphite, constant potential (x V, y F/mol), no reference electrode, Mg electrodes were additionaly activated by soaking them in diluted HCl_{aq} before reaction, undivided cell, ElectraSyn 2.0 IKA potentiostat, argon [a]Based on ^{1}H NMR analysis using 1,3,5-trimethoxybenzene as an internal standard [b]isolated yield in parentheses

Classical synthesis (*t*-BuOK as a base catalyst)

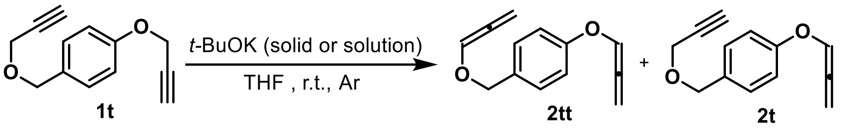

As a general rule, reactions were performed in a 0.25-0.50 mmol scale of **1t**, that was placed in 50 mL round-bottom flask along with anhydrous THF (0.05M concentration) under argon. Potassium *t*-butoxide was added in an appropriate manner, stirring continued for a given period of time, then reaction was quenched with small amount of $NH_4Cl_{aq.}$ Mixture was transferred to a separatory funnel, diluted with diethyl ether (approximately 50 mL), 10% NH_4Cl_{aq}. (50 mL) and in some cases the internal standard (1,3,5-trimethoxybenzene, 0.333 equiv.) were added. The phases were separated, the organic layer was washed with additional water (50 mL) and brine (50 mL), then dried over anhydrous Na_2SO_4. The drying agent was filtered off, the solvents were removed by evaporation and the crude product was analyzed by ^{1}H NMR to investigate the conversion of substrate as well as yield of the product and/or purified by a (flash) column chromatography on SiO_2 to isolate the pure allene.

Table 8. Reactivity of **1l** in classical allene synthesis

10	Base form and amount	Base addition	Time [h]	1t conversion[a] [%]	2tt yield[a,b] [%]	2t yield[a,b] [%]
**1**[c]	solid *t*BuOK (30%)	one portion	2	100	75	0
**2**	solid *t*BuOK (30%)	one portion	1	85	80(55)	0
**3**	1M *t*BuOK/THF (50%)	5 portionsevery 10 min (manually)	1	82	27	55
**4**	1M *t*BuOK/THF (30%)	dropwise (syringe pump) for 30 min	0.75	0	0	0
**5**	1M *t*BuOK/THF (50%)	one portion (20%) then dropwise (30%) using syringe pump for 30 min	0.75	80	~70	~10

Standard reaction conditions: **1t** (0.50 mmol), solvent (0.05M): anhydrous THF [a]Based on ^{1}H NMR analysis using 1,3,5-trimethoxybenzene as an internal standard [b]solvent (0.05M): anhydrous CH_3CN, [b]isolated yield in parentheses [c]0.25 mmol scale of **1t**

After ^{1}H NMR analysis the crude reaction mixtures were combined and the products were separated usings flash chromatography to give pure allenes.

1-(Propa-1,2-dien-1-yloxy)-4-((propa-1,2-dien-1-yloxy)methyl)benzene (**2tt**)

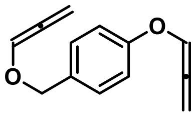

Colorless oil

^{1}H NMR (500 MHz, $CDCl_3$) δ 7.31 (d, *J* = 8.4 Hz, 2H), 7.08 – 7.02 (m, 2H), 6.84 (t, *J* = 5.9 Hz, 1H), 6.82 (t, *J*= 5.9 Hz, 1H), 5.48 (d, *J* = 6.0 Hz, 2H), 5.45 (d, *J* = 6.0 Hz, 2H), 4.57 (s, 2H) ppm.

^{13}C NMR (126 MHz, $CDCl_3$) δ 202.7, 201.3, 156.9, 131.6, 129.3, 121.5, 117.8, 116.7, 91.0, 89.6, 70.2 ppm.

HRMS (APCI-TOF) m/z: $[M + H]^+$ calculated for $C_{13}H_{13}O_2$ 201.0916; found 201.0913.

1-((Prop-2-yn-1-yloxy)methyl)-4-(propa-1,2-dien-1-yloxy)benzene (**2t**)

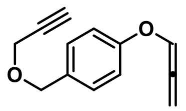

Colorless oil

^{1}H NMR (600 MHz, $CDCl_3$) δ 7.31 (d, *J* = 8.9 Hz, 2H), 7.05 (d, *J* = 8.9 Hz, 2H), 6.84 (t, *J* = 5.9 Hz, 1H),6.09 (s, TMB), 5.45 (d, *J* = 6.0 Hz, 2H), 4.56 (s, 2H), 4.16 (d, *J* = 2.4 Hz, 2H),3.77 (s, TMB), 2.47 (t, *J* = 2.4 Hz, 1H) ppm.

^{13}C NMR (151 MHz, $CDCl_3$) δ 202.7, 161.5, 156.9, 131.6, 129.6, 117.8, 116.8, 92.9, 89.7, 79.6, 74.6, 71.0, 56.9, 55.3 ppm (red –1,3,5-trimethoxybenzene as internal standard).

HRMS (APCI-TOF, ESI-TOF): decomposition

4-((Prop-2-yn-1-yloxy)methyl)phenol (**3t**)

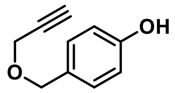

Colorless oil

^{1}H NMR (600 MHz, $CDCl_3$) δ 7.21 (d, *J* = 8.6 Hz, 2H), 6.79 (d, *J* = 8.7 Hz, 2H), 5.98 (s, 1H), 4.54 (s, 2H), 4.15 (d, *J* = 2.4 Hz, 2H), 2.48 (t, *J* = 2.4 Hz, 1H) ppm.

^{13}C NMR (151 MHz, $CDCl_3$) δ 155.7, 130.1, 128.8, 115.4, 79.5, 74.8, 71.2, 56.6 ppm.

The results of ^{1}H and ^{13}C NMR analysis are in agreement with the literature data.[35]

Reactivity of 1u

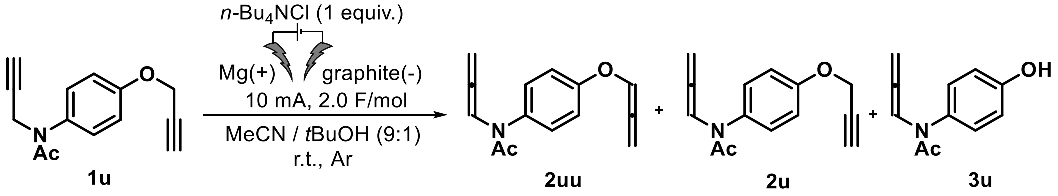

Run 1: Following the general procedure B in CC (constant current) mode (10 mA, 2.0 F/mol) on a 0.5 mmol scale of **1u**: 20 mg (0.088 mmol, 18%) of **2uu** as a yellow oil, 6 mg (0.026 mmol, 6%) of **2u** as a yellow oil and 31 mg (0.164 mmol, 32%) of **3u** as a yellow oil (that later solidified) were obtained (entry 1, Table 6).
Run 2: CC (4 mA 1.2 F/mol): 57% isolated yield of **2uu** and 19% isolated yield of **2u**(full conversion of **1u**)(entry 2, Table 6).

Table 9. Electrochemical reactivity of **1u** using low current

Entry	Current [mA]	Charge[F/mol]	1u conversion[a] [%]	2uu yield[a,b] [%]	2u yield[a,b] [%]	3u yield[a,b] [%]
**1***	10	2.0	100	18	6	**32**
**2***	4	1.2	100	**57**	19	n.d.
**1**	3	1.0	47	7	21	n.d.
**2**	3	1.2	54	10	26	n.d.
**3**	2.5	1.5	80	28	**52**	n.d.
**4**	2.5	2.0	100	14	44	n.d.
**5[c]**	2.0	1.5	87	34	31	n.d.

Standard reaction conditions: **S9** (0.25 mmol), ***n*-Bu4NCl** (dried at high temperature under high vacuum overnight, 1.0 equiv.), solvent (0.05M): anhydrous CH_3CN (4.5 mL) + anhydrous *t*BuOH (0.5 mL),

[35]*Green Chem.*, **2023**, 25, 2401-2408

Mg(+)|(-)graphite, constant current (x mA, y F/mol), Mg electrodes were additionaly activated by soaking them in diluted HCl_{aq} before reaction undivided cell, ElectraSyn 2.0 IKA potentiostat, argon [a]Based on ^{1}H NMR analysis using 1,3,5-trimethoxybenzene as an internal standard [b]isolated yield in parentheses [c]average of two runs.*0.5 mmol scale of **1u**.

Classical synthesis (*t*-BuOK as a base catalyst)

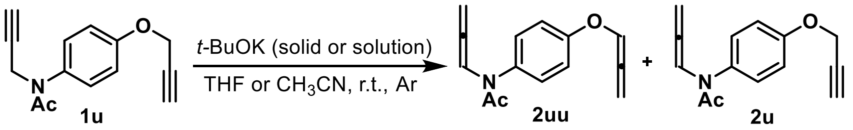

As a general rule, reactions were performed in a 0.50 mmol scale of **S9**, that was placed in 50 mL round-bottom flask along with anhydrous solvent (0.05M concentration) under argon. Potassium t-butoxide was added in an appropriate manner, stirring continued for a given period of time, then reaction was quenched with small amount of $NH_4Cl_{aq.}$ Mixture was transferred to a separatory funnel, diluted with diethyl ether (approximately 50 mL), 10% NH_4Cl_{aq}. (50 mL) and in some cases the internal standard (1,3,5-trimethoxybenzene, 0.333 equiv.) were added. The phases were separated, the organic layer was washed with additional water (50 mL) and brine (50 mL), then dried over anhydrous Na_2SO_4. The drying agent was filtered off, the solvents were removed by evaporation and the crude product was analyzed by ^{1}H NMR to investigate the conversion of substrate as well as yield of the product and/or purified by a (flash) column chromatography on SiO_2 to isolate the pure allene.

Table 10. Reactivity of **1u** in classical allene synthesis

Entry	Base form and ammount	Base addition	Total reactiontime [h]	1u conversion[a] [%]	2uu yield[a] [%]	2u yield[a] [%]
**1**	solid*t*BuOK (30%)	one portion	2	100	29	<5
**2**	1M *t*BuOK/THF (30%)	dropwise (manually) for 10 min	2	100	32	12
**3**	1M *t*BuOK/THF (30%)	dropwise (manually) for 10 min	1	100	52	13
**4**	1M *t*BuOK/THF (30%)	dropwise (manually) for 30 min	0.5	35	5	30
**5**	1M *t*BuOK/THF (30%)	5 portionsevery 10 min (manually)	1	100	27	24
**6**	1M *t*BuOK/THF (30%)	dropwise (syringe pump) for 30 min	0.75	0	0	0
**7[b]**	1M *t*BuOK/THF (30%)	dropwise (syringe pump) for 30 min	0.75	100	60	20

Standard reaction conditions: **S9** (0.50 mmol), solvent (0.05M): anhydrous THF [a]Based on ^{1}H NMR analysis using 1,3,5-trimethoxybenzene as an internal standard [b]solvent (0.05M): anhydrous CH_3CN

N-(propa-1,2-dien-1-yl)-*N*-(4-(propa-1,2-dien-1-yloxy)phenyl)acetamide (**2uu**)

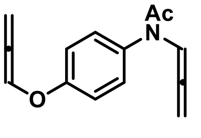

^{1}H NMR (500 MHz, $CDCl_3$) δ 7.66 (t, *J* = 6.4 Hz, 1H), 7.14 – 7.05 (m, 4H), 6.84 (t, *J* = 5.9 Hz, 1H), 5.47 (d, *J* = 5.9 Hz, 2H), 5.00 (d, *J* = 6.4 Hz, 2H), 1.88 (s, 3H) ppm.

^{13}C NMR (126 MHz, $CDCl_3$) δ 202.8, 202.6, 168.7, 156.9, 134.7, 129.6,117.3, 117.2, 101.1, 89.8, 86.4, 22.9 ppm.

HRMS (ESI-TOF) m/z: $[M + Na]^+$ calculated for $C_{14}H_{13}NO_2Na$ 250.0844; found 250.0847.

N-(4-(Prop-2-yn-1-yloxy)phenyl)-*N*-(propa-1,2-dien-1-yl)acetamide (**2u**)

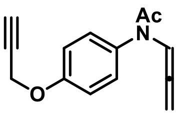

^{1}H NMR (500 MHz, $CDCl_3$) δ 7.66 (t, *J* = 6.4 Hz, 1H), 7.10 (d, *J* = 8.7 Hz, 2H), 7.00 (d, *J* = 8.8 Hz, 2H), 5.00 (d, *J* = 6.4 Hz, 2H), 4.71 (d, *J* = 2.4 Hz, 2H), 2.55 (t, *J* = 2.5 Hz, 1H), 1.89 (s, 3H) ppm.
^{13}C NMR (126 MHz, $CDCl_3$) δ 202.6, 168.8, 157.3, 133.7, 129.5, 115.5, 101.1, 86.4, 78.1, 75.9, 56.0, 22.3 ppm.
HRMS (ESI-TOF) m/z: $[M + Na]^+$ calculated for $C_{14}H_{13}NO_2Na$ 250.0844; found 250.0848.

N-(4-Hydroxyphenyl)-*N*-(propa-1,2-dien-1-yl)acetamide (**3u**)

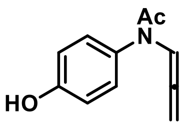

^{1}H NMR (500 MHz, $CDCl_3$) δ 7.66 (t, *J* = 6.4 Hz, 1H), 7.03 (d, *J* = 8.7 Hz, 2H), 6.89 (d, *J* = 8.6 Hz, 2H), 6.07 (s, 1H), 5.01 (d, *J* = 6.4 Hz, 2H), 1.91 (s, 3H) ppm.
^{13}C NMR (126 MHz, $CDCl_3$) δ 202.7, 169.3, 156.0, 132.6, 129.5, 116.2, 101.1, 86.4, 22.8 ppm.
HRMS (ESI-TOF) m/z: $[M - H]^-$ calculated for: $C_{11}H_{10}NO_2$ 188.0712; found 188.0715.

Reactivity of 1w

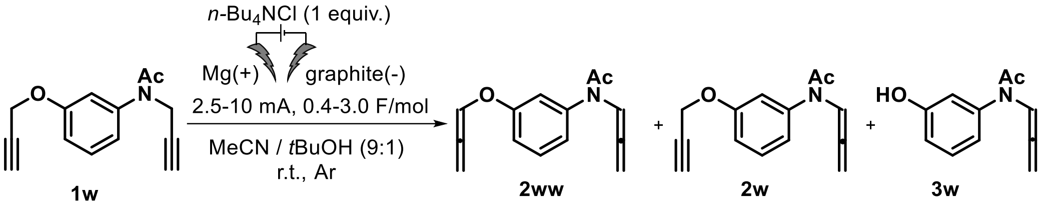

Table 11. Electrochemical reactivity of **1w**

Entry	Current [mA]	Charge[F/mol]	1wconversion[a] [%]	2ww yield[a,b] [%]	2w yield[a,b] [%]	3w yield[a,b] [%]
**1**	4	1.5	100	39	17	15
**2**	4	0.6	70	25	30	n.d.
**3**	4	0.4	68	19	**47**	n.d.
**4**	3	0.8	100	47	37	n.d.
**5**	3	0.4	20	2	18	n.d.
**6**	2.5	1.5	100	50	12	n.d.
**7**	10	2.0	100	30	n.d.	34
**8**	10	3.0	100	n.d.	n.d.	47
**9**	inconstant[c]	3.0	100	n.d.	n.d.	(50)

Standard reaction conditions: **S22** (0.25 mmol), ***n*-Bu₄NCl** (dried at high temperature under high vacuum overnight, 1.0 equiv.), solvent (0.05M): anhydrous CH_3CN (4.5 mL) + anhydrous *t*BuOH (0.5 mL), Mg(+)|(-)graphite, constant current (x mA, y F/mol), Mg electrodes were additionaly activated by soaking them in diluted HCl_{aq} before reaction undivided cell, ElectraSyn 2.0 IKA potentiostat, argon [a]Based on ^{1}H NMR analysis using 1,3,5-trimethoxybenzene as an internal standard [b]isolated yield in parentheses [c] constant potential (2.0V), no reference electrode

After ^{1}H NMR analysis the crude reaction mixtures were combined and the products were separated usings flash chromatography to give pure allenes.

N-(Propa-1,2-dien-1-yl)-N-(3-(propa-1,2-dien-1-yloxy)phenyl)acetamide (**2ww**)

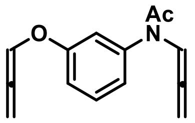

Orange oil

^{1}H NMR (500 MHz, $CDCl_3$) δ 7.63 (s, 1H), 7.35 (t, *J* = 8.0 Hz, 1H), 7.07 (dd, *J* = 8.3, 2.8 Hz, 1H), 6.92 – 6.84 (m, 2H), 6.81 (t, *J* = 5.9 Hz, 1H), 5.45 (d, *J* = 5.9 Hz, 2H), 5.01 (d, *J* = 6.4 Hz, 2H), 1.91 (s, 3H) ppm.

^{13}C NMR (126 MHz, $CDCl_3$) δ 202.5, 202.4, 168.2, 157.7, 141.1, 130.1, 122.7, 117.5, 117.1, 116.9, 100.7, 90.0, 86.5, 29.6, 22.8ppm.

HRMS (APCI-TOF) m/z: $[M + H]^+$ calculated for $C_{14}H_{14}NO_2$ 228.1025; found 228.1021.

N-(3-(Prop-2-yn-1-yloxy)phenyl)-*N*-(propa-1,2-dien-1-yl)acetamide (**2w**)

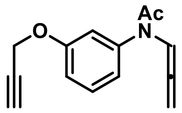

Orange oil

^{1}H NMR (500 MHz, $CDCl_3$) δ 7.62 (s, 1H), 7.32 (t, *J* = 8.1 Hz, 1H), 7.02 – 6.92 (m, 1H), 6.85 – 6.74 (m, 2H), 4.99 (d, *J* = 6.5 Hz, 2H), 4.79 – 4.61 (m, 2H), 2.52 (t, *J* = 2.4 Hz, 1H)1.90 (s, 3H)ppm.

^{13}C NMR (126 MHz, $CDCl_3$) δ 202.4, 168.3, 158.1, 141.0, 130.0, 121.4, 115.1, 115.1, 100.6, 86.4, 78.0, 75.9, 55.9, 22.8ppm.

HRMS (APCI-TOF) m/z: $[M + H]^+$ calculated for $C_{14}H_{14}NO_2$ 228.1025; found: 228.1022.

N-(3-Hydroxyphenyl)-*N*-(propa-1,2-dien-1-yl)acetamide (**3w**)

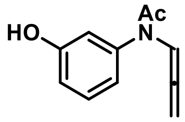

Orange oily solid

^{1}H NMR (500 MHz, $CDCl_3$) δ 7.78 (bs, 1H), 7.60 (t, *J* = 6.8 Hz, 1H), 7.26 (t, *J* = 8.3 Hz, 1H), 7.00 – 6.88 (m, 1H), 6.76 – 6.64 (m, 2H), 5.03 (d, *J* = 6.5 Hz, 2H), 1.96 (s, 3H) ppm.

^{13}C NMR (126 MHz, $CDCl_3$) δ 202.7, 169.6, 157.5, 140.7, 130.2, 119.7, 116.1, 115.4, 100.5, 86.5, 22.8, 15.0 ppm

HRMS (ESI-TOF) m/z: $[M - H]^-$ calculated for $C_{11}H_{10}NO_2$ 188.0712; found 188.0715.

Reactivity of 1x

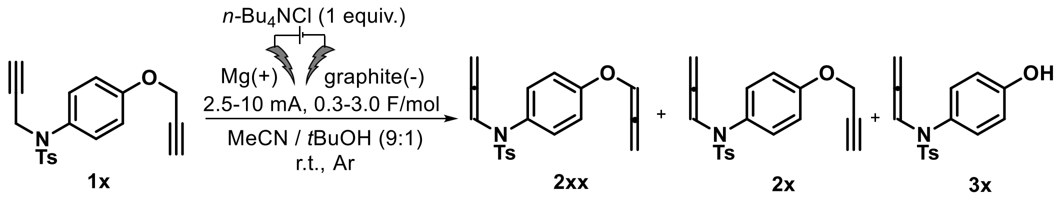

Table 12. Electrochemical reactivity of **1x**

Entry	Current [mA]	Charge[F/mol]	1xconversion[a] [%]	2xxyield[a,b] [%]	2xyield[a,b] [%]	3xyield[a,b] [%]
**1**	5	0.3	45	2	26	n.d.
**2**	5	1.0	84	10	45	n.d.
**3**	4	0.8	84	18	**58**	n.d.
**4**	3	1.0	100	20	50	n.d.
**5**	2.5	1.0	82	**32**	3	n.d.
**6**	10	3.0	100	<5	<5	n.d.
**7**	inconstant[c]	0.4	75	9	62 (48)	n.d.

Standard reaction conditions: **S22** (0.25 mmol), ***n*-Bu$_4$NCl** (dried at high temperature under high vacuum overnight, 1.0 equiv.), solvent (0.05M): anhydrous CH_3CN (4.5 mL) + anhydrous *t*BuOH (0.5 mL), Mg(+)|(-)graphite, constant current (x mA, y F/mol), Mg electrodes were additionaly activated by soaking them in diluted HCl_{aq} before reaction undivided cell, ElectraSyn 2.0 IKA potentiostat, argon [a]Based on ^{1}H NMR analysis

using 1,3,5-trimethoxybenzene as an internal standard [b]isolated yield in parentheses [c] constant potential (1.0V), no reference electrode, 0.5 mmol scale

After ^{1}H NMR analysis the crude reaction mixtures were combined and the products were separated usings flash chromatography to give pure allenes.

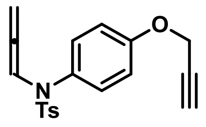

4-methyl-*N*-(4-(prop-2-yn-1-yloxy)phenyl)-*N*-(propa-1,2-dien-1-yl)benzenesulfonamide (**2x**)

Orange oil

^{1}H NMR (500 MHz, $CDCl_3$)δ7.54 (d, *J* = 8.2 Hz, 2H), 7.29 – 7.22 (m, 2H), 7.10 (t, *J* = 6.3 Hz, 1H), 6.91 (d, *J* = 9.0 Hz, 2H), 6.85 (d, *J* = 9.0 Hz, 2H), 5.02 (d, *J* = 6.3 Hz, 2H), 4.65 (d, *J* = 2.4 Hz, 2H), 2.53 (t, *J* = 2.4 Hz, 1H), 2.43 (s, 3H) ppm.
^{13}C NMR (126 MHz, $CDCl_3$) δ 200.9, 157.5, 143.8, 135.2, 130.7, 130.4, 129.5, 127.7, 114.8, 102.6, 87.5, 78.1, 75.8, 55.9, 21.6 ppm.
HRMS (ESI-TOF) m/z: $[M + Na]^+$ calculated for $C_{19}H_{17}NO_3SNa$ 362.0827; found 362.0838.

4-Methyl-N-(propa-1,2-dien-1-yl)-N-(4-(propa-1,2-dien-1-yloxy)phenyl)benzenesulfonamide (**2xx**)

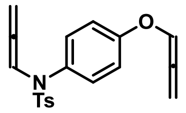

Yellow oil

^{1}H NMR (500 MHz, $CDCl_3$) δ 7.55 (d, *J* = 8.1 Hz, 2H), 7.27 (d, *J* = 8.0 Hz, 2H), 7.11 (t, *J* = 6.2 Hz, 1H), 6.99 – 6.90 (m, 4H), 6.80 (t, *J* = 5.9 Hz, 1H), 5.45 (d, *J* = 5.9 Hz, 2H), 5.03 (d, *J* = 6.2 Hz, 2H), 2.44 (s, 3H) ppm.
^{13}C NMR (126 MHz, $CDCl_3$) δ 202.8, 201.0, 157.1, 143.9, 135.2, 131.5, 130.8, 129.5, 127.7, 117.1, 116.5, 102.6, 89.6, 87.5, 21.6 ppm.
HRMS (ESI-TOF) m/z: $[M + Na]^+$ calculated for $C_{19}H_{17}NO_3SNa$ 362.0827; found: 362.0830.

Reactivity of S8

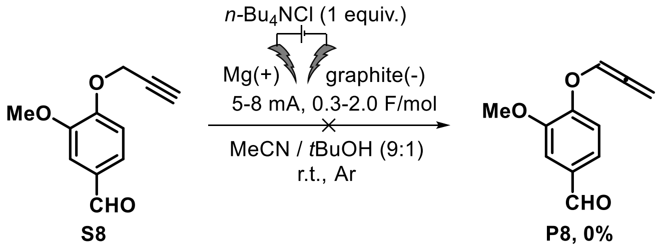

Run 1: Following the general procedure B in CC (constant current) mode (5 mA, 0.3 F/mol) on a 0.5 mmol scale of **S8**: slight conversion of **S8** (NMR), no allene observed
Run 2: CC (8 mA, 2.0 F/mol): >80% conversion of **S8** (NMR), no allene observed, decomposition

Reactivity of S9

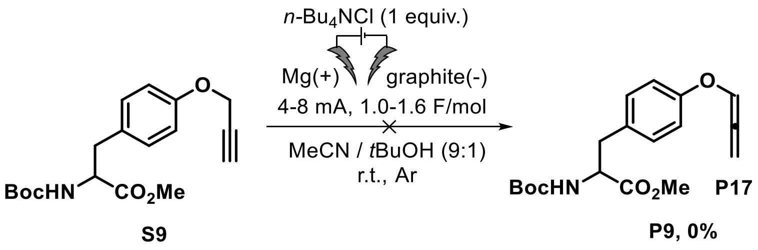

Run 1: Following the general procedure B in CC (constant current) mode (4 mA, 1.0 F/mol) on a 0.5 mmol scale of **S9**: **25%** conversion of **S9** (NMR), no allene observed
Run 2: CC (8 mA, 1.6 F/mol): **47%** conversion of **S9** (NMR), no allene observed

Reactivity of S10

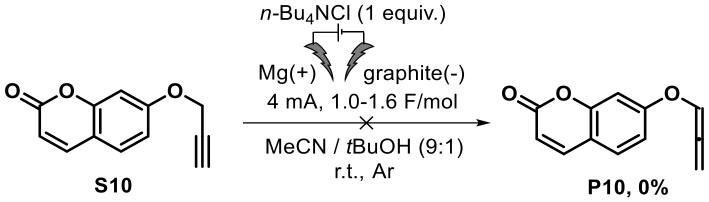

S10 **P10, 0%**

Run 1: Following the general procedure B in CC (constant current) mode (4 mA, 1.6 F/mol) on a 0.5 mmol scale of **S10**: **100%** conversion of **S10** (NMR), no allene observed, decomposition
Run 2: CC (4 mA, 1.0 F/mol): **100%** conversion of **S10** (NMR), no allene observed, decomposition

Reactivity of S12

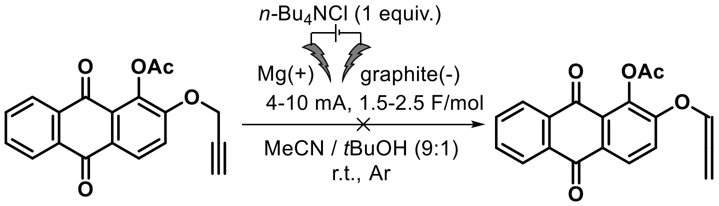

S12 **P12, 0%**

Run 1: Following the general procedure B in CC (constant current) mode (4 mA, 1.5 F/mol) on a 0.5 mmol scale of **S12**: no allene observed
Run 2: CC (10 mA, 2.5 F/mol): no allene observed, decomposition

Reactivity of S13

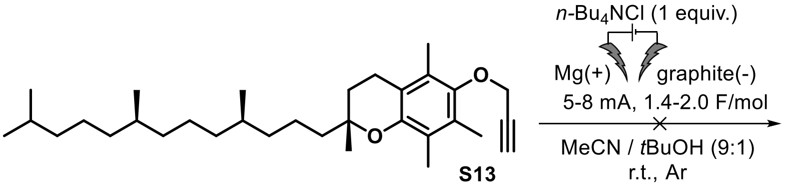

S13 **P13, 0%**

Run 1: Following the general procedure B in CC (constant current) mode (4 mA, 1.5 F/mol) on a 0.5 mmol scale of **S13**: ~20% conversion of **S13**, traces (<2%) allene observed
Run 2: CC (8 mA, 2.0 F/mol): ~25% conversion of **S13**, no allene observed

Reactivity of S14

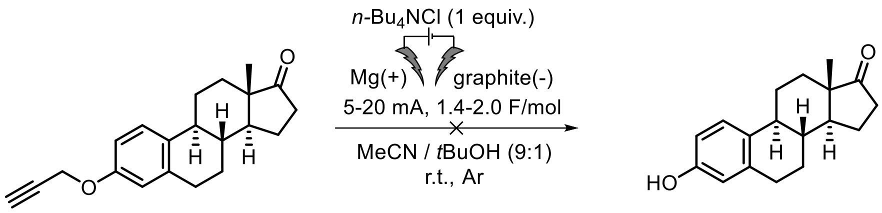

S14, 0% **P14**, 26%

Run 1: Following the general procedure B in CC (constant current) mode (5 mA, 0.8 F/mol) on a 0.5 mmol scale of **S14**: 0% conversion of **S14**

Run 2: CC (20 mA, 2.0 F/mol): ~43% conversion of **S14**, no allene observed, phenol 26% NMR yield. Productwas not isolated, identification by comparison of the crude mixture spectra with the literature spectra for **P14**.[36]

Reactivity of 2l

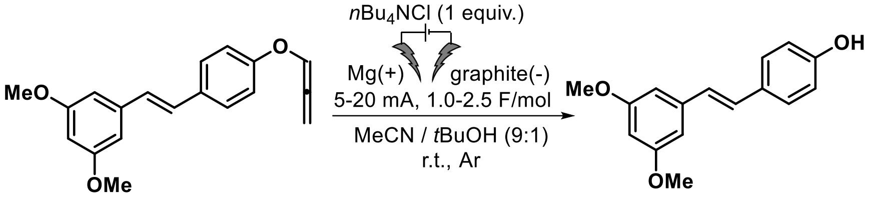

2l **3l**

Run 1: Following the general procedure B in CC (constant current) mode (5 mA, 1.0 F/mol) on a 0.5 mmol scale of **2l**: 30% conversion of **2l** (NMR), 15% yield of **3l** (NMR)

Run 2: CC (20 mA, 2.5 F/mol) on a 0.5 mmol scale of **2l**: 100% conversion of **2l** (NMR), 83% yield of **3l** (NMR)

[36] *Eur. J. Org. Chem.* **2022**, art. no. E202201112

4. Cyclic Voltammetry measurements

General Information:

Voltammetric measurements were performed using a BioLogic SP-200 computer-controlled potentiostat in a three-electrode configuration under an inert gas atmosphere at room temperature. A glassy carbon disk (d = 3 mm) was used as the working electrode, and a platinum wire served as the counter electrode. The reference electrode was Ag/AgCl. Prior to each measurement, the analyte solution was purged with argon. For each figure, the first scan recorded after holding the initial potential at 0.0V vs Ag/AgCl for 10 seconds is presented keeping IUPAC convention.

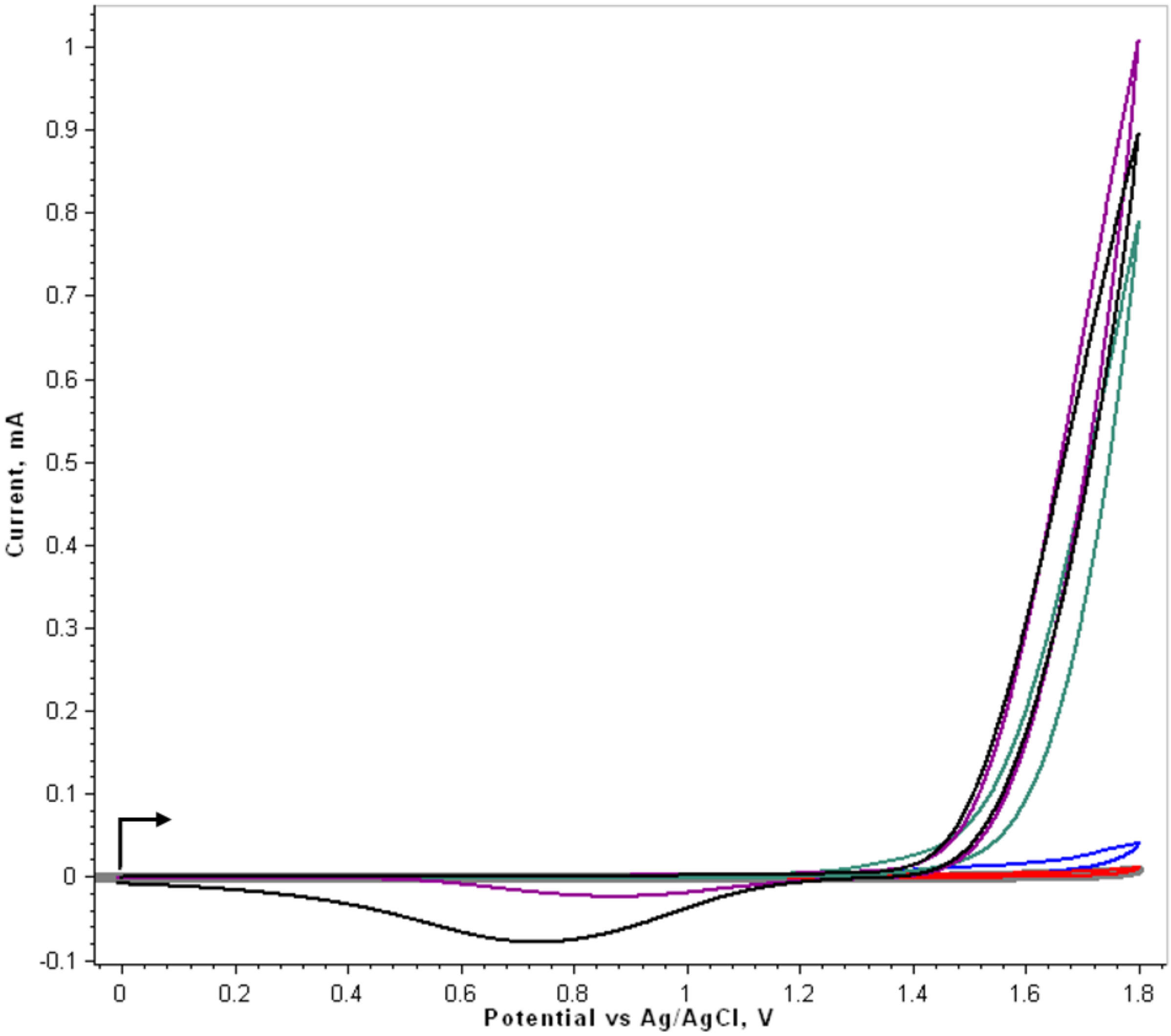

Figure 1. Cyclic voltammograms of **1a** (red), **2a** (blue), ***n*-Bu$_4$NCl** (black), **1a + *n*-Bu$_4$NCl** (green), and **2a + *n*-Bu$_4$NCl** (purple) recorded in MeCN solution of 0.1 M *n*-Bu$_4$NPF$_6$ (grey). The concentration of each analyte was 50 mM. The cyclic voltammetry in the oxidation region was recorded with 100 mV/s scan rate under Ar using glassy carbon disc electrode (*d* = 3 mm) as working electrode, platinum wire as counter electrode and Ag/AgCl as referenceelectrode.

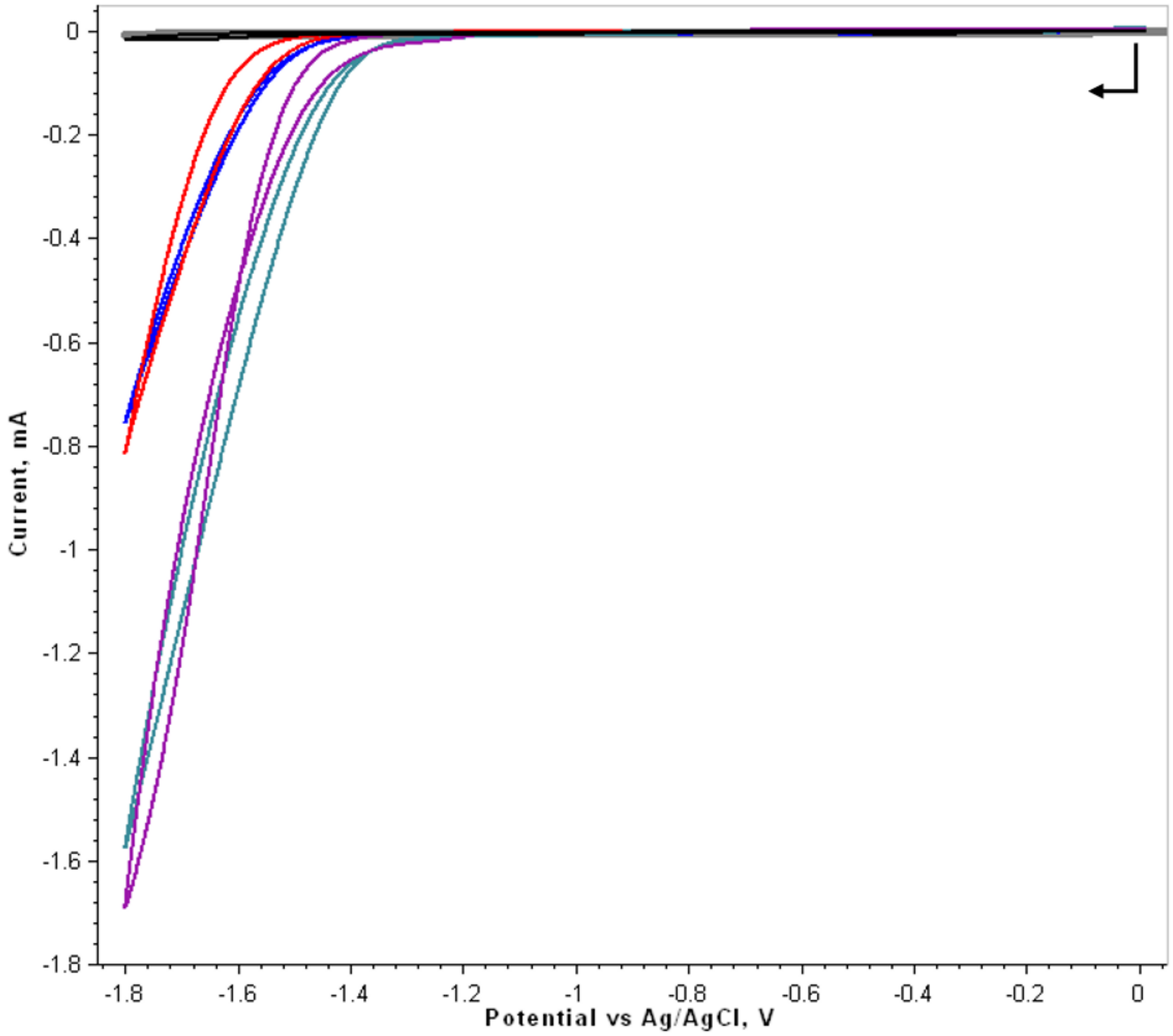

Figure 2. Cyclic voltammograms of **1a** (red), **2a** (blue), ***n*-Bu$_4$NCl** (black), **1a + *n*-Bu$_4$NCl** (green), and **2a + *n*-Bu$_4$NCl** (purple) recorded in MeCN solution of 0.1 M *n*-Bu$_4$NPF$_6$ (grey). The concentration of each analyte was 50 mM. The cyclic voltammetry in the oxidation region was recorded with 100 mV/s scan rate under Ar using glassy carbon disc electrode (*d* = 3 mm) as working electrode, platinum wire as counter electrode and Ag/AgCl as reference electrode.

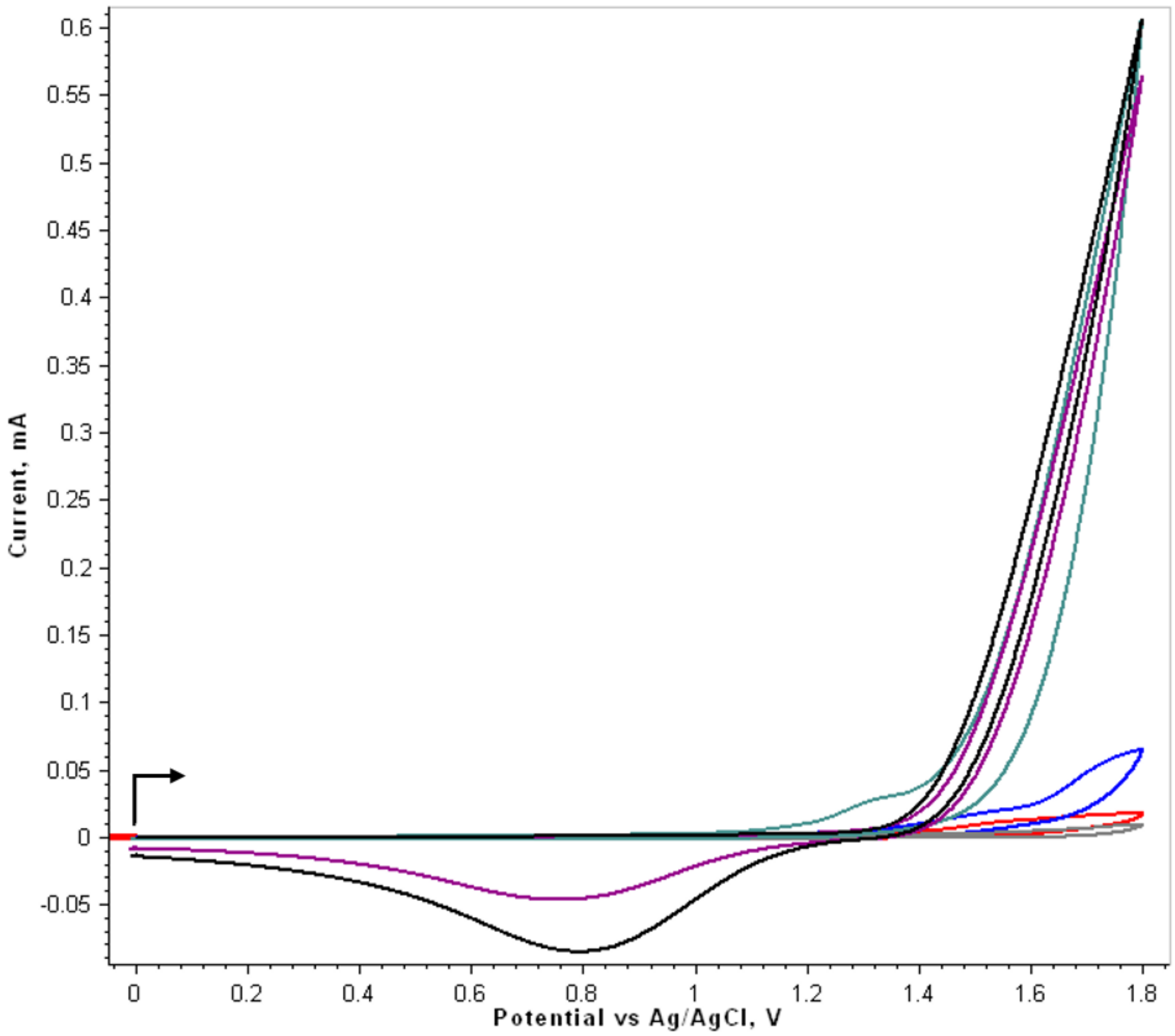

Figure 3. Cyclic voltammograms of **1a** (red), **2a** (blue), ***n*-Bu$_4$NCl** (black), **1 + *n*-Bu$_4$NCl** (green), and **2 + *n*-Bu$_4$NCl** (purple) recorded in MeCN – *t*-BuOH (9:1) solution of 0.1 M N-***n*-Bu$_4$NPF$_6$** (grey). The concentration of each analyte was 50 mM. The cyclic voltammetry in the oxidation region was recorded with 100 mV/s scan rate under Ar using glassy carbon disc electrode (*d* = 3 mm) as working electrode, platinum wire as counter electrode and Ag/AgCl as reference electrode.

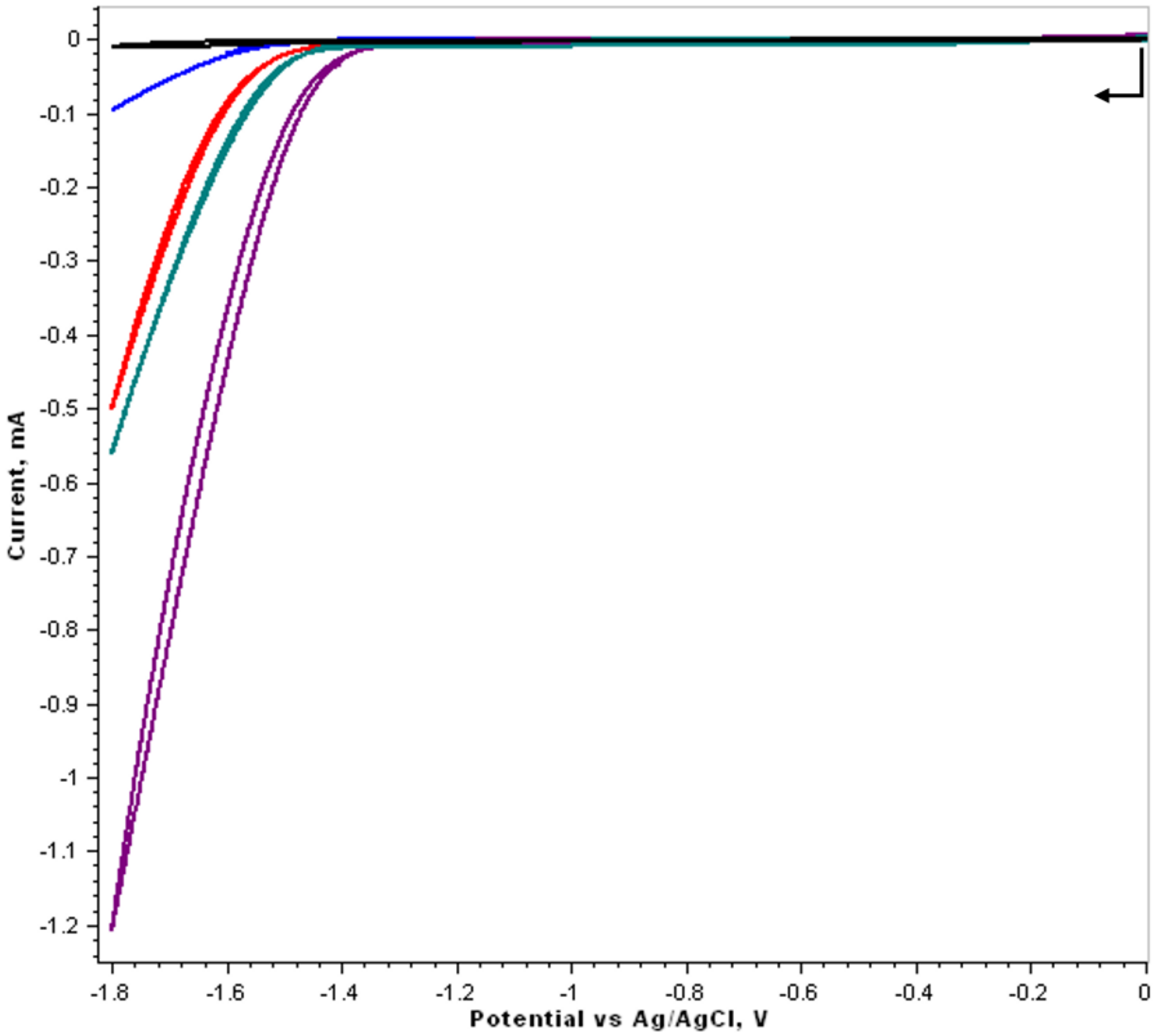

Figure 4. Cyclic voltammograms of **1a** (red), **2a** (blue), ***n*-Bu$_4$NCl** (black), **1a + *n*-Bu$_4$NCl** (green), and **2a + *n*-Bu$_4$NCl** (purple) recorded in MeCN – *t*-BuOH (9:1) solution of 0.1 M *n*-Bu$_4$NPF$_6$ (grey). The concentration of each analyte was 50 mM. The cyclic voltammetry in the oxidation region was recorded with 100 mV/s scan rate under Ar using glassy carbon disc electrode (*d* = 3 mm) as working electrode, platinum wire as counter electrode and Ag/AgCl as reference electrode.

5. References

[1] Y. Chen, A. Dubrovskiy, R. C. Larock *Org. Syn.***2012**, 89, 294-306

[2] J. Cui, L. Meng, X. Chi, Q. Liu, P. Zhao, D.-P. Zhang, L. Chen, X. Li, Y. Donga, H. Liu *Chem. Commun.* **2019**, *55*, 4355-4358

[3] A. De Nisi, C. Bergamini, M. Leonzio, G. Sartor, R. Fato, M. Naldi, M. Monari, N. Calonghi, M. Bandini *Dalton Trans.*, **2016**, 45, 1546–1553

[4] B. Rajagopal, C.-H, C. Ching-Cheng, C, P.-C. Lin *Org. Lett.* **2014***,* 16, 14, 3752–3755

[5] a) Y. Xiong, H. W. Moore *J. Org. Chem.***1996**, 61, 26, 9168–9177; b) L. Helmecke, M. Spittler, B. M. Schmidt, C. Czekelius *Synthesis***2021**, 53, 123–134

[6] Y. Jiang, A. B. Diagne, R. J. Thomson, S. E. Schaus *J. Am. Chem. Soc.* **2017***,* 139, 1998–2005

[7] A. De Nisi, C. Bergamini, M. Leonzio, G. Sartor, R. Fato, M. Naldi, M. Monari, N. Calonghi, M. Bandini *Dalton. Trans.* **2016**, 45, 1546–1553

[8] J. Xu, R. Tong *Green. Chem.* **2017**, 19, 2952–2956

[9] D. Lasányi, G. L. Tolnai *Org. Lett.* **2019***,* 21, 24, 10057–10062

[10] S. Palakhachane, Y. Ketkaew, N. Chuaypen, J. Sirirak, J. Boonsombat, S. Ruchirawat, P. Tangkijvanich, A. Suksamrarn, P. Limpachayaporn*Bioorg. Chem.* **2021**, 112, 104831

[11] H. Cailuan, M. Lixia, L. Guoxun, W. Wenqian, X. Yuxuan, Z. Chuhao Patent nr CN119462384A

[12] S. S. Chandankar, S. Raghavan *Org. Lett.* **2020**, 22, 653–655

[13] D. Ormerod, A. Buekenhoudt, B. Bongers, T. Baramov, J. Hassfeld*Org. Process Res. Dev.***2018** 22 (11), 1509-1517

[14] V. M. Lau, W. C.Pfalzgraff, T. E. Markland, M. W. Kanan *J. Am. Chem. Soc.* **2017**, 139, 11, 4035–4041

[15] D. Cheng, J. Peng. Y. Lv, D. Su, D. Liu, M. Chen, L. Yuan, X. Zhang *J. Am. Chem. Soc.* **2019**, 141, 15, 6352–6361

[16] L.-Z. Qin, Y.-L. Cheng, X. Wen, Q.-L. Xu, L. Zhen *Tetrahedron*, **2021**, 77, 131742

[17] A. B. Dapkekar, S. K. Nag, G. Satyanarayana *Adv. Synth. Catal.* **2025**, 367, e202401349.

[18] E. O. Abramova, A. V. Paderina, S. O. Slavova, E. A. Kostenko, E. V. Eliseenkov, S. K. Petrovskii, A. Yu. Gitlina, V. P. Boyarskiy, E. V. Grachova*Inorg. Chem.* **2021**, 60, 24, 18715–18725

[19] S. Lai, B. Wang, K. Sun, F. Li, Q. Liu, X.-A. Yu, L. Jiang, L. Wang *Molecules* **2024**, 29, 8, 1845

[20] X. Chen, L.Li, C. Pei, J.Li, D. Zou, Y. Wu, Y. Wu *J. Org. Chem.***2021**, 86, 3, 2772–2783

[21] A. Lapini, P. Fabbrizzi, M. Piccardo, M. di Donato, L. Lascialfari, P. Foggi, S. Cicchi, M. Biczysko, I. Carnimeo, F. Santoro, C. Cappellide, R. Righini*Phys. Chem. Chem. Phys.* **2014**, 16, 10059–10074

[22]N. Savadkouhi, Z. Mazarei, M. Esmaeelzadeh, P. Salehi, H. Rafati *Bioorg. Med. Chem.* **2021**, 40, 127907

[23] Y. Yang, X. Meng, B. Zhu, Y. Jia, X.Cao, S. Huang *Eur. J. Org. Chem.***2019**, 1166-1169.

[24] X.-X. Li, L.-L. Zhu, W. Zhou, Z. Chen *Org. Lett.* **2012**, 14, 2, 436–439

[25] L. García, J. Sendra, N. Miralles, E. Reyes, J. J. Carbó, J. L. Vicario, E. Fernández *Chem. Eur. J.* **2018**, 24, 53, 14059–14063

[26] P. Sarathi B. Yafia, K. Mirza, V, Ntuli, Y. Soorni, R. Karpoormath, M. Bera *Org. Lett.* **2025**, 27, 9, 2053–2059

[27] T. Watanabe, S. Oishi, N. Fujii, H. Ohno *Org. Lett.* **2007**, 9, 23, 4821–4824

[28] F. Calogero, L. Wilczek, E. Pinosa, A. Gualandi, R. Dorta, A. Herrera, Y. Dai, A. Rossignol, F. Negri, Z. Ziani, A. Fermi, P. Ceroni, P. G. Cozzi *Angew. Chem. Int. Ed.* **2024**, 63, e202411074

[29] A. Long, D. Li, B. Yan, Y. Yuan, H. Liu, L. Yang, Y. Chen, S. He *RSC. Adv.* **2025**, 15, 11770–11773

[30] D. Jankovič, D. Jankovič, J. Košmrlj, M. Gazvoda *J. Org. Chem.* **2025**, 90, 9, 3480–3484

[31] W. A. Carrick, M. C. Eng, S. Liu, J. S. Johnson*Org. Lett.* **2025**, 27, 45, 12448–12452

[32] C. Zhu, R. Wang, J. R. Falck*Org. Lett.* **2012**, 14, 13, 3494–3497

[33] H.-C. Lin, G. J Knox, C. M Pearson, C. Yang, V. Carta, T. N Snaddon*Angew. Chem. Int. Ed.* **2022**, *61*, e202201753

[34] M. Giedyk, J. Turkowska, S. Lepak, M. Marculewicz, K. ó Proinsias, D. Gryko *Org. Lett.* **2017**, 19, 10, 2670–2673

[35] C. Margarita, D. Di Francesco, H. Tuñon, I. Kumaniaev, C. Jansson Radaa, H. Lundberg *Green Chem.,* **2023***,* 25*,* 2401-2408

[36] B. Kokić, Ž. Selaković, A. M. Nikolić, A. Andrijević, B. Anđelković, V. Ajdačić, I. M. Opsenica*Eur. J. Org. Chem.***2022**, art. no. E202201112

6. NMR spectra

tert-butyl 5-methoxy-3-(2-(*N*-(prop-2-yn-1-yl)acetamido)ethyl)-1H-indole-1-carboxylate (**1h**)

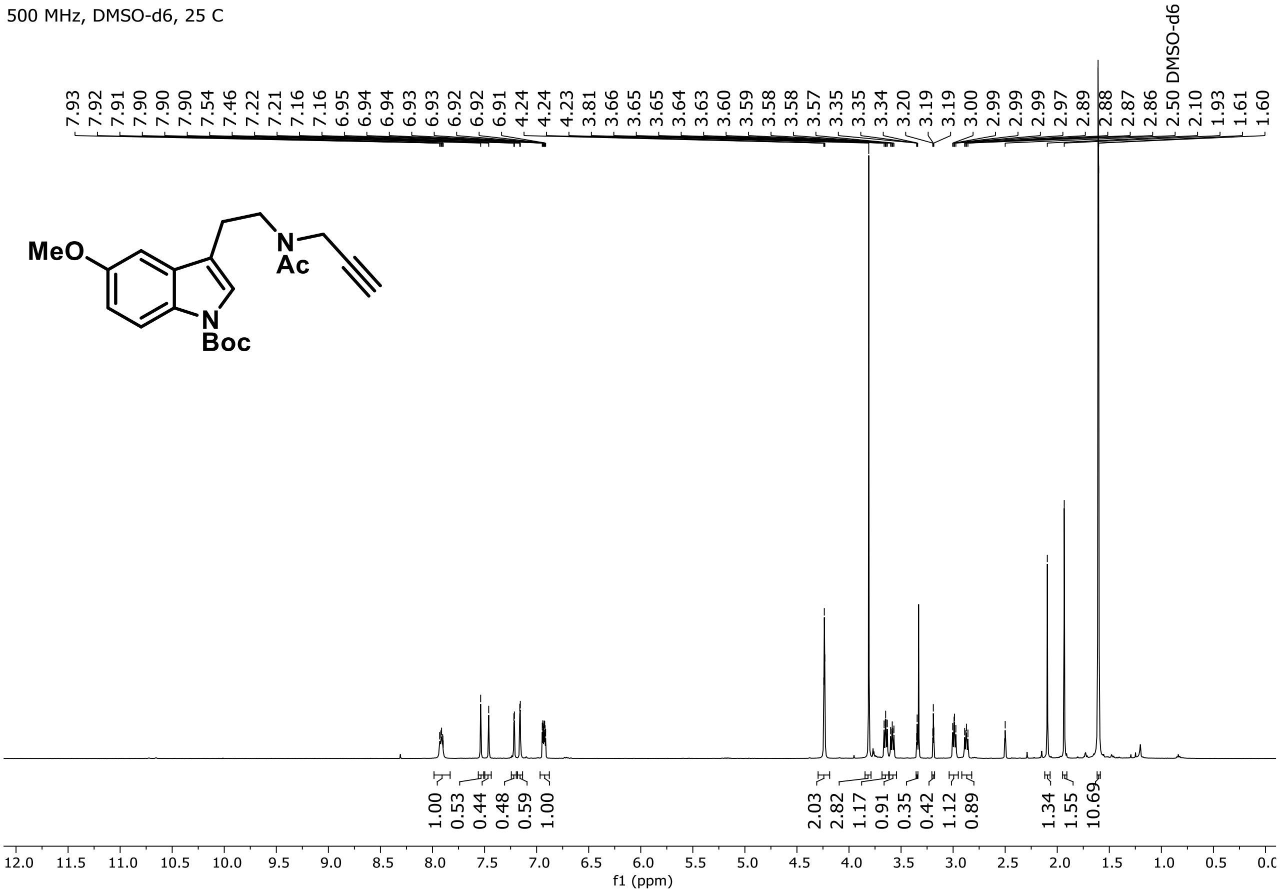

500 MHz, DMSO d-6, 80 C

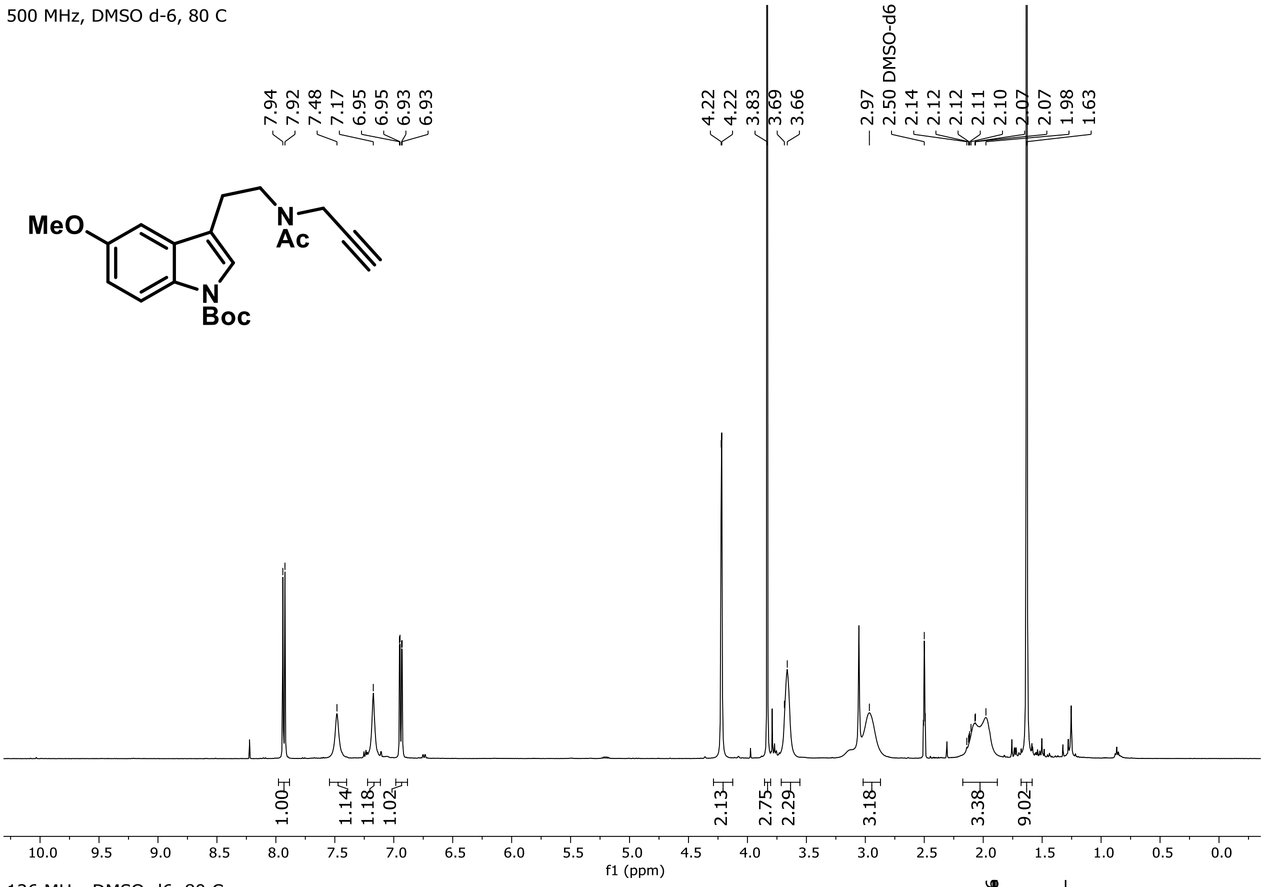

126 MHz, DMSO-d6, 80 C

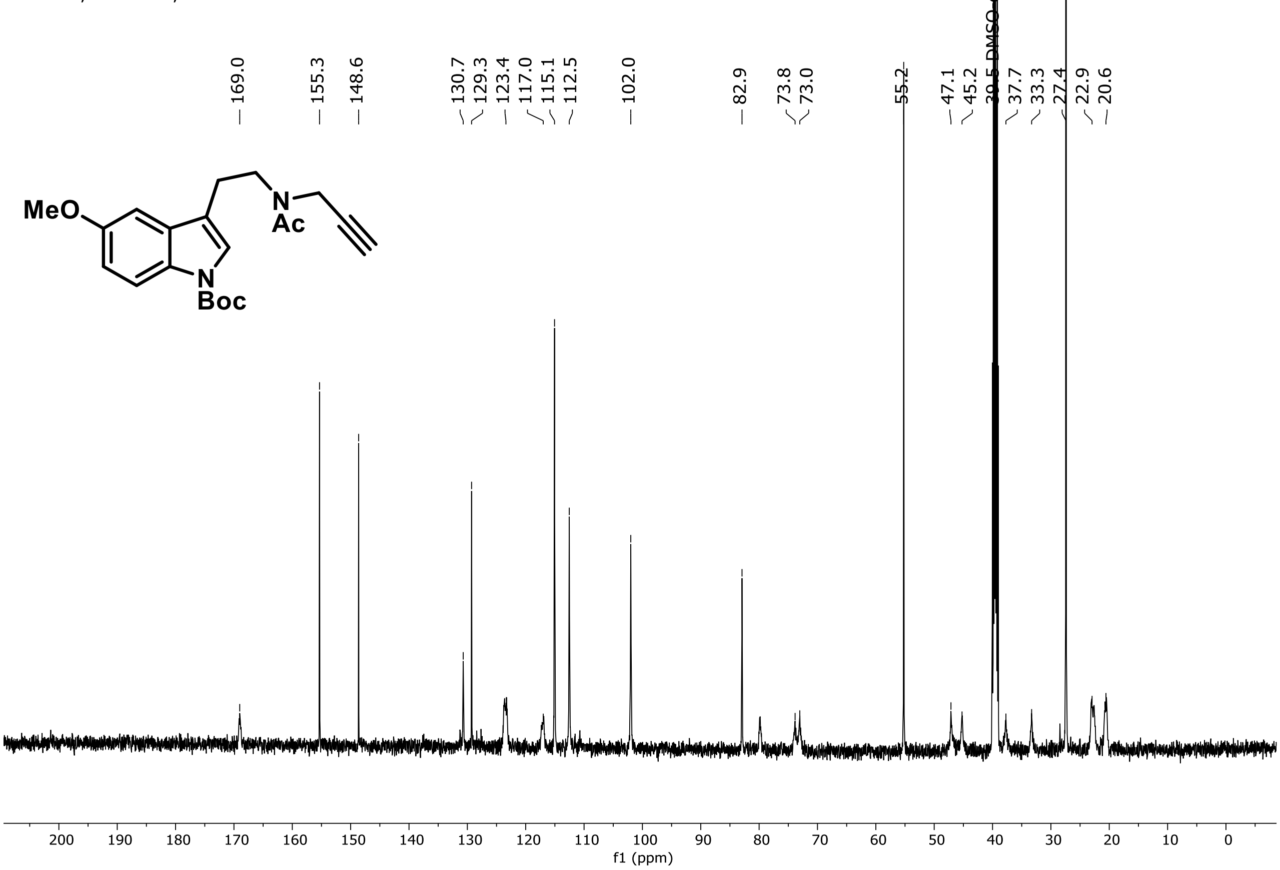

1,3-dimethoxy-2-(prop-2-yn-1-yloxy)benzene (**1p**)

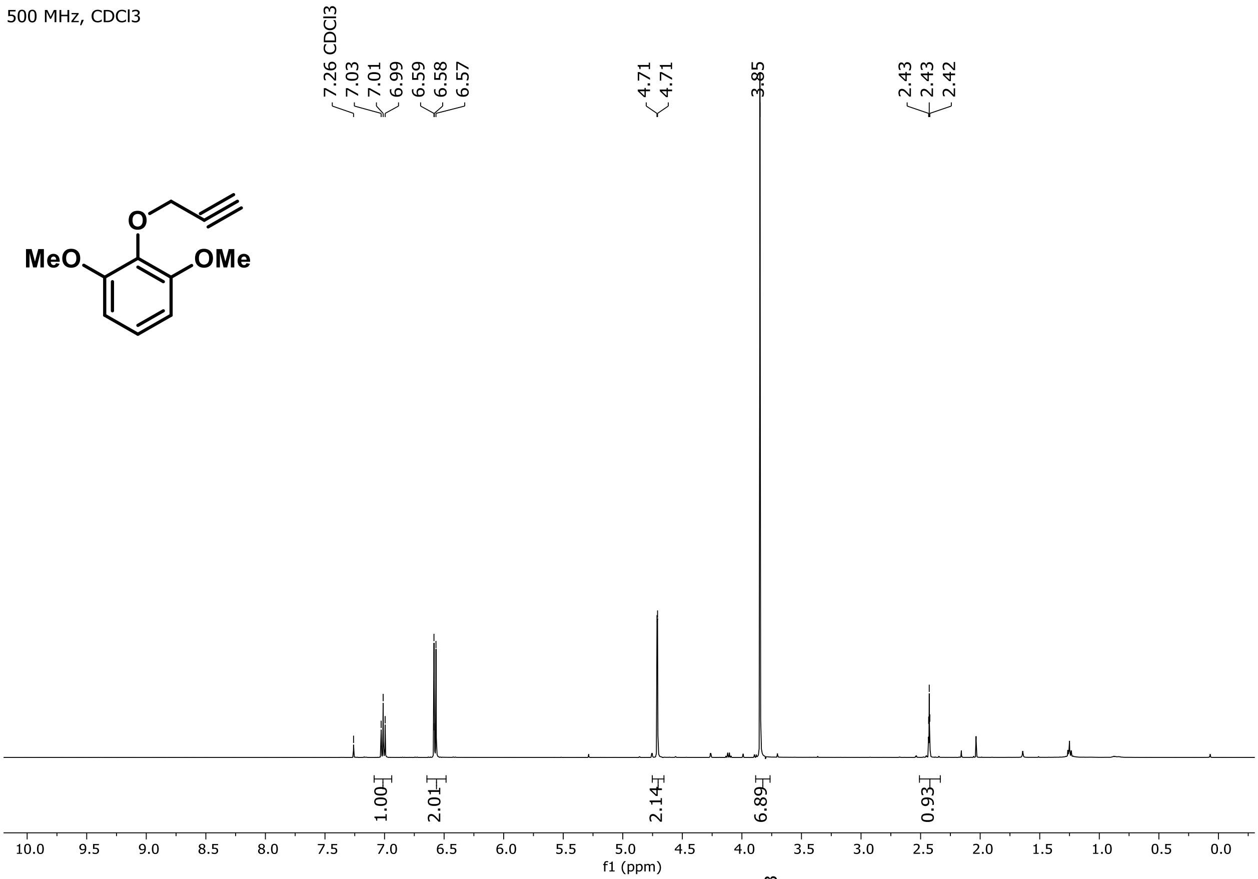

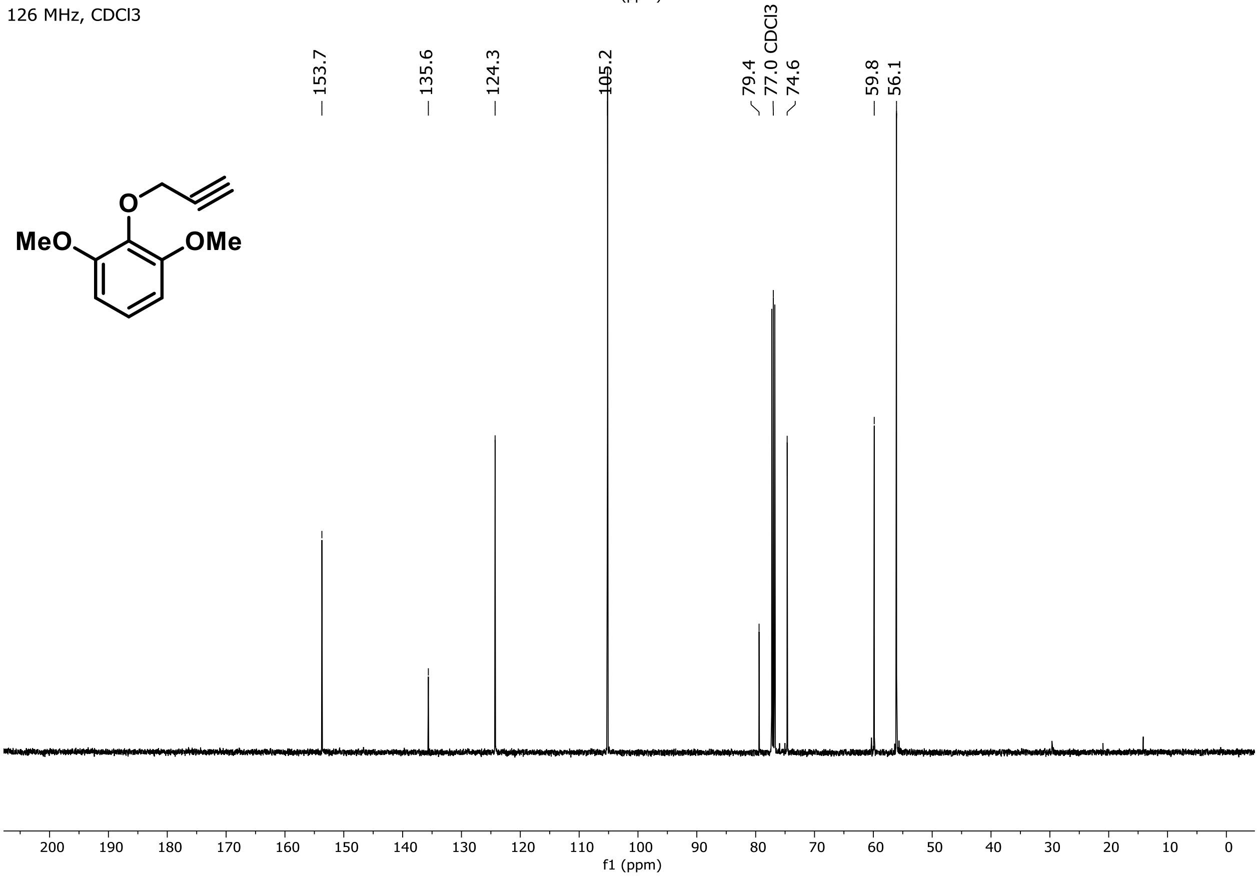

1-(prop-2-yn-1-yloxy)-4-((prop-2-yn-1-yloxy)methyl)benzene (**1t**)

500 MHz, CDCl3

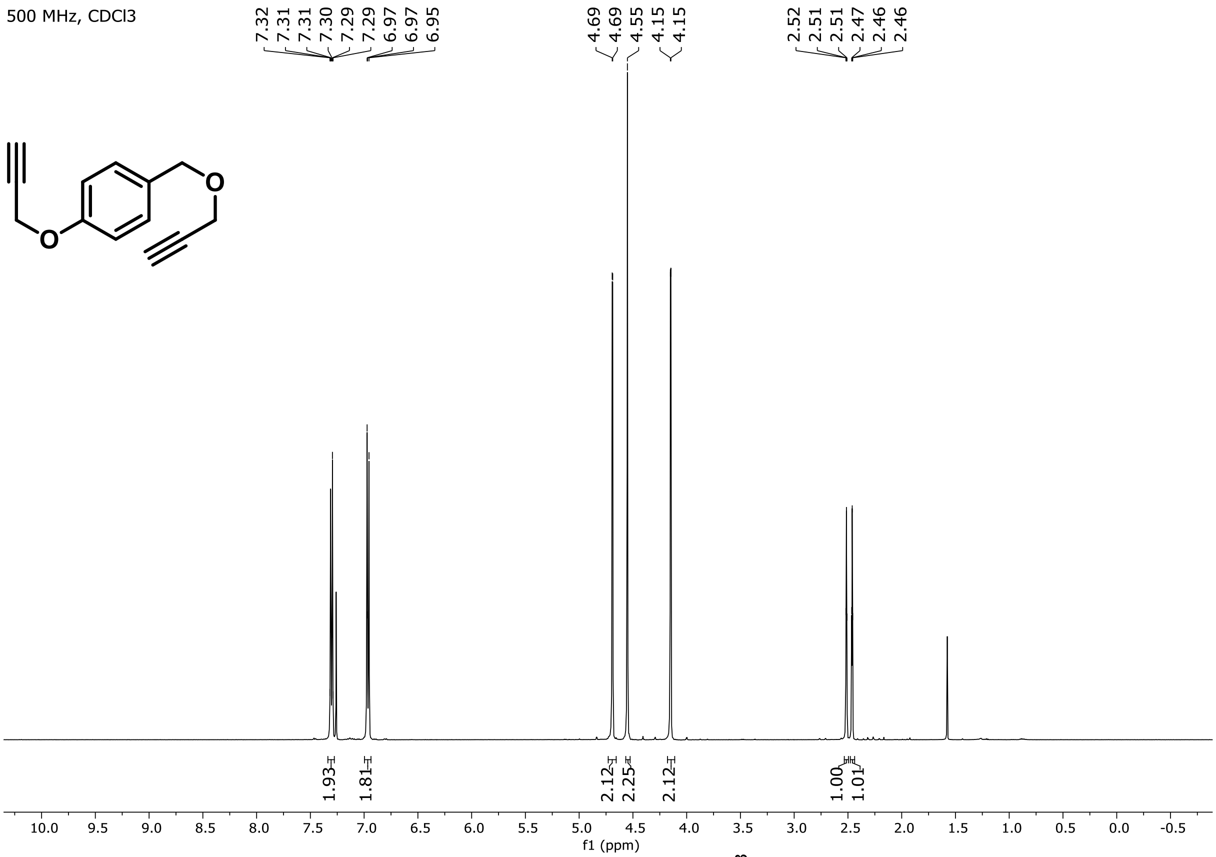

126 MHz, CDCl3

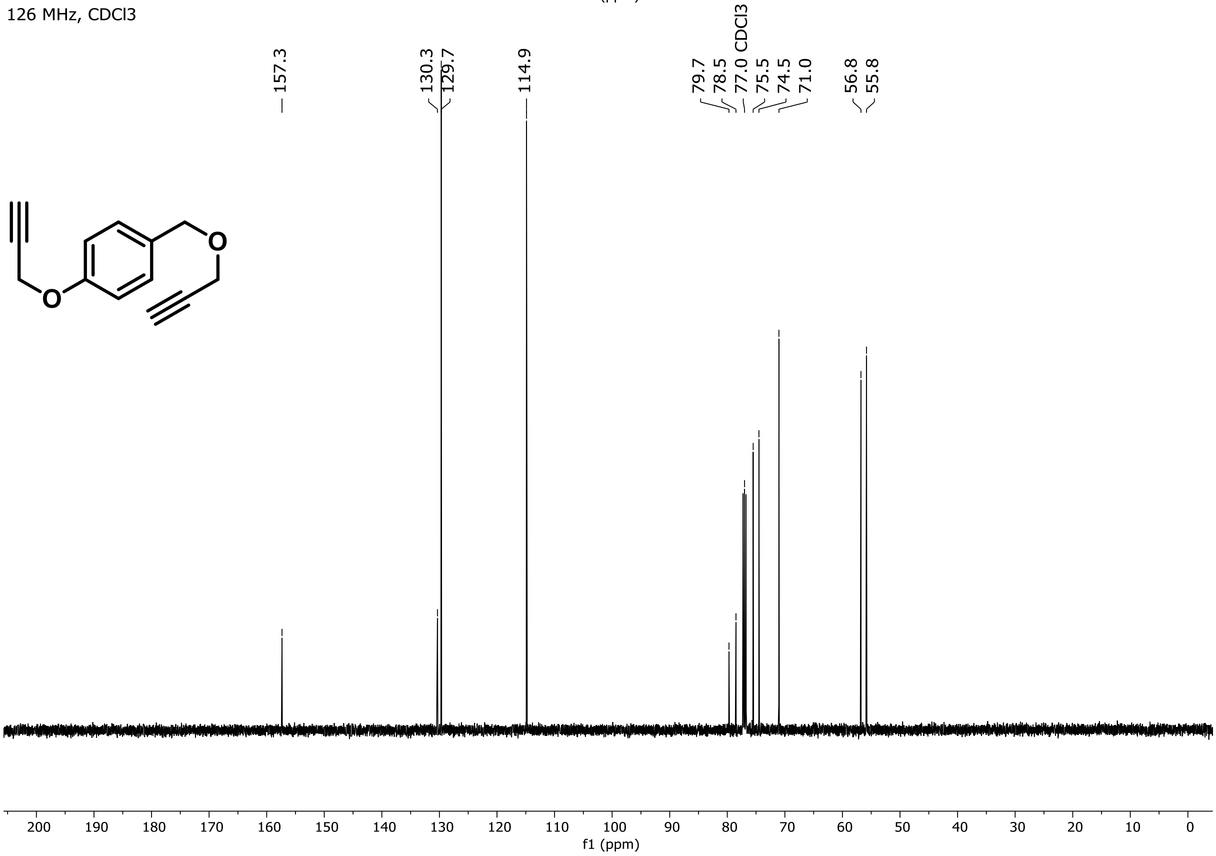

N-(prop-2-yn-1-yl)-*N*-(4-(prop-2-yn-1-yloxy)phenyl)acetamide (**1u**)

500 MHz, CDCl3

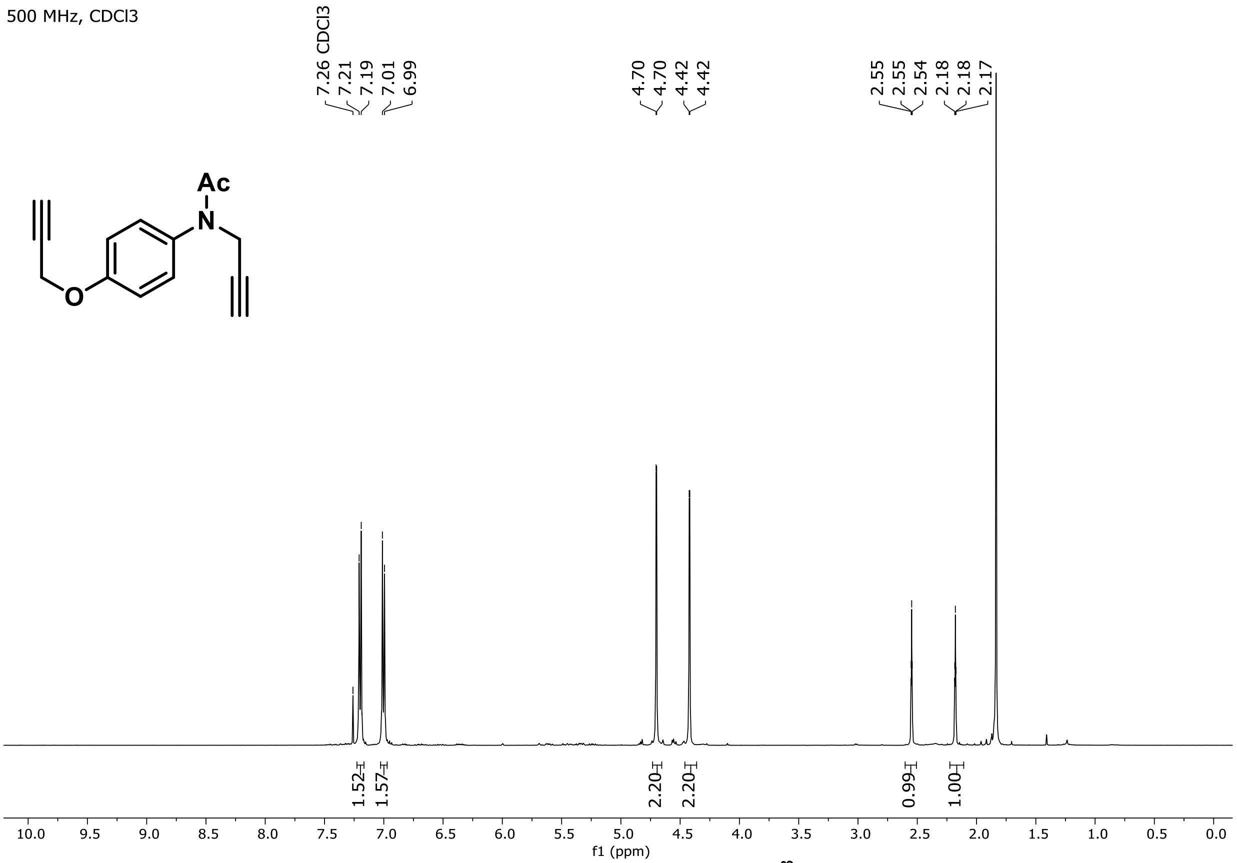

126 MHz, CDCl3

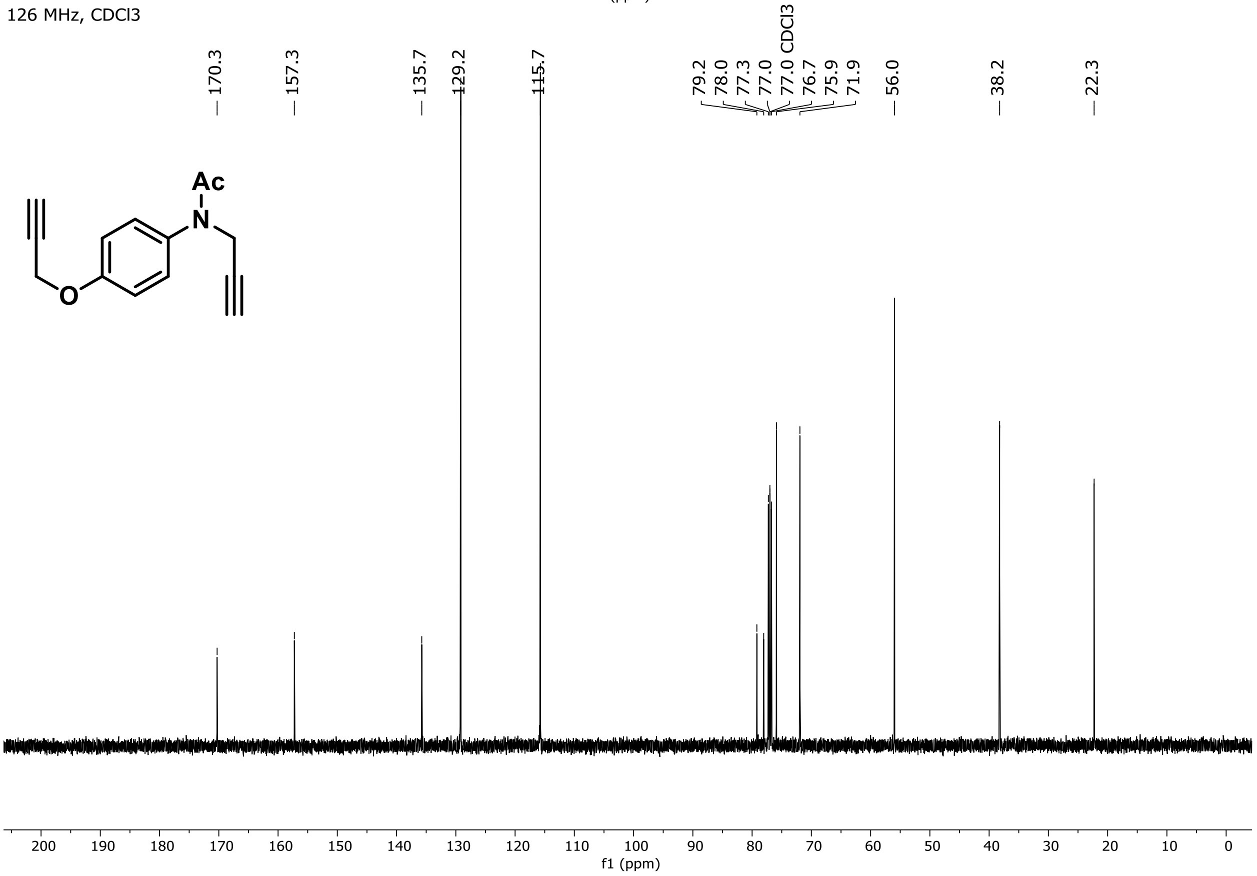

N-(prop-2-yn-1-yl)-*N*-(3-(prop-2-yn-1-yloxy)phenyl)acetamide (**1w**)

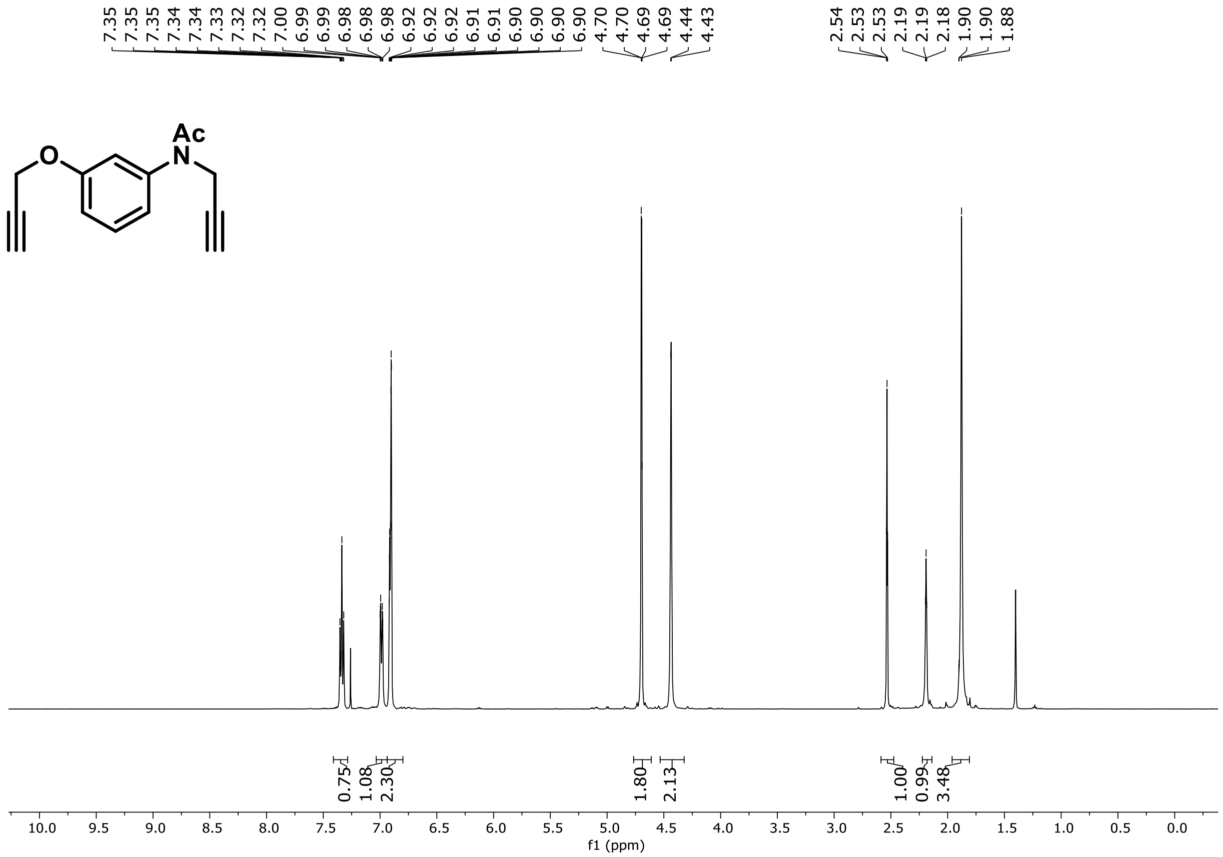

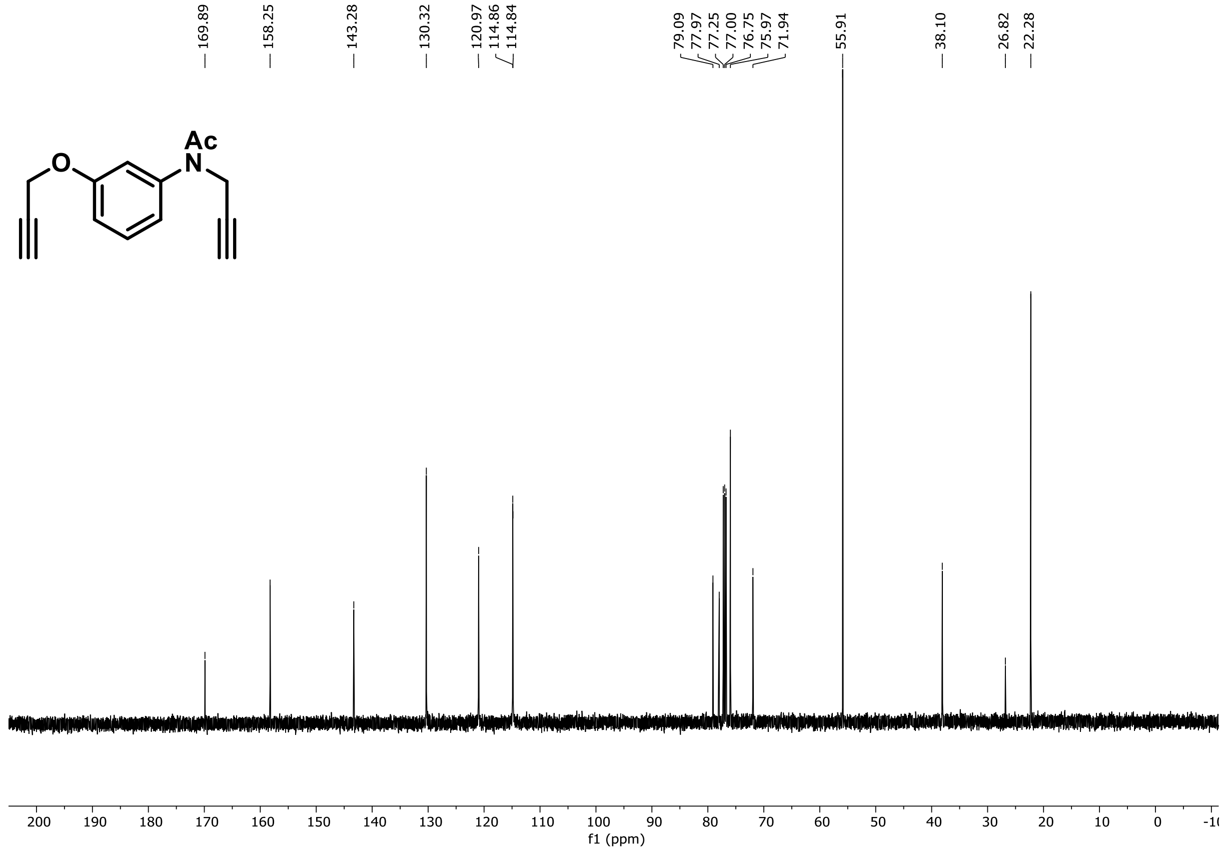

4-methyl-*N*-(prop-2-yn-1-yl)-*N*-(4-(prop-2-yn-1-yloxy)phenyl)benzenesulfonamide (**1x**)

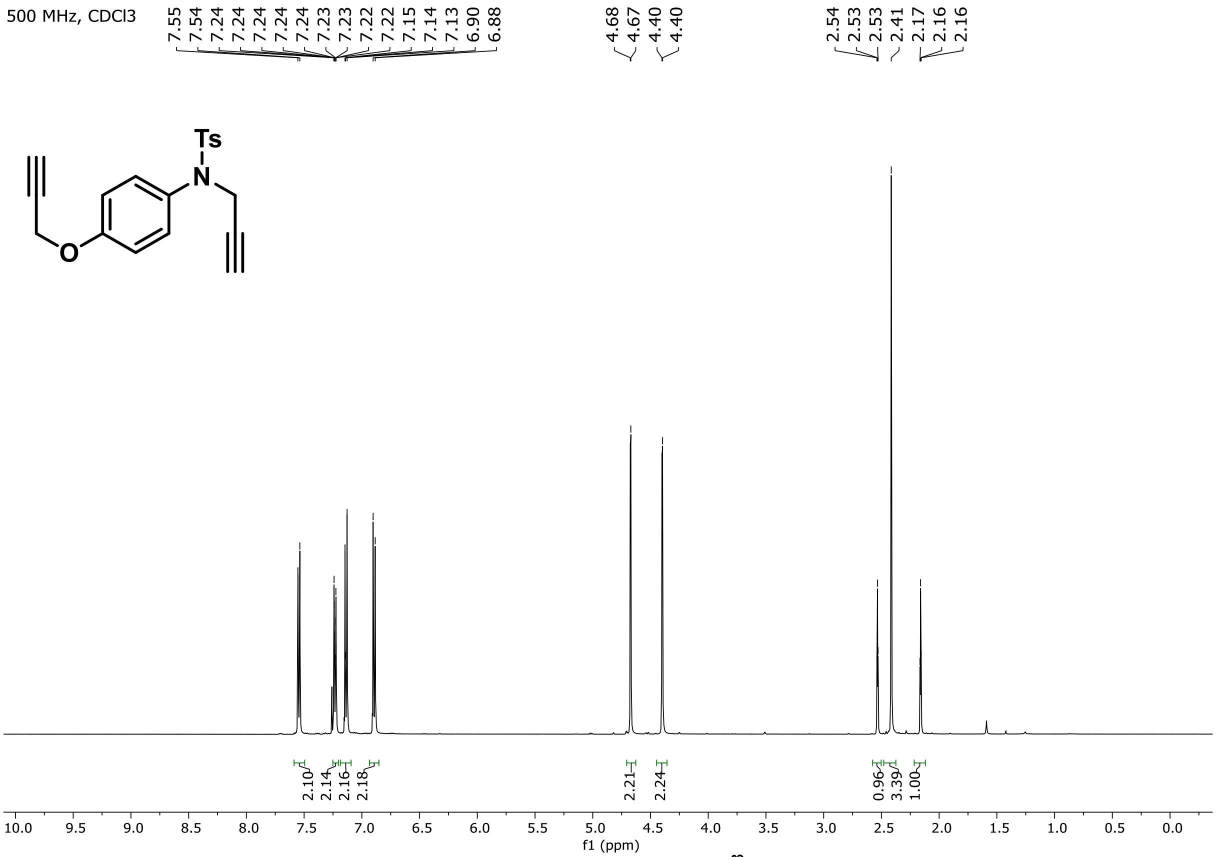

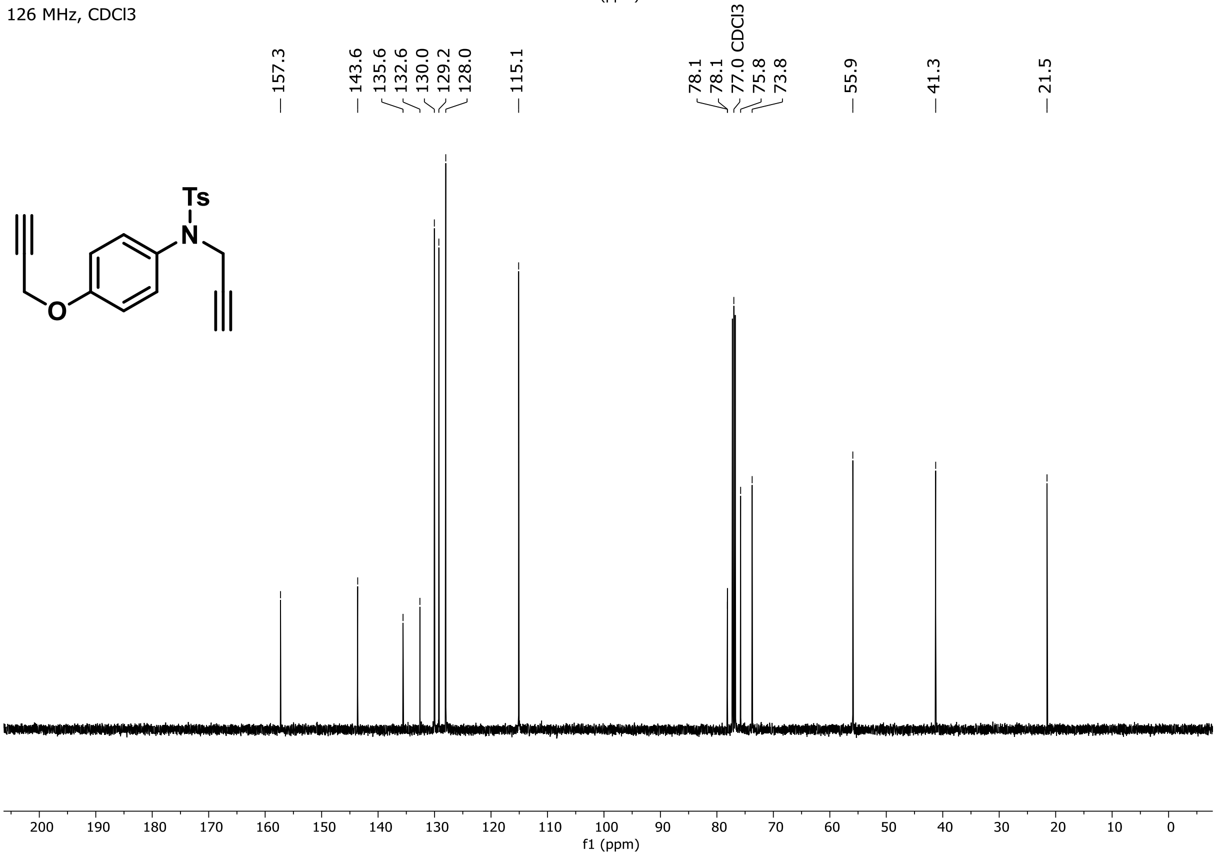

9,10-dioxo-2-(prop-2-yn-1-yloxy)-9,10-dihydroanthracen-1-yl acetate (**S12**)

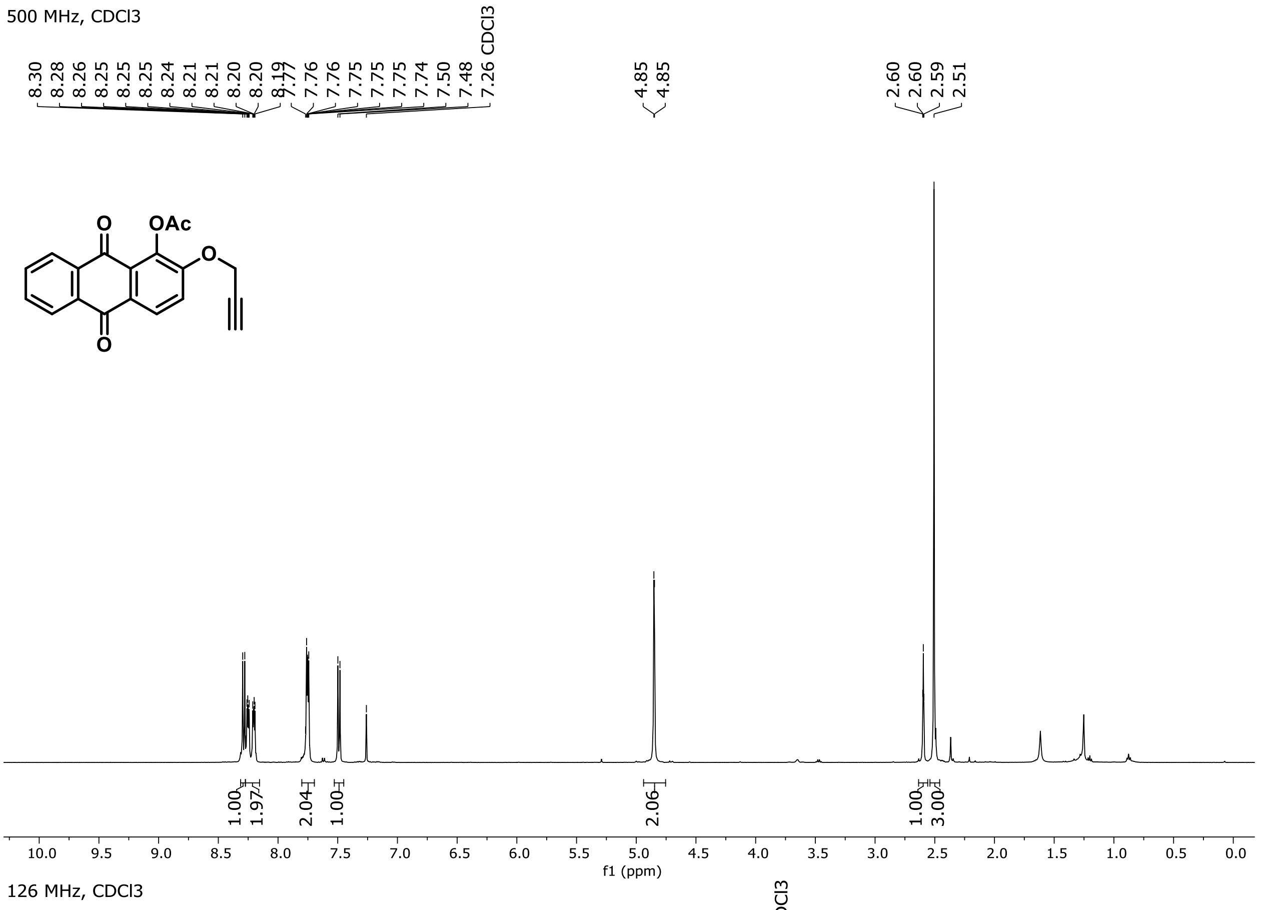

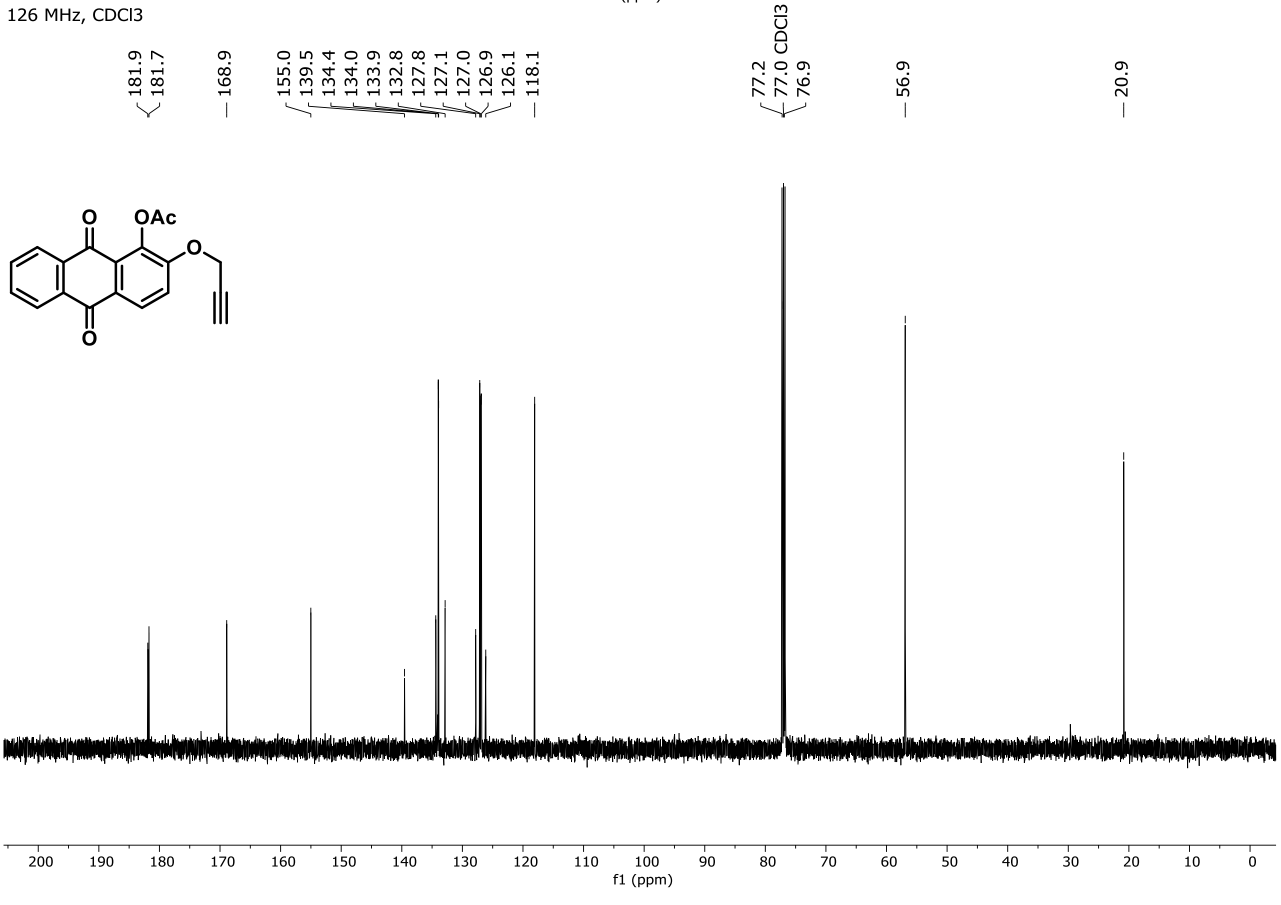

4-methyl-*N*-phenyl-*N*-(propa-1,2-dien-1-yl)benzenesulfonamide (**2a**)

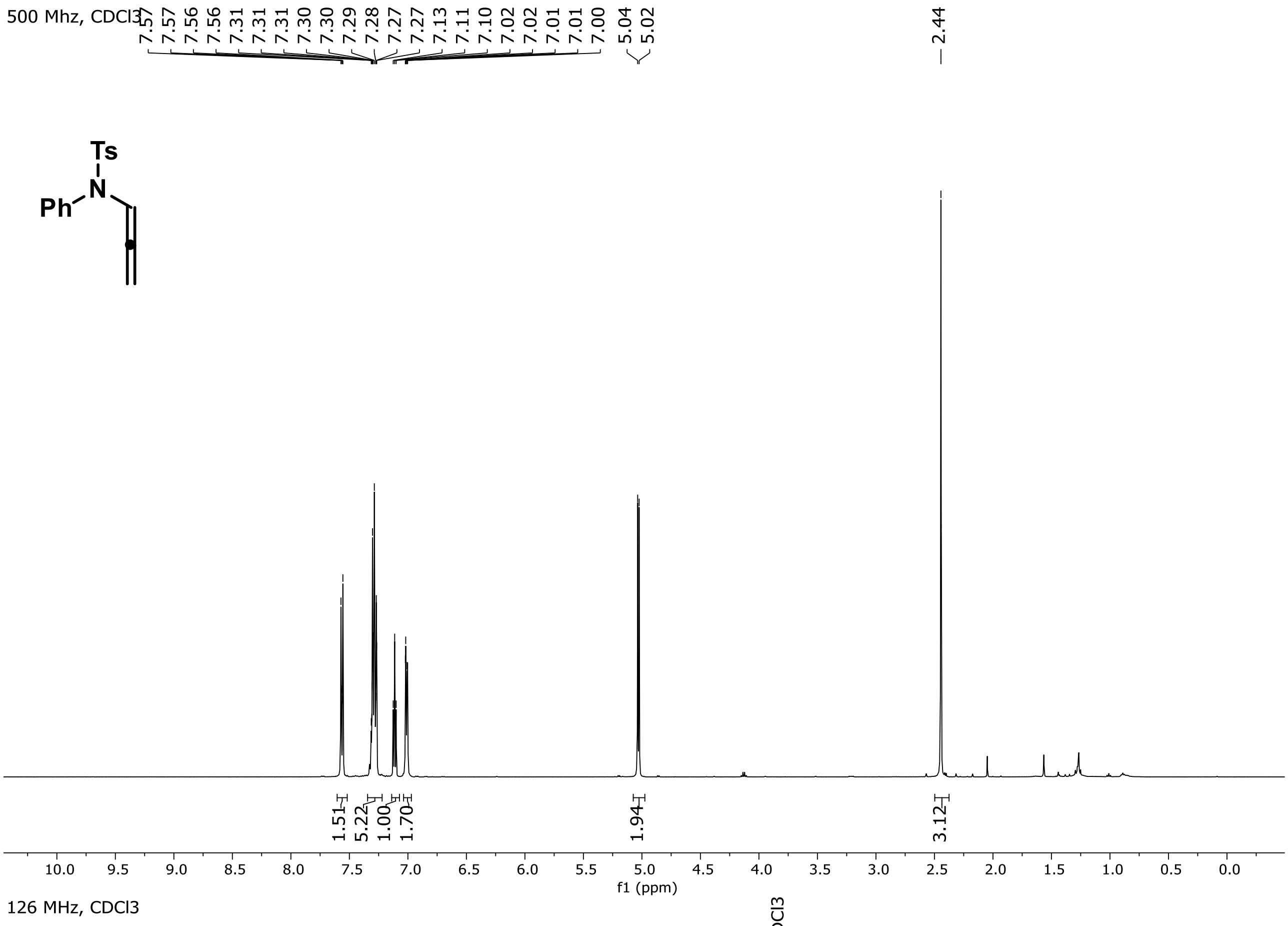

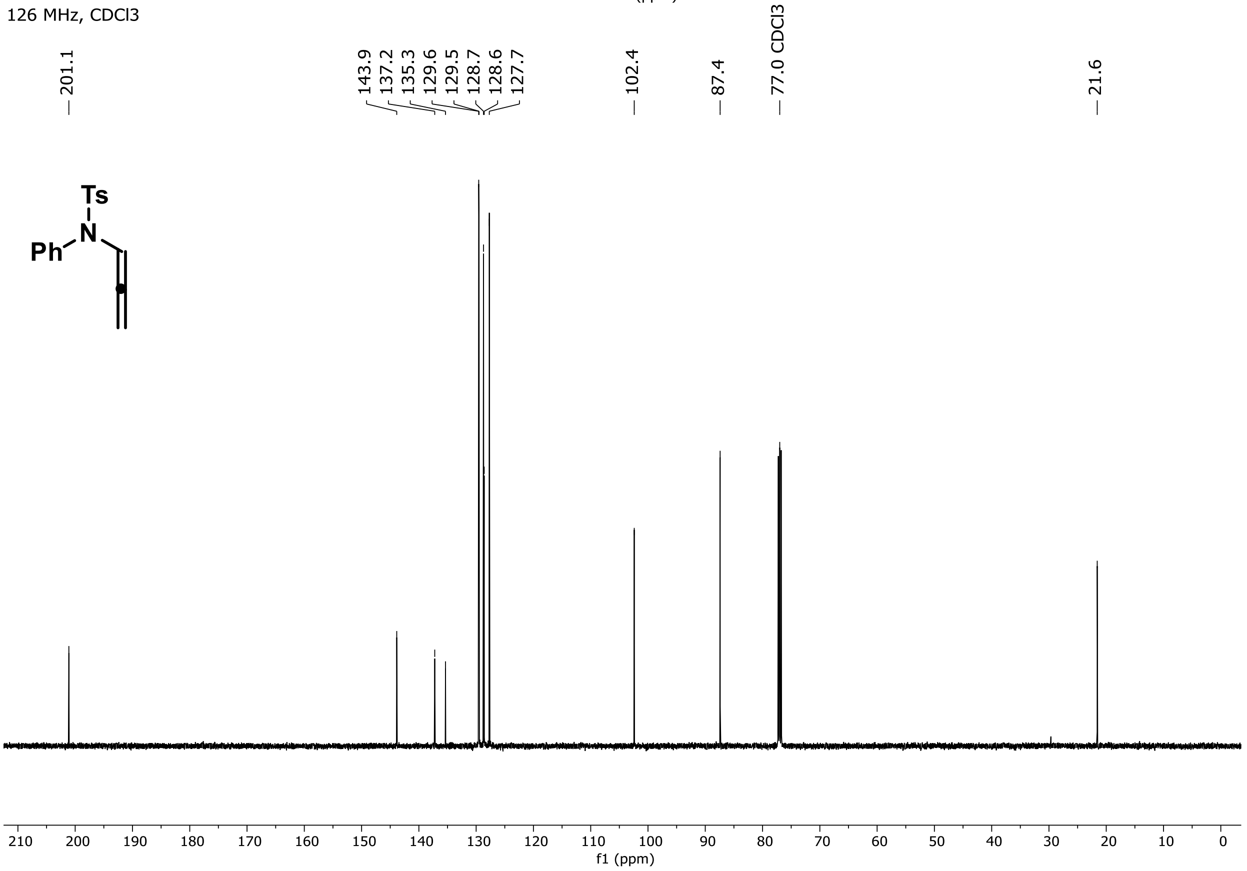

N-phenyl-*N*-(propa-1,2-dien-1-yl)acetamide (**2b**)

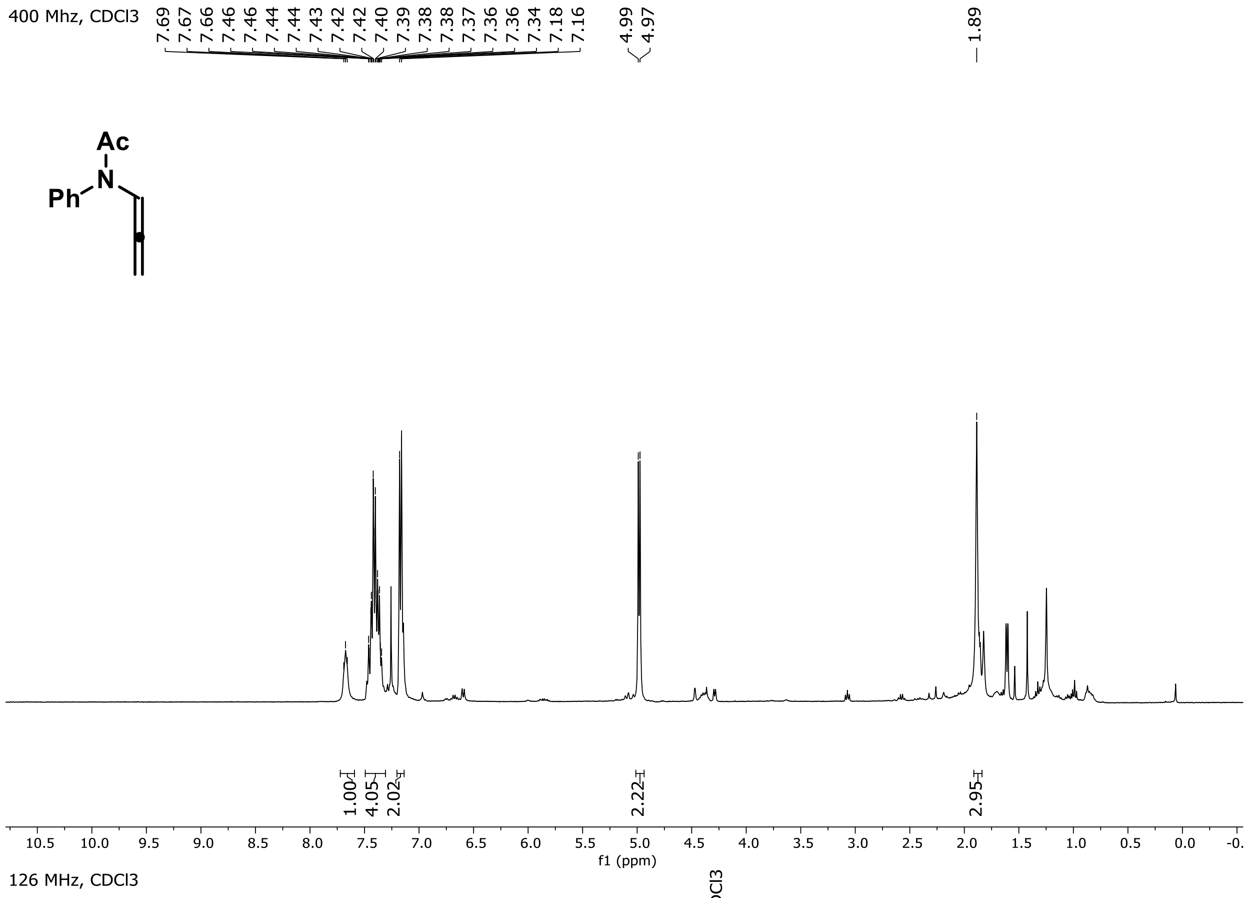

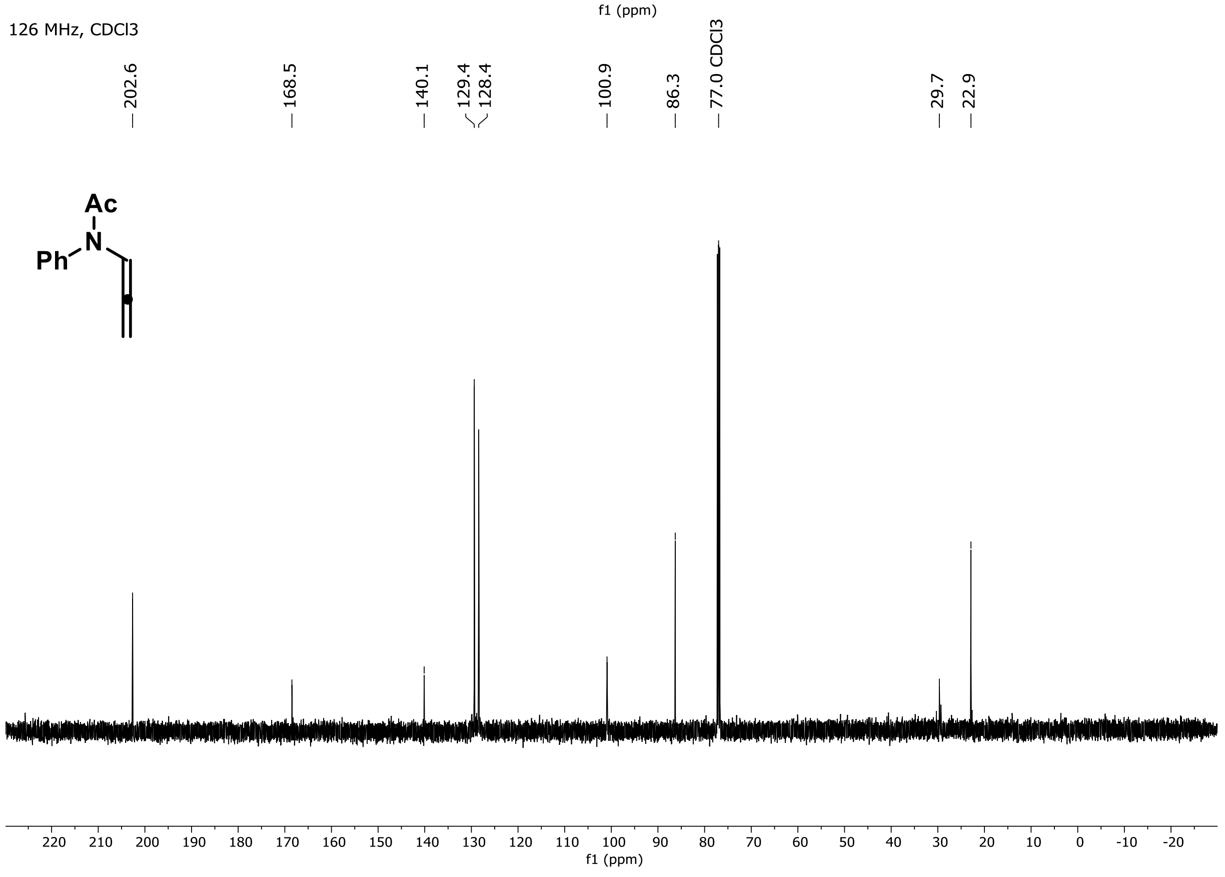

tert-butyl phenyl(propa-1,2-dien-1-yl)carbamate (**2c**)

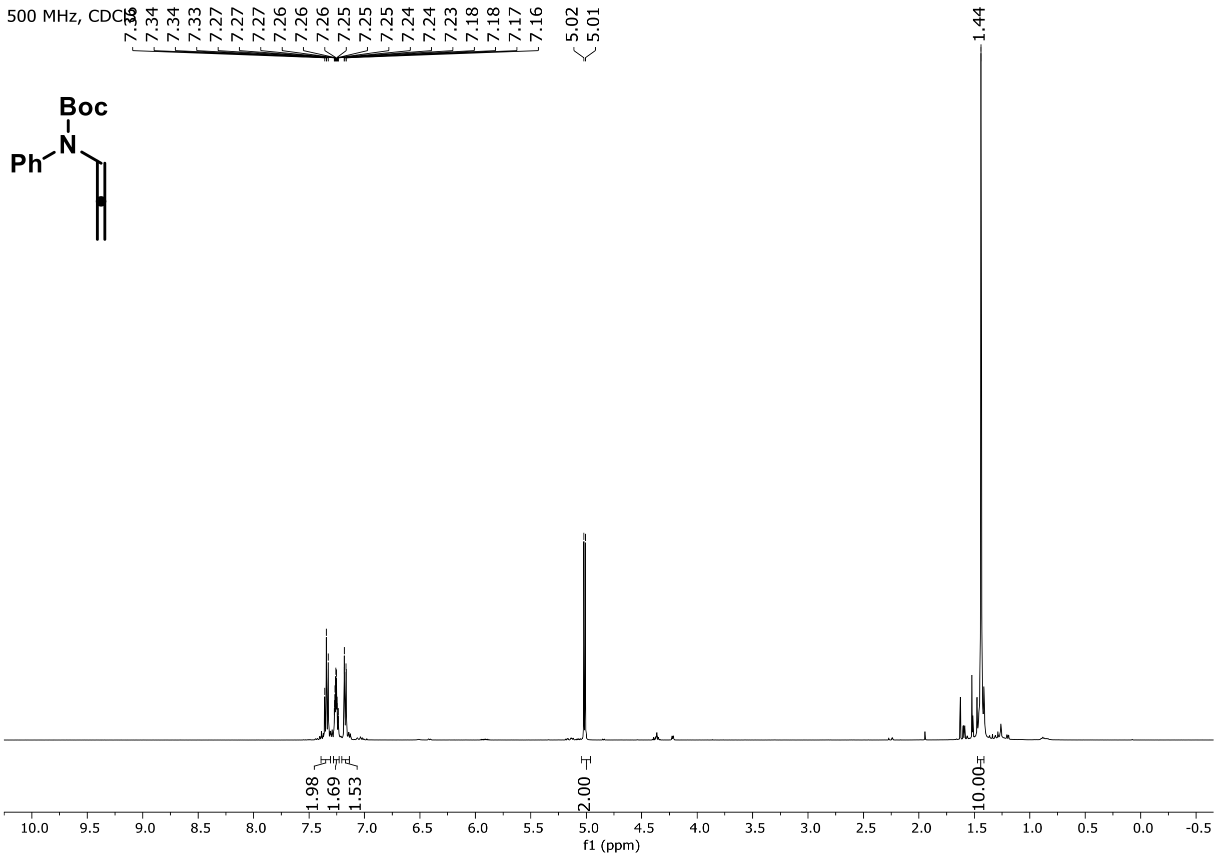

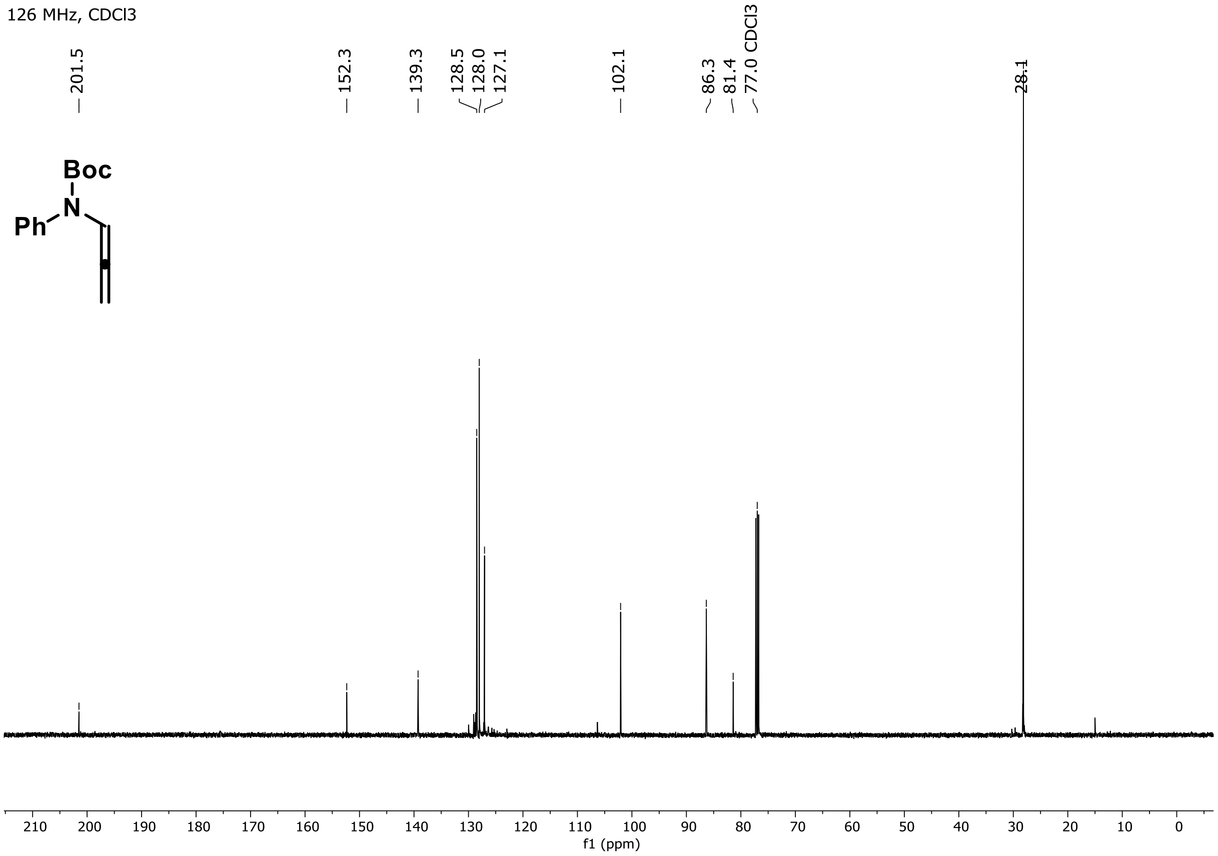

methyl phenyl(propa-1,2-dien-1-yl)carbamate(**2d**)

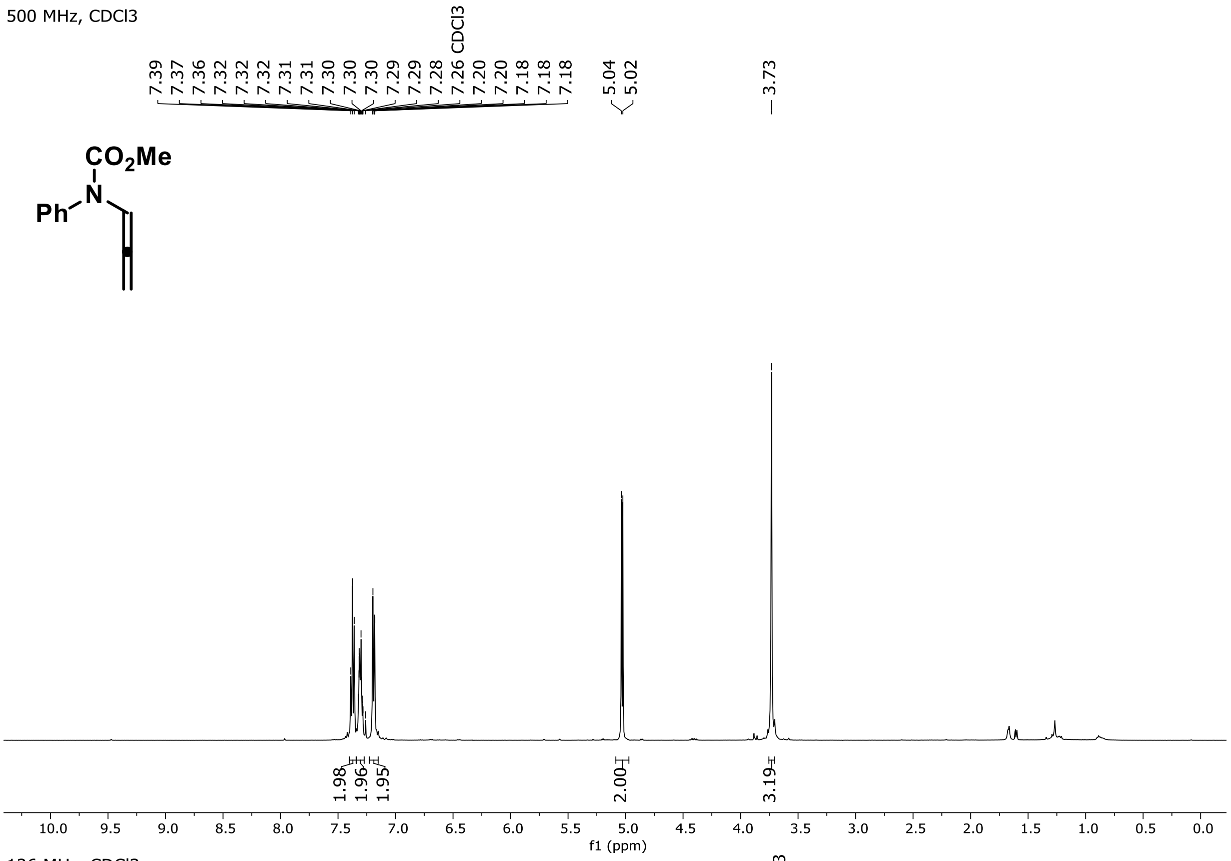

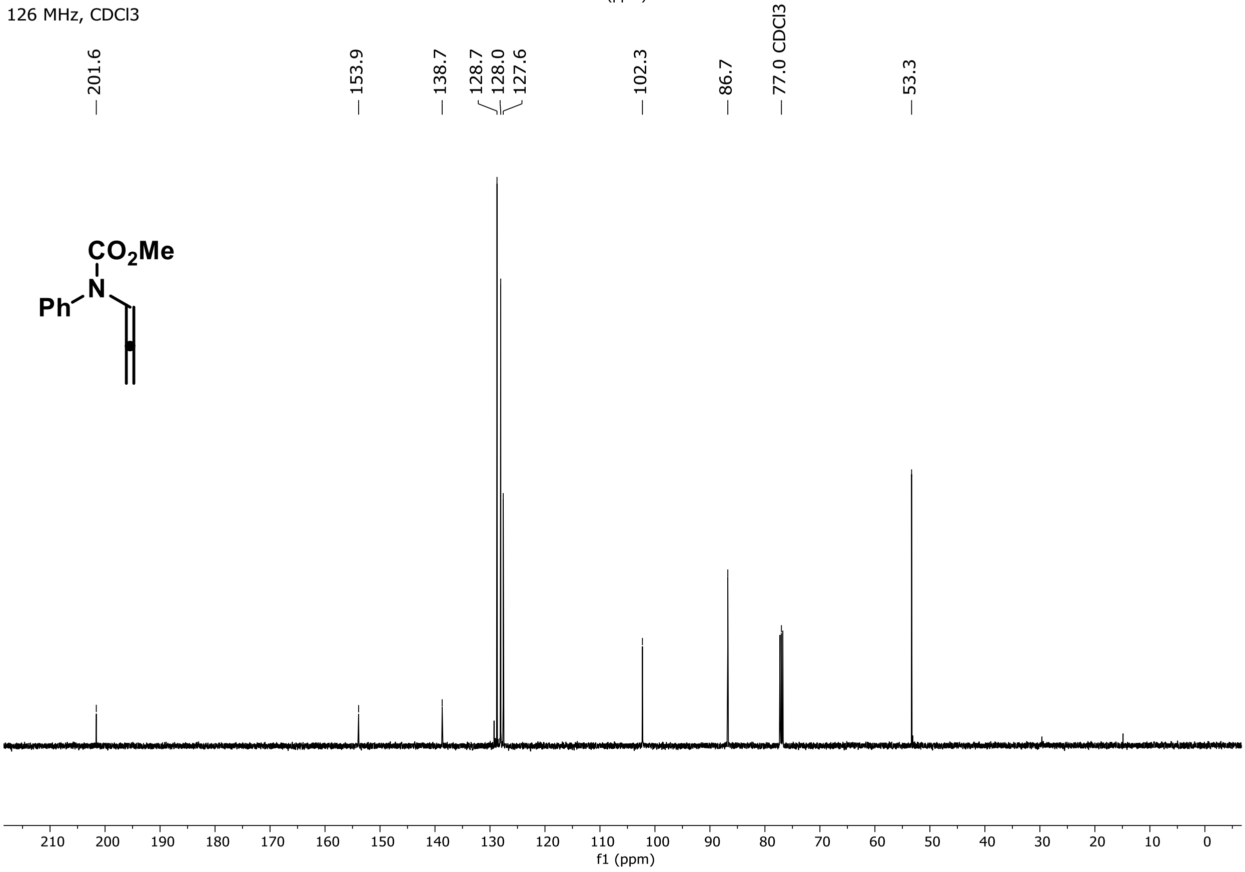

N-phenyl-*N*-(propa-1,2-dien-1-yl)benzamide **(2e)**

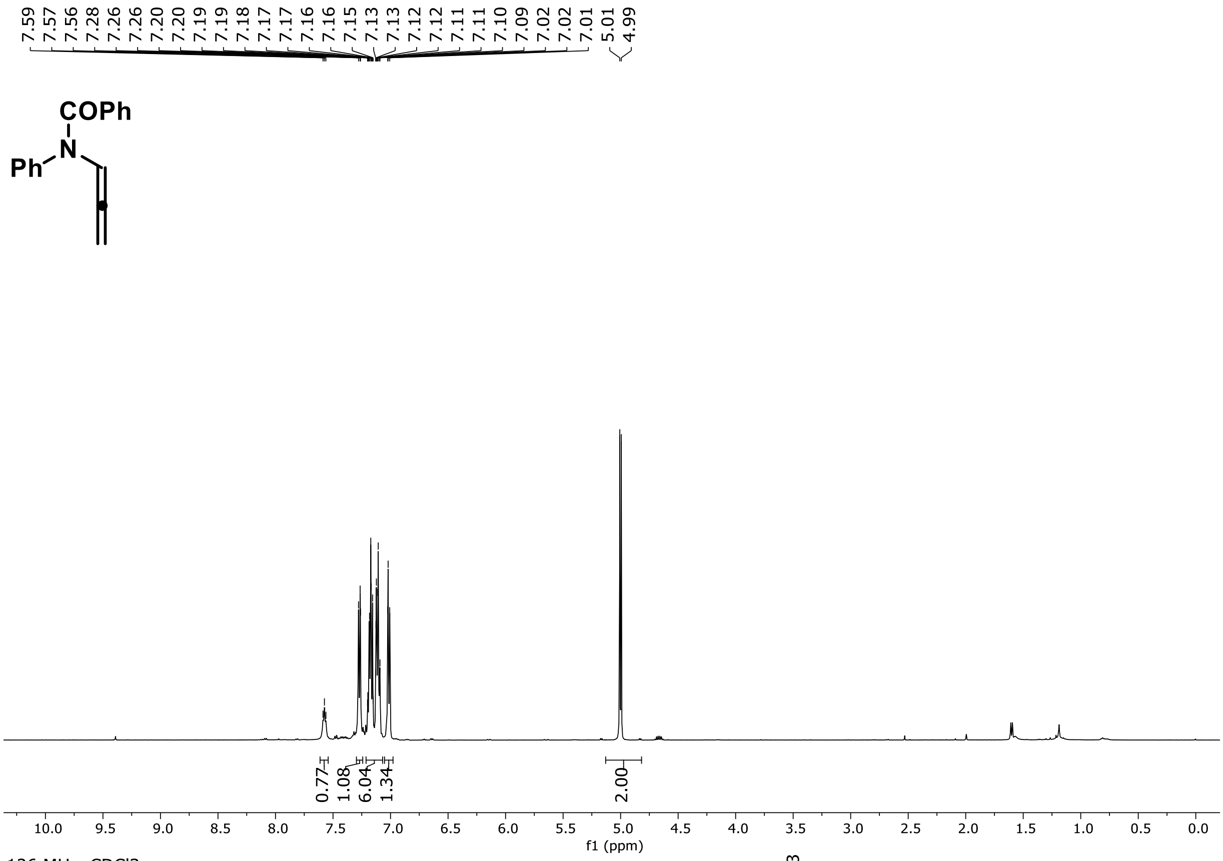

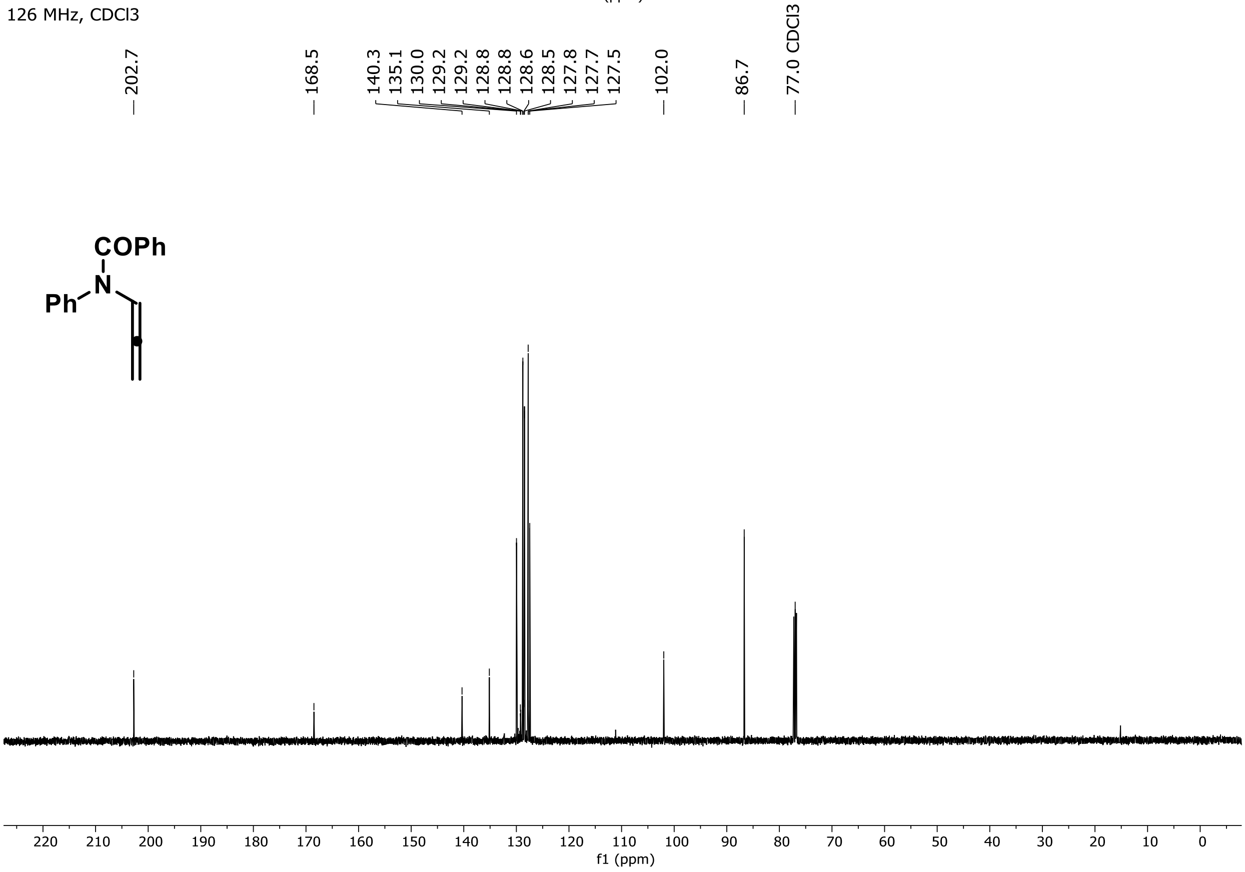

N,4-dimethyl-*N*-(propa-1,2-dien-1-yl)benzenesulfonamide **(2g)**

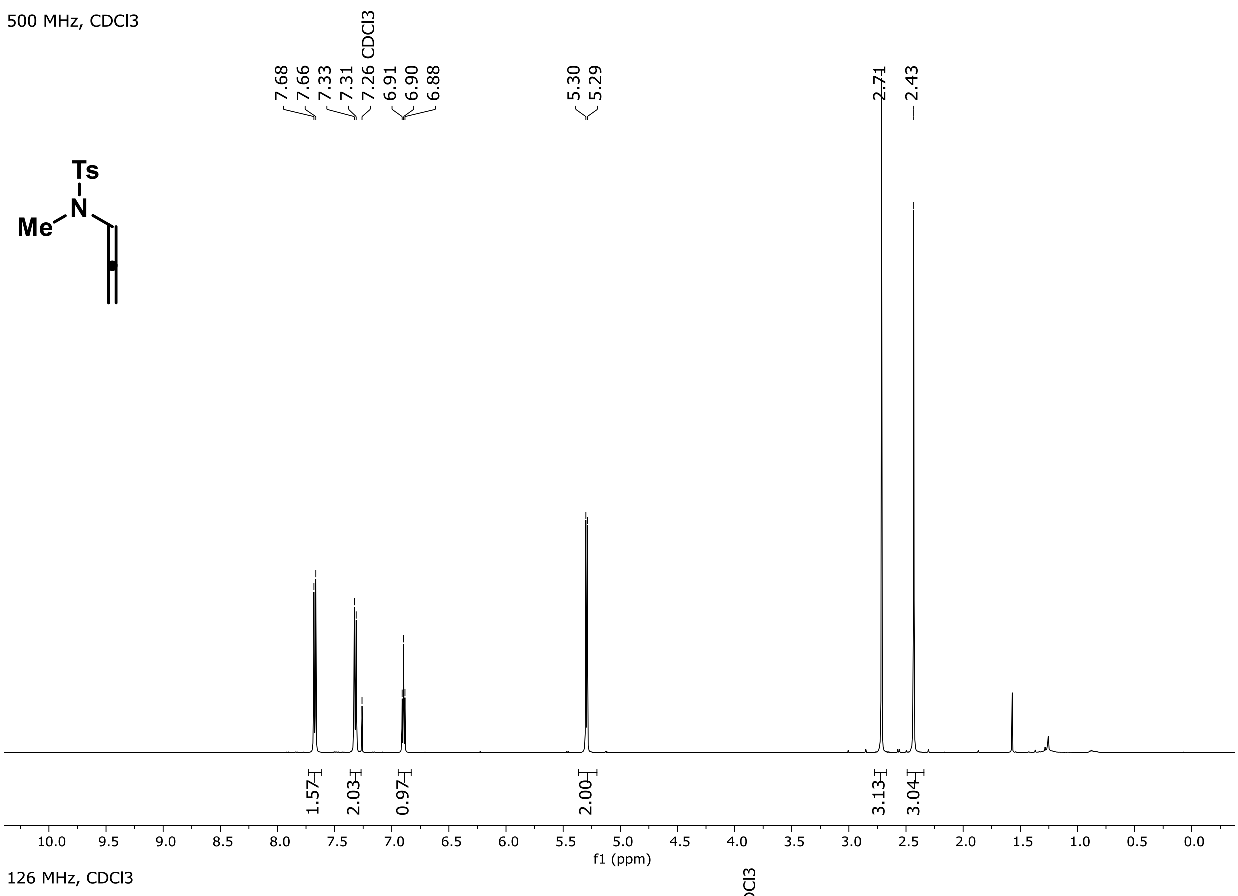

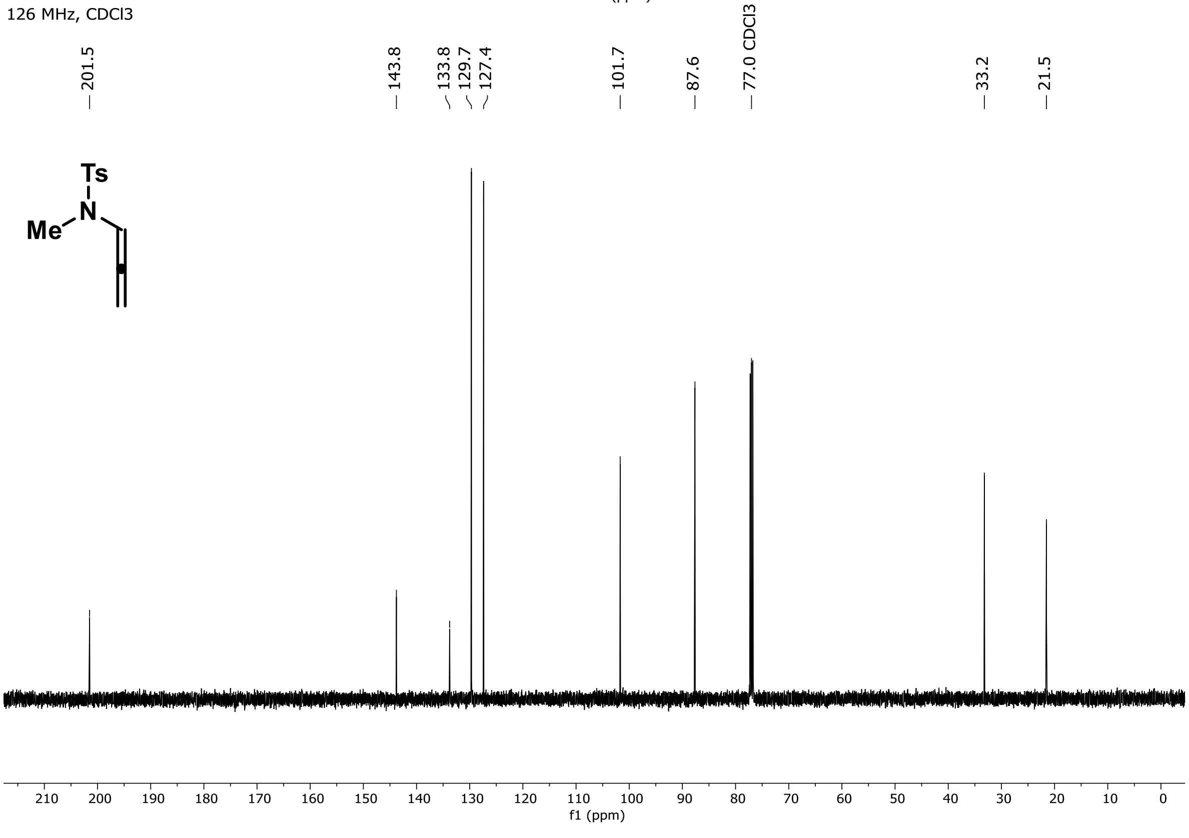

tert-butyl 5-methoxy-3-(2-(N-(propa-1,2-dien-1-yl)acetamido)ethyl)-1H-indole-1-carboxylate (**2h**)

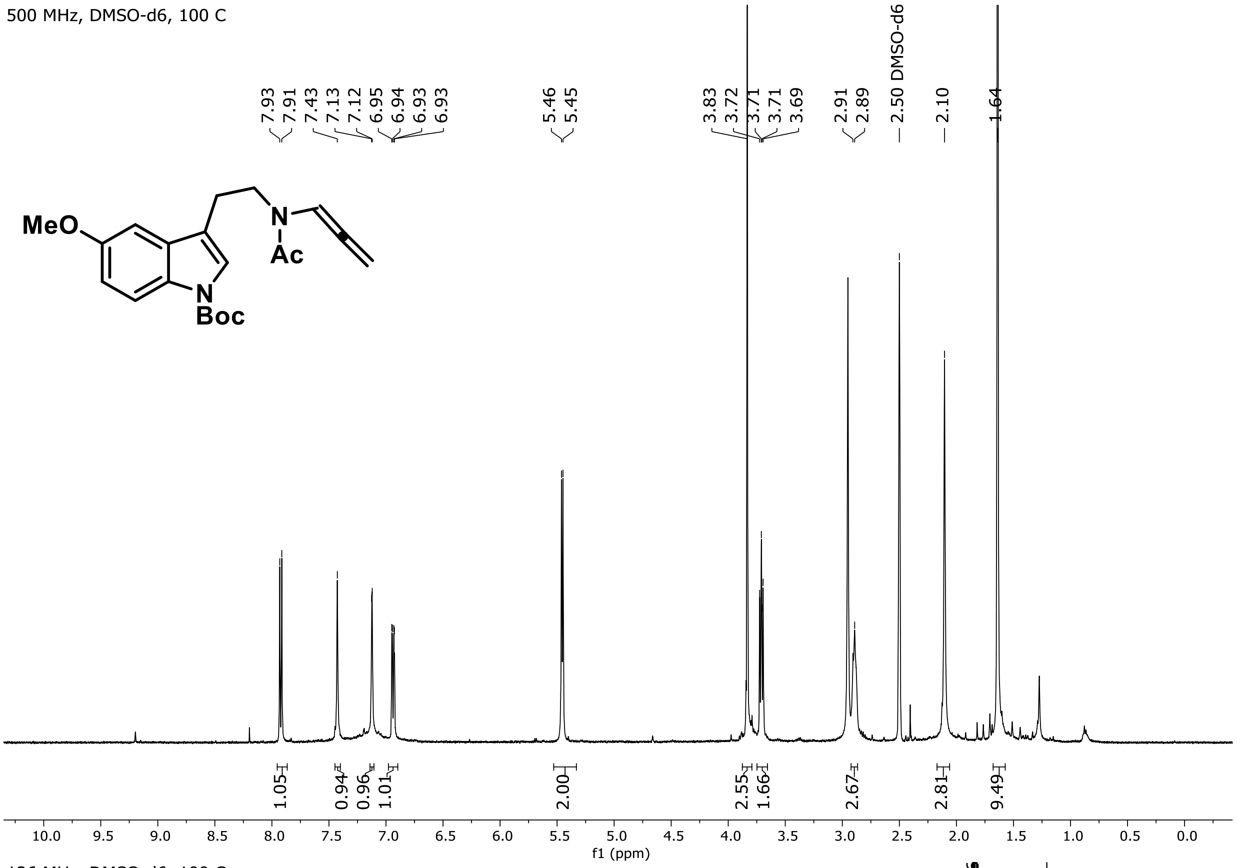

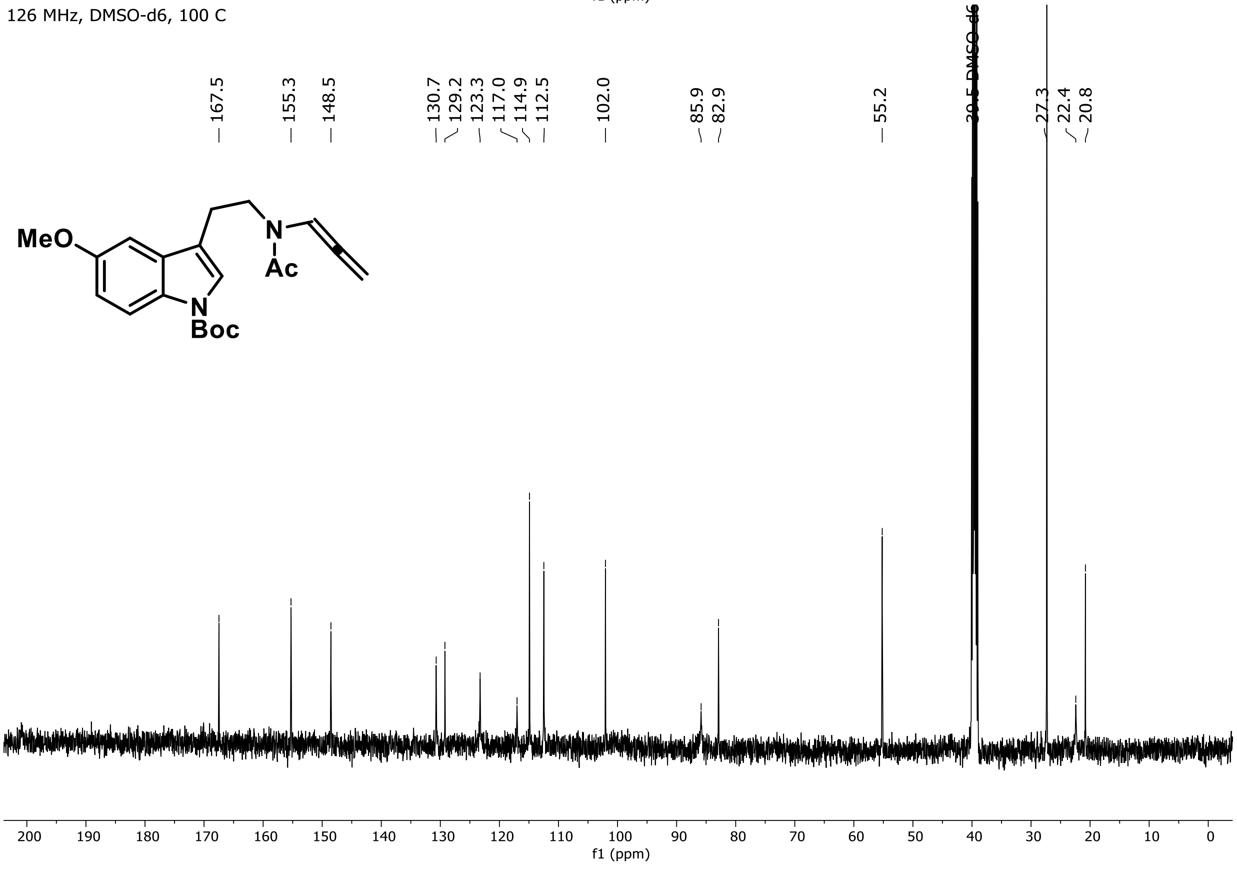

1-bromo-2-(propa-1,2-dien-1-yloxy)naphthalene **(2i)**

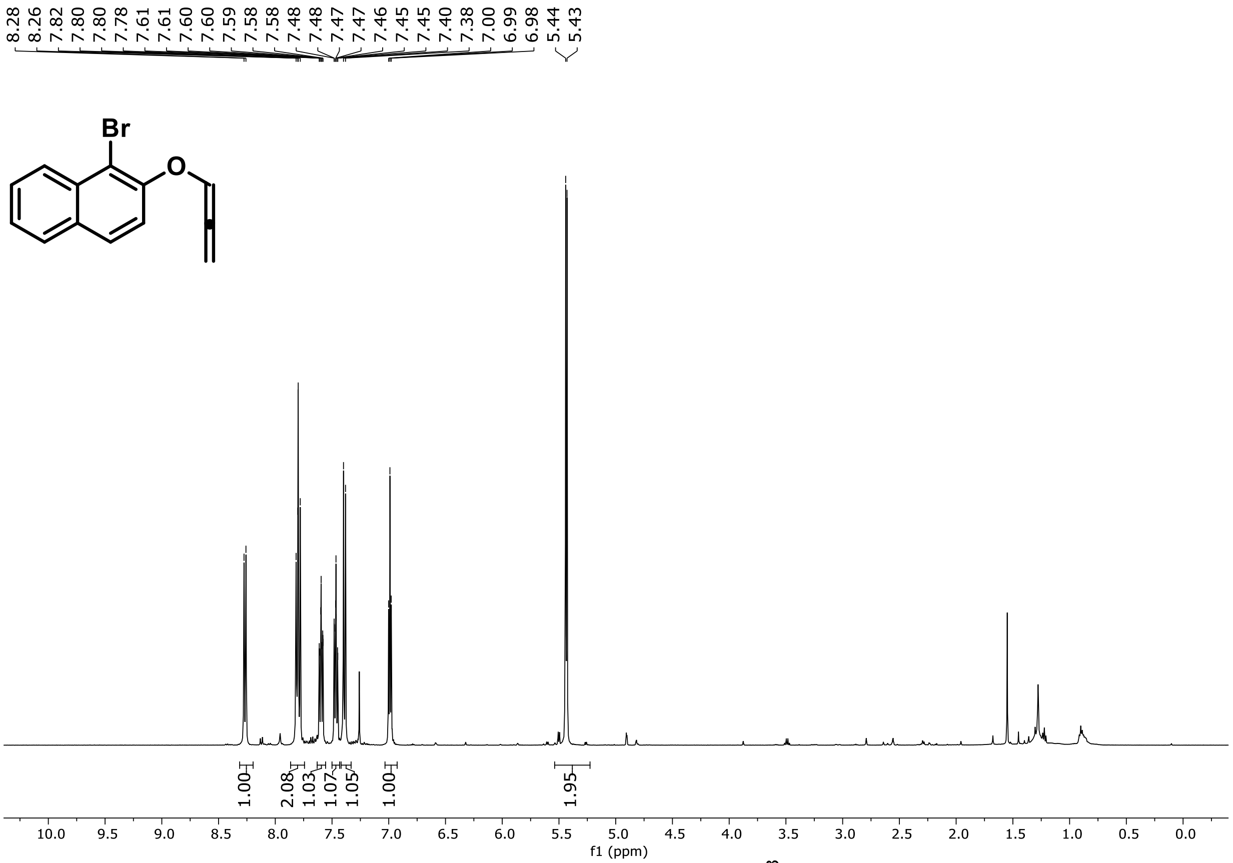

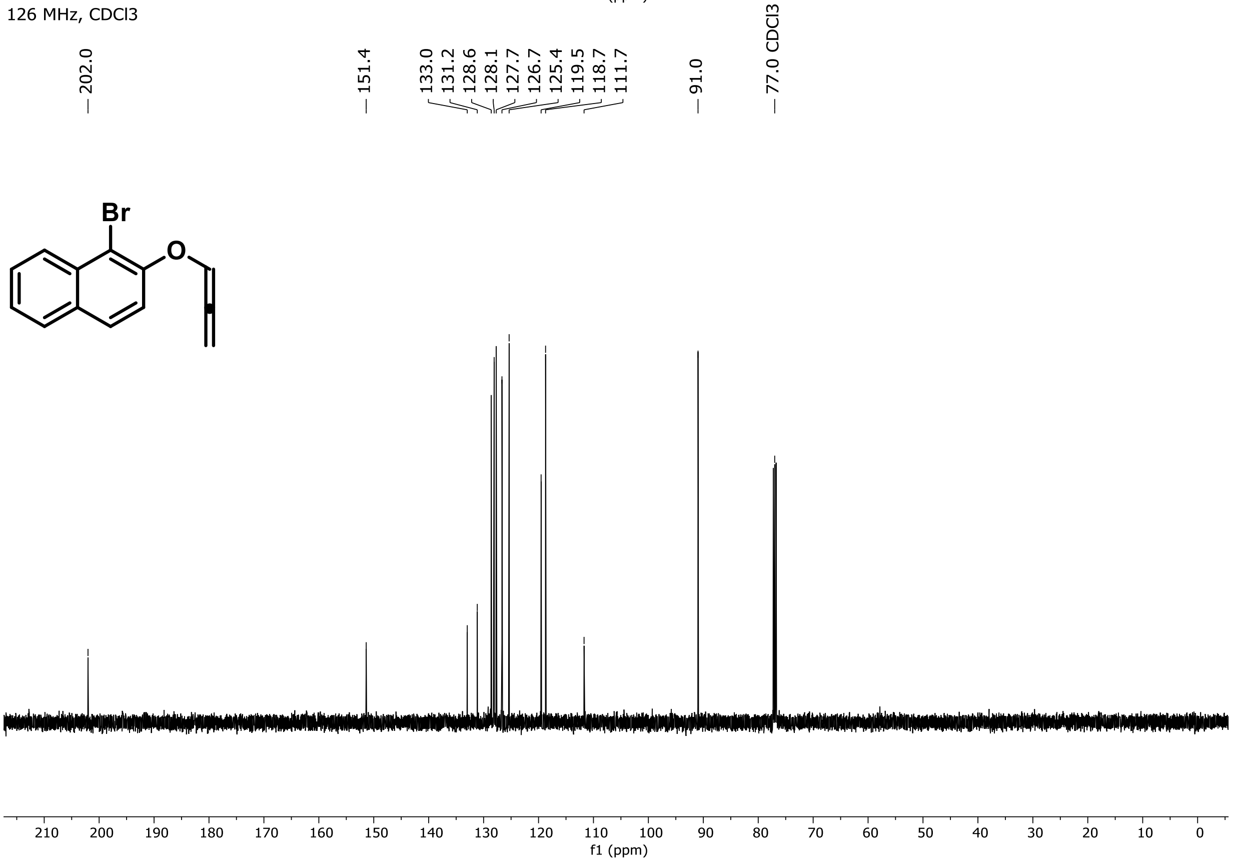

1-fluoro-4-(propa-1,2-dien-1-yloxy)benzene **(2j)**

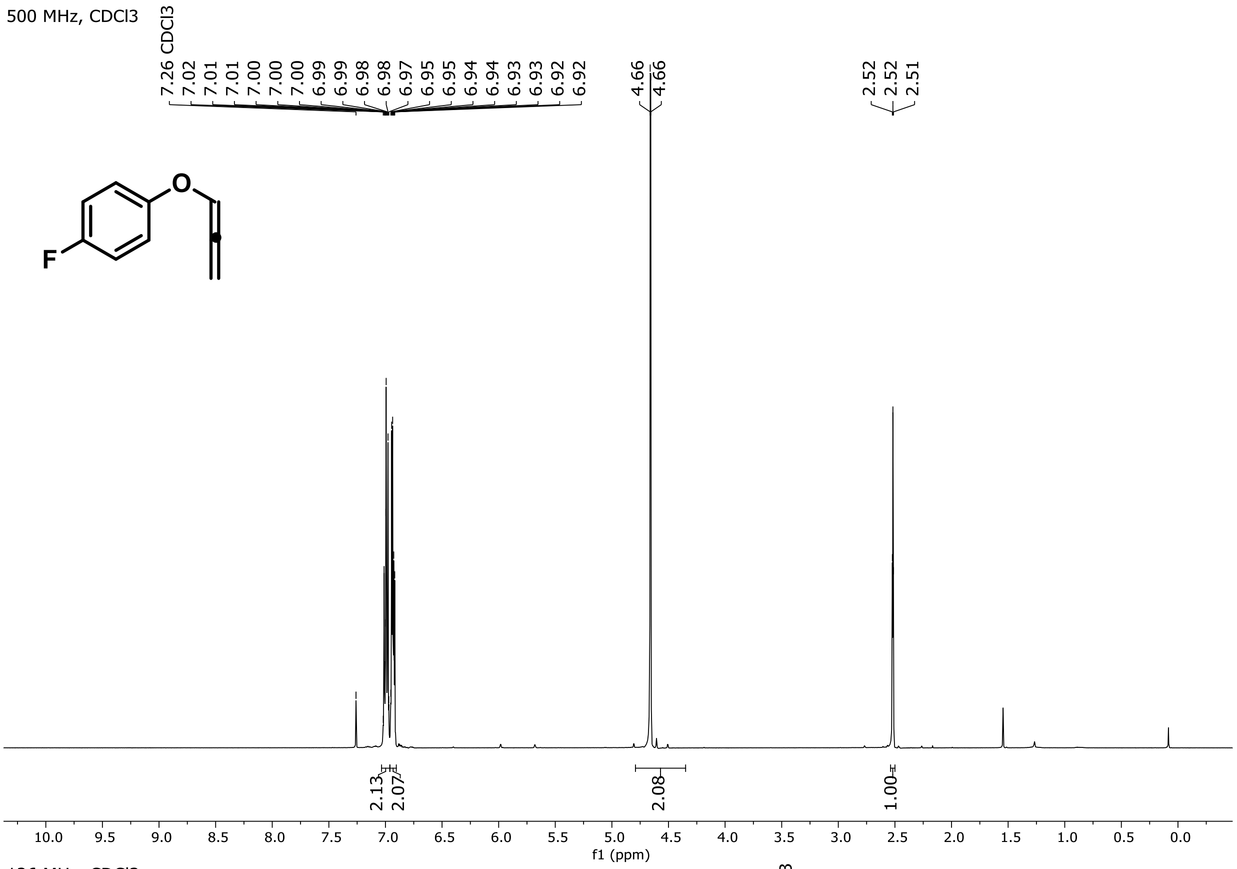

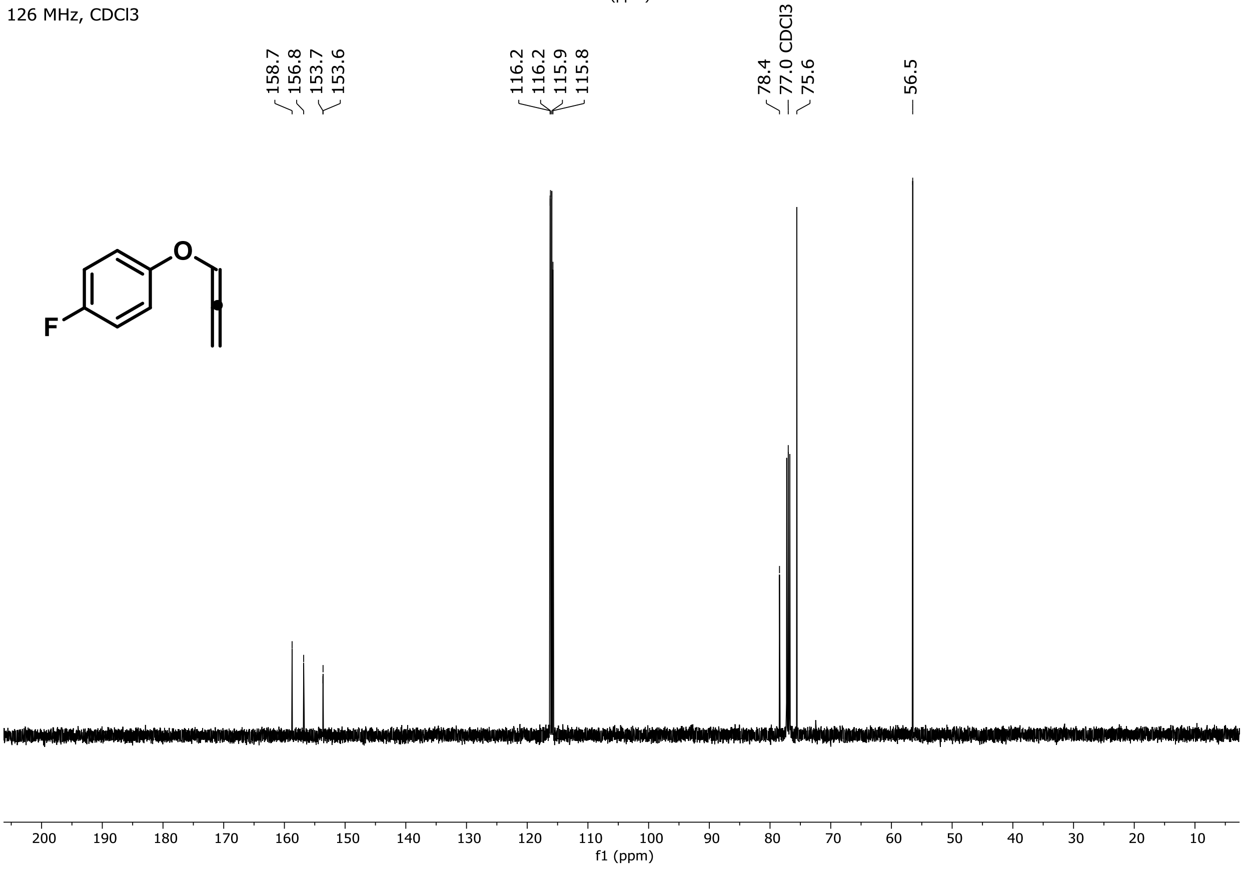

1-fluoro-3-(propa-1,2-dien-1-yloxy)benzene (**2k**)

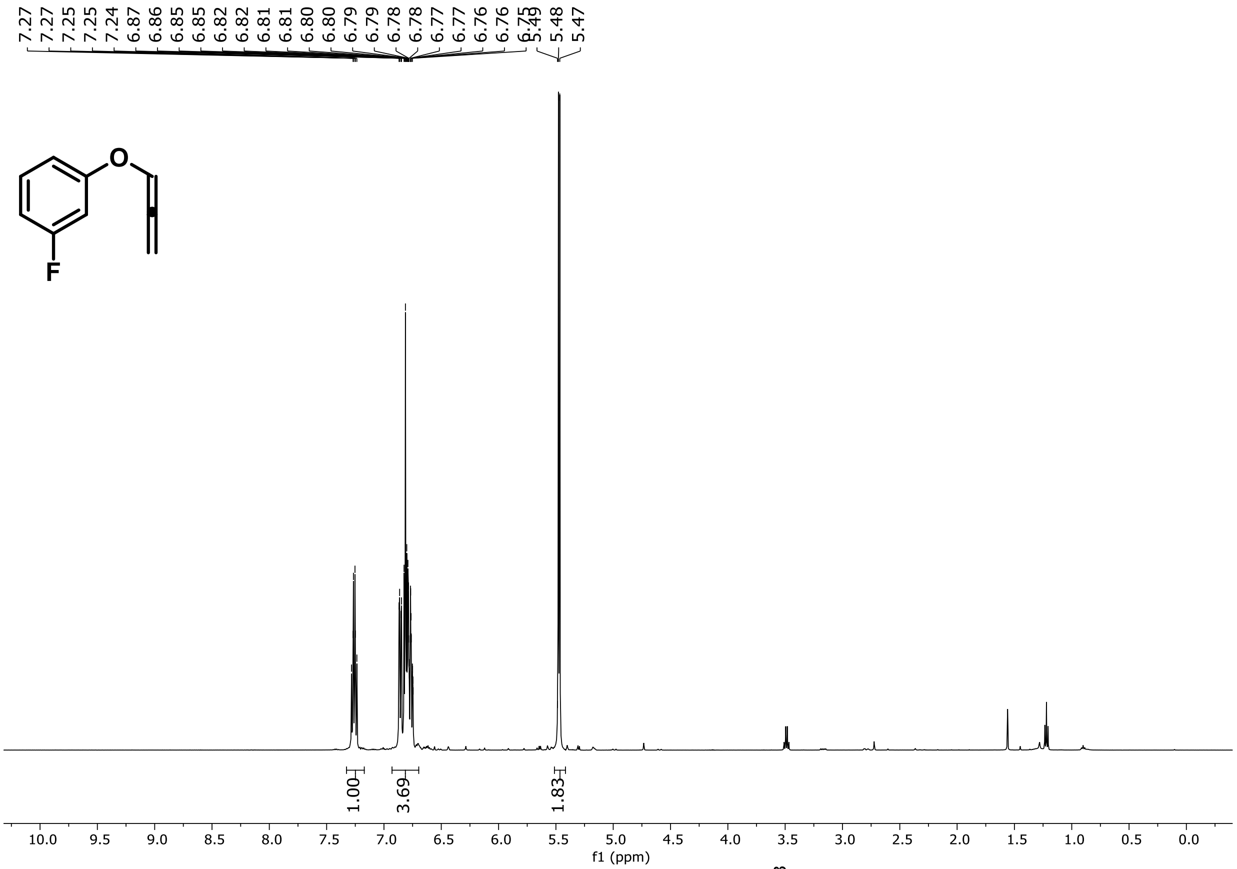

126 MHz, CDCl3

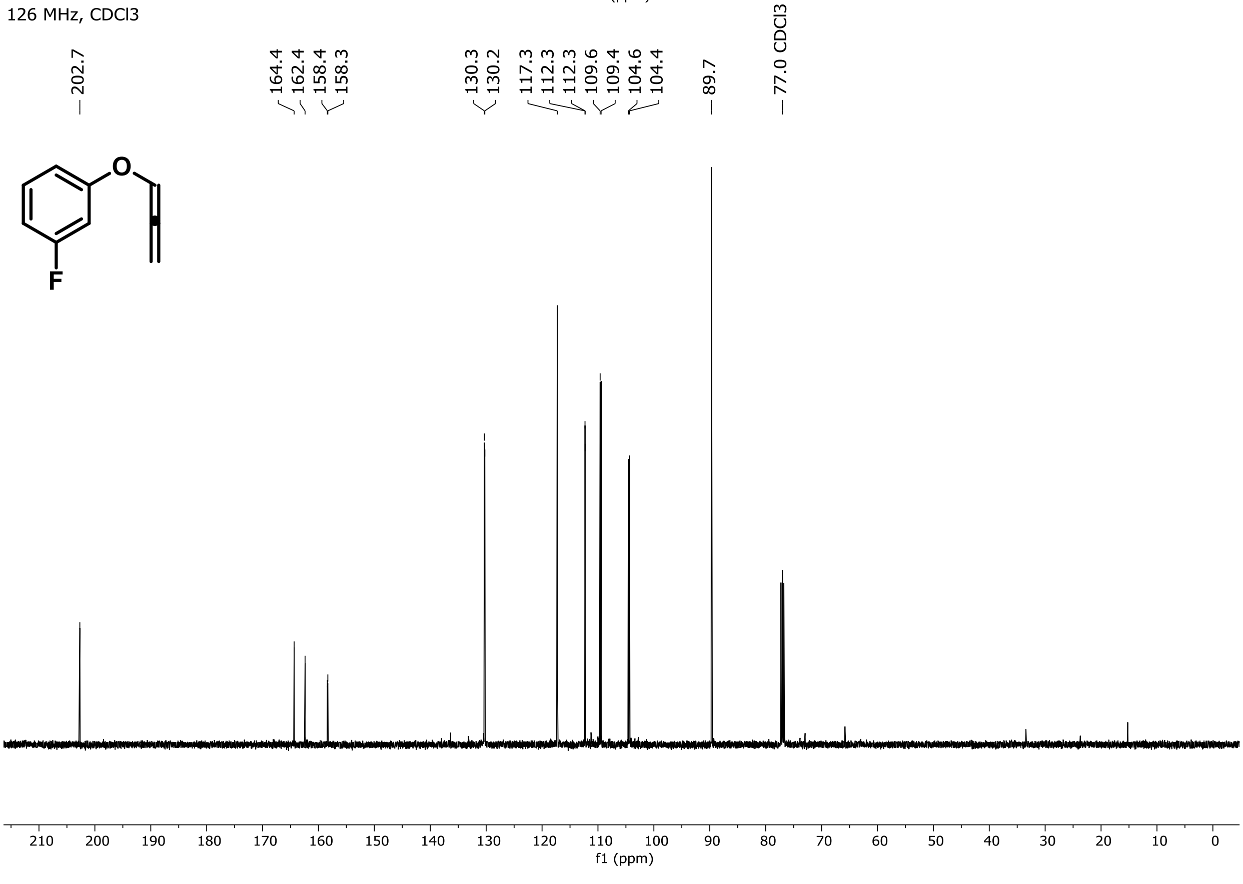

(*E*)-1,3-dimethoxy-5-(4-(propa-1,2-dien-1-yloxy)styryl)benzene (**2l**)

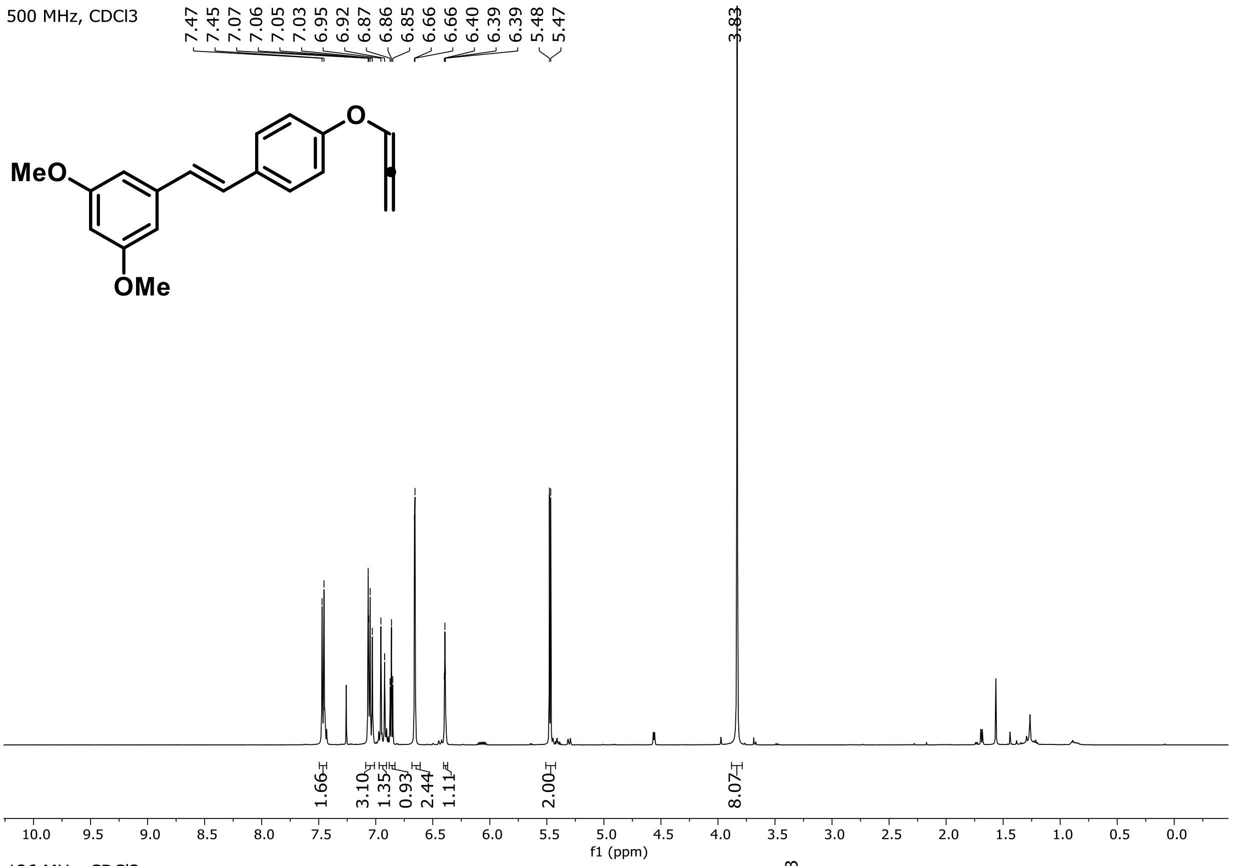

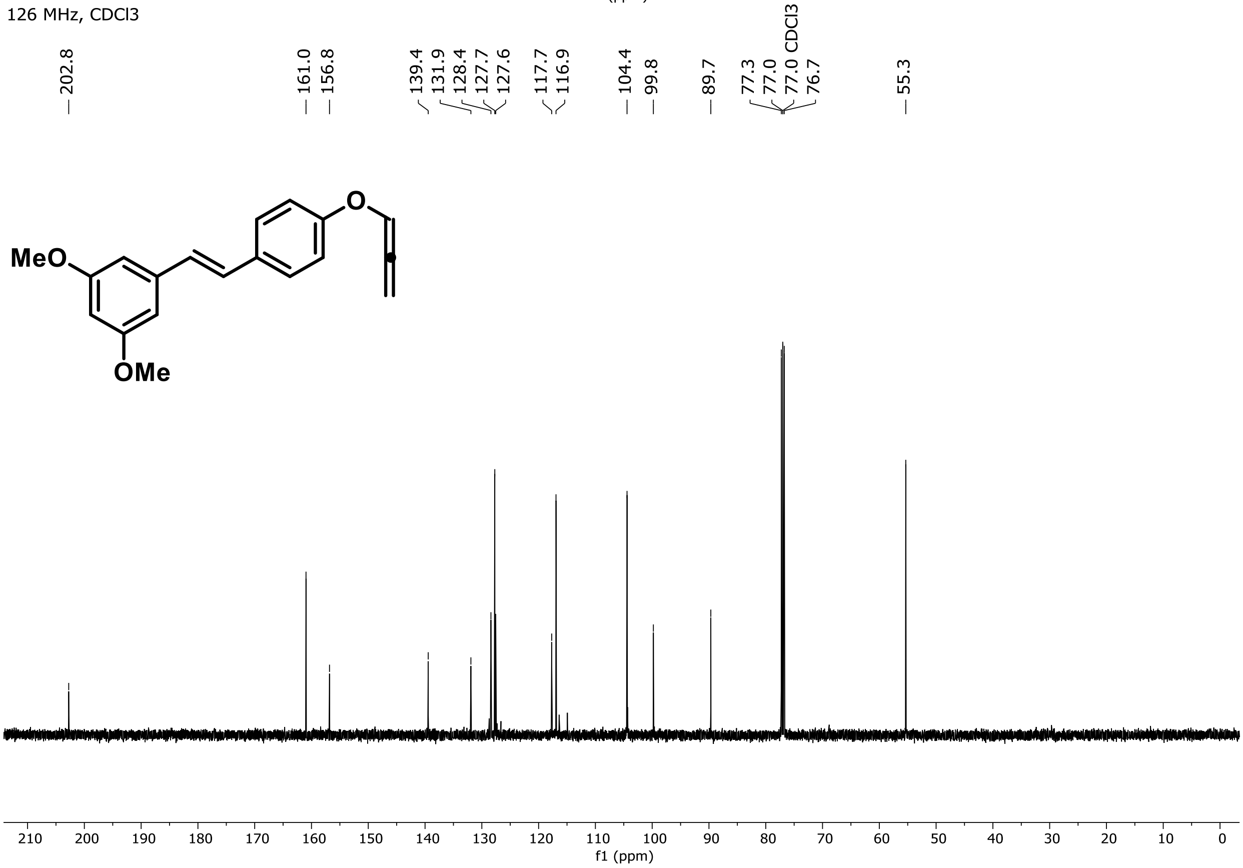

1-((prop-2-yn-1-yloxy)methyl)-4-(propa-1,2-dien-1-yloxy)benzene (**2t**) (NMR spectra with addition on TMB (trimethoxybenzen as internal standard)

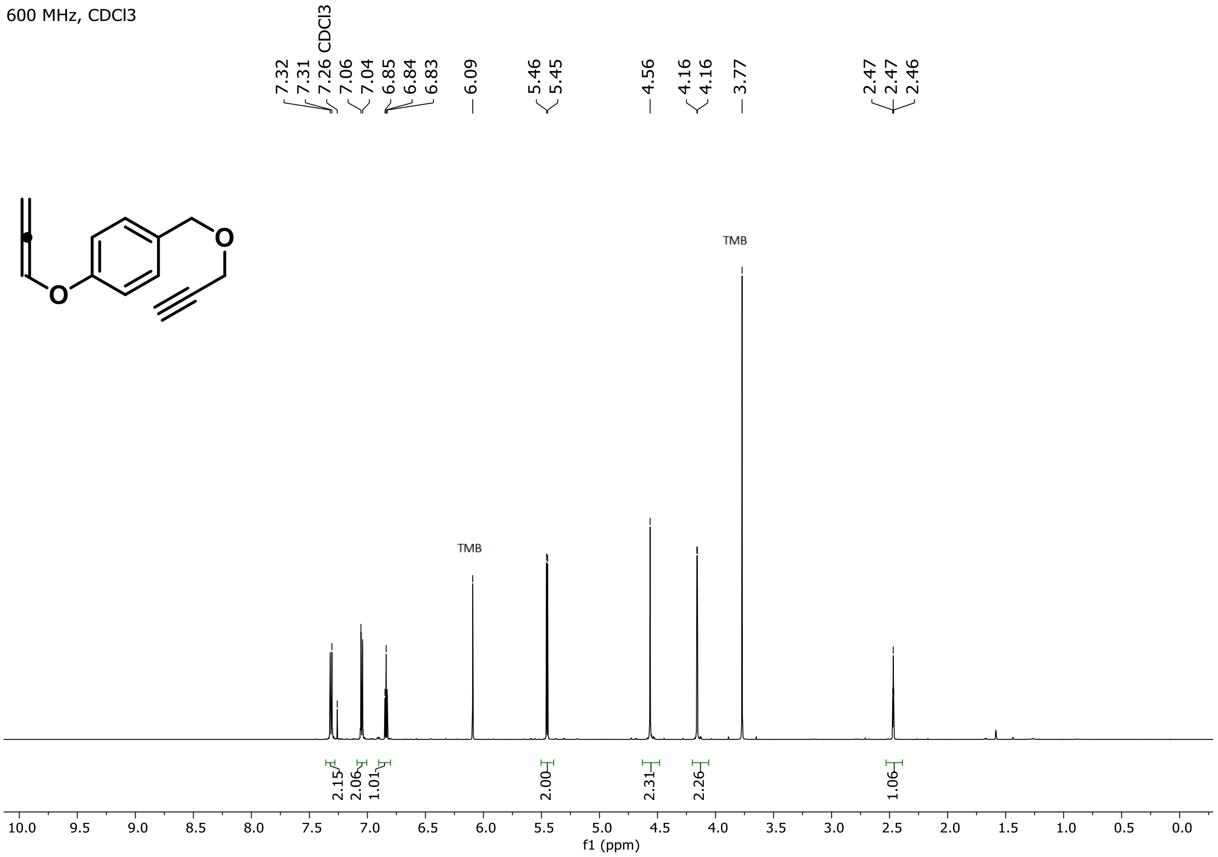

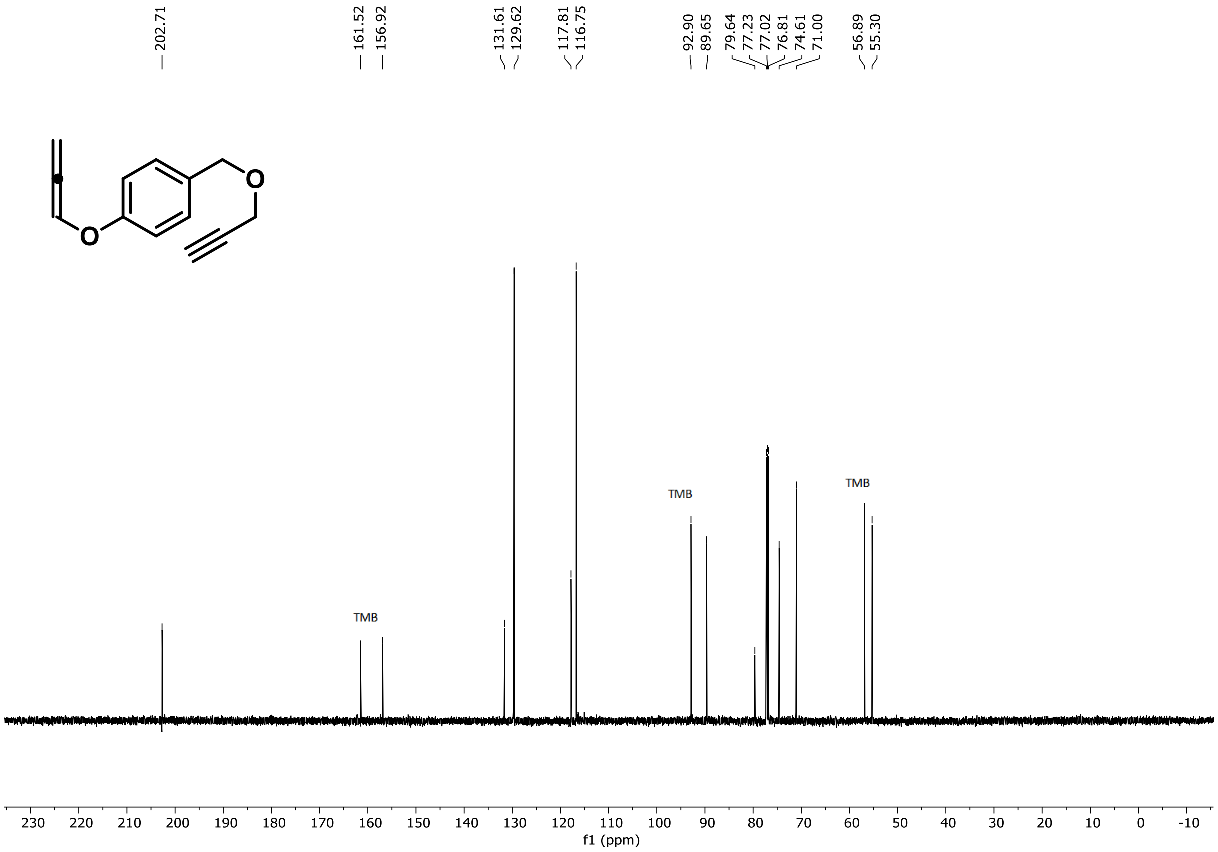

1-(propa-1,2-dien-1-yloxy)-4-((propa-1,2-dien-1-yloxy)methyl)benzene (**2tt**)

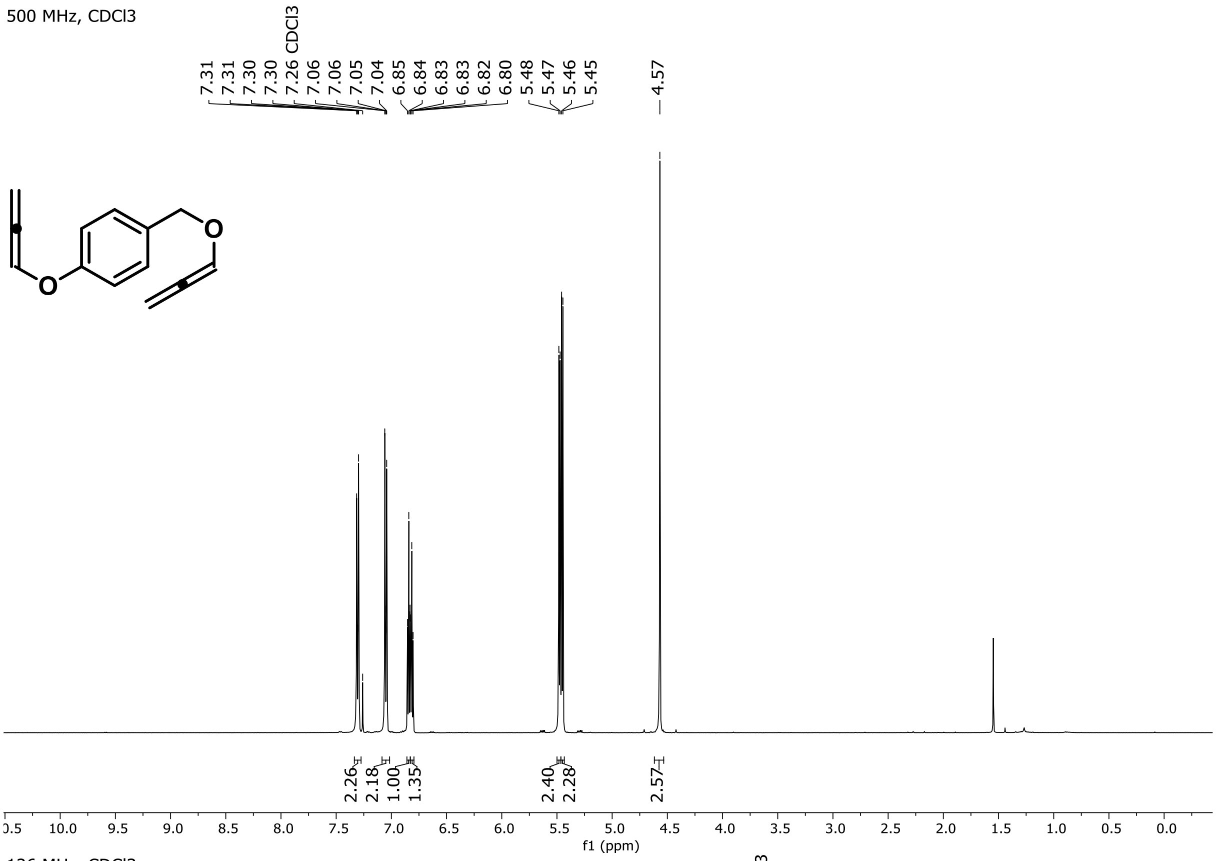

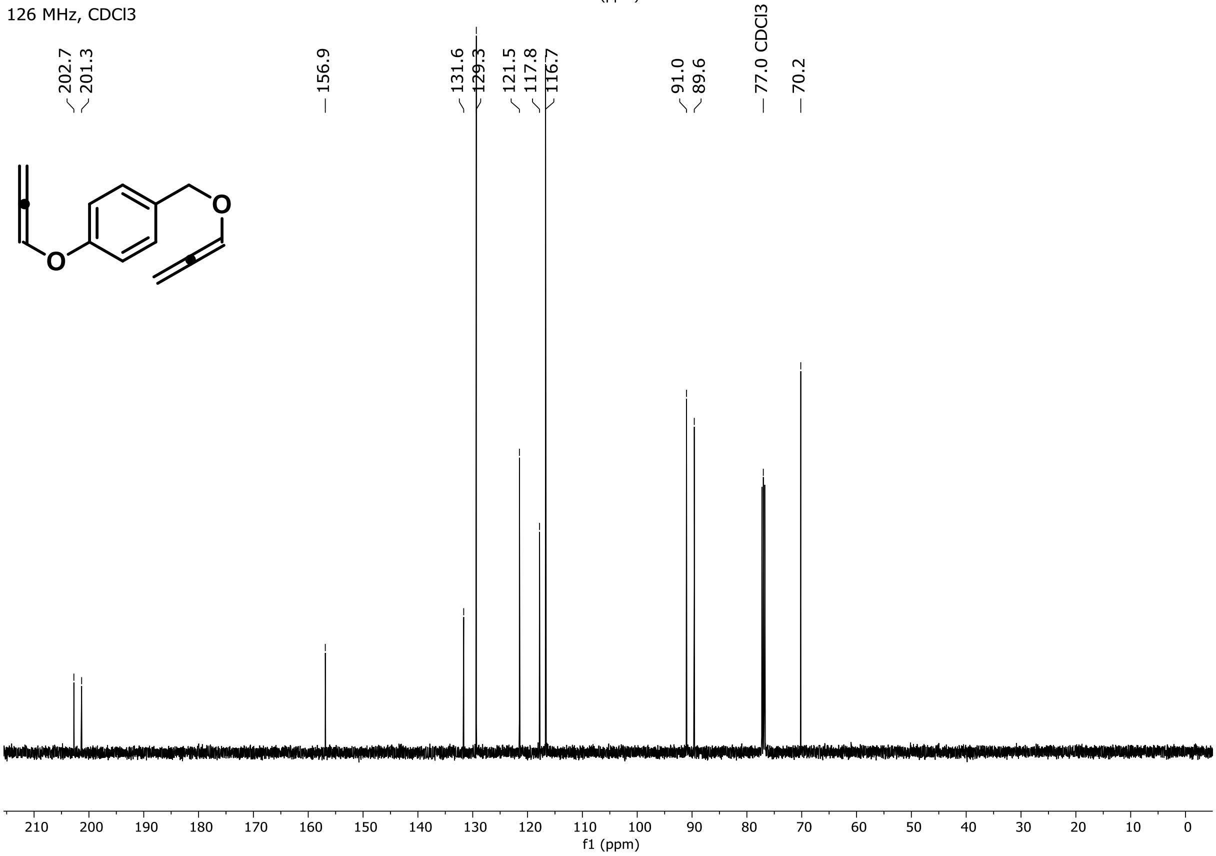

4-((prop-2-yn-1-yloxy)methyl)phenol (**3t**)

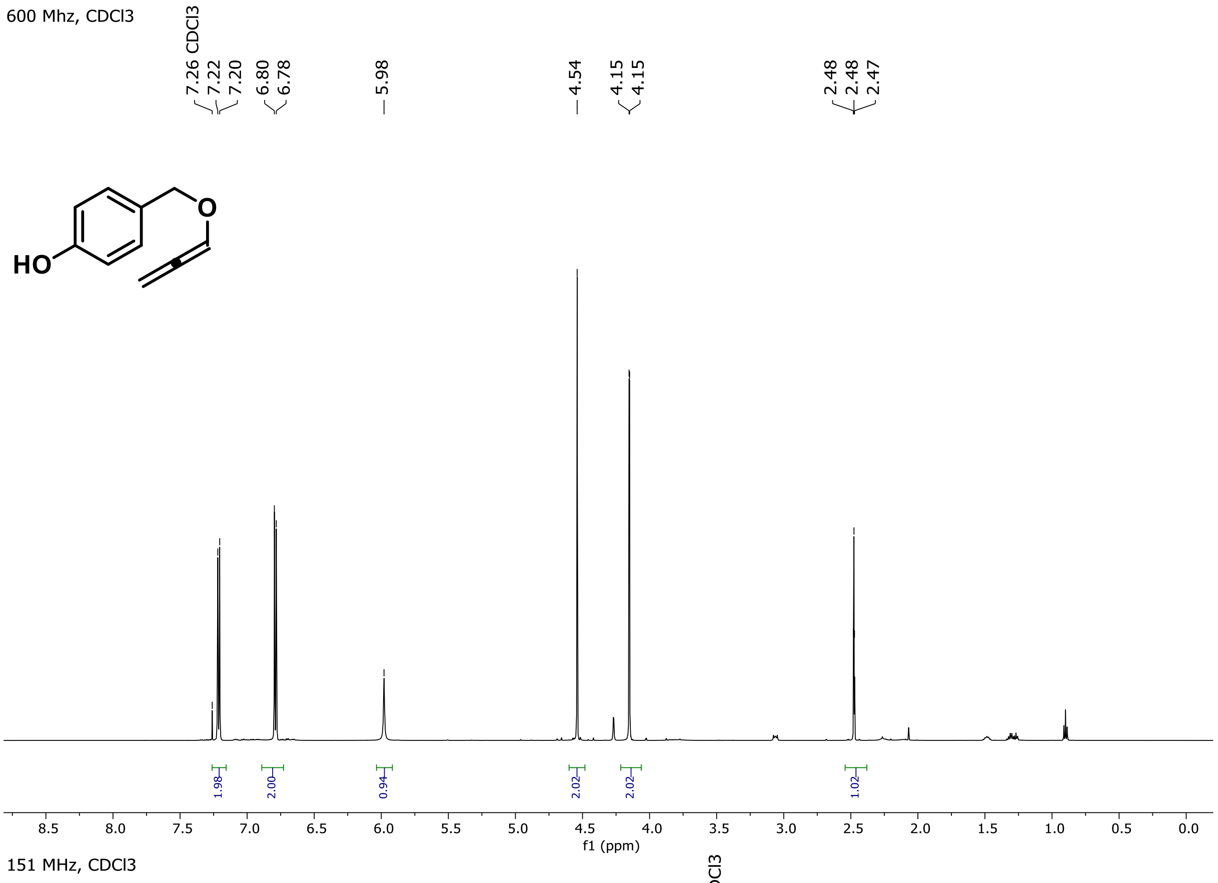

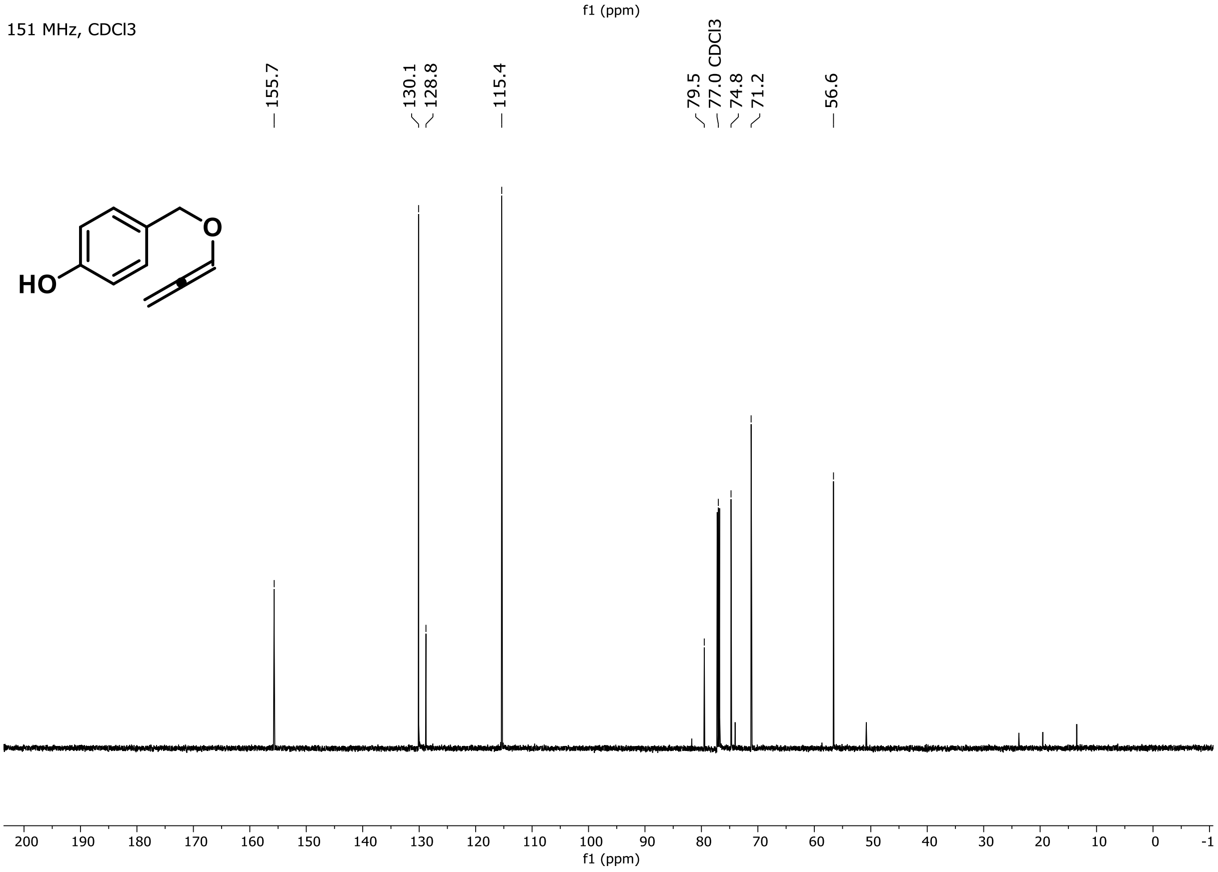

(E)-4-(3,5-dimethoxystyryl)phenol, pterostilbene (**3l**)

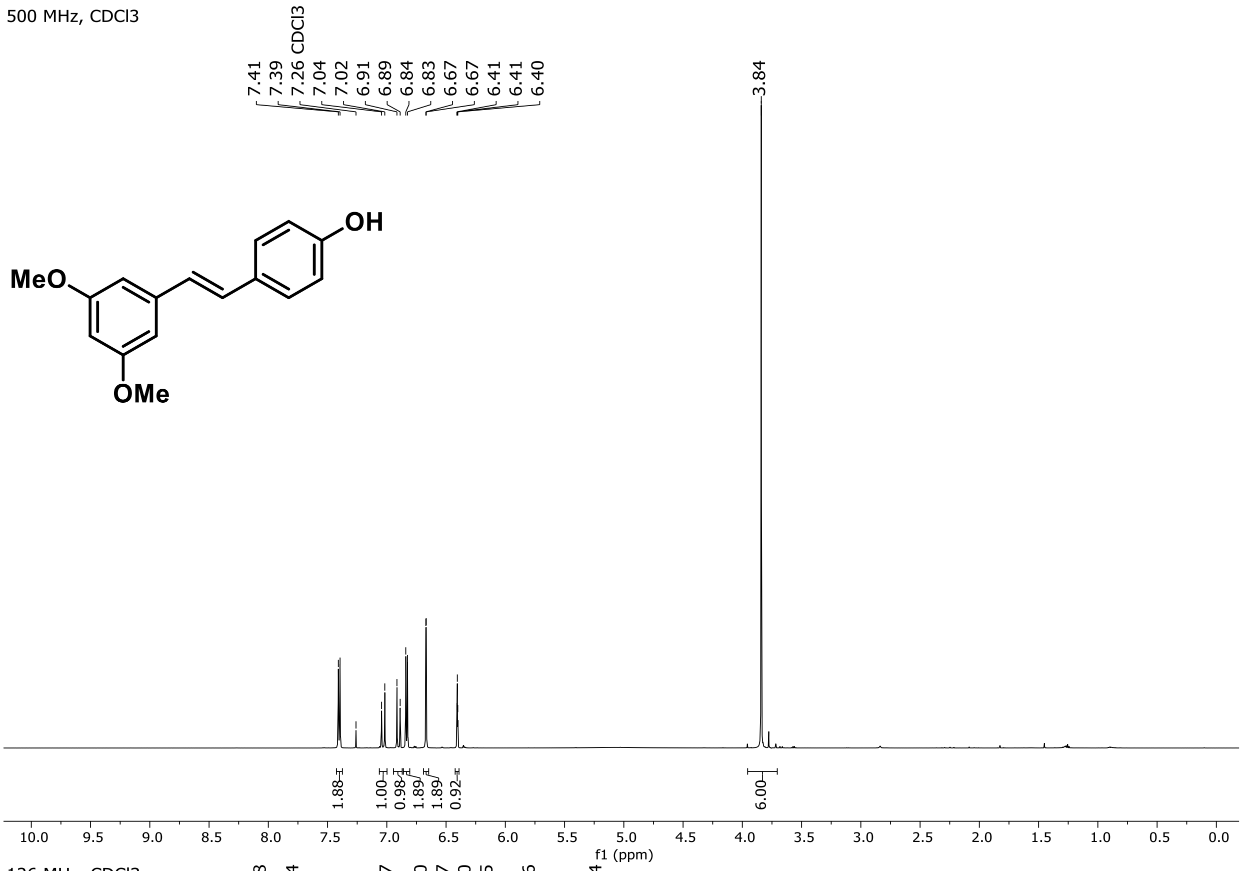

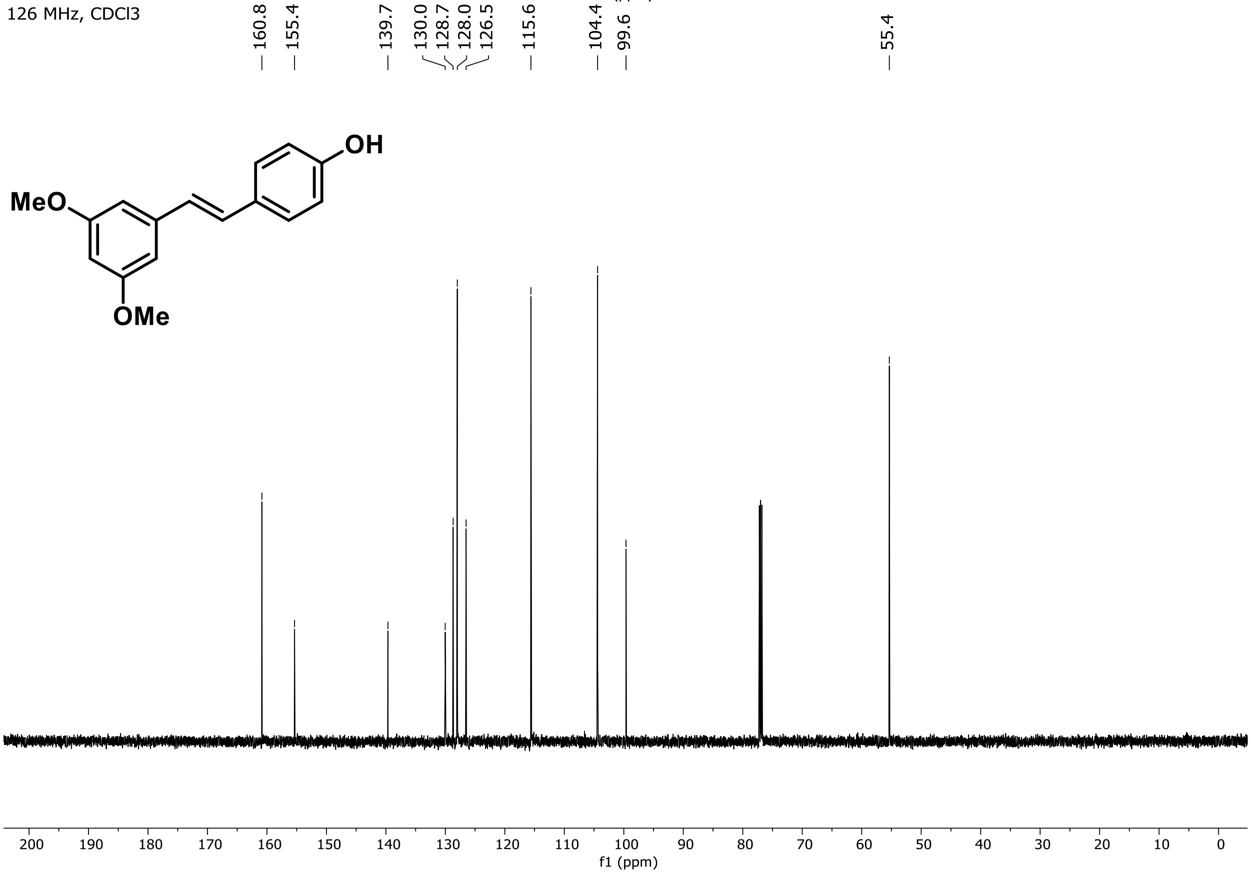

N-(4-(prop-2-yn-1-yloxy)phenyl)-*N*-(propa-1,2-dien-1-yl)acetamide (**2u**)

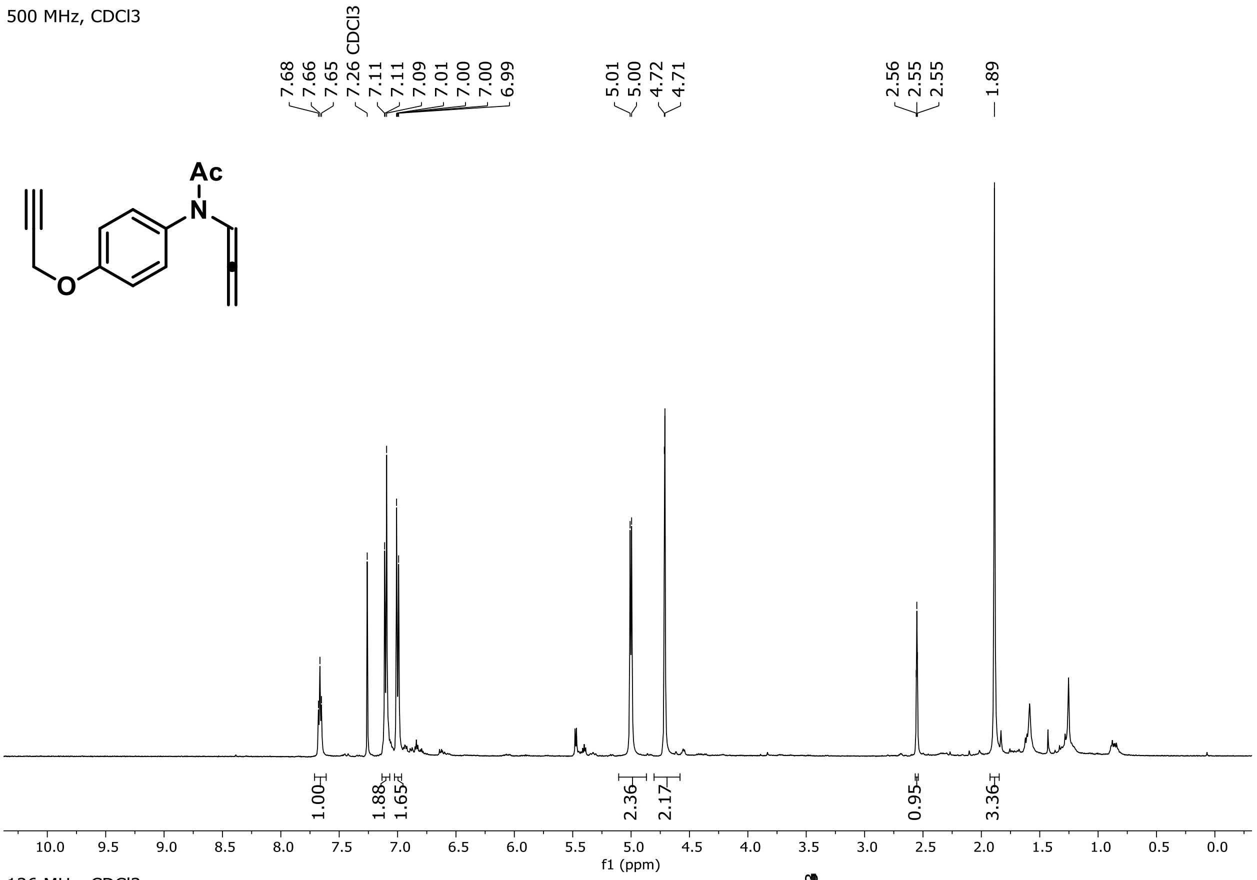

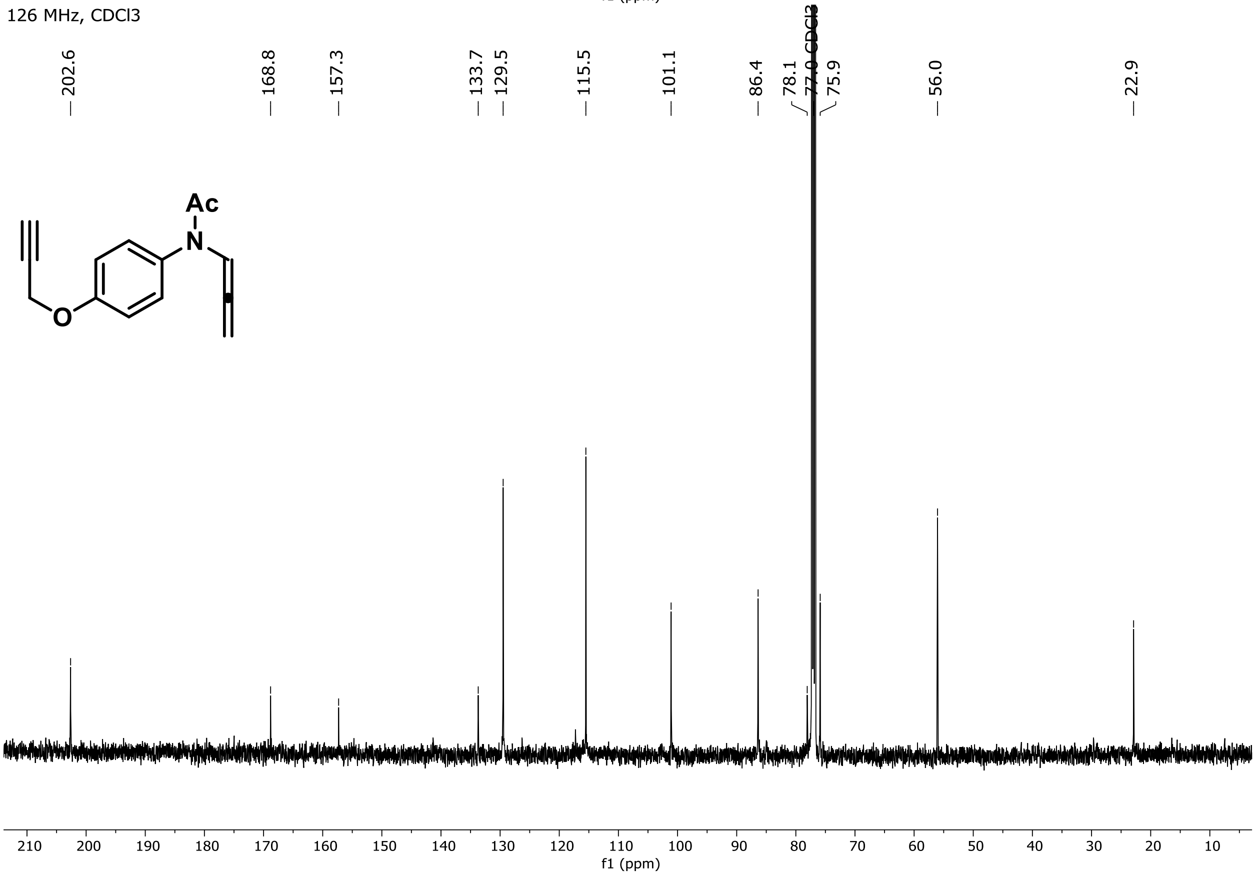

N-(propa-1,2-dien-1-yl)-*N*-(4-(propa-1,2-dien-1-yloxy)phenyl)acetamide (**2uu**)

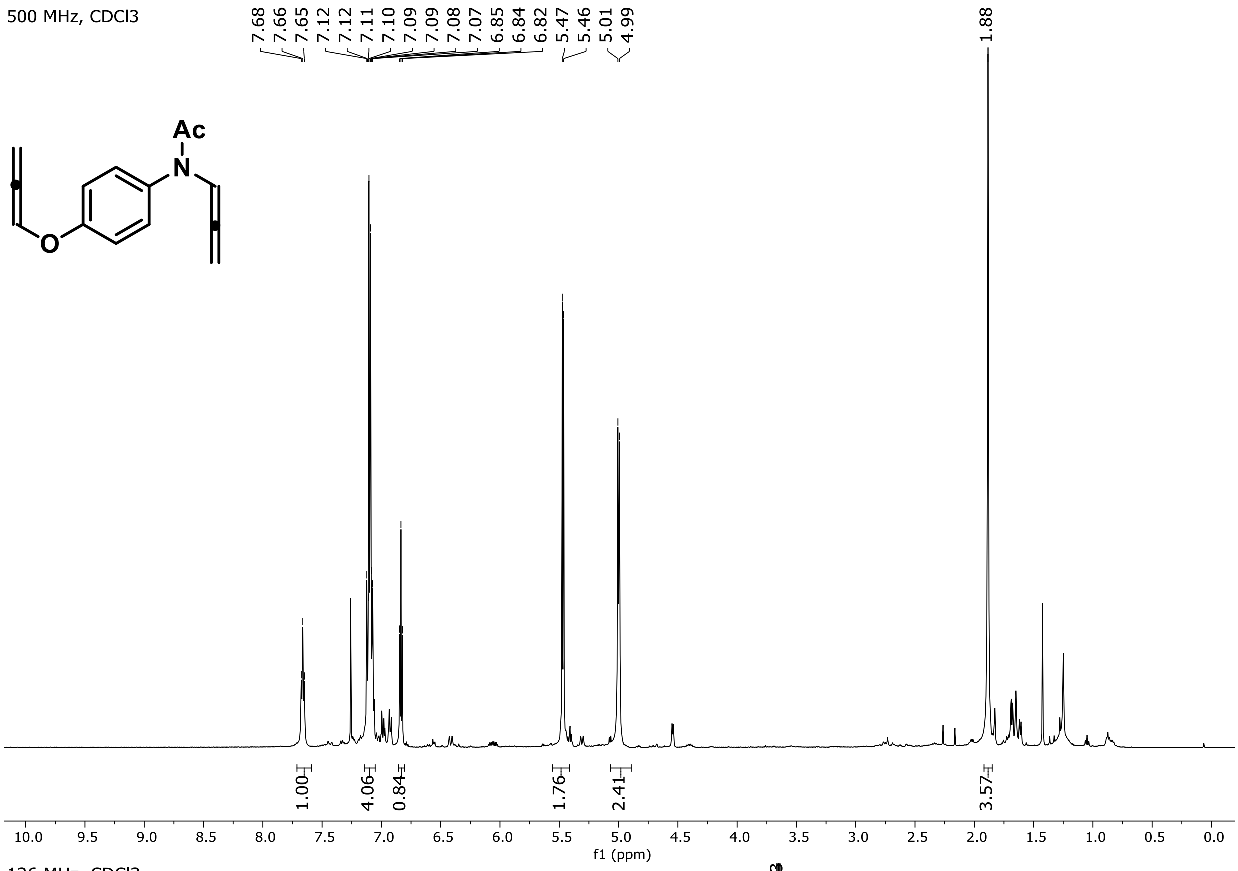

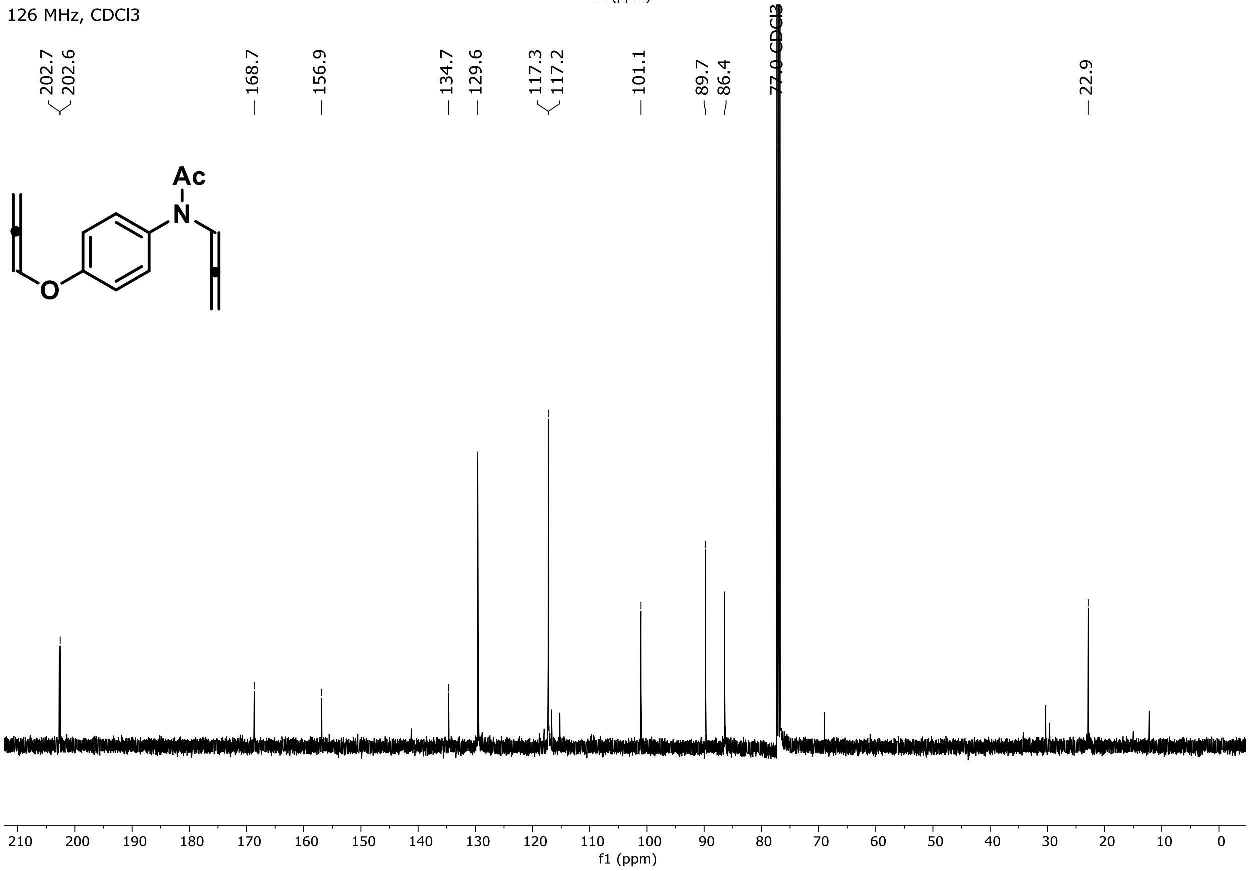

N-(4-hydroxyphenyl)-*N*-(propa-1,2-dien-1-yl)acetamide (**3u**)

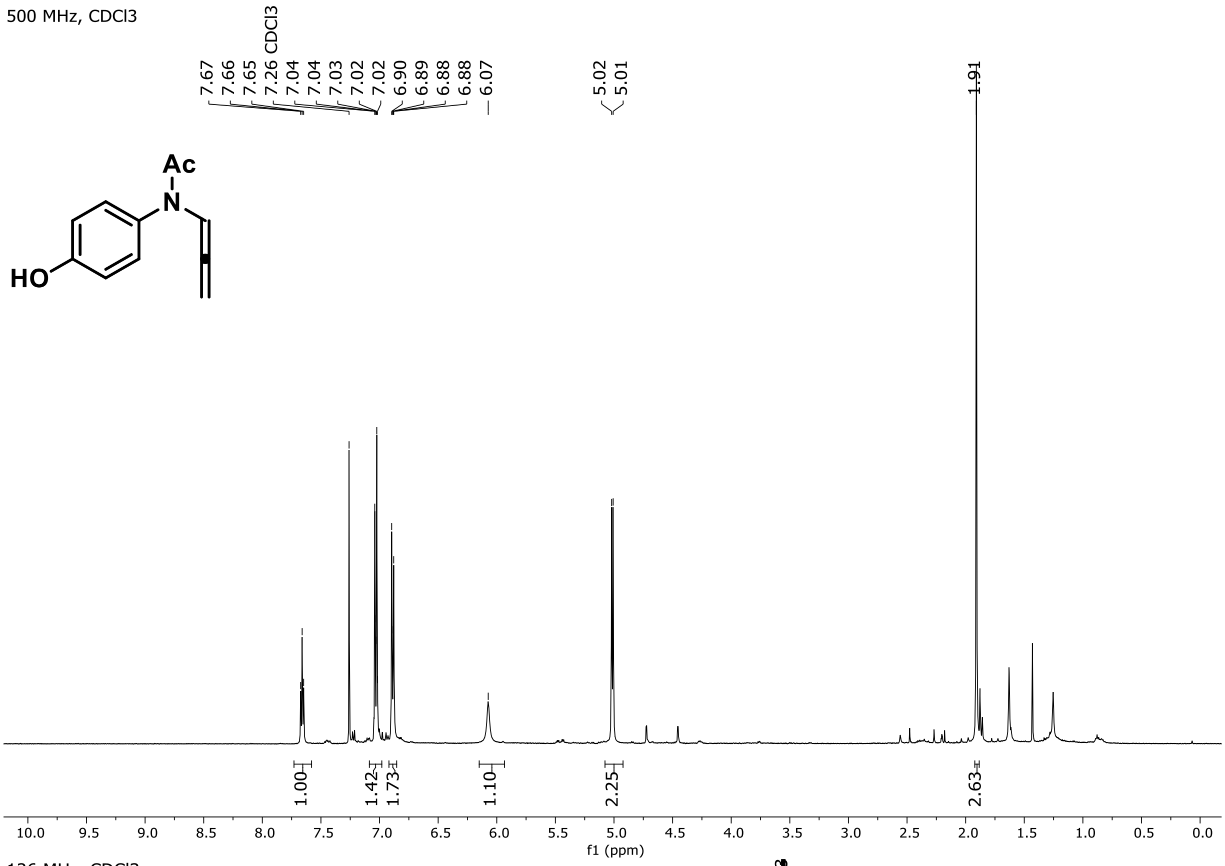

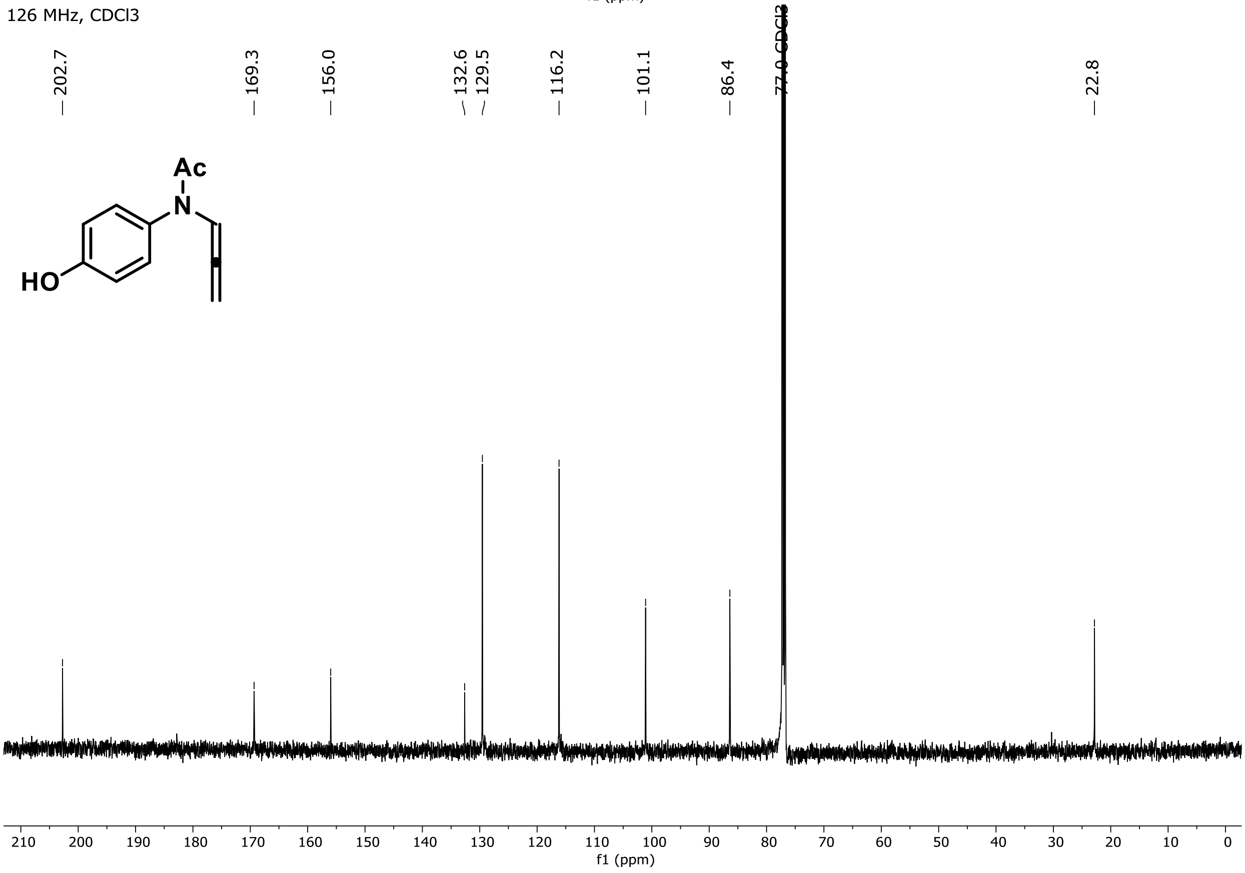

N-(3-(prop-2-yn-1-yloxy)phenyl)-*N*-(propa-1,2-dien-1-yl)acetamide (**2w**)

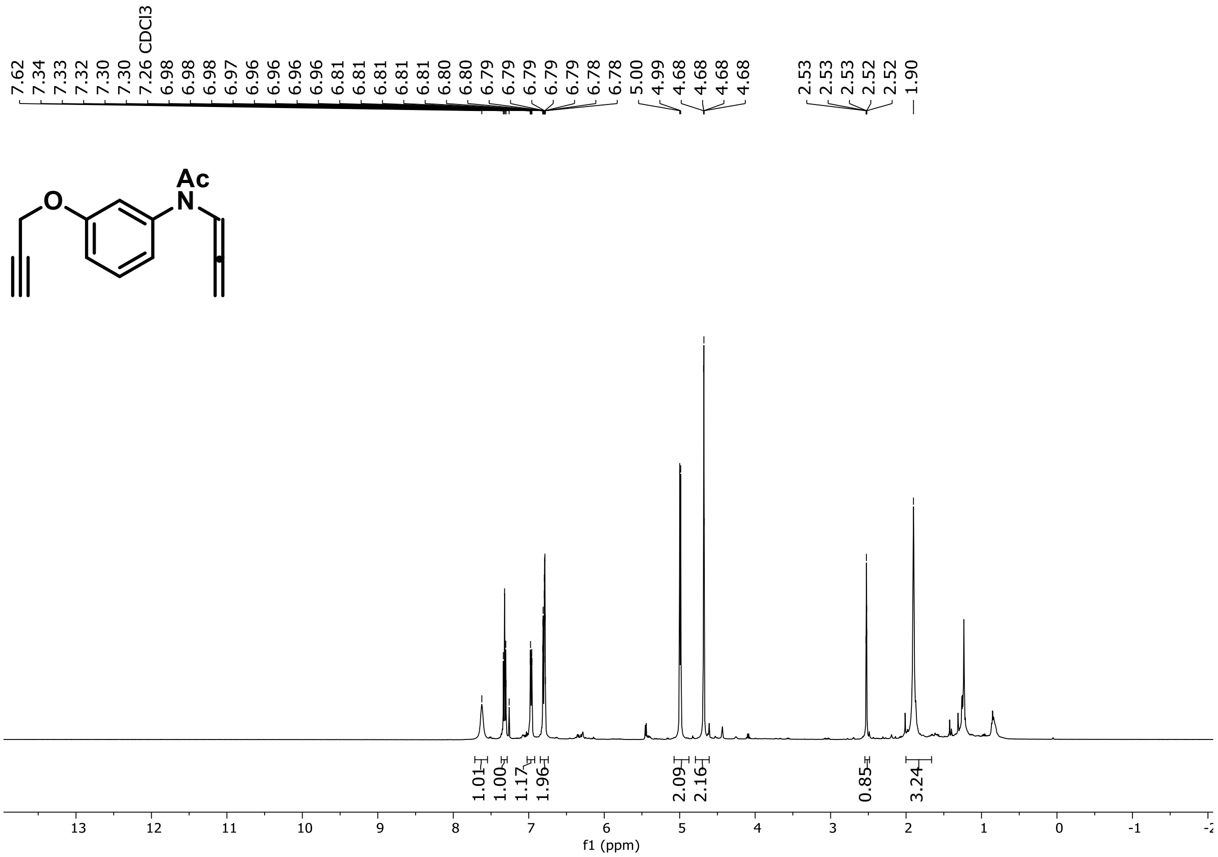

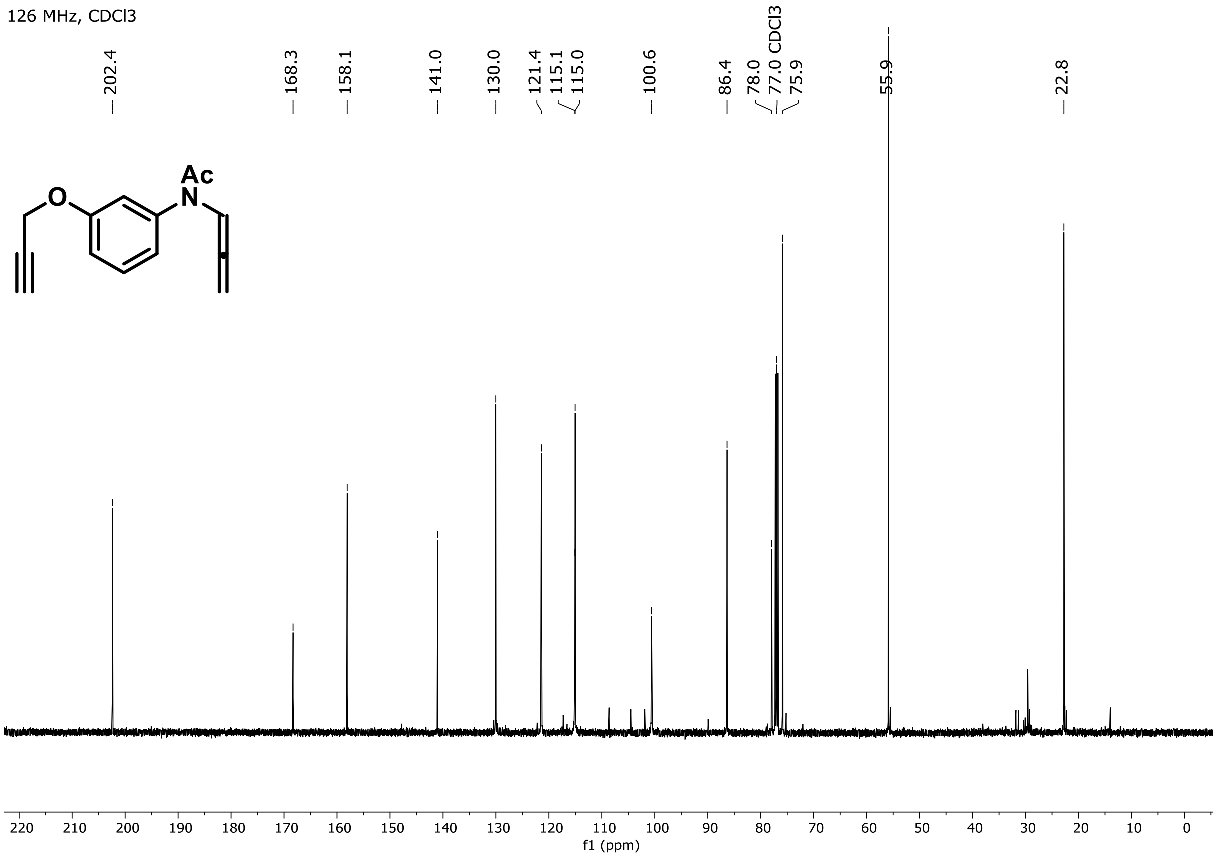

N-(propa-1,2-dien-1-yl)-*N*-(3-(propa-1,2-dien-1-yloxy)phenyl)acetamide (**2ww**)

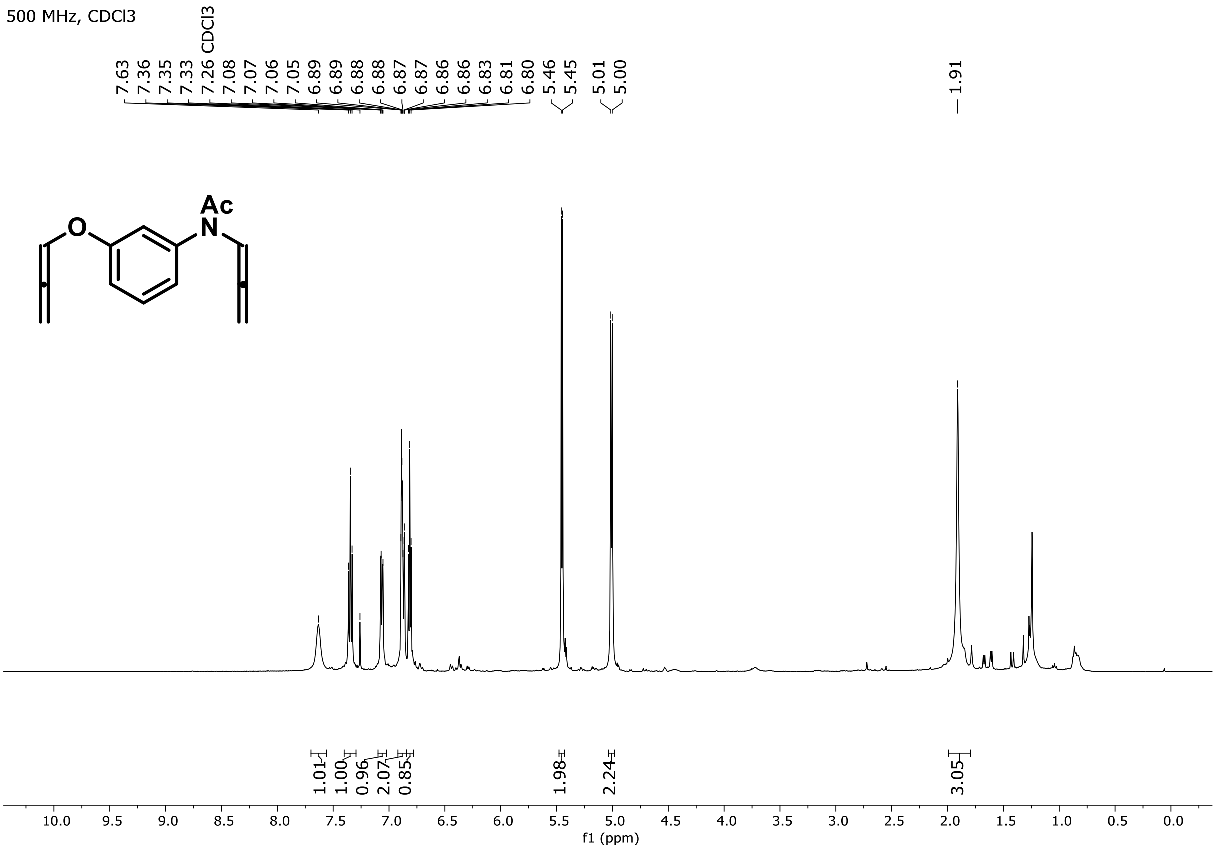

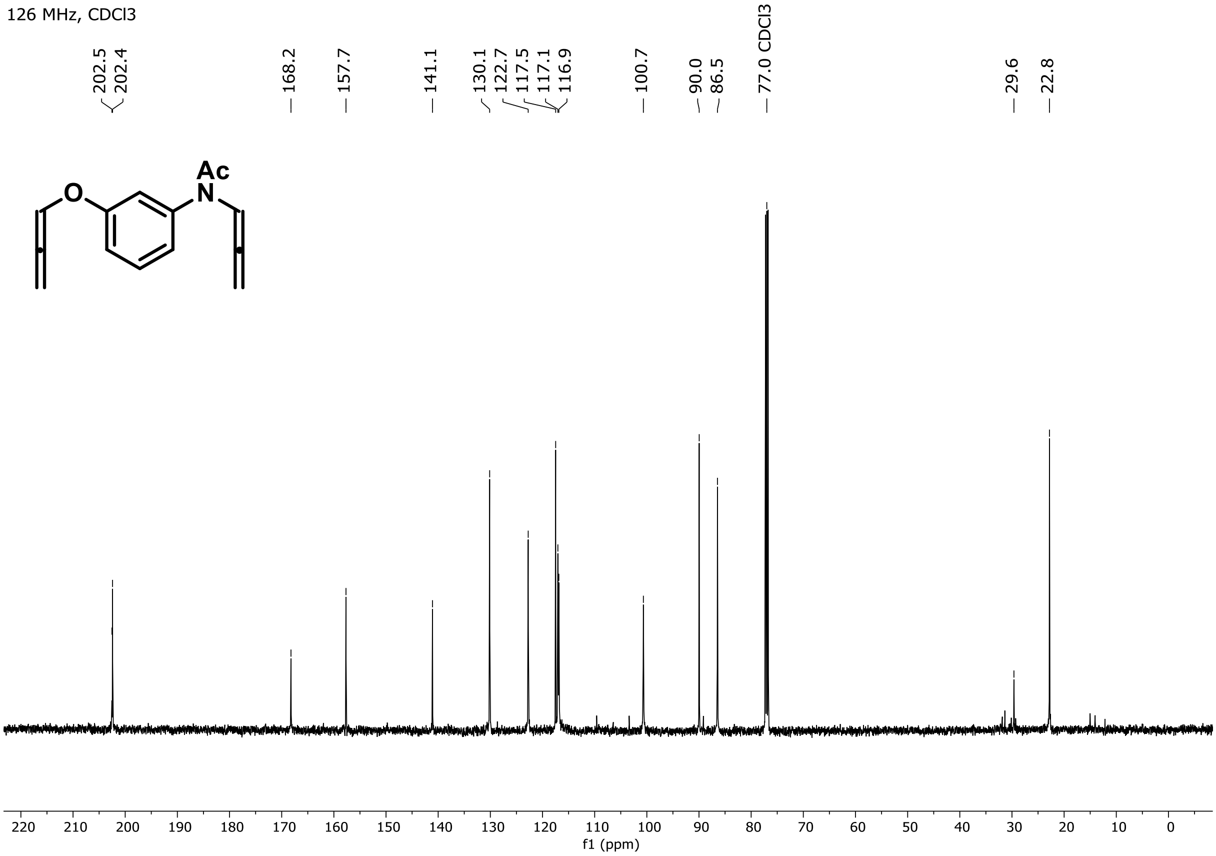

N-(3-hydroxyphenyl)-*N*-(propa-1,2-dien-1-yl)acetamide (**3w**)

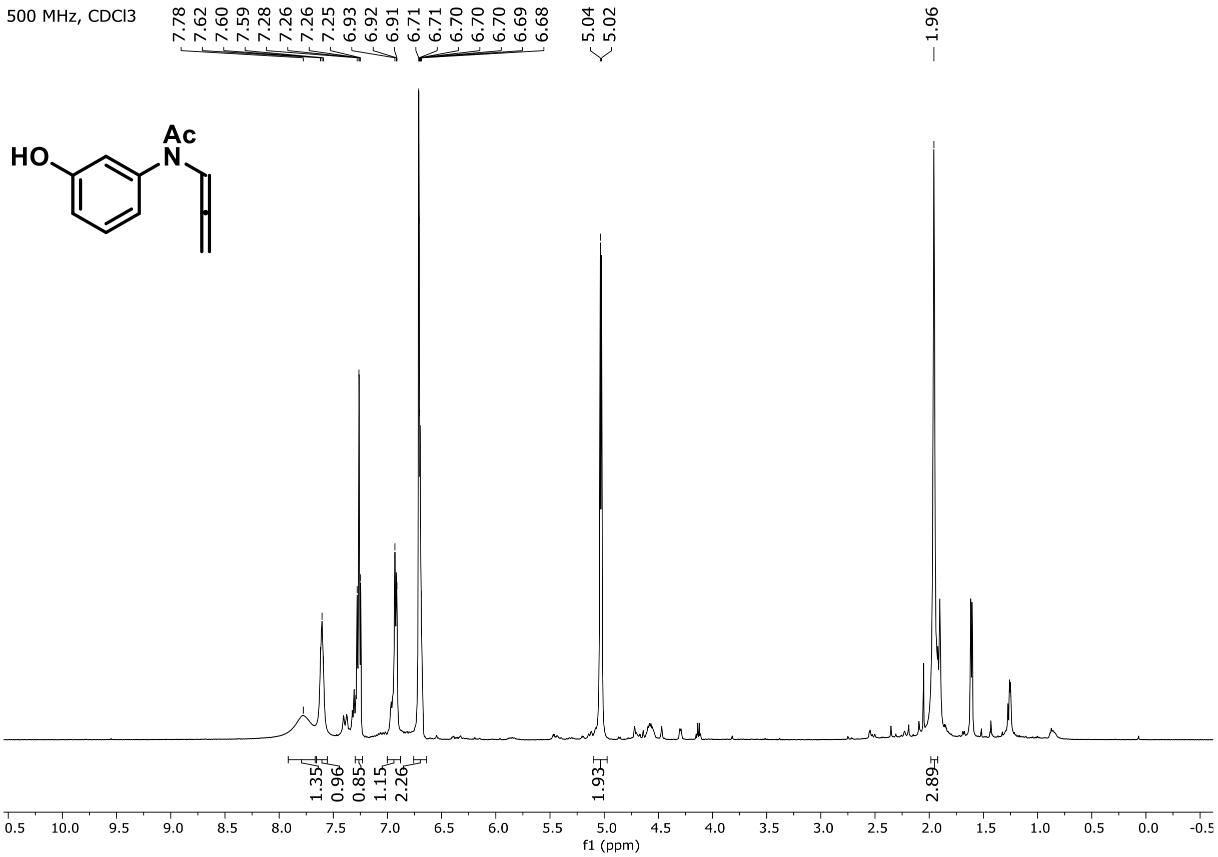

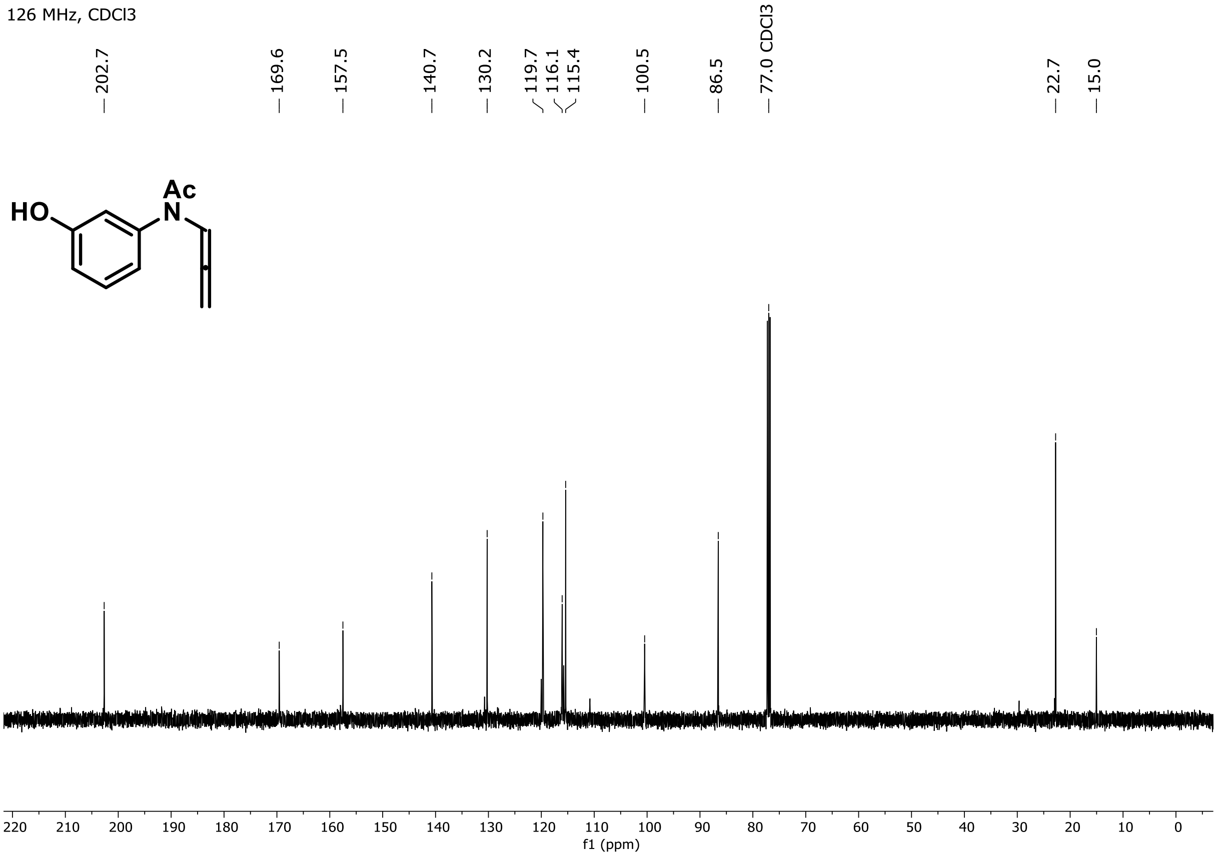

4-methyl-*N*-(4-(prop-2-yn-1-yloxy)phenyl)-*N*-(propa-1,2-dien-1-yl)benzenesulfonamide (**2x**)

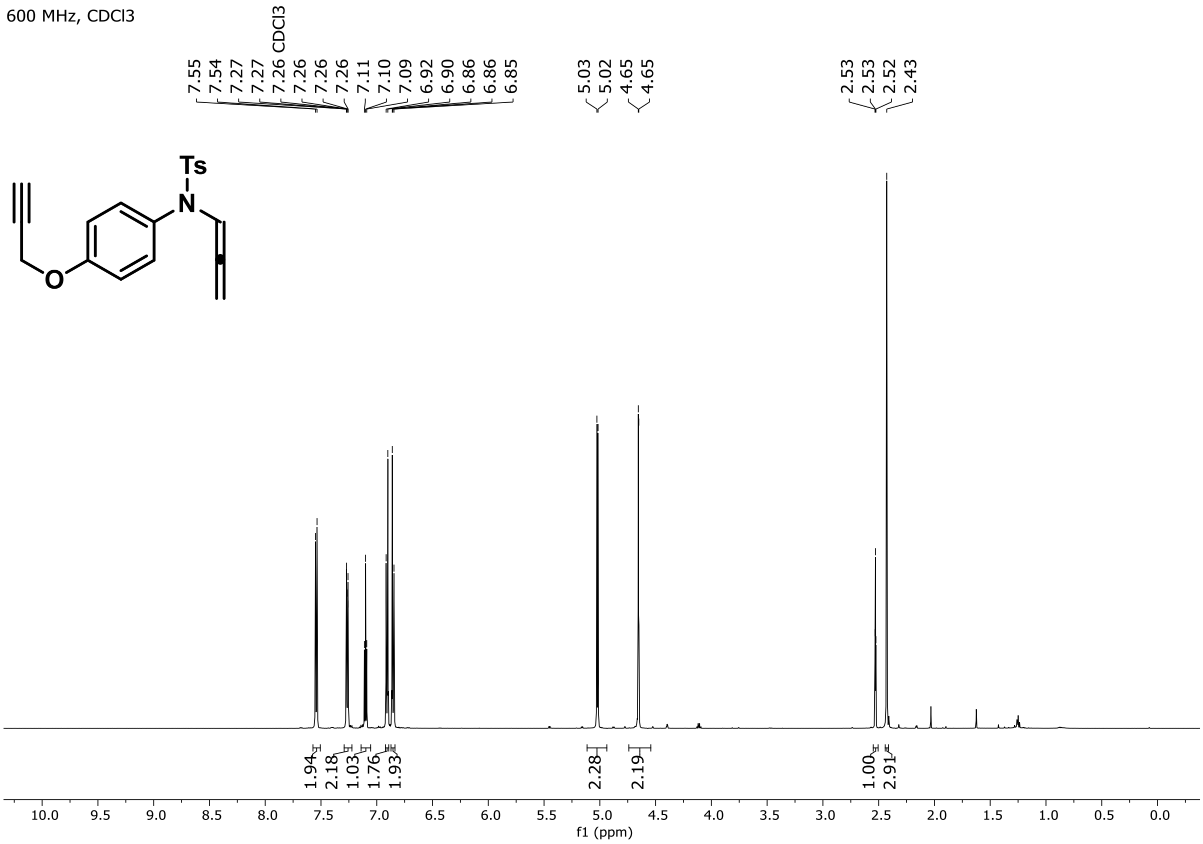

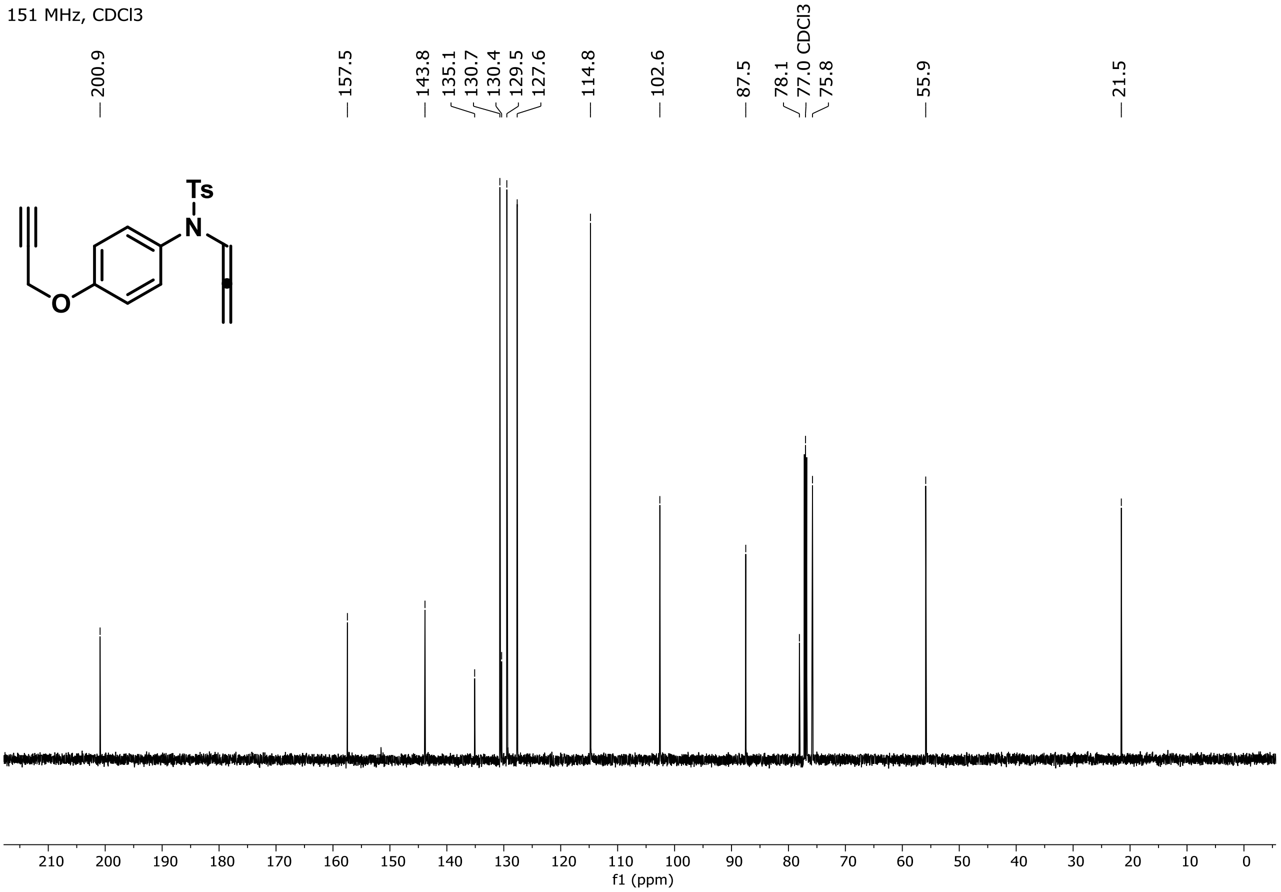

4-methyl-*N*-(propa-1,2-dien-1-yl)-*N*-(4-(propa-1,2-dien-1-yloxy)phenyl)benzenesulfonamide (**2xx**)

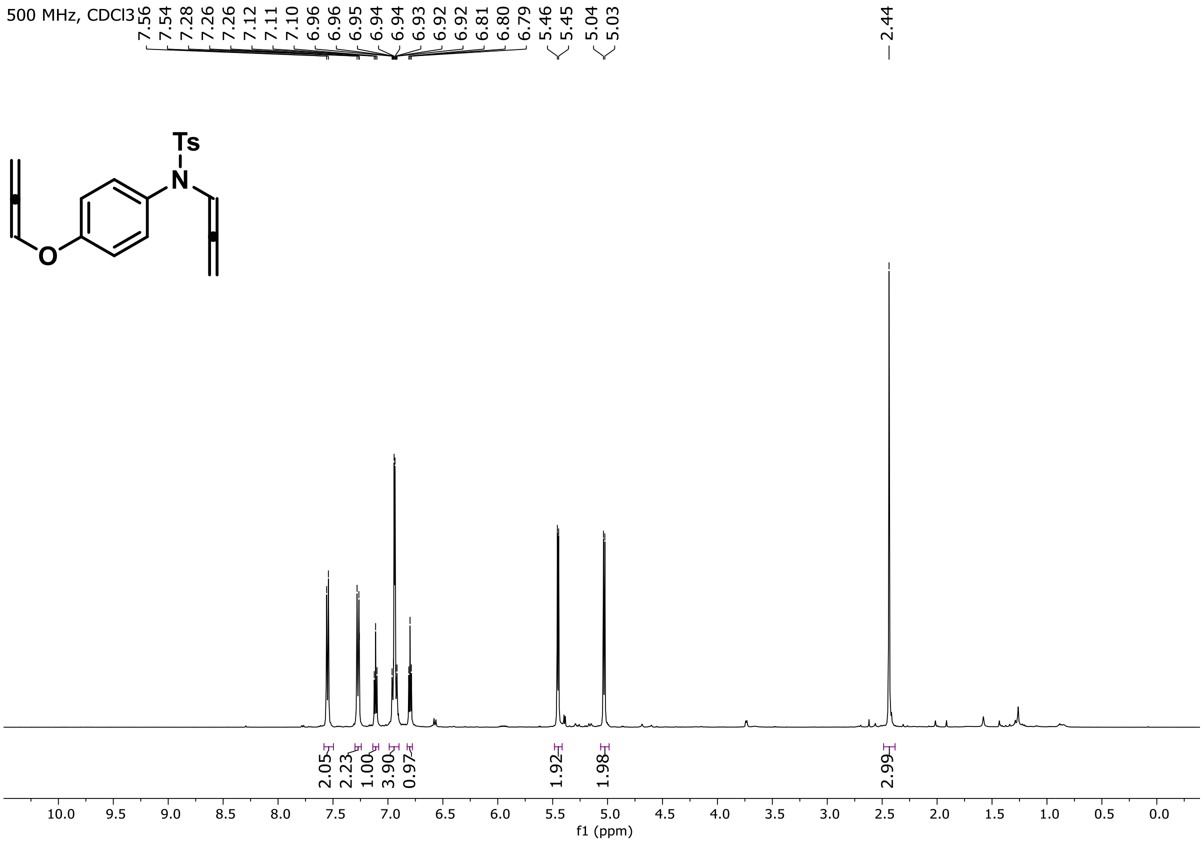

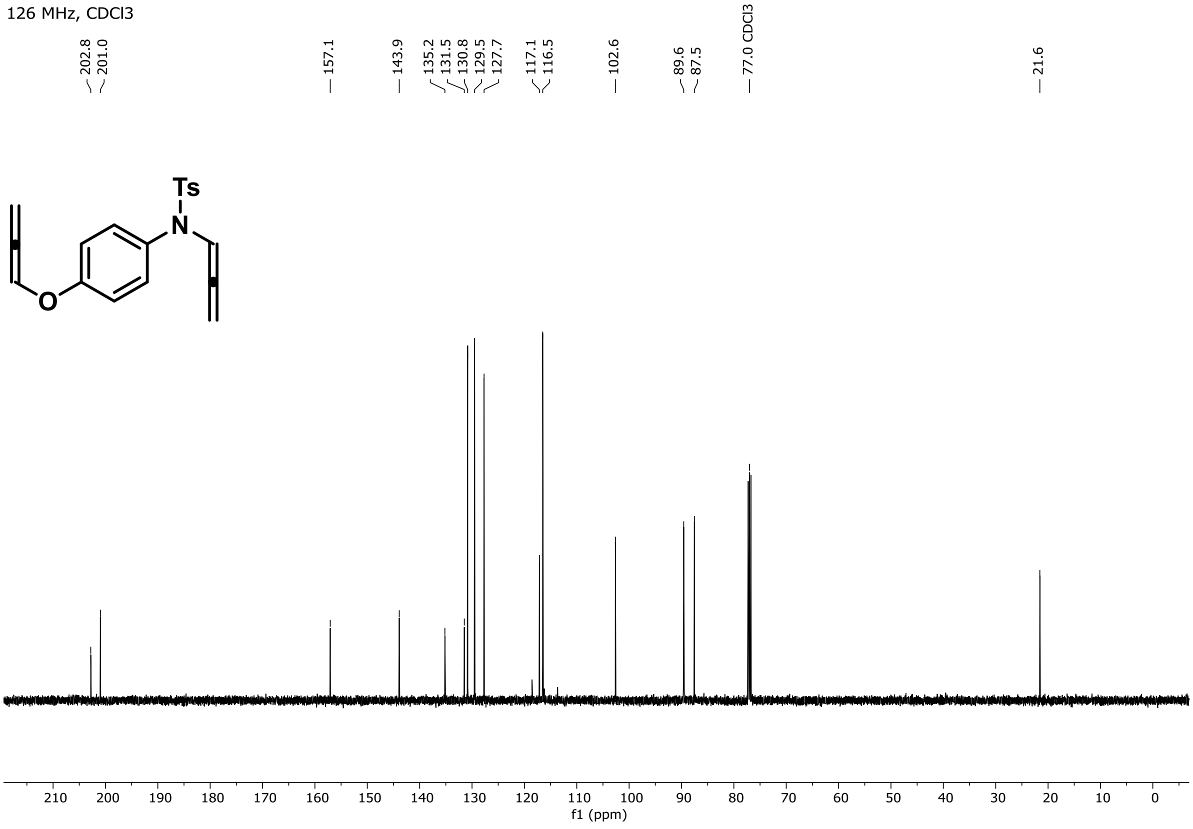

7. Theoretical considerations and calculations

7.1. General information

The calculation were carried out with Gaussian 16C software package[1] using ωB97XD functional.[2] Geometries were optimized with SMD solvation model – in acetonitrile,[3] using Def2-SVP basis set.[4] The acetonitrile SMD solvation model was used instead of defining a model for the 9:1 acetonitrile–tert-butanol mixture, due to the complete characterization of SMD parameters for acetonitrile in Gaussian 16, whereas solvent mixtures have not been fully parameterized.For each stationary point a thorough conformational analysis was performed in order to locate the conformer with the lowest energy. This was done by identifying key rotatable bonds and manually building possible starting geometries for optimizations. The identified lowest energy stationary points were then characterized by frequency calculations to confirm their character as minima (no imaginary frequencies) or transitions states (a single imaginary frequency). Transitions state character was confirmed also by IRC calculation as well as by manual relaxtion. The final energies were obtained from single-point calculations on the optimized geometries with a larger Def2-TZVP basis set[4](with SMD solvation) and were corrected for the thermodynamic effects at 25 °C using the quasi-harmonic approximation[5] (100 cm^{-1} cut-off) as implemented in GoodVibes program.[6]Electronic potential for each stationary point for was calculated in reference tocalculated Fc/Fc^+ pair. The shown potentials are not absolute[7] as no attempt was made to calibrate each class of species separately in isodesmic reactions with appropriate reference compounds with known redox potentials vs experimental Fc/Fc^+ value. The primary objective was solely to parameterize the reaction space.

To evaluate the accuracy of the computational methodology, selected geometries were reoptimized and characterized by frequency calculations with the Def2-TZVP basis set, and thermodynamic corrections at 25 °C were applied using the same approach as for Def2-SVP. Single-point electronic energies of selected stationary points (Table S3) were then recalculated using: (1) the ωB97XD functional with the Def2-TZVPP basis set,[4] and (3) DLPNO-CCSD(T)[8] (UHF reference) with the extrapolation to the basis set limit; both with the SMD solvation. The latter calculations were performed in Orca 6.0.1,[9] with the application of RIJCOSX[10] method and auxiliary basis sets Def2/J,[11] Def2/JK,[12] Def2-SVP/C,[13] and Def2-TZVP/C.[13] The extrapolation to the basis set limit was carried out using double-/triple-ζ scheme for both the SCF[14] and correlation energy[15] with Def2-SVP/Def2-TZVP basis sets, respectively, using the exponents α=10.39 and β=2.4.[16]All molecular representations shown in this work were rendered using XYZViewer v0.970 using default settings (scaling factor 1.0, weak perspective).[17]

The kinetic simulations were carried out using COPASI 4.46 software.[18] Zero-, first-, and second-order reversible rate laws were defined manually in the function section. The presented data were obtained using time-course simulations with an automatically determined number of intervals and a fixed interval size of 0.01 s for the defined reaction system and deterministic LSODA solver.Calculations with Gaussian 16C were carried outat LEM cluster of PLGrid WCSS (Alma Linux, with Intel Xeon Platinum 8462Y and AMD Epyc 9554 proccesors). The DLPNO-CCSD(T) single-point energies (Orca 6.0.1), thermodynamical corrections (Goodvibes), kinetic simulations (COPASI 4.46)and data treatment were performed on HPZ840 workstation (Ubuntu 24.04.4 LTS, with 2x Intel Xeon E5-2698 v3).

7.2. Notation for Structures and Thermodynamic Parameters

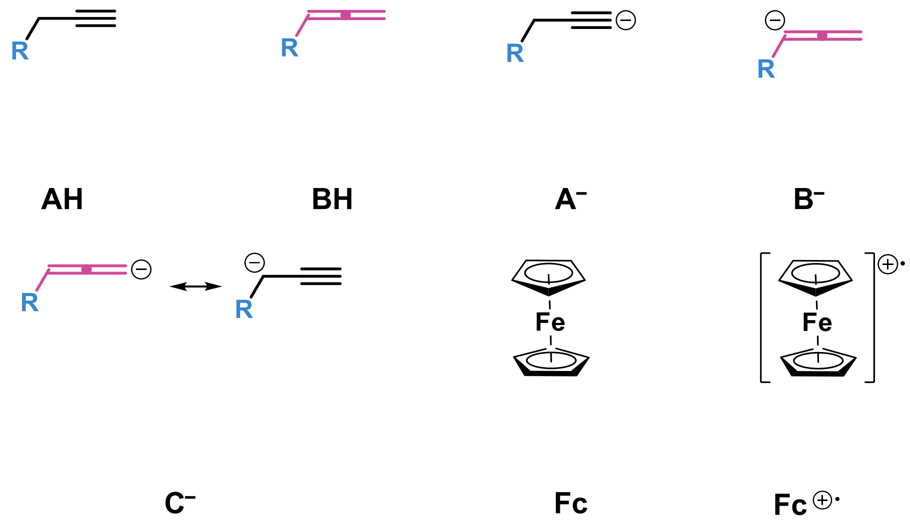

Figure TS1. Notation for structures.

$\mathrm{AH} + t\text{-BuO}^{\ominus} \rightleftharpoons \mathrm{A}^{\ominus} + t\text{-BuOH}$	$\Delta G\ (\mathrm{AH/A^{\ominus}})$
$\mathrm{BH} + t\text{-BuO}^{\ominus} \rightleftharpoons \mathrm{B}^{\ominus} + t\text{-BuOH}$	$\Delta G\ (\mathrm{BH/B^{\ominus}})$
$\mathrm{BH} + t\text{-BuO}^{\ominus} \rightleftharpoons \mathrm{A}^{\ominus} + t\text{-BuOH}$	$\Delta G\ (\mathrm{BH/A^{\ominus}})$
$\mathrm{A}^{\ominus} \rightleftharpoons \mathrm{B}^{\ominus}$	$\Delta G\ (\mathrm{A^{\ominus}/B^{\ominus}})$
$\mathrm{A}^{\ominus} \rightleftharpoons \mathrm{C}^{\ominus}$	$\Delta G\ (\mathrm{A^{\ominus}/C^{\ominus}})$
$\mathrm{B}^{\ominus} \rightleftharpoons \mathrm{C}^{\ominus}$	$\Delta G\ (\mathrm{B^{\ominus}/C^{\ominus}})$
$\mathrm{AH} \rightleftharpoons \mathrm{BH}$	$\Delta G\ (\mathrm{AH/BH})$

Figure TS2. Equilibrium reactions and their Gibbs free energies.

$\mathrm{AH} + \mathrm{Fc}^{\oplus\cdot} \underset{}{\overset{+e^{\ominus}}{\rightleftharpoons}} \mathrm{AH}^{\oplus\cdot} + \mathrm{Fc}$	$E_{ox}\ (\mathrm{AH})$
$\mathrm{BH} + \mathrm{Fc}^{\oplus\cdot} \overset{+e^{\ominus}}{\rightleftharpoons} \mathrm{BH}^{\oplus\cdot} + \mathrm{Fc}$	$E_{ox}\ (\mathrm{BH})$
$\mathrm{AH} + \mathrm{Fc} \overset{+e^{\ominus}}{\rightleftharpoons} \mathrm{AH}^{\ominus\cdot} + \mathrm{Fc}^{\oplus\cdot}$	$E_{red}\ (\mathrm{AH})$
$\mathrm{BH} + \mathrm{Fc} \overset{+e^{\ominus}}{\rightleftharpoons} \mathrm{BH}^{\ominus\cdot} + \mathrm{Fc}^{\oplus\cdot}$	$E_{red}\ (\mathrm{BH})$
$\mathrm{A}^{\ominus} + \mathrm{Fc}^{\oplus\cdot} \overset{+e^{\ominus}}{\rightleftharpoons} \mathrm{A}^{\cdot} + \mathrm{Fc}$	$E_{ox}\ (\mathrm{A}^{\ominus})$
$\mathrm{B}^{\ominus} + \mathrm{Fc}^{\oplus\cdot} \overset{+e^{\ominus}}{\rightleftharpoons} \mathrm{B}^{\cdot} + \mathrm{Fc}$	$E_{ox}\ (\mathrm{B}^{\ominus})$
$\mathrm{C}^{\ominus} + \mathrm{Fc}^{\oplus\cdot} \overset{+e^{\ominus}}{\rightleftharpoons} \mathrm{C}^{\cdot} + \mathrm{Fc}$	$E_{ox}\ (\mathrm{C}^{\ominus})$

Figure TS3. Half-cell equations for electrochemical potentials.

7.3. Kinetics simulations for 1a isomerization

Model assumptions

To illustrate the reaction dynamics and the approach to equilibrium, kinetic simulations on simplified zero-dimensional minimal model were performed using COPASI. In the absence of experimental kinetic data, a sensivity-analysis approach was performed by two complementary modes of consideration. At first one (Figures S6-S15) identical forward rate constants for exergonic process (k_1) were assigned to all elementary steps and varied systematically over a broad range (10^1–10^{10} L·mol^{-1}·s^{-1} for bimolecular reactions or s^{-1} for unimolecular steps). At complementary approach (Figures S16-S19) the deprotonation k_1 was considered as ruled by restrictedslow diffusion regime (10^8 L·mol^{-1}·s^{-1}) and isomerizations k_1 were scanned (10^2/10^4/10^6/10^{10}s^{-1}). The corresponding reverse rate constants (k_2) were calculated from the Gibbs free energy differences (ΔG) using the thermodynamic relationship between k_1 and k_2. To compare different base delivery modes, simulations were conducted with initial *tert*-butoxide concentrations of 0.02, 0.05, and 0.1 M, without further base addition (Figures S20–25).

In constructing the model, only pathways involving single proton-transfer events were considered; direct interconversion between A and B but also protonation of higher-energy intermediates (e.g., C → BH) were not included. Protonation steps were evaluated exclusively for the lowest-energy anion accessible via a single deprotonation event. Base generation was modeled as a zero-order process, in which *tert*-butoxide is formed at a constant rate determined by the applied current, assuming 100% faradaic efficiency – oxidation of chloride anion was not considered. The conversion of A to B was assumed to proceed exclusively via intermediate C (Figure S5), while a concerted two-proton transfer pathway was not included. Accordingly, these transformations were described by two first-order kinetic equations, whereas the deprotonation steps of AH and BH were modeled using second-order rate laws. The concentration of *tert*-butanol was calculated without accounting for volume contraction effects. Magnesium was defined as an infinite reservoir, as electrode passivation was omitted in the model. The initial concentration of AH was set to 0.05 mol·dm^{-3}, in accordance with the experimental conditions. The reaction volume was assumed to remain constant (10 mL), since volume changes resulting from *tert*-butanol electrolysis were considered negligible. Given the simplified kinetic zero-dimensional framework and the assumptions adopted for the electrochemical system, the simulations are intended to deliver qualitative insight into the reaction network and equilibrium dynamics, rather than quantitative rate or reaction time predictions.

$$\frac{d([AH]\cdot V_{Cell})}{dt} = -V_{Cell}\cdot(k1_{(R2)}\cdot[AH]\cdot[tBuO] - k2_{(R2)}\cdot[A]\cdot[tBuOH])$$

$$\frac{d([B]\cdot V_{Cell})}{dt} = +V_{Cell}\cdot(k1_{(R4)}\cdot[C] - k2_{(R4)}\cdot[B]) - V_{Cell}\cdot(k1_{(R5)}\cdot[B]\cdot[tBuOH] - k2_{(R5)}\cdot[BH]\cdot[tBuO])$$

$$\frac{d([C]\cdot V_{Cell})}{dt} = +V_{Cell}\cdot(k1_{(R3)}\cdot[A] - k2_{(R3)}\cdot[C]) - V_{Cell}\cdot(k1_{(R4)}\cdot[C] - k2_{(R4)}\cdot[B])$$

$$\frac{d([BH]\cdot V_{Cell})}{dt} = +V_{Cell}\cdot(k1_{(R5)}\cdot[B]\cdot[tBuOH] - k2_{(R5)}\cdot[BH]\cdot[tBuO])$$

$$\frac{d([Mg]\cdot V_{Cell})}{dt} = -V_{Cell}\cdot(k_{(R1)}) \qquad \frac{d([Mg\_cat]\cdot V_{Cell})}{dt} = +V_{Cell}\cdot(k_{(R1)})$$

$$\frac{d([A]\cdot V_{Cell})}{dt} = +V_{Cell}\cdot(k1_{(R2)}\cdot[AH]\cdot[tBuO] - k2_{(R2)}\cdot[A]\cdot[tBuOH]) - V_{Cell}\cdot(k1_{(R3)}\cdot[A] - k2_{(R3)}\cdot[C])$$

$$\frac{d([tBuO]\cdot V_{Cell})}{dt} = +V_{Cell}\cdot(k_{(R1)}) - V_{Cell}\cdot(k1_{(R2)}\cdot[AH]\cdot[tBuO] - k2_{(R2)}\cdot[A]\cdot[tBuOH]) + V_{Cell}\cdot(k1_{(R5)}\cdot[B]\cdot[tBuOH] - k2_{(R5)}\cdot[BH]\cdot[tBuO])$$

$$\frac{d([tBuOH]\cdot V_{Cell})}{dt} = -V_{Cell}\cdot(k_{(R1)}) + V_{Cell}\cdot(k1_{(R2)}\cdot[AH]\cdot[tBuO] - k2_{(R2)}\cdot[A]\cdot[tBuOH]) - V_{Cell}\cdot(k1_{(R5)}\cdot[B]\cdot[tBuOH] - k2_{(R5)}\cdot[BH]\cdot[tBuO])$$

Figure TS4. Differential equations describing the change in the total amount (n = c·V) of each chemical species.

$$\mathbf{Mg} + t\text{-}\mathbf{BuOH} \longrightarrow \mathbf{Mg}^{\oplus} + t\text{-}\mathbf{BuO}^{\ominus} \qquad I = 5\ mA$$

$$\mathbf{AH} + t\text{-}\mathbf{BuO}^{\ominus} \underset{k_2}{\overset{k_1}{\rightleftharpoons}} \mathbf{A}^{\ominus} + t\text{-}\mathbf{BuOH} \qquad \Delta G\ (AH/A^{\ominus})$$

$$\mathbf{BH} + t\text{-}\mathbf{BuO}^{\ominus} \underset{k_2}{\overset{k_1}{\rightleftharpoons}} \mathbf{B}^{\ominus} + t\text{-}\mathbf{BuOH} \qquad \Delta G\ (BH/B^{\ominus})$$

$$\mathbf{A}^{\ominus} \underset{k_1}{\overset{k_2}{\rightleftharpoons}} \mathbf{C}^{\ominus} \qquad \Delta G\ (A^{\ominus}/C^{\ominus})$$

$$\mathbf{B}^{\ominus} \underset{k_1}{\overset{k_2}{\rightleftharpoons}} \mathbf{C}^{\ominus} \qquad \Delta G\ (B^{\ominus}/C^{\ominus})$$

Figure TS5. Reaction equations defined in the kinetic model.

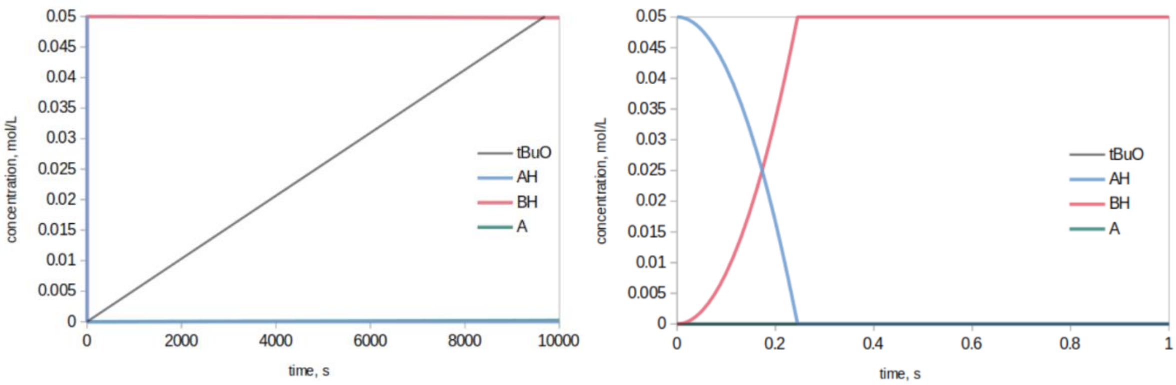

Figure TS6. Simulation of kinetics of *1a* isomerization for identical forward rate constant of each reaction step $k_1=10^{10}$.

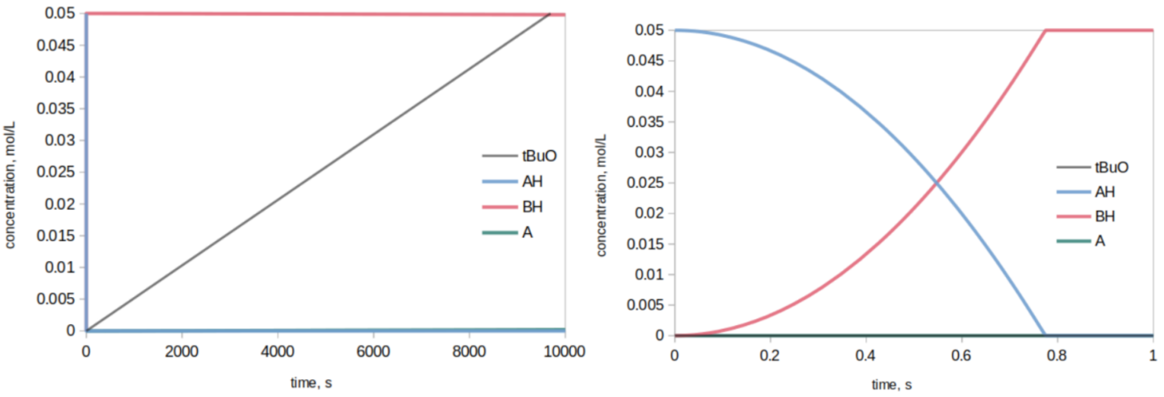

Figure TS7. Simulation of kinetics of *1a* isomerization for identical forward rate constant of each reaction step $k_1=10^9$.

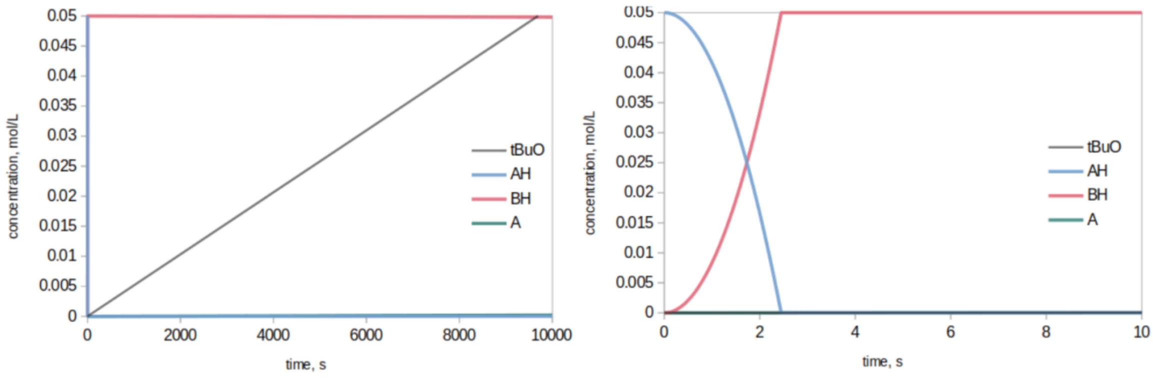

Figure TS8. Simulation of kinetics of *1a* isomerization for identical forward rate constant of each reaction step $k_1=10^8$.

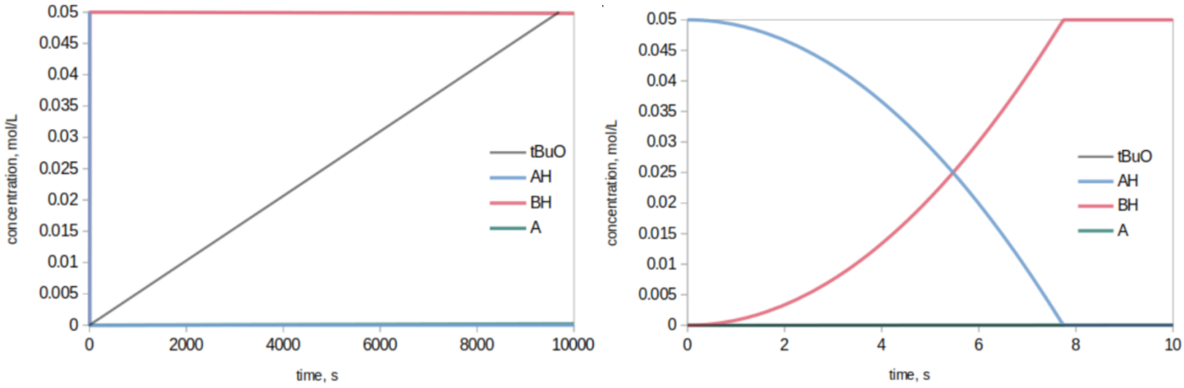

Figure TS9. Simulation of kinetics of *1a* isomerization for identical forward rate constant of each reaction step $k_1=10^7$.

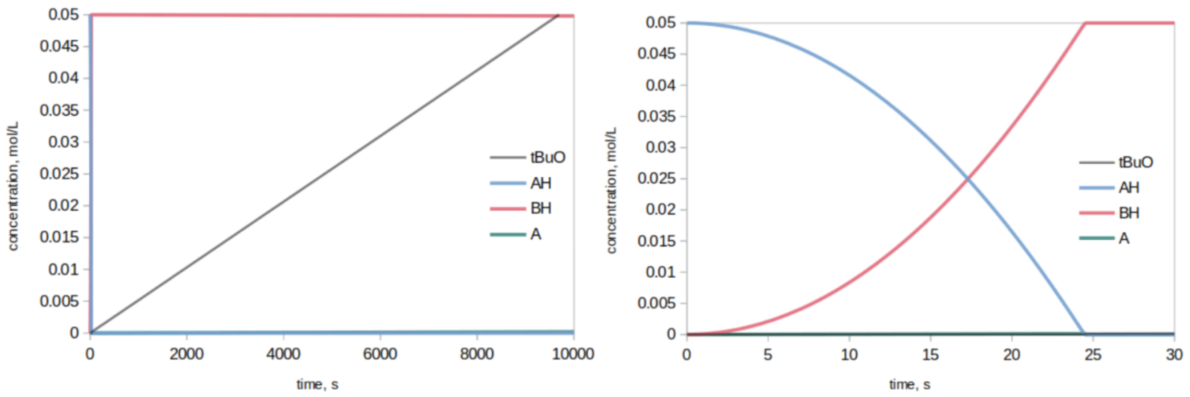

Figure TS10. Simulation of kinetics of *1a* isomerization for identical forward rate constant of each reaction step $k_1=10^6$.

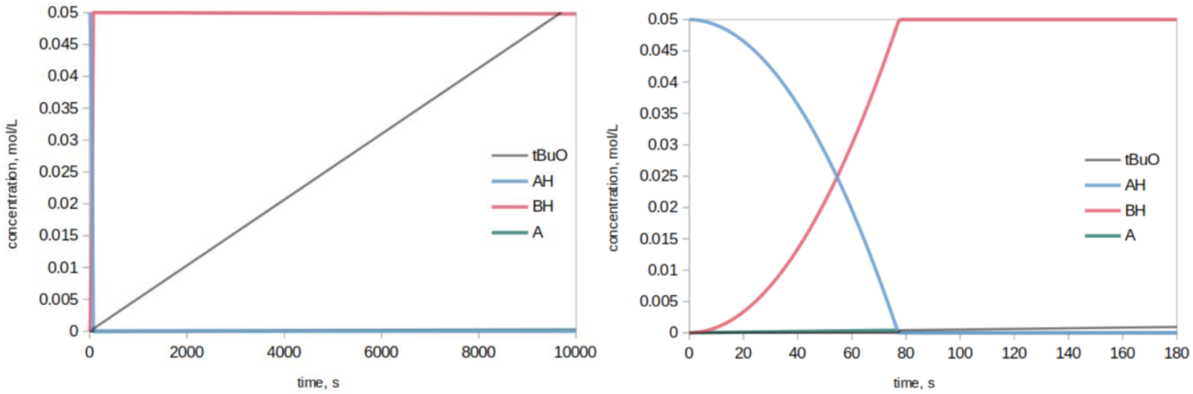

Figure TS11. Simulation of kinetics of *1a* isomerization for identical forward rate constant of each reaction step $k_1=10^5$.

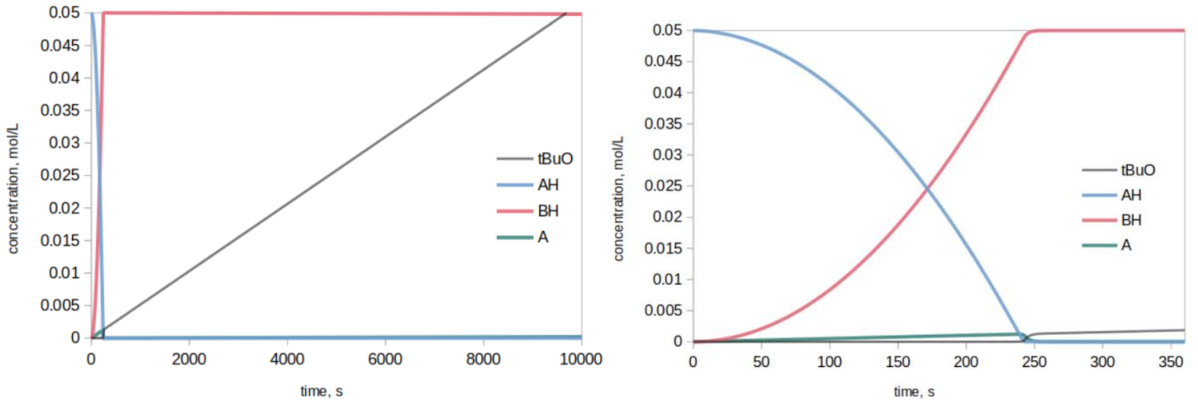

Figure TS12. Simulation of kinetics of *1a* isomerization for identical forward rate constant of each reaction step $k_1=10^4$.

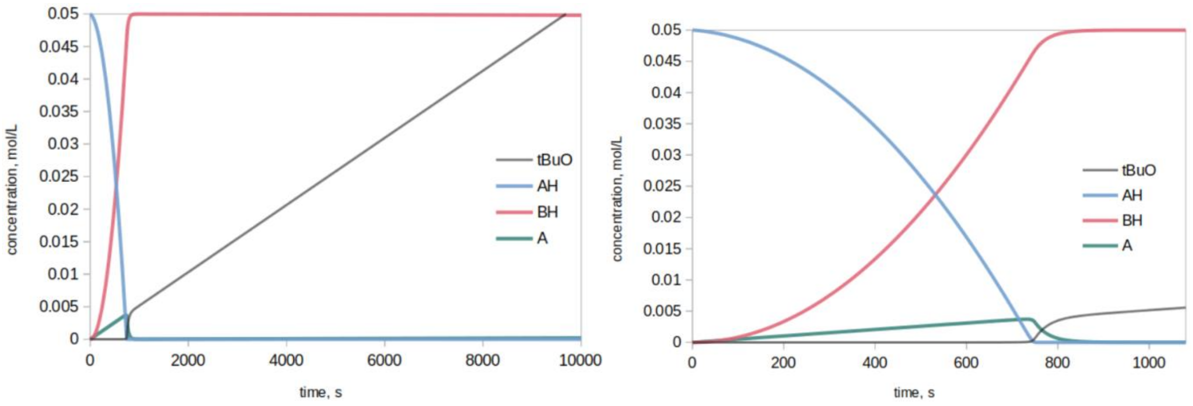

Figure TS13. Simulation of kinetics of *1a* isomerization for identical forward rate constant of each reaction step $k_1=10^3$.

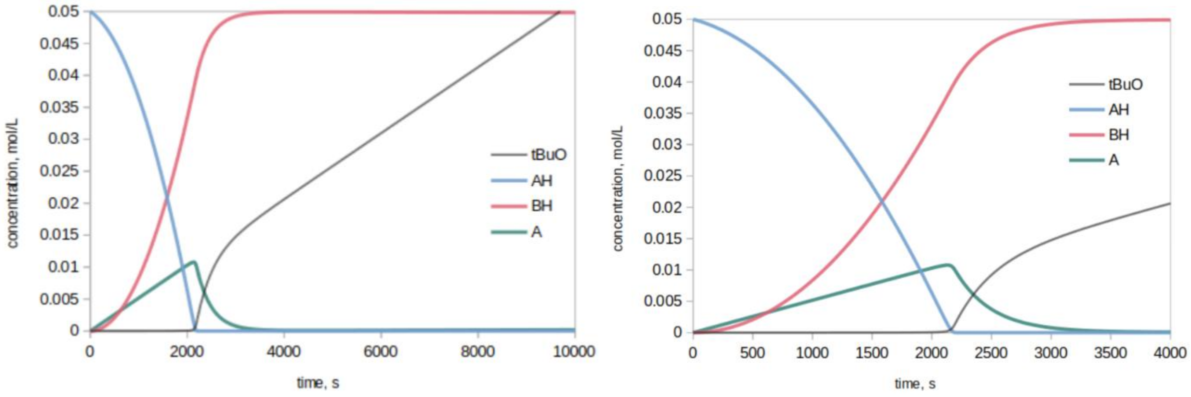

Figure TS14. Simulation of kinetics of *1a* isomerization for identical forward rate constant of each reaction step $k_1=10^2$.

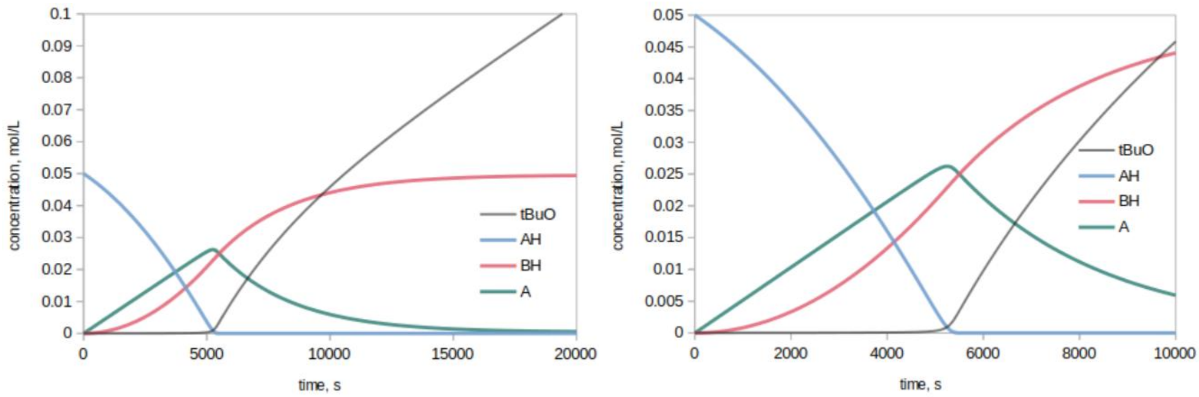

Figure TS15. Simulation of kinetics of *1a* isomerization for identical forward rate constant of each reaction step $k_1=10$.

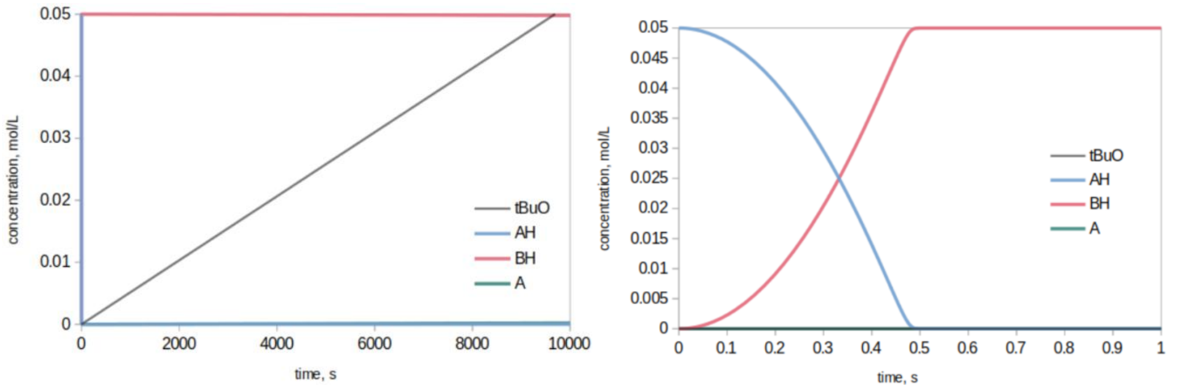

Figure TS16. Simulation of kinetics of *1a* isomerization for deprotonation forward rate constant equal 10^8 and anion interconversion forward rate constant equal to 10^{10}.

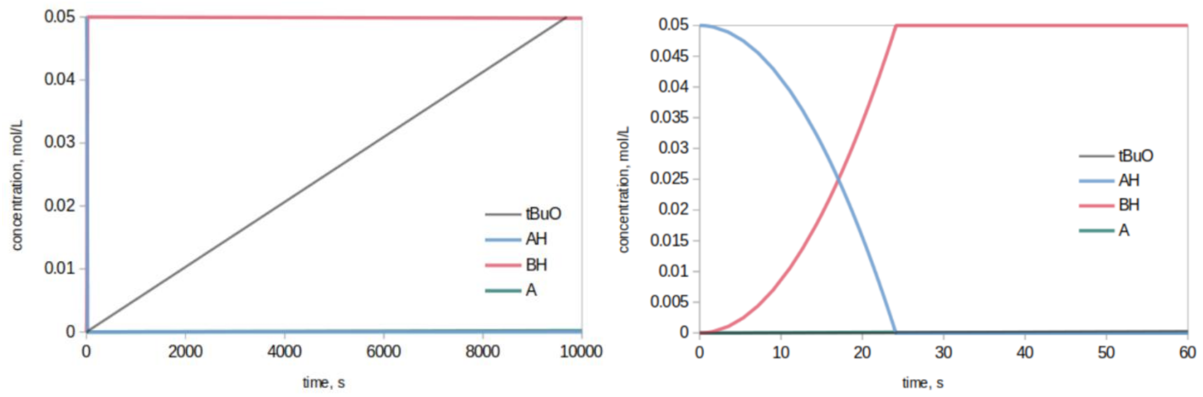

Figure TS17. Simulation of kinetics of *1a* isomerization for deprotonation forward rate constant equal 10^8 and anion interconversion forward rate constant equal to 10^6.

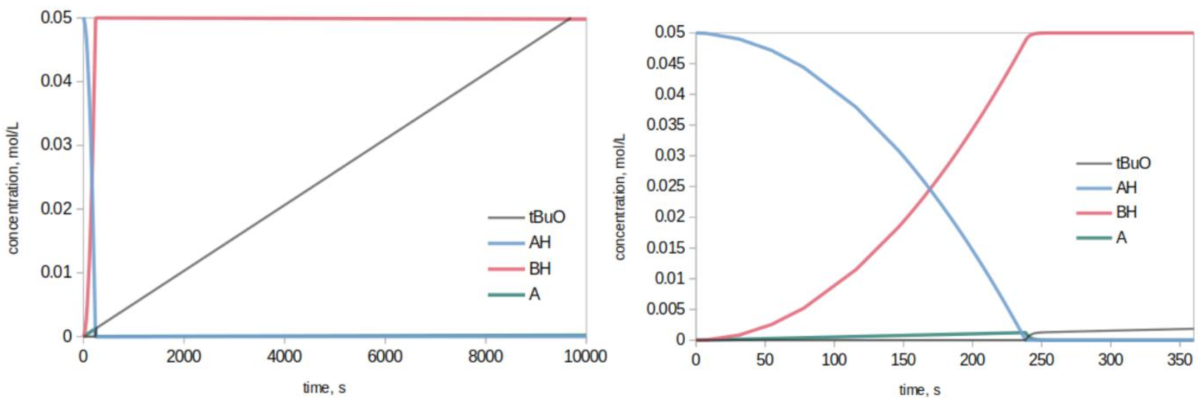

Figure TS18. Simulation of kinetics of *1a* isomerization for deprotonation forward rate constant equal 10^8 and anion interconversion forward rate constant equal to 10^4.

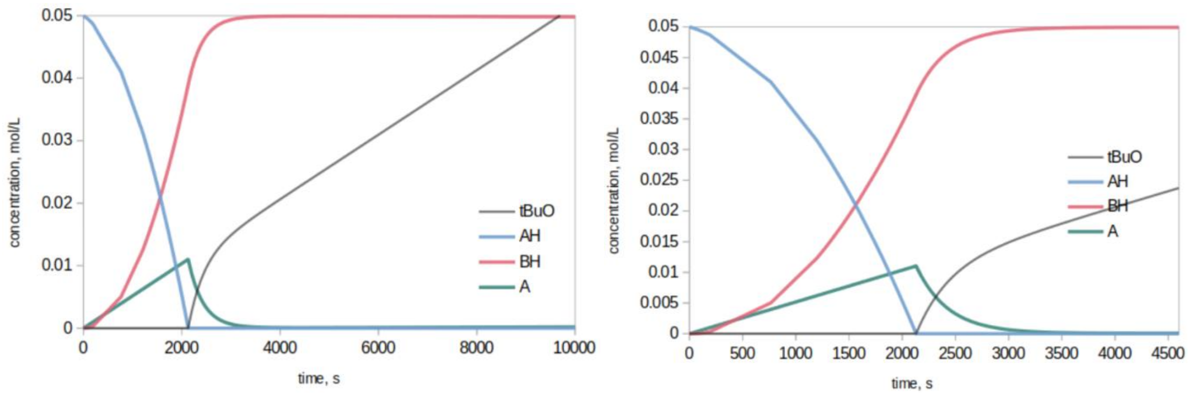

Figure TS19. Simulation of kinetics of *1a* isomerization for deprotonation forward rate constant equal 10^8 and anion interconversion forward rate constant equal to 10^2.

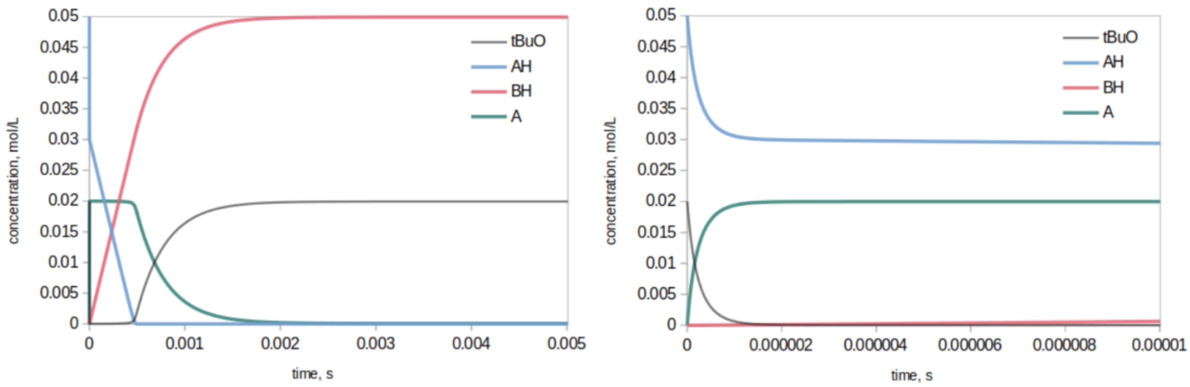

Figure TS20. Simulation of kinetics of *1a* isomerization for identical forward rate of each step $k_1=10^8$and initial *t*-BuO$^{\ominus}$ concentration 0.02 M and no further addition.

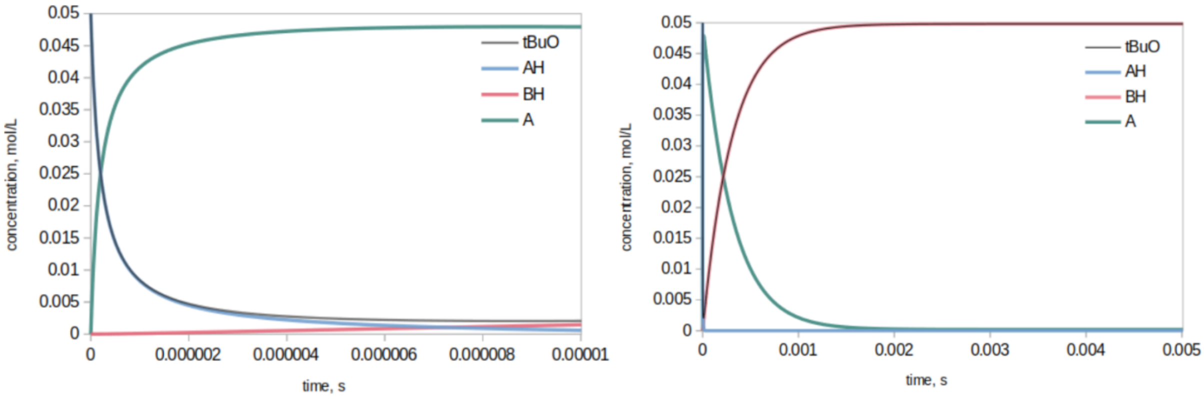

Figure TS21. Simulation of kinetics of *1a* isomerization for identical forward rate of each step $k_1=10^8$and initial *t*-BuO$^{\ominus}$ concentration 0.05 M and no further addition.

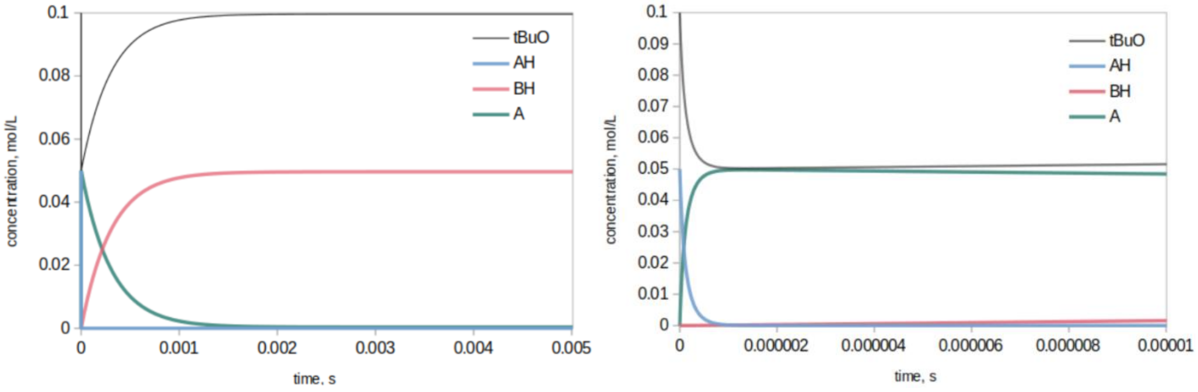

Figure TS22. Simulation of kinetics of *1a* isomerization for identical forward rate of each step $k_1=10^8$and initial *t*-BuO$^\ominus$ concentration 0.1 M and no further addition.

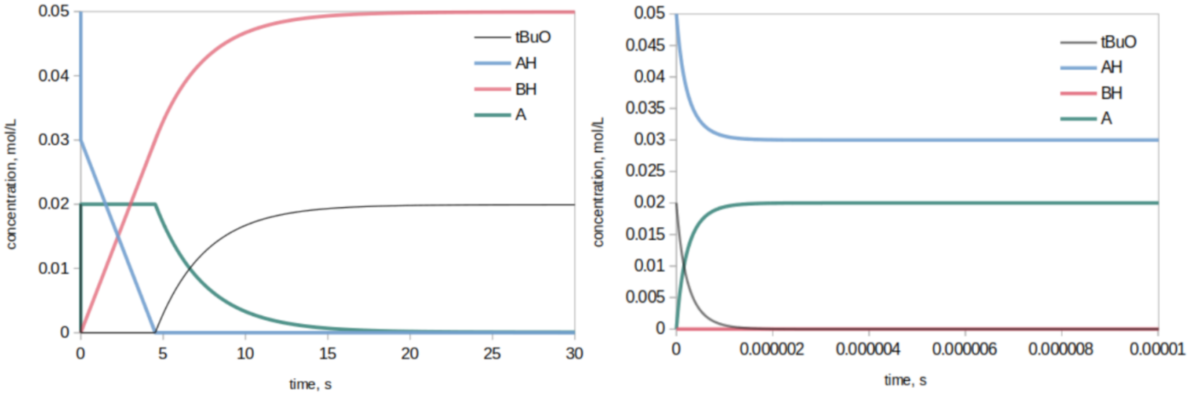

Figure TS23. Simulation of kinetics of *1a* isomerization for deprotonation forward rate constant equal 10^8, anion interconversion forward rate constant equal to $10^4$and initial *t*-BuO$^\ominus$ concentration 0.02 M and no further addition.

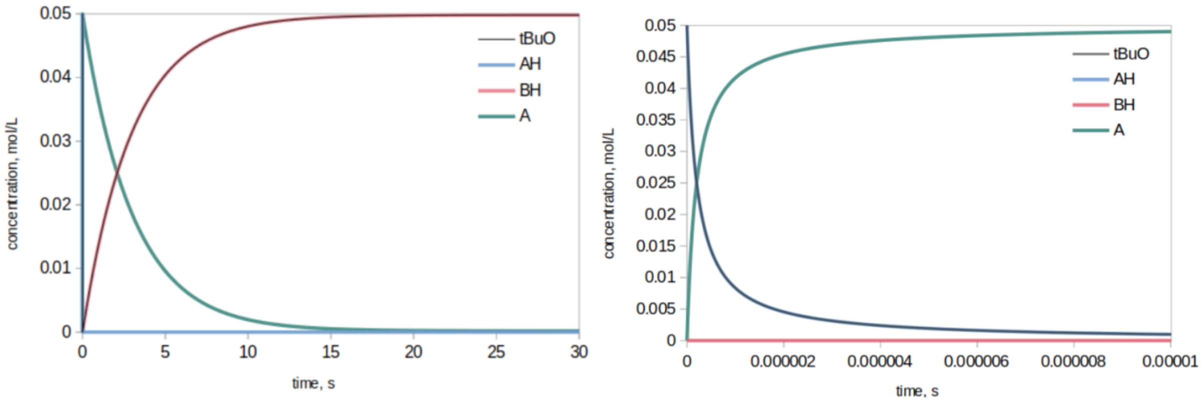

Figure TS24. Simulation of kinetics of *1a* isomerization for deprotonation forward rate constant equal 10^8, anion interconversion forward rate constant equal to $10^4$and initial *t*-BuO$^\ominus$ concentration 0.05 M and no further addition.

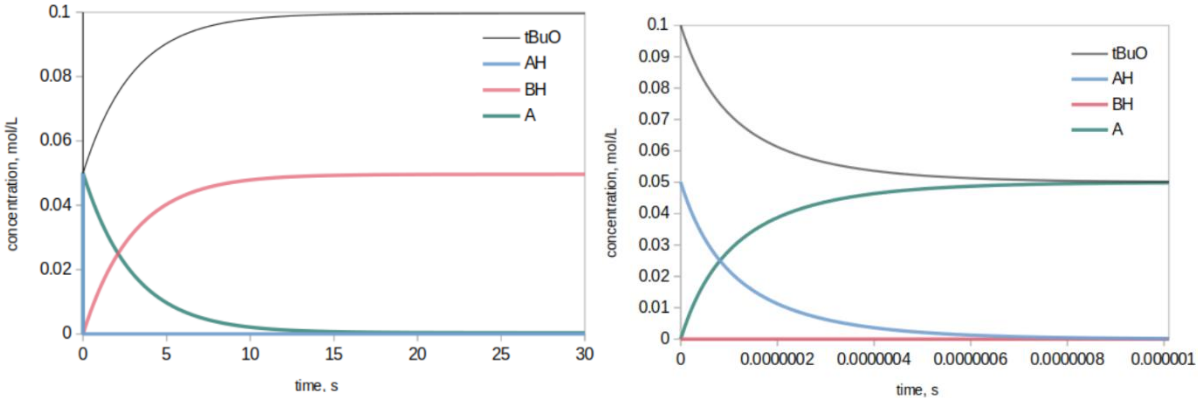

Figure TS25. Simulation of kinetics of *1a* isomerization for deprotonation forward rate constant equal 10^8, anion interconversion forward rate constant equal to $10^4$and initial *t*-BuO$^{\ominus}$ concentration 0.01 M and no further addition.

7.4. Thermodynamic scatter plots

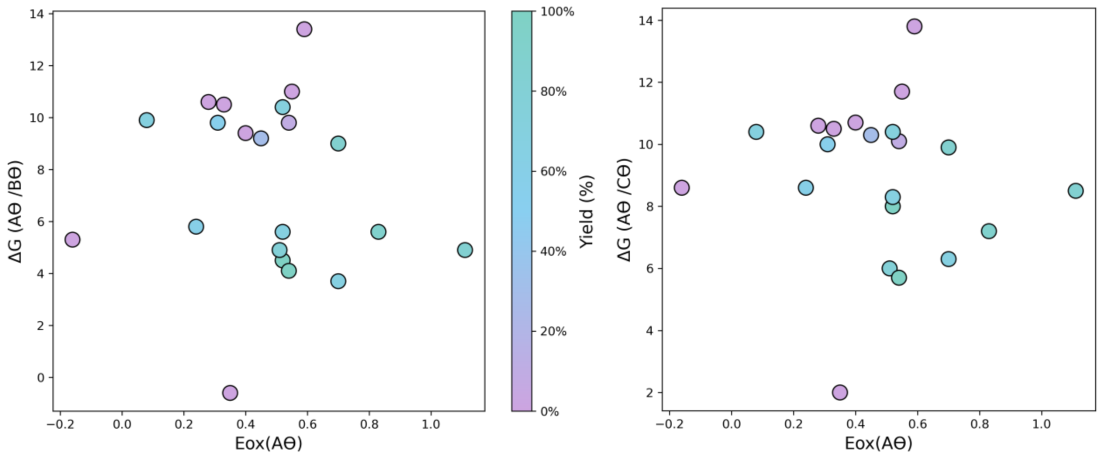

Figure TS26. Distribution of products yield (color scale) at functions of E_{ox}(A$^{\ominus}$), and ΔG (A$^{\ominus}$ /B$^{\ominus}$) (left) or ΔG (A$^{\ominus}$ /C$^{\ominus}$) (right).

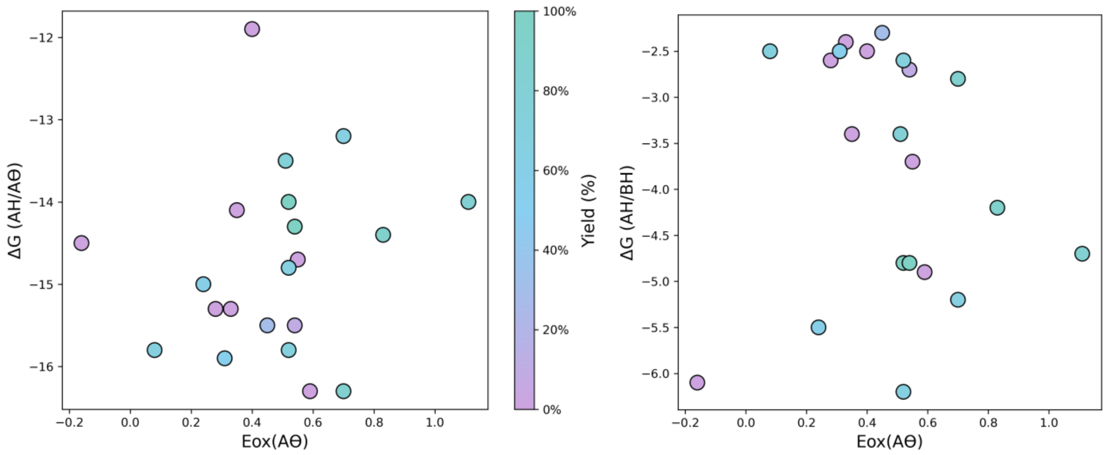

Figure TS27. Distribution of products yield (color scale) at functions of E_{ox} (A$^{\ominus}$), and ΔG (AH/A$^{\ominus}$) (left) or ΔG (AH/BH) (right).

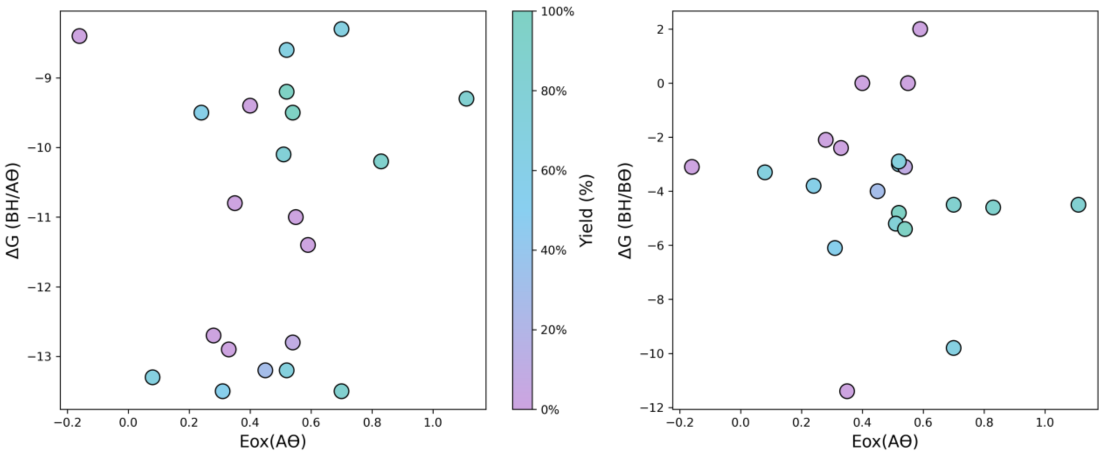

Figure TS28. Distribution of products yield (color scale) at functions of E_{ox} ($A^{\ominus}$), and ΔG (BH/$A^{\ominus}$) (left) or ΔG (AH/BH) (right).

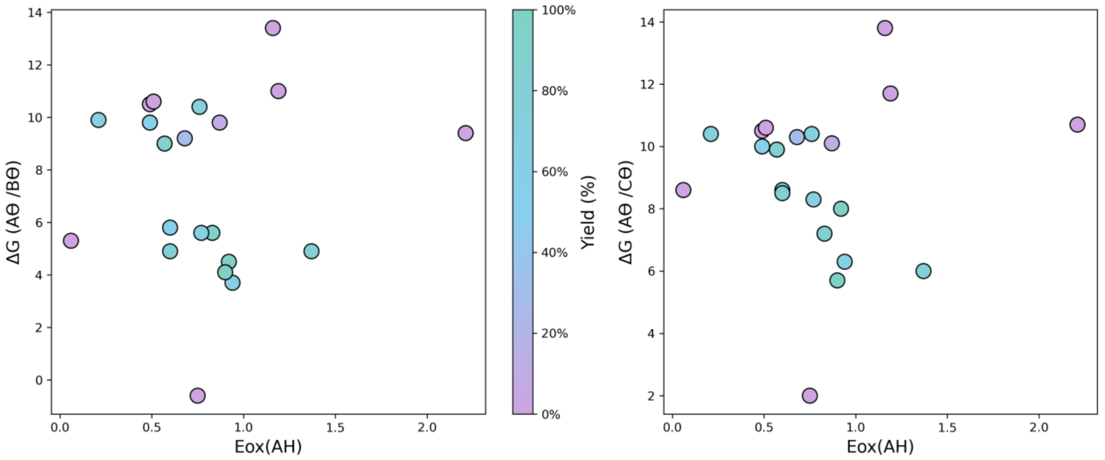

Figure TS29. Distribution of products yield (color scale) at functions of E_{ox} (AH), and ΔG ($A^{\ominus}$ /$B^{\ominus}$) (left) or ΔG ($A^{\ominus}$ / $C^{\ominus}$) (right).

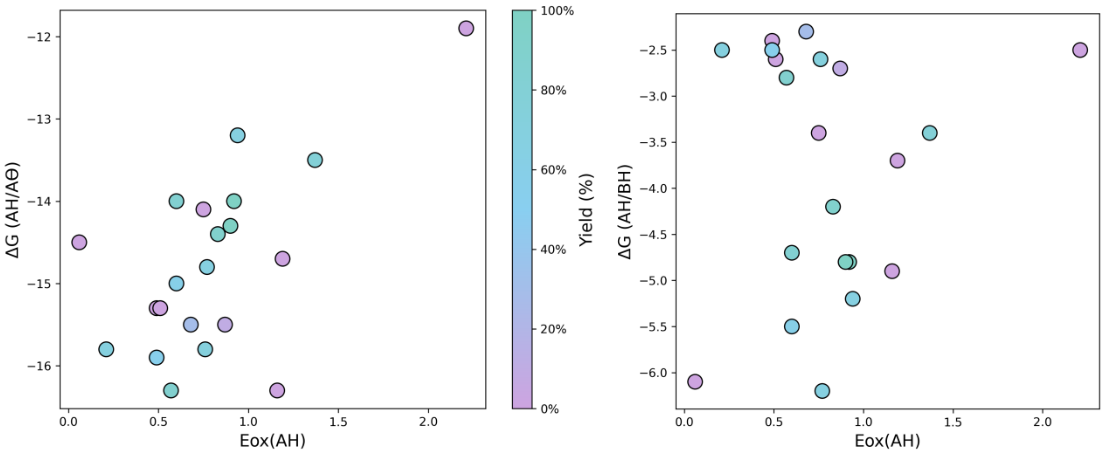

Figure TS30. Distribution of products yield (color scale) at functions of E_{ox}(AH), and ΔG (AH/$A^{\ominus}$) (left) or ΔG (AH / BH) (right).

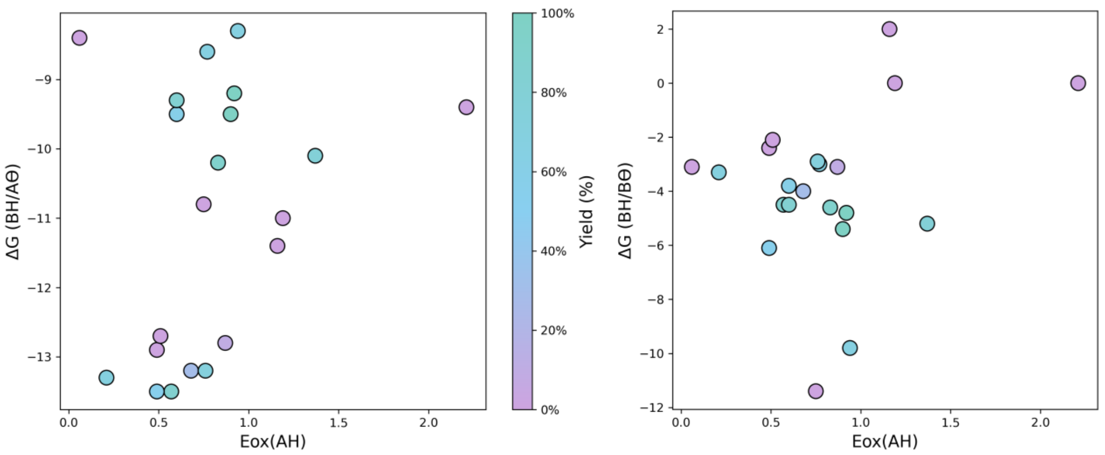

Figure TS31. Distribution of products yield (color scale) at functions of E_{ox}(AH), and ΔG (BH /$A^{\ominus}$) (left) or ΔG ($BH^{\ominus}$ / $B^{\ominus}$) (right).

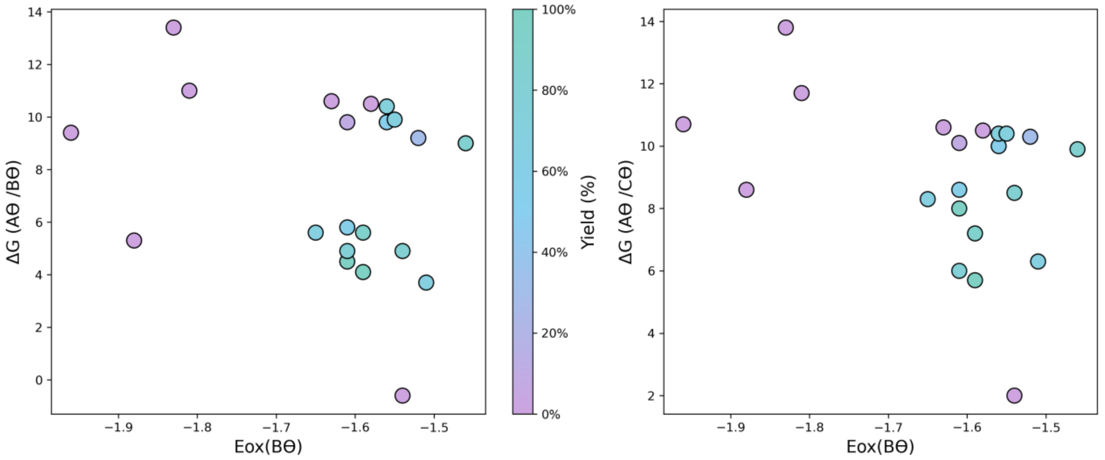

Figure TS32. Distribution of products yield (color scale) at functions of E_{ox}($B^{\ominus}$), and ΔG ($A^{\ominus}$ /$B^{\ominus}$) (left) or ΔG ($A^{\ominus}$ / $C^{\ominus}$) (right).

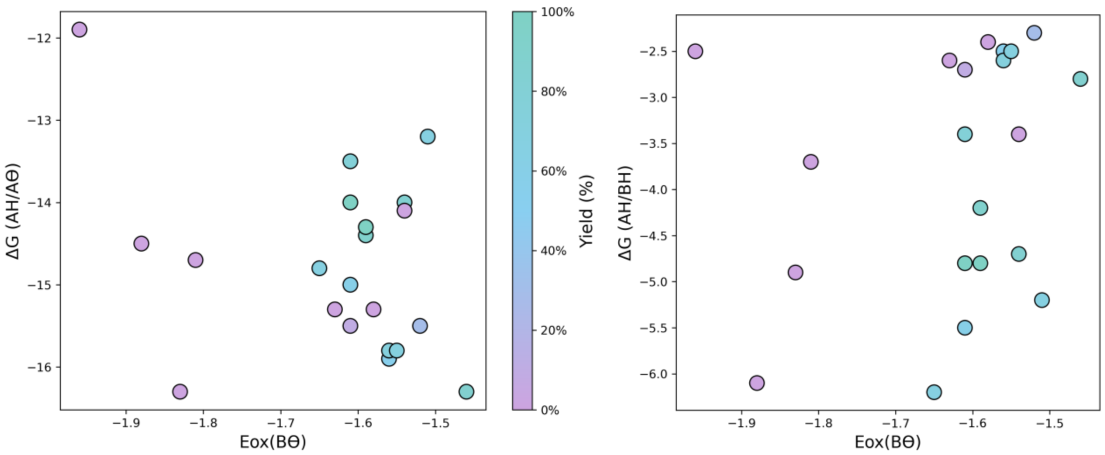

Figure TS33. Distribution of products yield (color scale) at functions of E_{ox} ($B^{\ominus}$), and ΔG (AH /$A^{\ominus}$) (left) or ΔG (AH/ BH) (right).

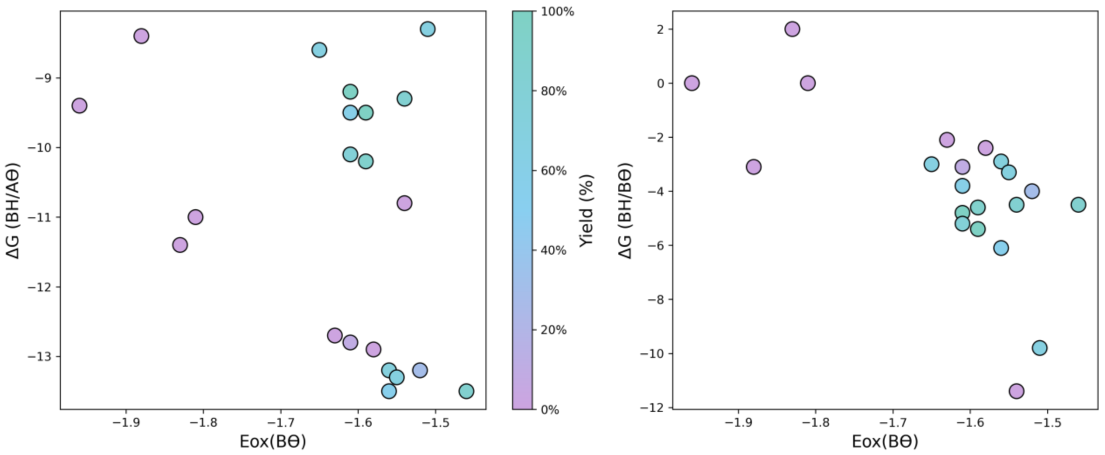

Figure TS34. Distribution of products yield (color scale) at functions of $E_{ox}(B^{\ominus})$, and ΔG (BH /$A^{\ominus}$) (left) or ΔG ($BH^{\ominus}$ / $B^{\ominus}$) (right).

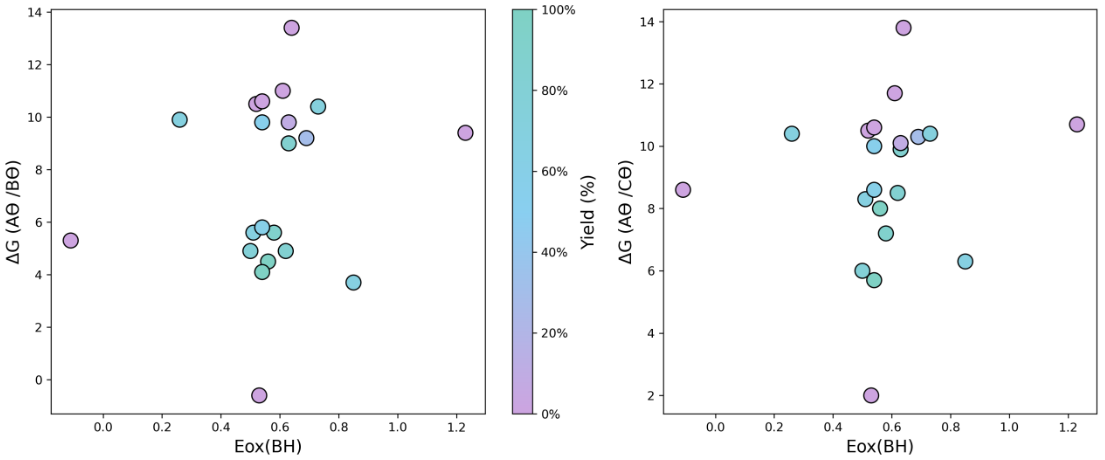

Figure TS35. Distribution of products yield (color scale) at functions of E_{ox} (BH), and ΔG (BH /$A^{\ominus}$) (left) or ΔG ($BH^{\ominus}$ / $B^{\ominus}$) (right).

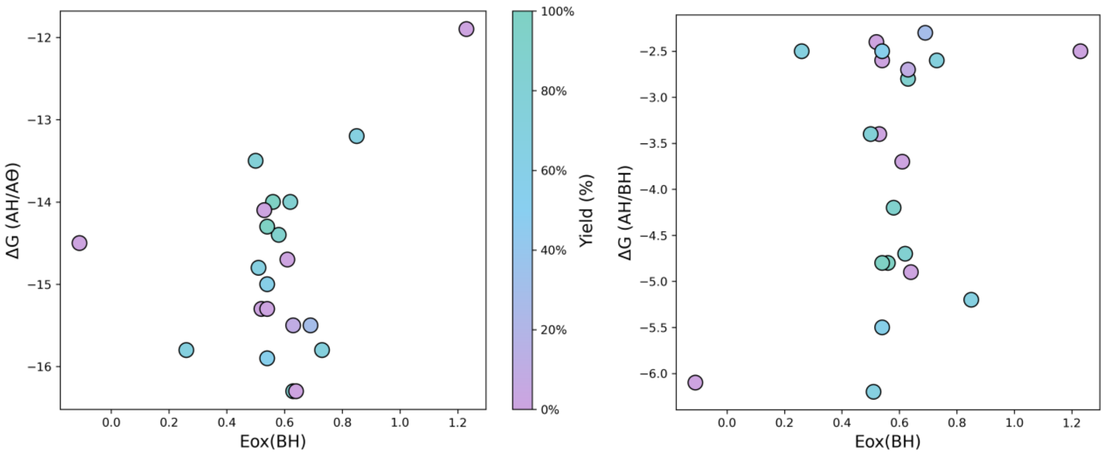

Figure TS36. Distribution of products yield (color scale) at functions of E_{ox}(BH), and ΔG (AH /$A^{\ominus}$) (left) or ΔG (AH/ BH) (right).

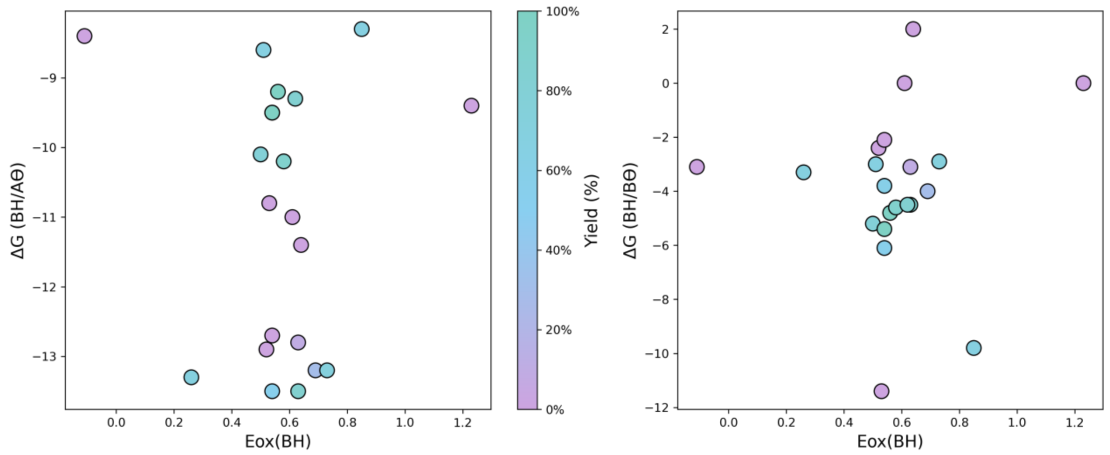

Figure TS37. Distribution of products yield (color scale) at functions of E_{ox} (BH), and ΔG ($A^{\ominus}$ /$B^{\ominus}$) (left) or ΔG ($A^{\ominus}$ / $C^{\ominus}$) (right).

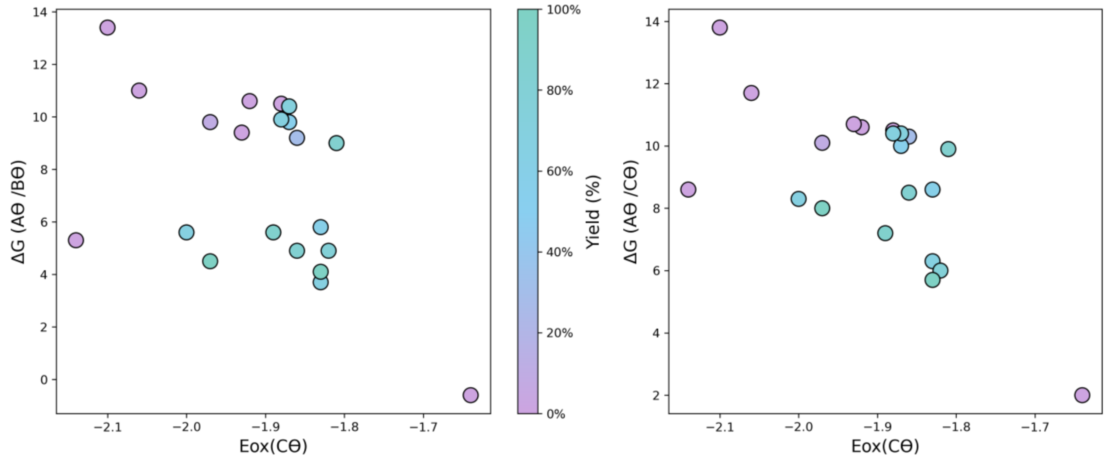

Figure TS38. Distribution of products yield (color scale) at functions of E_{ox} ($C^{\ominus}$), and ΔG ($A^{\ominus}$ /$B^{\ominus}$) (left) or ΔG ($A^{\ominus}$ / $C^{\ominus}$) (right).

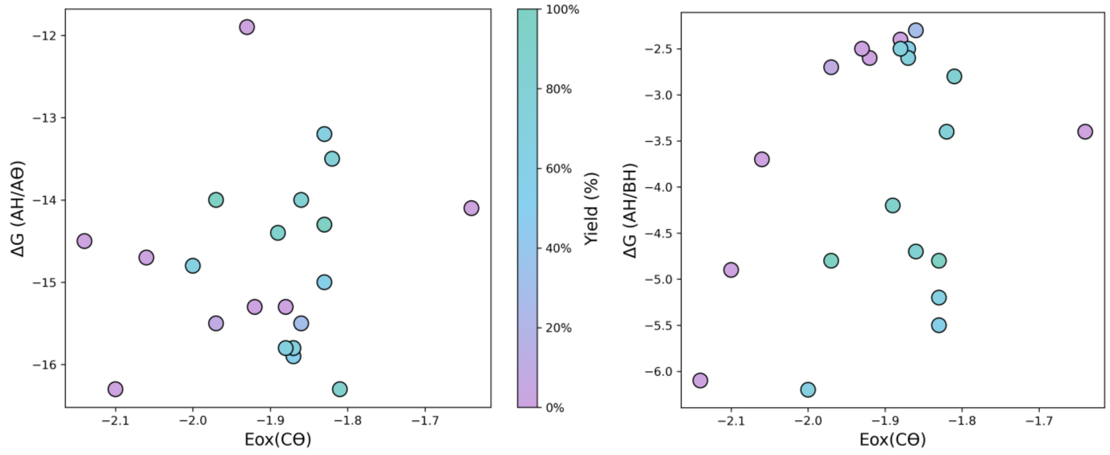

Figure TS39. Distribution of products yield (color scale) at functions of E_{ox} ($C^{\ominus}$), and ΔG (AH /$A^{\ominus}$) (left) or ΔG (AH/ BH) (right).

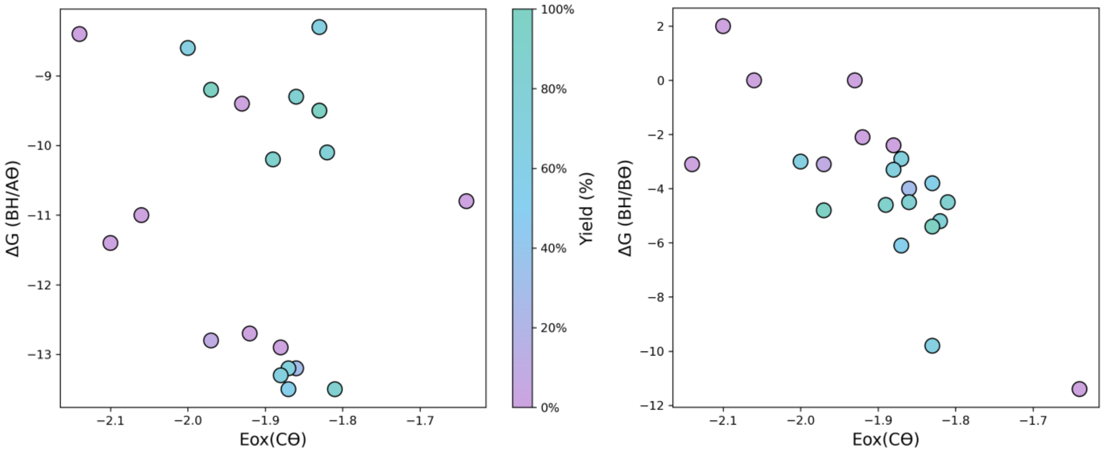

Figure TS40. Distribution of products yield (color scale) at functions of $E_{ox}(C^{\ominus})$, and ΔG (BH /$A^{\ominus}$) (left) or ΔG ($BH^{\ominus}$ / $B^{\ominus}$) (right).

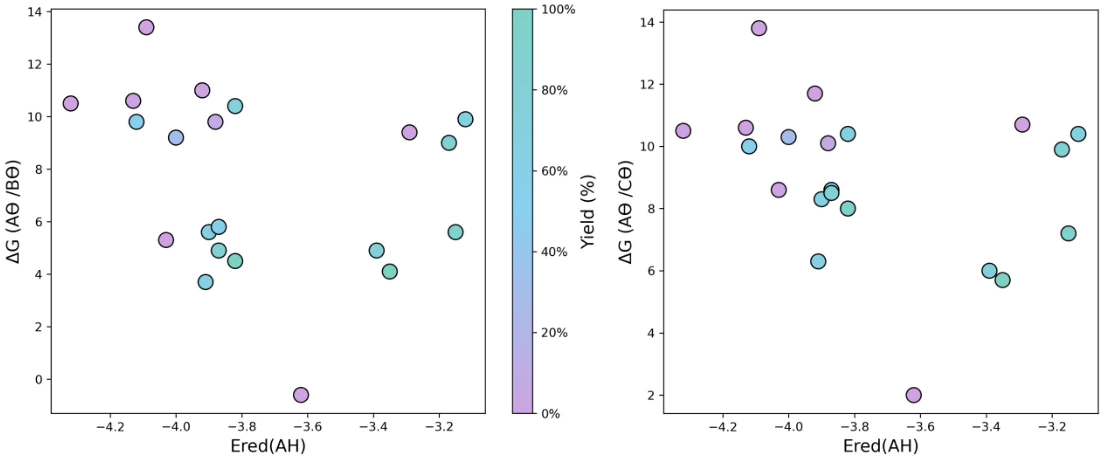

Figure TS41. Distribution of products yield (color scale) at functions of $E_{red}(AH)$, and ΔG ($A^{\ominus}$ /$B^{\ominus}$) (left) or ΔG ($A^{\ominus}$ / $C^{\ominus}$) (right).

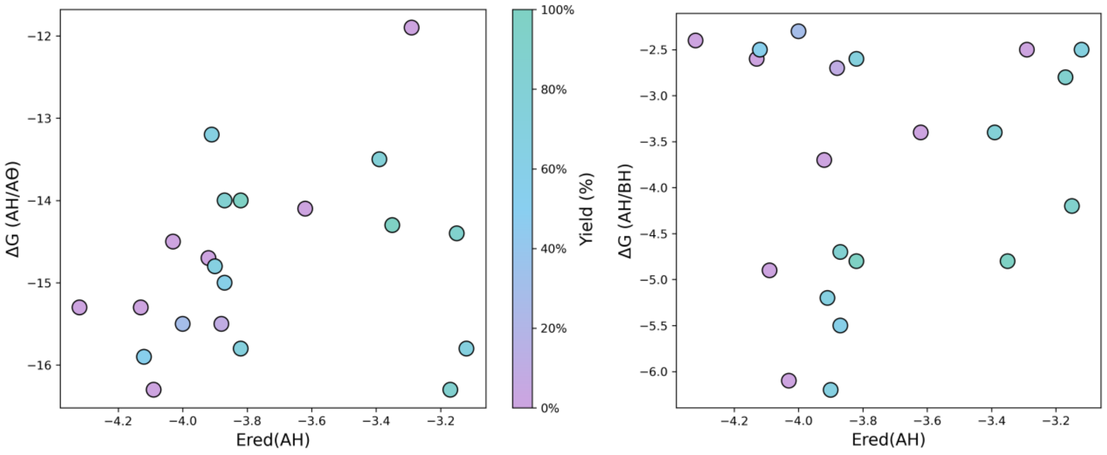

Figure TS42. Distribution of products yield (color scale) at functions of $E_{red}(AH)$, and ΔG (AH /$A^{\ominus}$) (left) or ΔG (AH/ BH) (right).

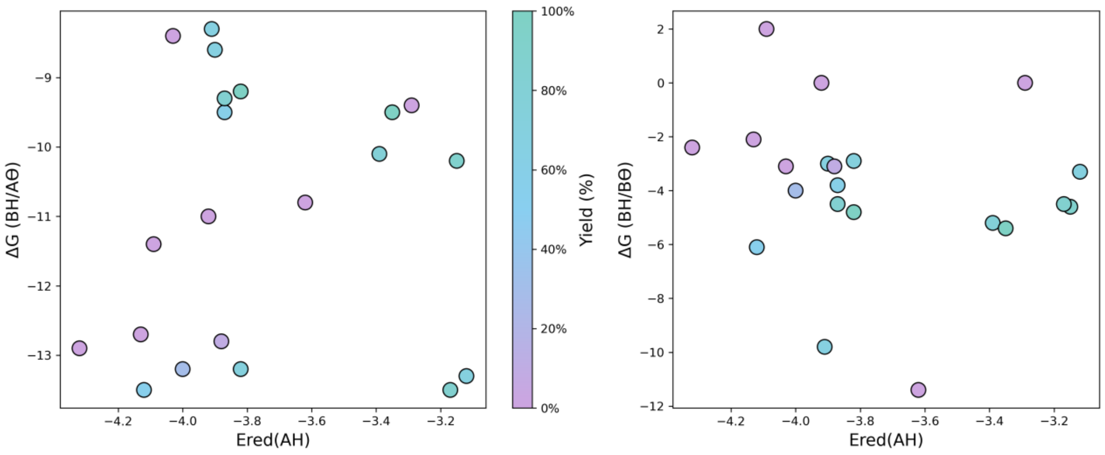

Figure TS43. Distribution of products yield (color scale) at functions of E_{red}(AH), and ΔG (BH /A$^{\ominus}$) (left) or ΔG (BH$^{\ominus}$ / B$^{\ominus}$) (right).

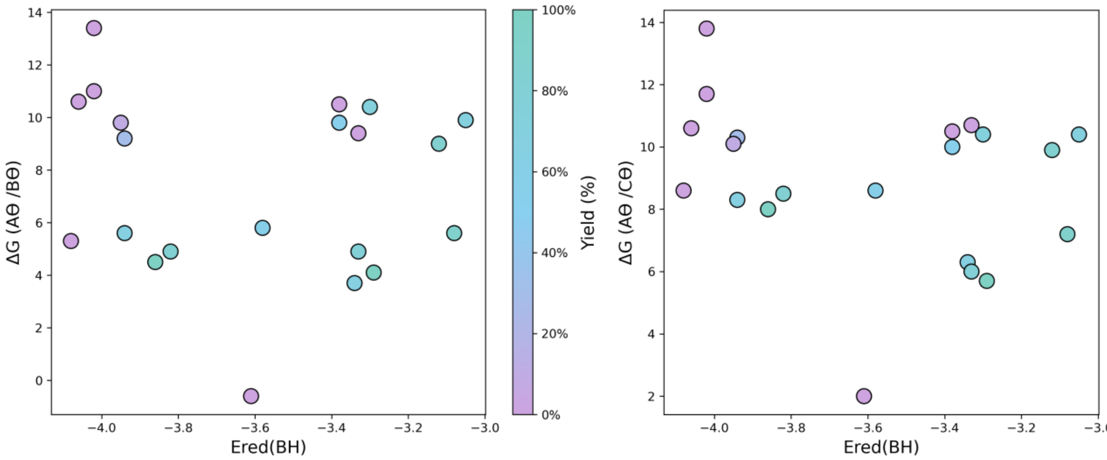

Figure TS44. Distribution of products yield (color scale) at functions of E_{red}(BH), and ΔG (BH /A$^{\ominus}$) (left) or ΔG (BH$^{\ominus}$ / B$^{\ominus}$) (right).

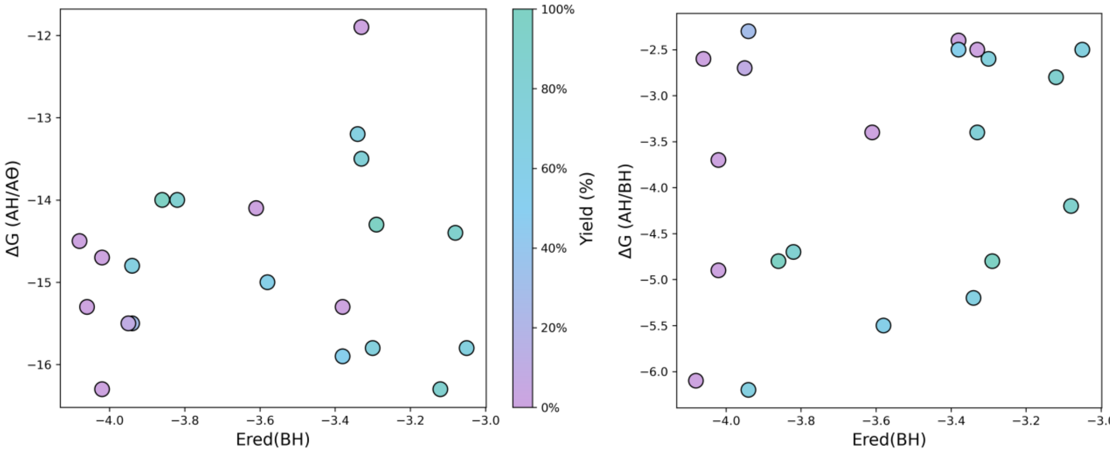

Figure TS45. Distribution of products yield (color scale) at functions of E_{red}(BH), and ΔG (AH /A$^{\ominus}$) (left) or ΔG (AH/ BH) (right).

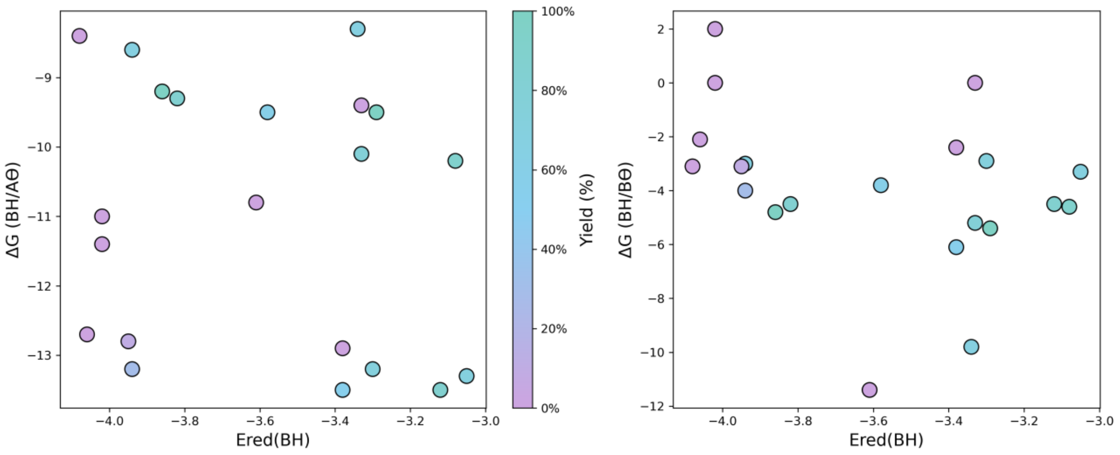

Figure TS46. Distribution of products yield (color scale) at functions of E_{red} (BH), and ΔG ($A^{\ominus}$ /$B^{\ominus}$) (left) or ΔG ($A^{\ominus}$ / $C^{\ominus}$) (right).

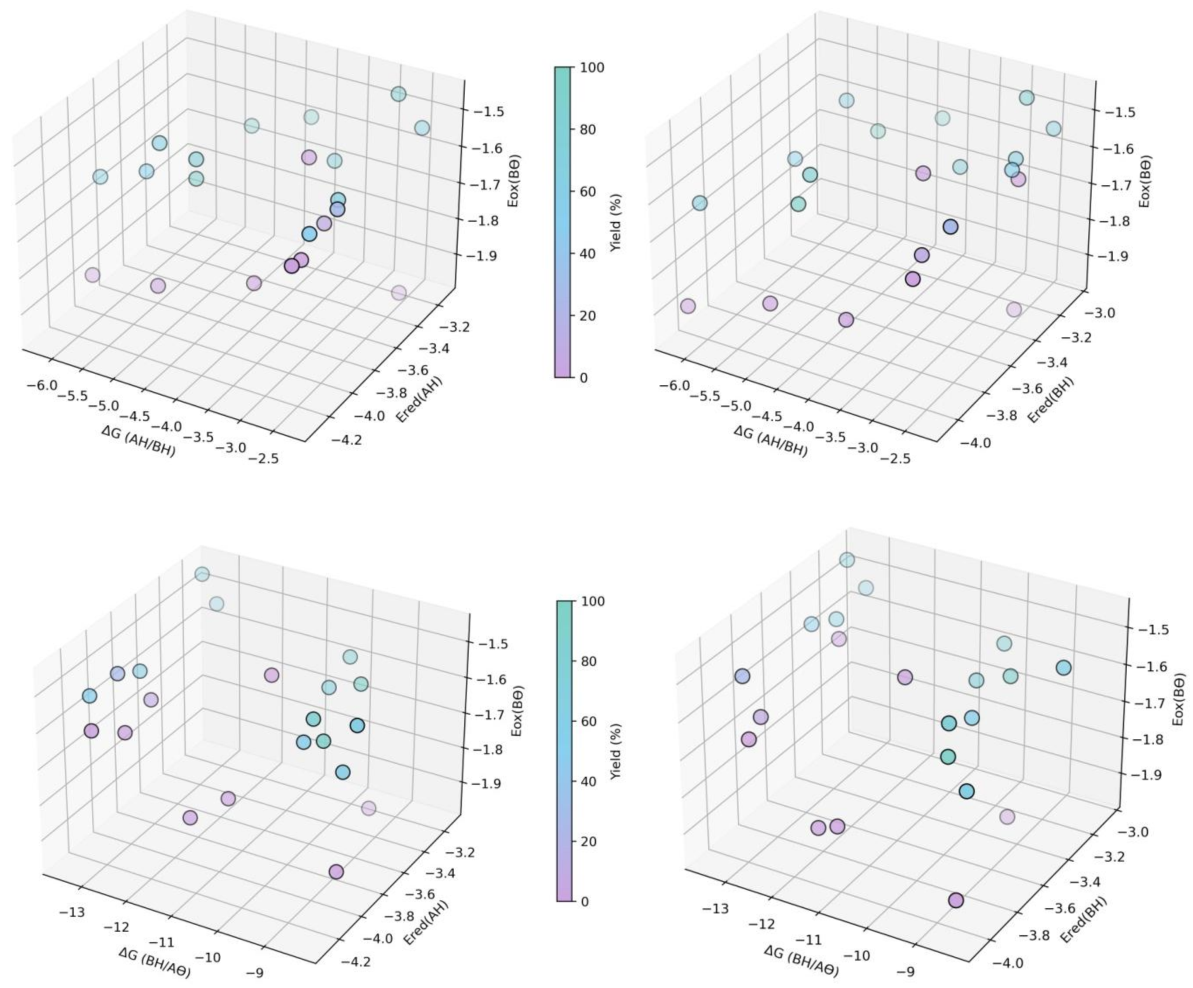

Figure TS47. Distribution of products yield (color scale) at functions of E_{red} ($B^{\ominus}$) and other two parameters.

7.5. Theoretical considerations

Given the scarcity of mechanistic insight in the literature on the topic of the propargyl–allene isomerization and the experimentally observed dependence of the reaction outcome on the applied current, we performed density functional theory (DFT) calculations at the ωB97X-D/def2-TZVP/def2-SVP level of theory (for details, see section: *General Information*) to elucidate the role of the base-generation rate and define the thermodynamic parameter space for our scope. In line with previous work supporting thermodynamic control in studied isomerizations,[19] we focused on free energy differences and calculated redox potentials of stationary points rather than on transition-state analysis, which has been reported to overestimate proton transfer barriers in cases of co-solvent participation in proton transfer mechanism.[20] We considered simplified mechanistic pathway (Figure S48) for model substrate 1a (denoted AH). Deprotonation of AHat the terminal (≡CH) group by t-BuO$^-$ to give the acetylide anion A^- is strongly exergonic (-14.3 kcal mol^{-1}). Subsequent single proton transfer from methylene unit (-CH_2-) of acetylide to formerly deprotonated carbonprovides a rearranged to higher energetically propargylic anionic intermediate C^- (+5.7 kcal mol^{-1}), which can evolve to allenyl anion B^- (-1.7 kcal mol^{-1} relative to C^-) by next proton transfer. Final protonation of B^- affords the neutral allene BH (5.5 kcal mol^{-1} relative to B^-, -4.8 kcal mol^{-1} relative to AH). Within this model, the initially formed acetylideanion A^- is the thermodynamically most stable species along the computed profile. However, as the reaction is conducted in MeCN/tBuOH (9:1), the protic co-solvent is expected to shift equilibria toward the lowest energetically neutral species **BH** (-4.8 kcal/mol relative to **AH**). To confirm a final steady state at our simplified model and evaluate how kinetic factors - particularly the mode of base generation - may influence the distribution of intermediates under these thermodynamic constraints, kinetic zero-dimensional simulations were performed using COPASI. Because transition states were not explicitly located, a sensitivity-analysis-type approach was adopted in which forward rate constants for exergonic steps were varied over several orders of magnitude. In particular, the rate constant for deprotonation and isomerization were explored to mimic different possible scenarios; reverse rate constants were derived from the calculated free energy differences (for details, see section: *Kinetic simulations for isomerization of 1b*). Across all tested models, the final equilibrium composition remained largely invariant, giving BH as the major species. However, the transient concentration of the acetylide anion A^- was found to depend strongly on the assumed reactions rate constants (Figure 49). Simulations in which t-BuO$^-$ was present at high initial concentration resulted in pronounced, short-lived accumulation of A (Figure 50), whereas continuous low-rate generation of base led to substantially lower steady-state concentrations of this intermediate. lowest energetically neutral speciesspecies BH (-4.8 kcal/mol relative to AH).

Figure S48. Energetic profile of proposed pathway of reaction

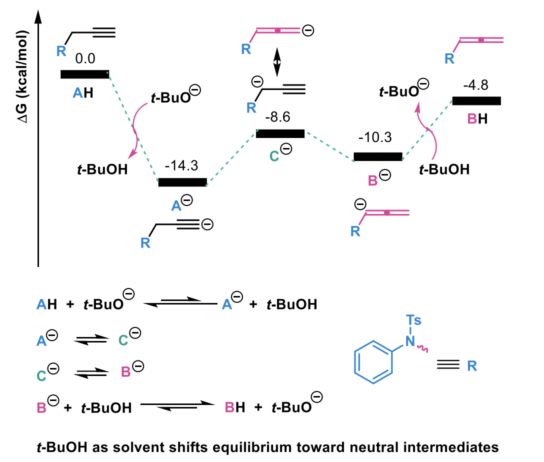

Our minimal kinetic model, together with observed impact of rate of tert-BuO$^-$ production suggests that elevated transient concentrations of anionic species may promote competitive reaction, such as anion oxidation, polymerization or other unproductive side reactions. Finally, correlations between the calculated thermodynamic and redox parameters and the experimental yields for 20

Figure 49. Impact of kinetic constants of isomerizations on concentration profile of A.

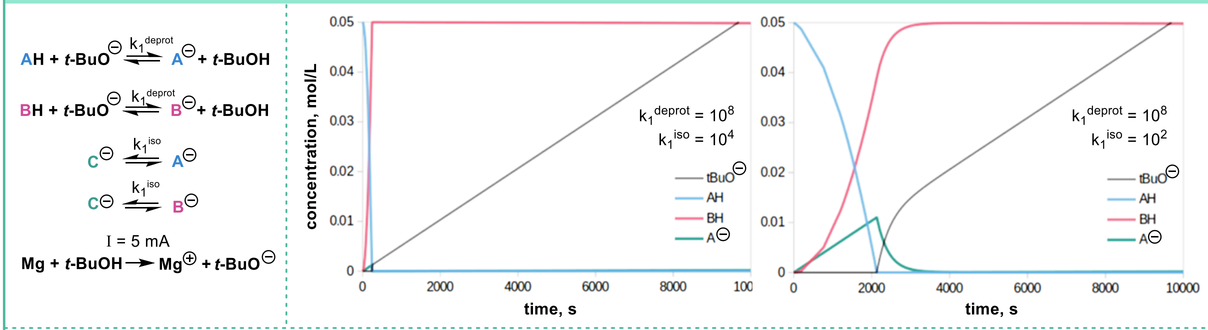

The kinetic simmulation of zero-dimensional minimal model based on thermodynamic control (for details, see SI 2) does not aim to predict time of reaction.

Figure S50. Comparision of kinetic profiles with selected amount of t-BuO⊖

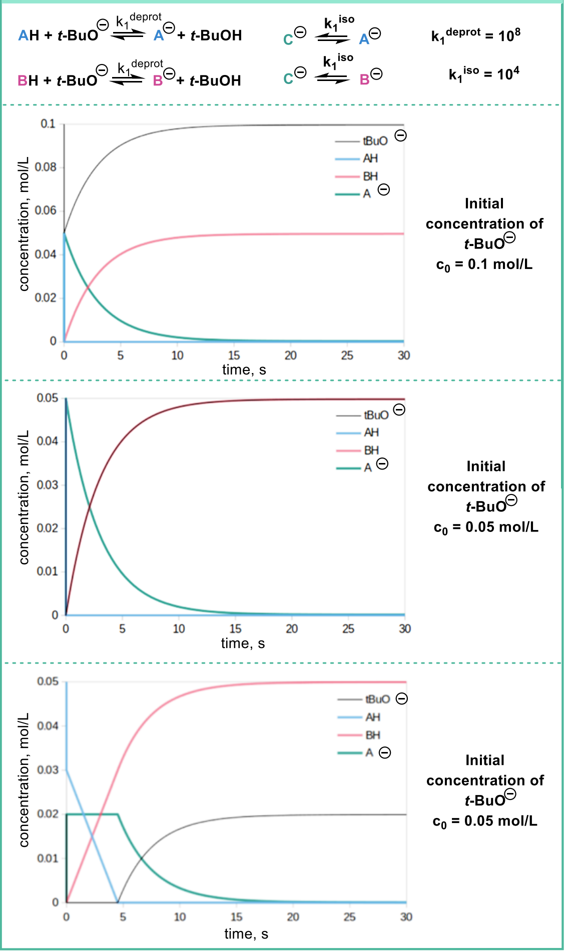

Figure S51. Energetic profile of C-O bond cleavage.

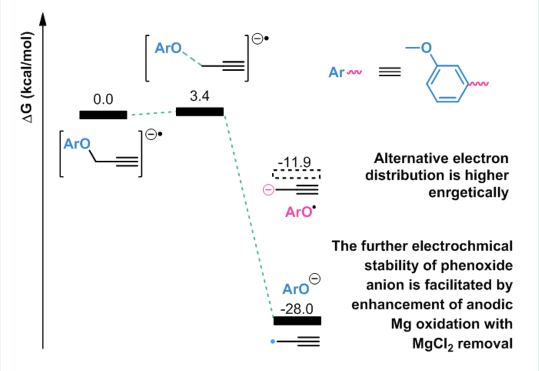

selected substrates were explored through multidimensional scatter analyses (for details, see SI 2). The analysis had only exploratory character due to too low number of points to build full statistical model. We generalized the notation presented for 1a (Figure 5) to all substrates, using AH, A^-, C^-, B^-, BH as the general names of stationary points. The most productive substrates cluster within a well-defined parameter space is one where the axis corresponds to: free energy of BH dissociation to A^-, potential of AH reduction and redox potential of B^- (Figure 8). As far as, concentration of B^- was close to zero at all of our simulation, the additional driving force coming from its depletion, before its protonation, could become increasingly relevantfor a model as the instantaneous concentration of the anionic intermediate, such as A^-, rises. The next descriptor namely potential of AH reduction can result from competition of desired isomerization and various reduced substrate decomposition pathways. In case of phenolic ethersthe reduction of AH intermediate can be followed by depropargylation reaction.

In this context we conducted an investigation of the depropargylation observed experimentally at elevated applied currents, focusing on the most relevant mechanistic step that is C-O bond cleavage (Figure 9). Computational studies on 1r indicate that the pathway proceeding via single-electron reduction to the propargyl radical anion, followed by formation of the phenoxide anion and the propargyl radical, features a low energy barrier (3.4 kcal/mol) and is highly exergonic (-28.0 kcal/mol). During our experiments produced phenoxide anion appeared to be enough electrochemically stable to be isolated as phenol after reaction quenching. The enhancement of anodic

magnesium oxidation by precipitation of produced Mg^{2+} cations with chloride anions in solution was efficient enough to prevent possible oxidative phenoxide decomposition. An alternative mechanism of propargyl group removal starting from substrate oxidized to radical cation was also considered but was found to be energetically disfavored (for details, see section *Additional Computational data*).

7.6. Thermodynamic parameters

Table TS1. Equilibrium reactions Gibbs free energies (in kcal/mol)calculatated with ωB97XD/Def2-TZVP/Def-2SVP level of theory.

	ΔG (AH/BH)	ΔG (AH/A$^{\ominus}$)	ΔG (BH/B$^{\ominus}$)	ΔG (BH/A$^{\ominus}$)	ΔG (A$^{\ominus}$ /B$^{\ominus}$)	ΔG (A$^{\ominus}$ /C$^{\ominus}$)	ΔG (B$^{\ominus}$ /C$^{\ominus}$)
**1a**	-4.8	-14.3	-5.4	-9.5	4.1	5.7	1.7
**1b**	-4.8	-14.0	-4.8	-9.2	4.5	8.0	3.5
**1c**	-6.2	-14.8	-3.0	-8.6	5.6	8.3	2.7
**1d**	-5.5	-15.0	-3.8	-9.5	5.8	8.6	2.8
**1e**	-4.2	-14.4	-4.6	-10.2	5.6	7.2	1.6
**1f**	-6.1	-14.5	-3.1	-8.4	5.3	8.6	3.3
**1g**	-3.4	-13.5	-5.2	-10.1	4.9	6.0	1.1
**1h**	-5.2	-13.2	-9.8	-8.3	3.7	6.3	2.6
**1i**	-2.8	-16.3	-4.5	-13.5	9.0	9.9	0.9
**1j**	-2.6	-15.8	-2.9	-13.2	10.4	10.4	0.0
**1k**	-2.5	-15.9	-3.6	-13.5	9.8	10.0	0.1
**1l**	-2.5	-15.8	-3.3	-13.3	9.9	10.4	0.5
**1m**	-3.7	-14.7	0.0	-11.0	11.0	11.7	0.7
**1n**	-4.9	-16.3	-2.0	-11.4	13.4	13.8	0.4
**1o**	-2.5	-11.9	0.0	-9.4	9.4	10.7	1.4
**1r**	-2.7	-15.5	-3.1	-12.8	9.8	10.1	0.3
**1s**	-3.4	-14.1	-11.4	-10.8	-0.6	2.0	2.6
**1t to 2t**	-2.3	-15.5	-4.0	-13.2	9.2	10.3	1.1
**1u to 2u**	-4.7	-14.0	-4.5	-9.3	4.9	8.5	3.7
**1u to 2u’**	-2.4	-15.6	-3.4	-13.2	9.9	10.2	0.4
**2u to 2uu**	-2.6	-15.9	-3.4	-13.3	9.9	10.3	0.4
**2u’ to 2uu**	-2.6	-11.5	-4.0	-9.0	5.0	5.7	0.7
**S14**	-2.6	-15.3	-2.1	-12.7	10.6	10.6	0.0

Table TS2. Electrochemical redox potential calculated with ωB97XD/Def2-TZVP/Def-2SVP level of theory.

	E_{ox}(AH)	E_{ox}(BH)	E_{red}(AH)	E_{red}(BH)	E_{ox}($A^{⊖}$)	E_{ox}($B^{⊖}$)	E_{ox}($C^{⊖}$)
**1a**	0.90	0.54	-3.35	-3.29	0.54	-1.59	-1.83
**1b**	0.92	0.56	-3.82	-3.86	0.52	-1.61	-1.97
**1c**	0.77	0.51	-3.90	-3.94	0.52	-1.65	-2.00
**1d**	0.87	0.63	-3.88	-3.95	0.54	-1.61	-1.97
**1e**	0.83	0.58	-3.15	-3.08	0.83	-1.59	-1.89
**1f**	0.06	-0.11	-4.03	-4.08	-0.16	-1.88	-2.14
**1g**	1.37	0.5	-3.39	-3.33	0.51	-1.61	-1.82
**1h**	0.41	0.40	-3.70	-3.77	0.31	-1.71	-1.95
**1i**	0.57	0.63	-3.17	-3.12	0.70	-1.46	-1.81
**1j**	0.76	0.73	-3.82	-3.30	0.52	-1.56	-1.87
**1k**	0.94	0.85	-3.91	-3.34	0.70	-1.51	-1.83
**1l**	0.21	0.26	-3.12	-3.05	0.08	-1.55	-1.88
**1m**	1.19	0.61	-3.92	-4.02	0.55	-1.81	-2.06
**1n**	1.16	0.64	-4.09	-4.02	0.59	-1.83	-2.10
**1o**	2.21	1.23	-3.29	-3.33	0.40	-1.96	-1.93
**1p**	0.49	0.52	-4.32	-3.38	0.33	-1.58	-1.88
**1r**	0.49	0.54	-4.12	-3.38	0.31	-1.56	-1.87
**1s**	0.75	0.53	-3.62	-3.61	0.35	-1.54	-1.64
**1t to 2t**	0.68	0.69	-4.00	-3.94	0.45	-1.52	-1.86
**1u to 2u**	0.60	0.54	-3.87	-3.58	0.24	-1.61	-1.83
**1u to 2u’**	0.60	0.62	-3.87	-3.82	1.11	-1.54	-1.86
**2u to 2uu**	0.54	0.55	-3.58	-3.46	0.39	-1.53	-1.81
**2u’ to 2uu**	0.54	0.55	-3.58	-3.46	0.51	-1.63	-1.84
**S14**	0.51	0.54	-4.13	-4.06	0.28	-1.63	-1.92

Table TS3. Comparison of relative energies obtained with different levels of theory.

**Substrate**	Parameter	ωB97XD/Def2-TZVP/Def-2SVP	ωB97XD/Def2-TZVP	ωB97XD/Def2-TZVPP/Def-2TZVP	DLPNO-CCSD(T) – CBS
**1b**	ΔG (AH/BH)	-4.8	-4.9	-4.9	-1.7
	ΔG (AH/$A^{⊖}$)	-14.0	-14.4	-15.0	-14.2
	ΔG (BH/$B^{⊖}$)	-4.8	-4.8	-5.3	-4.9
	ΔG (BH/$A^{⊖}$)	9.2	9.5	10.1	12.5
	ΔG ($A^{⊖}$ /$B^{⊖}$)	4.5	4.7	4.7	7.6
	ΔG ($A^{⊖}$ /$C^{⊖}$)	8.0	7.7	7.8	11.1
	ΔG ($B^{⊖}$ /$C^{⊖}$)	3.5	3.0	3.0	3.5
**1n**	ΔG (AH/BH)	-4.9	-4.7	-4.7	-1.2
	ΔG (AH/$A^{⊖}$)	-16.3	-14.8	-15.4	-14.4
	ΔG (BH/$B^{⊖}$)	2.0	1.6	1.1	2.0
	ΔG (BH/$A^{⊖}$)	11.4	10.1	10.6	13.1
	ΔG ($A^{⊖}$ /$B^{⊖}$)	13.4	11.7	11.8	15.1
	ΔG ($A^{⊖}$ /$C^{⊖}$)	13.8	12.1	12.1	15.4
	ΔG ($B^{⊖}$ /$C^{⊖}$)	0.4	0.4	0.4	0.3
**1k**	ΔG (AH/BH)	-2.5	-2.2	-2.1	1.3
	ΔG (AH/$A^{⊖}$)	-15.9	-16.2	-16.7	-15.9
	ΔG (BH/$B^{⊖}$)	-3.6	-4.0	-4.6	-3.3
	ΔG (BH/$A^{⊖}$)	13.5	14.0	14.6	17.2
	ΔG ($A^{⊖}$ /$B^{⊖}$)	9.8	10.0	10.0	13.9
	ΔG ($A^{⊖}$ /$C^{⊖}$)	10.0	10.2	10.2	13.9

	ΔG ($B^{\ominus}$ /$C^{\ominus}$)	0.1	0.2	0.2	0.0

Table TS4. Comparison of electrochemical potentials obtained with different levels of theory.

Substrate	Parameter	ωB97XD/Def2-TZVP/Def-2SVP	ωB97XD/Def2-TZVP	ωB97XD/Def2-TZVPP/Def-2TZVP	DLPNO-CCSD(T) – CBS
**1b**	E_{ox}(AH)	0.92	0.96	1.00	1.61
	E_{ox}(BH)	0.56	0.59	0.64	1.29
	E_{red}(AH)	-3.82	-3.82	-3.80	-3.34
	E_{red}(BH)	-3.86	-3.88	-3.83	-3.34
	E_{ox}($A^{\ominus}$)	0.52	0.54	0.58	1.30
	E_{ox}($B^{\ominus}$)	-1.61	-1.55	-1.51	-0.92
	E_{ox}($C^{\ominus}$)	-1.97	-1.94	-1.90	-1.38
**1n**	E_{ox}(AH)	1.16	1.20	1.24	1.90
	E_{ox}(BH)	0.64	0.67	0.71	1.42
	E_{red}(AH)	-4.09	-4.05	-4.01	-3.53
	E_{red}(BH)	-4.02	-4.05	-4.00	-3.51
	E_{ox}($A^{\ominus}$)	0.59	0.54	0.59	1.28
	E_{ox}($B^{\ominus}$)	-1.83	-1.80	-1.76	-1.16
	E_{ox}($C^{\ominus}$)	-2.10	-2.08	-2.04	-1.49
**1k**	E_{ox}(AH)	0.94	0.98	1.02	1.72
	E_{ox}(BH)	0.85	0.85	0.89	1.66
	E_{red}(AH)	-3.91	-3.94	-3.90	-3.37
	E_{red}(BH)	-3.34	-3.31	-3.27	-2.82
	E_{ox}($A^{\ominus}$)	0.70	0.74	0.78	1.47
	E_{ox}($B^{\ominus}$)	-1.51	-1.49	-1.44	-0.85
	E_{ox}($C^{\ominus}$)	-1.27	-1.25	-1.21	-1.22

7.7. Additional computational data

We propose that aryl propargyl ethers undergo depropargylation via a reductive C–O bond cleavage pathway (Figure TS52).

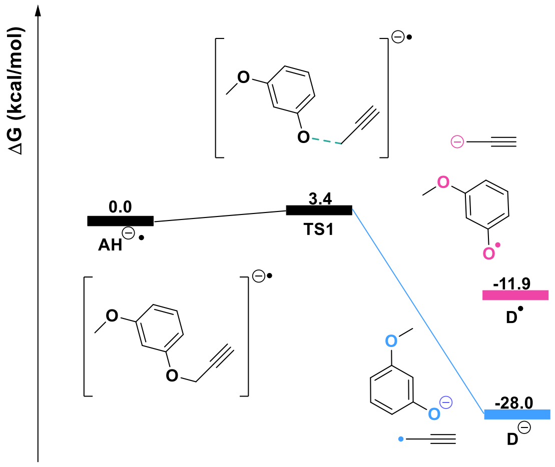

Figure TS52. Energetic profile of the depropargylationreaction step via reductive C–O bond cleavage for 1r .

The oxidative C–O bond cleavage pathway was also considered but the calculations revealed the reaction to be energetically disfavoured and reverse reaction to be barierless (Figure TS53).

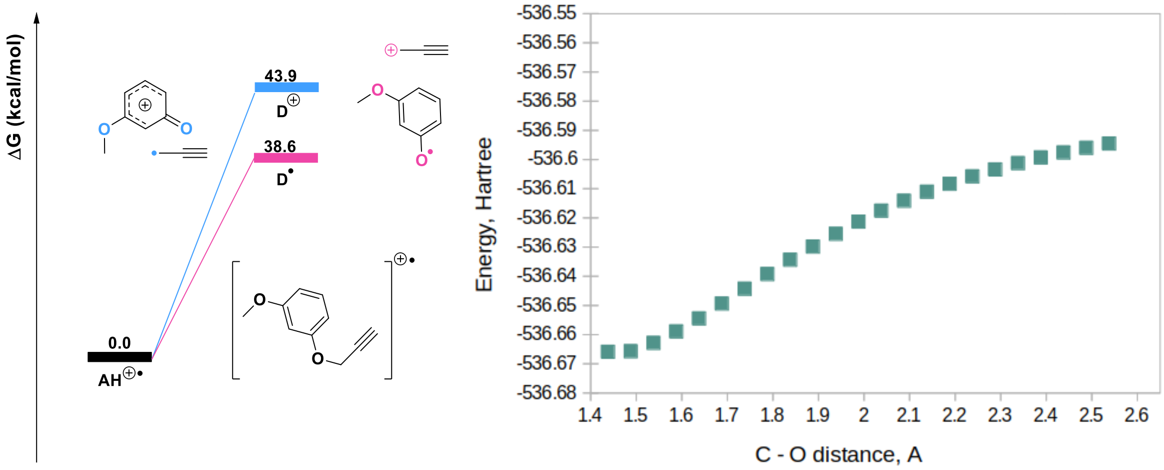

Figure TS53. Energetic profile of oxidative C-O bond cleavage (ωB97XD/ Def-2TZVP) and electronic-energy scans for C-O bond cleavage (ωB97XD/ Def-2SVP) for 1r.

7.8. References

[1] M. J. Frisch, G. W. Trucks, H. B. Schlegel, G. E. Scuseria, M. A. Robb, J. R. Cheeseman, G. Scalmani, V. Barone, G. A. Petersson, H. Nakatsuji, X.- S. Li, M. Caricato, A. V. Marenich, J. Bloino, B. G. Janesko, R. Gomperts, B. Mennucci, H. P. Hratchian, J. V. Ortiz, A. F. Izmaylov, J. L. Sonnenberg, D. Williams-Young, F. Ding, F. Lipparini, F. Egidi, J. Goings, B. Peng, A. Petrone, T. Henderson, D. Ranasinghe, V. G. Zakrzewski, J. Gao, N. Rega, G. Zheng, W. Liang, M. Hada, M. Ehara, K. Toyota, R. Fukuda, J. Hasegawa, M. Ishida, T. Nakajima, Y. Honda, O. Kitao, H. Nakai, T. Vreven, K. Throssell, J. A. Montgomery, Jr., J. E. Peralta, F. Ogliaro, M. J. Bearpark, J. J. Heyd, E. N. Brothers, K. N. Kudin, V. N. Staroverov, T. A. Keith, R. Kobayashi, J. Normand, K. Raghavachari, A. P. Rendell, J. C. Burant, S. S. Iyengar, J. Tomasi, M. Cossi, J. M. Millam, M. Klene, C. Adamo, R. Cammi, J. W. Ochterski, R. L. Martin, K. Morokuma, O. Farkas, J. B. Foresman, D. J. Fox, Gaussian 16 Rev. C.02, Gaussian, Inc., Wallingford CT, **2019**.

[2] J.-D. Chai, M. Head-Gordon, *Phys. Chem. Chem. Phys.* **2008,** *10*, 6615-6620.

[3] M. V. Marenich, C. J. Cramer, D. G. Truhlar, *J. Phys. Chem. B* **2009**, 113, 6378-6396.

[4] D. Weigend, R. Ahlrichs, *Phys. Chem. Chem. Phys.* **2005**, *7*, 3297-3305.

[5] S. Grimme, *Chem. Eur. J.* **2012**, *18*, 9955-9964.

[6] G. Luchini, J. V. Alegre-Requena, I. Funez-Ardoiz, R. S. Paton F1000Research, **2020**, 9, 291.GoodVibes version 3.2 (DOI: 10.12688/f1000research.22758.1).

[7] O. Hammerich, B. Speiser, Eds. *Organic Electrochemistry Revised and Expanded. Chapter 6. Theoretical Calculation Of Reduction Potentials* **2015**, DOI 10.1201/b19122.

[8] a) C. Riplinger, F. Neese, *J. Chem. Phys*. **2013**, *138*, 034106; b) C. Riplinger, B. Sandhoefer, A. Hansen, F. Neese, *J. Chem. Phys*. **2013**, *139*, 134101; c) C. Riplinger, P. Pinski, U. Becker, E. F. Valeev, F. Neese, *J. Chem. Phys*. **2016**, *144*, 024109.

[9] F. Neese, *WIREs Comput Mol Sci.* **2022**, *12*, e1606.

[10] B. Helmich-Paris, B. de Souza, F. Neese, R. Izsák, *J. Chem. Phys.* **2021**, *155*, 104109.

[11] F. Weigend, *Phys. Chem. Chem. Phys*. **2006**, *8*, 1057-1065.

[12] F. Weigend, *J. Comput. Chem.* **2008**, *29*, 167-175.

[13] A. Hellweg, C. Hattig, S. Hofener and W. Klopper, *Theor. Chem. Acc.* **2007**, *117*, 587- 597.

[14] S. Zhong, E. C. Barnes, G. A. Petersson, *J. Chem. Phys.* **2008**, *129*, 184116

[15] T. Helgaker, W. Klopper, H. Koch, J. Noga, *J. Chem. Phys.* **1997**, *106*, 9639-9646.

[16] F. Neese, E. F. Valeev, *J. Chem. Theory Comput*. **2011**, *7*, 33-43.

[17] S. A. De Marothy, XYZViewer version 0.97, Stockholm, **2010**.

[18] S. Hoops, S. Sahle, R. Gauges, C. Lee, J. Pahle, N. Simus, M. Singhal, L. Xu, P. Mendes, U. Kummer *Bioinformatics* **2006**, *22*, 3067-74

[19] A. Navarro-Vázquez *Beilstein J. Org. Chem.***2015**, *11*, 1441–1446.

[20] R. E. Plata, D. A. Singleton *J. Am. Chem. Soc.* **2015** *137*,3811-3826

7.9. Energies and Energy Corrections of Stationary Points(in atomic units)

Table TS5. Energies and energy corrections of stationary points at 1a isomerization reaction.

Stationary point	ωB97XD/Def2-SVP (MeCN)	ωB97XD/Def2-TZVP (MeCN) single-point	Thermal correction to Gibbs free energy at 25 °C
**AH**	-1221.013682	-1222.096851	0.233912
**A**	-1220.498094	-1221.588356	0.223285
**C**	-1220.489166	-1221.575694	0.219751
**B**	-1220.492290	-1221.579390	0.220815
**BH**	-1221.020344	-1222.103062	0.232463
$\mathbf{AH}^{\oplus\bullet}$	-1220.792038	-1221.873795	0.233186
$\mathbf{AH}^{\ominus\bullet}$	-1221.020344	-1222.157451	0.232463
$\mathbf{BH}^{\oplus\bullet}$	-1220.811899	-1221.892203	0.230541
$\mathbf{BH}^{\ominus\bullet}$	-1221.084066	-1222.166143	0.227429
$\mathbf{A}^{\bullet}$	-1220.292210	-1221.376018	0.220819
$\mathbf{B}^{\bullet}$	-1220.363807	-1221.445156	0.217284
$\mathbf{C}^{\bullet}$	-1220.371107	-1221.453120	0.219118

Table TS6. Energies and energy corrections of stationary points at 1b isomerization reaction.

Stationary point	ωB97XD/Def2-SVP (MeCN) optimization	ωB97XD/Def2-TZVP (MeCN) single-point	Thermal correction to Gibbs free energy at 25 °C
**AH**	-555.142900	-555.742454	0.159272
**A**	-554.625617	-555.233087	0.148223
**C**	-554.613850	-555.217578	0.145466
**B**	-554.618883	-555.222837	0.145075
**BH**	-555.151147	-555.749456	0.158571
$\mathbf{AH}^{\oplus\bullet}$	-554.919494	-555.519550	0.159293
$\mathbf{AH}^{\ominus\bullet}$	-555.151147	-555.782449	0.158571
$\mathbf{BH}^{\oplus\bullet}$	-554.939918	-555.537676	0.156448
$\mathbf{BH}^{\ominus\bullet}$	-555.183844	-555.785860	0.147518
$\mathbf{A}^{\bullet}$	-554.421468	-555.021292	0.144528
$\mathbf{B}^{\bullet}$	-554.492207	-555.090399	0.142617
$\mathbf{C}^{\bullet}$	-554.502416	-555.100508	0.144995

Table TS7. Comparision of energies of stationary points at 1b isomerization reaction calculated at different levels of theory.

Stationary point	ωB97XD/Def2-TZVP (MeCN) optimization	ωB97XD/Def2-TZVPP (MeCN) single-point	DLPNO-CCSD(T)/basis set limit (MeCN) single-point	Thermal correction to Gibbs free energy at 25°C
**AH**	-555.743894	-555.748490	-554.972003	0.153867
**A**	-555.234507	-555.238664	-554.472526	0.143137
**C**	-555.219083	-555.223217	-554.451793	0.140042
**B**	-555.224080	-555.228178	-554.457479	0.140218
**BH**	-555.750775	-555.755326	-554.973860	0.152936
**AH**$^{\oplus\bullet}$	-555.520901	-555.525593	-554.741314	0.154324
**AH**$^{\ominus\bullet}$	-555.750775	-555.787072	-555.012999	0.152936
**BH**$^{\oplus\bullet}$	-555.538957	-555.543558	-554.752636	0.151201
**BH**$^{\ominus\bullet}$	-555.787164	-555.791793	-555.013663	0.143609
**A**$^{\bullet}$	-555.022464	-555.026669	-554.248787	0.139224
**B**$^{\bullet}$	-555.091726	-555.095854	-554.318000	0.139028
**C**$^{\bullet}$	-555.101856	-555.106062	-554.330268	0.139637

Table TS8. Energies and energy corrections of stationary points at 1c isomerization reaction.

Stationary point	ωB97XD/Def2-SVP (MeCN)	ωB97XD/Def2-TZVP (MeCN) single-point	Thermal correction to Gibbs free energy at 25 °C
**AH**	-748.125472	-748.933470	0.244010
**A**	-747.608776	-748.424840	0.232537
**C**	-747.594899	-748.408280	0.229253
**B**	-747.598890	-748.412070	0.228667
**BH**	-748.133792	-748.941109	0.241831
**AH**$^{\oplus\bullet}$	-747.906345	-748.715304	0.243203
**AH**$^{\ominus\bullet}$	-748.133792	-748.969693	0.241831
**BH**$^{\oplus\bullet}$	-747.923588	-748.731312	0.239990
**BH**$^{\ominus\bullet}$	-748.164234	-748.975812	0.232259
**A**$^{\bullet}$	-747.405617	-748.214652	0.230740
**B**$^{\bullet}$	-747.475135	-748.282171	0.227181
**C**$^{\bullet}$	-747.484884	-748.292075	0.228684

Table TS9. Energies and energy corrections of stationary points at 1d isomerization reaction.

Stationary point	ωB97XD/Def2-SVP (MeCN)	ωB97XD/Def2-TZVP (MeCN) single-point	Thermal correction to Gibbs free energy at 25 °C
**AH**	-630.285393	-630.972331	0.163447
**A**	-629.769489	-630.464333	0.152259
**C**	-629.756082	-630.447894	0.149537
**B**	-629.759917	-630.451964	0.149095
**BH**	-630.294126	-630.980082	0.162486
$\mathbf{AH}^{\oplus\bullet}$	-630.064694	-630.751511	0.163736
$\mathbf{AH}^{\ominus\bullet}$	-630.294126	-631.009651	0.162486
$\mathbf{BH}^{\oplus\bullet}$	-630.081820	-630.767561	0.162206
$\mathbf{BH}^{\ominus\bullet}$	-630.325034	-631.015422	0.153746
$\mathbf{A}^{\bullet}$	-629.564166	-630.251563	0.148494
$\mathbf{B}^{\bullet}$	-629.635270	-630.320489	0.147641
$\mathbf{C}^{\bullet}$	-629.645013	-630.330733	0.148941

Table TS10. Energies and energy corrections of stationary points at 1e isomerization reaction.

Stationary point	ωB97XD/Def2-SVP (MeCN) optimization	ωB97XD/Def2-TZVP (MeCN) single-point	Thermal correction to Gibbs free energy at 25 °C
**AH**	-746.681411	-747.478215	0.210408
**A**	-746.164476	-746.969231	0.199218
**C**	-746.151721	-746.954108	0.195631
**B**	-746.155136	-746.956392	0.195316
**BH**	-746.688122	-747.483804	0.209295
$\mathbf{AH}^{\oplus\bullet}$	-746.460865	-747.257879	0.209762
$\mathbf{AH}^{\ominus\bullet}$	-746.688122	-747.547605	0.209295
$\mathbf{BH}^{\oplus\bullet}$	-746.476108	-747.271931	0.208040
$\mathbf{BH}^{\ominus\bullet}$	-746.755256	-747.555263	0.204704
$\mathbf{A}^{\bullet}$	-745.955267	-746.748337	0.1981408
$\mathbf{B}^{\bullet}$	-746.029259	-746.824652	0.1943930
$\mathbf{C}^{\bullet}$	-746.039144	-746.834633	0.1956900

Table TS11. Energies and energy corrections of stationary points at 1f isomerization reaction.

Stationary point	ωB97XD/Def2-SVP (MeCN)	ωB97XD/Def2-TZVP (MeCN) single-point	Thermal correction to Gibbs free energy at 25 °C
**AH**	-672.725610	-673.433154	0.229641
**A**	-672.208166	-672.923353	0.217437
**C**	-672.195153	-672.906929	0.214730
**B**	-672.202298	-672.912768	0.215332
**BH**	-672.735046	-673.441794	0.228545
**AH**⊕•	-672.532050	-673.241235	0.229103
**AH**⊖•	-672.735046	-673.465757	0.228545
**BH**⊕•	-672.548455	-673.256545	0.228552
**BH**⊖•	-672.762583	-673.473363	0.220953
**A**•	-672.024864	-672.740128	0.217369
**B**•	-672.084650	-672.791114	0.213654
**C**•	-672.089500	-672.796527	0.214913

Table TS12. Energies and energy corrections of stationary points at 1g isomerization reaction.

Stationary point	ωB97XD/Def2-SVP (MeCN)	ωB97XD/Def2-TZVP (MeCN) single-point	Thermal correction to Gibbs free energy at 25 °C
**AH**	-1029.472045	-1030.359462	0.183506
**A**	-1028.953909	-1029.849825	0.173118
**C**	-1028.945564	-1029.837065	0.169956
**B**	-1028.948263	-1029.838795	0.169893
**BH**	-1029.478681	-1030.365205	0.183812
**AH**⊕•	-1029.232768	-1030.117270	0.180857
**AH**⊖•	-1029.478681	-1030.419608	0.183812
**BH**⊕•	-1029.272215	-1030.155445	0.181505
**BH**⊖•	-1029.539670	-1030.426512	0.178187
**A**•	-1028.751636	-1029.639627	0.170976
**B**•	-1028.821697	-1029.706532	0.167504
**C**•	-1028.829088	-1029.714716	0.169681

Table TS13. Energies and energy corrections of stationary points at 1h isomerization reaction.

Stationary point	ωB97XD/Def2-SVP (MeCN)	ωB97XD/Def2-TZVP (MeCN) single-point	Thermal correction to Gibbs free energy at 25 °C
**AH**	-1225.031155	-1226.359140	0.390532
**A**	-1224.514369	-1225.849002	0.379689
**C**	-1224.502151	-1225.834572	0.375320
**B**	-1224.508568	-1225.839476	0.376010
**BH**	-1225.040332	-1226.366809	0.389985
**AH**$^{\oplus\bullet}$	-1224.825543	-1226.154952	0.390392
**AH**$^{\ominus\bullet}$	-1225.040332	-1226.405166	0.389985
**BH**$^{\oplus\bullet}$	-1224.834619	-1226.162506	0.389585
**BH**$^{\ominus\bullet}$	-1225.082613	-1226.411569	0.384247
**A**$^{\bullet}$	-1224.311290	-1225.647447	0.378597
**B**$^{\bullet}$	-1224.385138	-1225.710971	0.373930
**C**$^{\bullet}$	-1224.391926	-1225.718252	0.376338

Table TS14. Energies and energy corrections of stationary points at 1i isomerization reaction.

Stationary point	ωB97XD/Def2-SVP (MeCN)	ωB97XD/Def2-TZVP (MeCN) single-point	Thermal correction to Gibbs free energy at 25 °C
**AH**	-3149.255380	-3150.184134	0.145525
**A**	-3148.741943	-3149.677747	0.133907
**C**	-3148.725252	-3149.658517	0.130465
**B**	-3148.725849	-3149.658807	0.129316
**BH**	-3149.258937	-3150.187110	0.144050
**AH**$^{\oplus\bullet}$	-3149.043118	-3149.972803	0.144137
**AH**$^{\ominus\bullet}$	-3149.258937	-3150.251391	0.144050
**BH**$^{\oplus\bullet}$	-3149.044227	-3149.974139	0.143373
**BH**$^{\ominus\bullet}$	-3149.325936	-3150.256148	0.138503
**A**$^{\bullet}$	-3148.532437	-3149.459846	0.130715
**B**$^{\bullet}$	-3148.595141	-3149.658807	0.128771
**C**$^{\bullet}$	-3148.607827	-3149.535200	0.129734

Table TS15. Energies and energy corrections of stationary points at 1j isomerization reaction.

Stationary point	ωB97XD/Def2-SVP (MeCN)	ωB97XD/Def2-TZVP (MeCN) single-point	Thermal correction to Gibbs free energy at 25 °C
**AH**	-521.601236	-522.182365	0.104309
**A**	-521.086849	-521.675239	0.092659
**C**	-521.069376	-521.655339	0.089330
**B**	-521.070295	-521.655314	0.089257
**BH**	-521.604819	-522.185249	0.103074
**AH**$^{\oplus\bullet}$	-521.382291	-521.964787	0.103636
**AH**$^{\ominus\bullet}$	-521.604819	-522.223348	0.103074
**BH**$^{\oplus\bullet}$	-521.387106	-521.967800	0.101495
**BH**$^{\ominus\bullet}$	-521.662570	-522.248325	0.098425
**A**$^{\bullet}$	-520.877952	-521.465960	0.091625
**B**$^{\bullet}$	-520.943411	-521.522241	0.087814
**C**$^{\bullet}$	-520.954738	-521.534835	0.089233

Table TS16. Energies and energy corrections of stationary points at 1k isomerization reaction.

Stationary point	ωB97XD/Def2-SVP (MeCN)	ωB97XD/Def2-TZVP (MeCN) single-point	Thermal correction to Gibbs free energy at 25 °C
**AH**	-521.603204	-522.184200	0.104336
**A**	-521.089159	-521.677377	0.092848
**C**	-521.072066	-521.657871	0.089202
**B**	-521.073236	-521.658163	0.089327
**BH**	-521.606343	-522.186699	0.102901
**AH**$^{\oplus\bullet}$	-521.376854	-521.958989	0.102823
**AH**$^{\ominus\bullet}$	-521.606343	-522.220124	0.102901
**BH**$^{\oplus\bullet}$	-521.383397	-521.963596	0.100077
**BH**$^{\ominus\bullet}$	-521.662913	-522.248486	0.098310
**A**$^{\bullet}$	-520.872605	-521.460567	0.090886
**B**$^{\bullet}$	-520.944206	-521.522966	0.087744
**C**$^{\bullet}$	-520.955797	-521.535803	0.089054

Table TS17. Comparision of energies of stationary points at 1k isomerization reaction calculated at different levels of theory.

Stationary point	ωB97XD/Def2-TZVP (MeCN) optimization	ωB97XD/Def2-TZVPP (MeCN) single-point	DLPNO-CCSD(T)/basis set limit (MeCN) single-point	Thermal correction to Gibbs free energy at 25°C
**AH**	-522.185342	-522.184324	-521.526222	0.100137
**A**	-521.678473	-521.681010	-521.029134	0.089118
**C**	-521.658960	-521.661477	-521.003707	0.085840
**B**	-521.659229	-521.661688	-521.003659	0.085743
**BH**	-521.187839	-521.190751	-521.523219	0.099155
**AH**⊕•	-521.960114	-521.963116	-521.290191	0.099152
**AH**⊖•	-522.221317	-522.224314	-521.566823	0.092655
**BH**⊕•	-521.964710	-521.967670	-521.286692	0.095569
**BH**⊖•	-522.249673	-522.252600	-521.586807	0.094522
**A**•	-521.461616	-521.464175	-520.801377	0.087532
**B**•	-521.524117	-521.526617	-520.861480	0.084237
**C**•	-521.536986	-521.539555	-520.876359	0.085570

Table TS18. Energies and energy corrections of stationary points at 1l isomerization reaction.

Stationary point	ωB97XD/Def2-SVP (MeCN)	ωB97XD/Def2-TZVP (MeCN) single-point	Thermal correction to Gibbs free energy at 25 °C
**AH**	-959.427875	-960.466596	0.280539
**A**	-958.914159	-959.959636	0.269130
**C**	-958.896965	-959.939925	0.265983
**B**	-958.898057	-959.940070	0.265405
**BH**	-959.432184	-960.469776	0.279737
**AH**⊕•	-959.229720	-960.269877	0.280654
**AH**⊖•	-959.432184	-960.536180	0.279737
**BH**⊕•	-959.232170	-960.271253	0.279744
**BH**⊖•	-959.503447	-960.541770	0.274825
**A**•	-958.722016	-959.767985	0.269469
**B**•	-958.770698	-959.806720	0.264075
**C**•	-958.781885	-959.819125	0.265197

Table TS19. Energies and energy corrections of stationary points at 1m isomerization reaction.

Stationary point	ωB97XD/Def2-SVP (MeCN)	ωB97XD/Def2-TZVP (MeCN) single-point	Thermal correction to Gibbs free energy at 25 °C
**AH**	-461.740730	-462.240307	0.140067
**A**	-461.224006	-461.731501	0.128542
**C**	-461.205510	-461.710215	0.125863
**B**	-461.206480	-461.711070	0.125647
**BH**	-461.747046	-462.245370	0.139171
**AH**$^{\oplus\bullet}$	-461.501572	-462.002886	0.135689
**AH**$^{\ominus\bullet}$	-461.747046	-462.277414	0.139171
**BH**$^{\oplus\bullet}$	-461.533227	-462.032191	0.137538
**BH**$^{\ominus\bullet}$	-461.775628	-462.278315	0.130638
**A**$^{\bullet}$	-461.021554	-461.521482	0.127934
**B**$^{\bullet}$	-461.089808	-461.587253	0.124464
**C**$^{\bullet}$	-461.099639	-461.596897	0.126042

Table TS20. Energies and energy corrections of stationary points at 1n isomerization reaction.

Stationary point	ωB97XD/Def2-SVP (MeCN)	ωB97XD/Def2-TZVP (MeCN) single-point	Thermal correction to Gibbs free energy at 25 °C
**AH**	-540.294147	-540.876293	0.193780
**A**	-539.777193	-540.367100	0.182300
**C**	-539.756524	-540.344729	0.178944
**B**	-539.758844	-540.345960	0.179470
**BH**	-540.303265	-540.883681	0.193336
**AH**$^{\oplus\bullet}$	-540.057611	-540.641792	0.191052
**AH**$^{\ominus\bullet}$	-540.303265	-540.906435	0.193336
**BH**$^{\oplus\bullet}$	-540.088264	-540.669138	0.191431
**BH**$^{\ominus\bullet}$	-540.329151	-540.913979	0.184722
**A**$^{\bullet}$	-539.573670	-540.156410	0.179283
**B**$^{\bullet}$	-539.642772	-540.222355	0.177727
**C**$^{\bullet}$	-539.652652	-540.232897	0.179066

Table TS21. Energies and energy corrections of stationary points at 1o isomerization reaction.

Stationary point	ωB97XD/Def2-SVP (MeCN)	ωB97XD/Def2-TZVP (MeCN) single-point	Thermal correction to Gibbs free energy at 25 °C
**AH**	-1088.601976	-1089.562557	0.200430
**A**	-1088.082120	-1089.050263	0.189873
**C**	-1088.066270	-1089.031411	0.188151
**B**	-1088.065594	-1089.031937	0.186511
**BH**	-1088.605958	-1089.566093	0.199982
**AH**$^{\oplus\bullet}$	-1088.326847	-1089.286919	0.195191
**AH**$^{\ominus\bullet}$	-1088.605958	-1089.625793	0.199982
**BH**$^{\oplus\bullet}$	-1088.368743	-1089.328360	0.196743
**BH**$^{\ominus\bullet}$	-1088.668527	-1089.628630	0.195678
**A**$^{\bullet}$	-1087.883867	-1088.844652	0.188112
**B**$^{\bullet}$	-1087.954963	-1088.913667	0.185314
**C**$^{\bullet}$	-1087.951591	-1088.910821	0.185854

Table S22. Energies and energy corrections of stationary points at 1r isomerization reaction.

Stationary point	ωB97XD/Def2-SVP (MeCN)	ωB97XD/Def2-TZVP (MeCN) single-point	Thermal correction to Gibbs free energy at 25 °C
**AH**	-536.876737	-537.464864	0.143614
**A**	-536.362166	-536.957331	0.132078
**C**	-536.344742	-536.937649	0.128472
**B**	-536.345737	-536.937575	0.127864
**BH**	-536.880194	-537.467616	0.142128
**AH**$^{\oplus\bullet}$	-536.667199	-537.256176	0.142188
**AH**$^{\ominus\bullet}$	-536.880194	-537.494266	0.142128
**BH**$^{\oplus\bullet}$	-536.668937	-537.257348	0.140760
**BH**$^{\ominus\bullet}$	-536.935531	-537.528212	0.137907
**A**$^{\bullet}$	-536.160605	-536.755628	0.130799
**B**$^{\bullet}$	-536.218897	-536.804754	0.126936
**C**$^{\bullet}$	-536.229989	-536.817069	0.128153

Table TS23. Energies and energy corrections of stationary points at 1r depropargylation reaction.

Stationary point	ωB97XD/Def2-TZVP (MeCN) - optimization	Thermal correction to Gibbs free energy at 25 °C
**$AH^{\oplus\bullet}$**	-537.257470	0.137353
**$AH^{\ominus\bullet}$**	-537.517263	0.135122
**$D^{\bullet}$**	-421.382688	0.091917
**$D^{\ominus}$**	-421.535211	0.092652
**$D^{\oplus}$**	-421.159058	0.092279
≡—⊖	-116.129708	0.020752
≡—⊕	-115.788620	0.018794
≡—•	-116.002620	0.016066

Table TS24. Energies and energy corrections of stationary points at 1s isomerization reaction.

Stationary point	ωB97XD/Def2-SVP (MeCN)	ωB97XD/Def2-TZVP (MeCN) single-point	Thermal correction to Gibbs free energy at 25 °C
**AH**	-745.402408	-745.911597	0.108241
**A**	-744.885502	-745.402114	0.096930
**C**	-744.882221	-745.396202	0.094167
**B**	-744.886955	-745.400498	0.094367
**BH**	-745.407633	-745.916623	0.107894
**$AH^{\oplus\bullet}$**	-745.183440	-745.694859	0.108356
**$AH^{\ominus\bullet}$**	-745.407633	-745.960636	0.107894
**$BH^{\oplus\bullet}$**	-745.195822	-745.707286	0.107038
**$BH^{\ominus\bullet}$**	-745.453158	-745.966117	0.101073
**$A^{\bullet}$**	-744.686810	-745.199116	0.095939
**$B^{\bullet}$**	-744.757245	-745.266283	0.092773
**$C^{\bullet}$**	-744.758344	-745.267014	0.093881

Table TS25. Energies and energy corrections of stationary points at 1t to 2t isomerization reaction.

Stationary point	ωB97XD/Def2-SVP (MeCN)	ωB97XD/Def2-TZVP (MeCN) single-point	Thermal correction to Gibbs free energy at 25 °C
**AH**	-652.201016	-652.914518	0.176932
**A**	-651.686447	-652.407247	0.165630
**C**	-651.668939	-652.387622	0.162368
**B**	-651.668274	-652.387492	0.160554
**BH**	-652.204509	-652.916901	0.175674
**AH**$^{\oplus\bullet}$	-651.985497	-652.699358	0.175949
**AH**$^{\ominus\bullet}$	-652.204509	-652.949184	0.175674
**BH**$^{\oplus\bullet}$	-651.989406	-652.701836	0.175057
**BH**$^{\ominus\bullet}$	-652.236822	-652.953860	0.168118
**A**$^{\bullet}$	-651.480446	-652.200180	0.164129
**B**$^{\bullet}$	-651.543431	-652.253907	0.160162
**C**$^{\bullet}$	-651.554499	-652.266491	0.161992

Table S26. Energies and energy corrections of stationary points at 1u toward 2uisomerization reaction.

Stationary point	ωB97XD/Def2-SVP (MeCN)	ωB97XD/Def2-TZVP (MeCN) single-point	Thermal correction to Gibbs free energy at 25 °C
**AH**	-745.603760	-746.416544	0.196497
**A**	-745.086263	-745.906923	0.186292
**C**	-745.073598	-745.890716	0.182585
**B**	-745.078868	-745.896097	0.182134
**BH**	-745.612038	-746.423612	0.196074
**AH**$^{\oplus\bullet}$	-745.393835	-745.196026	0.197809
**AH**$^{\ominus\bullet}$	-745.639201	-745.455524	0.188710
**BH**$^{\oplus\bullet}$	-745.402168	-746.214359	0.195922
**BH**$^{\ominus\bullet}$	-745.660368	-746.476726	0.191429
**A**$^{\bullet}$	-744.896703	-745.710010	0.186292
**B**$^{\bullet}$	-744.953292	-745.764564	0.180426
**C**$^{\bullet}$	-744.963349	-745.889841	0.182329

Table TS27. Energies and energy corrections of stationary points at 1u toward 2u' isomerization reaction.

Stationary point	ωB97XD/Def2-SVP (MeCN)	ωB97XD/Def2-TZVP (MeCN) single-point	Thermal correction to Gibbs free energy at 25 °C
**AH**	-745.603760	-746.416544	0.196497
**A**	-745.089663	-745.909640	0.185394
**C**	-745.072278	-745.889841	0.181273
**B**	-745.073219	-745.889790	0.181908
**BH**	-745.606999	-746.419091	0.195251
$\mathbf{AH}^{\oplus\bullet}$	-745.393835	-746.206749	0.196497
$\mathbf{AH}^{\ominus\bullet}$	-745.639201	-746.455524	0.188710
$\mathbf{BH}^{\oplus\bullet}$	-745.396042	-746.208229	0.196263
$\mathbf{BH}^{\ominus\bullet}$	-745.644633	-746.460001	0.187318
$\mathbf{A}^{\bullet}$	-744.881368	-745.676996	0.182529
$\mathbf{B}^{\bullet}$	-744.945118	-745.755644	0.179694
$\mathbf{C}^{\bullet}$	-744.956577	-745.768356	0.181155

Table TS28. Energies and energy corrections of stationary points at 2u toward 2uuisomerization reaction.

Stationary point	ωB97XD/Def2-SVP (MeCN)	ωB97XD/Def2-TZVP (MeCN) single-point	Thermal correction to Gibbs free energy at 25 °C
**AH**	-745.612038	-746.423612	0.196074
**A**	-745.098117	-745.916874	0.184749
**C**	-745.080737	-745.897036	0.181300
**B**	-745.081679	-745.897017	0.180637
**BH**	-745.61524	-746.426141	0.194530
$\mathbf{AH}^{\oplus\bullet}$	-745.402168	-746.214359	0.195922
$\mathbf{AH}^{\ominus\bullet}$	-745.660368	-746.476726	0.191429
$\mathbf{BH}^{\oplus\bullet}$	-745.404532	-746.21529	0.193197
$\mathbf{BH}^{\ominus\bullet}$	-745.666716	-746.483927	0.190162
$\mathbf{A}^{\bullet}$	-744.894399	-745.713062	0.184416
$\mathbf{B}^{\bullet}$	-744.953258	-745.762596	0.179272
$\mathbf{C}^{\bullet}$	-744.963673	-745.773744	0.180591

Table TS29. Energies and energy corrections of stationary points at 2u' toward 2uuisomerization reaction.

Stationary point	ωB97XD/Def2-SVP (MeCN)	ωB97XD/Def2-TZVP (MeCN) single-point	Thermal correction to Gibbs free energy at 25 °C
**AH**	-745.612038	-746.423612	0.196074
**A**	-745.089684	-745.909665	0.184441
**C**	-745.080557	-745.897037	0.180833
**B**	-745.083125	-745.898844	0.181544
**BH**	-745.615240	-746.426141	0.194530
**AH**$^{\oplus\bullet}$	-745.402168	-746.214359	0.195922
**AH**$^{\ominus\bullet}$	-745.660368	-746.476726	0.191429
**BH**$^{\oplus\bullet}$	-745.404532	-746.215290	0.193197
**BH**$^{\ominus\bullet}$	-745.666716	-746.483927	0.190162
**A**$^{\bullet}$	-745.089568	-745.909593	0.184406
**B**$^{\bullet}$	-744.956456	-745.767030	0.179145
**C**$^{\bullet}$	-744.964784	-745.775356	0.180759

Table TS30. Energies and energy corrections of stationary points at S14 isomerization reaction.

Stationary point	ωB97XD/Def2-SVP (MeCN)	ωB97XD/Def2-TZVP (MeCN) single-point	Thermal correction to Gibbs free energy at 25 °C
**AH**	-964.125215	-965.149709	0.362896
**A**	-963.610294	-964.642100	0.351565
**C**	-963.592549	-964.621898	0.348184
**B**	-963.593131	-964.621413	0.347760
**BH**	-964.128858	-965.152681	0.361687
**AH**$^{\oplus\bullet}$	-963.914635	-964.940706	0.361750
**AH**$^{\ominus\bullet}$	-964.128858	-965.179411	0.361687
**BH**$^{\oplus\bullet}$	-963.917541	-964.942387	0.360556
**BH**$^{\ominus\bullet}$	-964.157494	-965.184236	0.353461
**A**$^{\bullet}$	-963.408781	-964.440830	0.349611
**B**$^{\bullet}$	-963.468126	-964.490424	0.346045
**C**$^{\bullet}$	-963.478977	-964.502505	0.347377
